# Synthesis and functionalization of vinyl sulfonimidamides and their potential as electrophilic warheads†

**DOI:** 10.1039/d5sc02420j

**Published:** 2025-06-13

**Authors:** Yu Tung Wong, Charles Bell, Michael C. Willis

**Affiliations:** a Department of Chemistry, University of Oxford, Chemistry Research Laboratory Oxford OX1 3TA UK michael.willis@chem.ox.ac.uk

## Abstract

Covalent inhibitor design is dominated by the use of electrophilic acrylamide warheads. One limitation of acrylamides is that there are limited opportunities to modify their electrophilicity, and hence reactivity, by simple structural changes. Here we show that vinyl sulfonimidamides are effective electophilic groups for reaction with both sulfur- and nitrogen-based biologically relevant nucleophiles. The parent N–H vinyl sulfonimidamides are prepared in a single step from an aryl-ONSO reagent, a vinyl organometallic, and an appropriate amine. Imidic *N*-functionalisation is straightforward, providing a collection of electrophilic fragments of varied reactivity. We demonstrate that the electrophilicity of these new reagents can be modulated by choice of the imidic *N*-substituent, and when this is used in combination with alkene substituents, allows for a reactivity range both above and below that of the parent acrylamide.

## Abstract 2

Vinyl sulfonimidamides are effective electrophilic groups for reaction with both sulfur- and nitrogen-based biologically relevant nucleophiles. We show that the reactivity of these new reagents can be modulated by choice of the imidic *N*-substituent.

## Graphical abstract

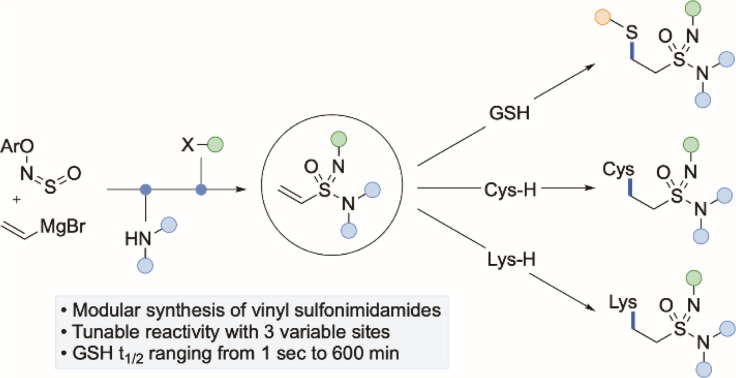

## Introduction

Covalent inhibitors are small molecules that inactivate their target through formation of a covalent linkage. Despite offering the advantages of high potency, long residence time, and decreased drug resistance rate,^1^ these inhibitors have for decades been typically avoided due to potential off-target effects and toxicity concerns. To address these drawbacks, approaches that target non-catalytic nucleophiles in proteins were established, and through rational design of the noncovalent backbone of the inhibitor, maximal inhibition could be achieved while minimising toxicity due to off-target effects.^2^ These advances have led to significant new and renewed interest in developing covalent drugs.^2^ One of the biggest advantages of covalent drugs is that they can target proteins that were considered as ‘undruggable’ due to shallow binding pockets. However, through covalent bond formation, success in targeting these proteins has been achieved.^3^ A recent example is KRAS(G12C), a protein that was considered as undruggable until the recent development of the small molecule inhibitors sotorasib and adagrasib.^4^

**Fig. 1:**
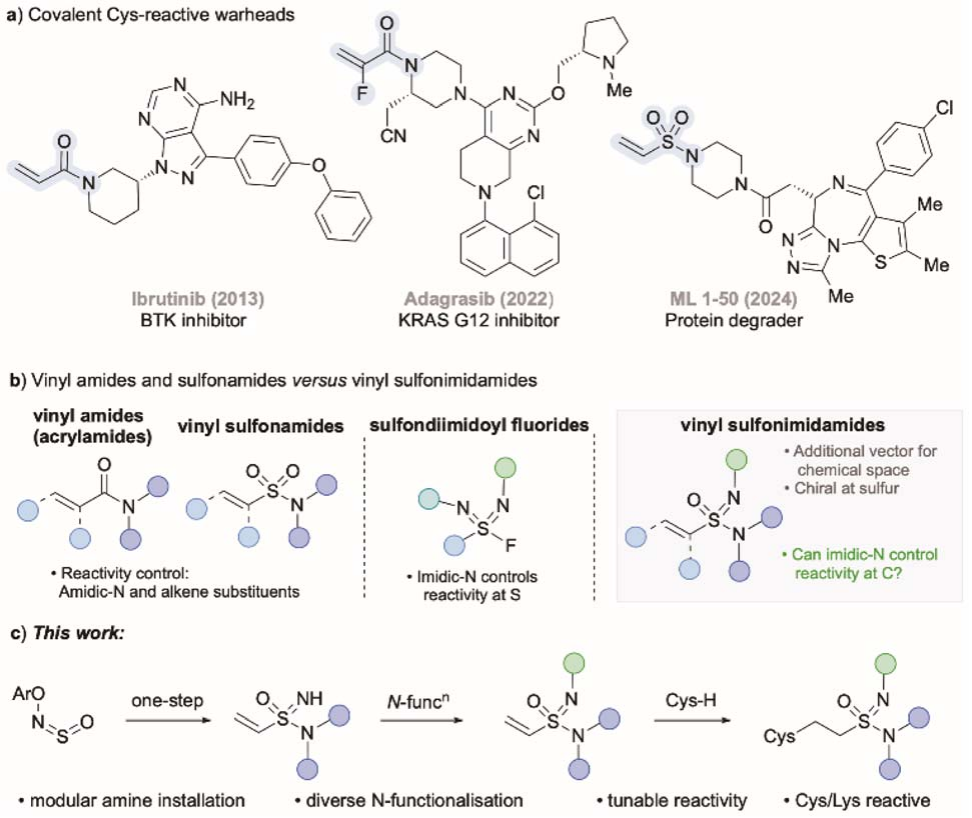
(a) Examples of covalent warheads. (b) Acrylamides, sulfondiimidoyl fluorides, and vinyl sulfonimidamides. (c) This work: synthesis, functionalisation and reactivity of vinyl sulfonimidamides.

Of the 10 000+ covalent inhibitors present in the Covalent Inhibitor Database,^5^ over 50% are classified as having a mechanism of action as Michael addition. Within this group, acrylamides are the most popular Michael acceptors used, and are the reactive warhead in 15 approved drugs and 50 reported covalent inhibitors,^5*b*^ including ibrutinib and afatinib (Fig. 1a), which are both listed as essential medicines by the World Health Organization.^6^ Acrylamide based drugs exhibit a wide range of biological effects, including anticancer,^7^ antiviral,^8^ antibacterial,^9^ anti-inflammatory,^10^ anti-fungal,^11^ and antidiabetic activities.^12^

Despite the success enjoyed with acrylamides as warheads in covalent inhibitors, the structure of the acrylamide group means that there are only limited opportunities to modify their reactivity from simple structural changes. Related to this, the effects on altering the amidic nitrogen substituent, as well as introducing α- or β-substituents on the alkene, on the reactivity of acrylamides with cysteine or glutathione (GSH) nucleophiles have been reported.^13^ Of note in this regard is the KRAS inhibitor adagrasib, featuring a 2-fluoro-substituted acrylamide unit, which was introduced to minimise GSH metabolism and improve bioavailability.^14^

Vinyl sulfonamides have recently been explored as alternatives to acrylamide warheads in covalent inhibitors. These groups are generally more electrophilic, which provides potential advantages for targeting non-catalytic amino acid residues, including cysteines^15^ and lysines.^16^ Their application has emerged in antibody–drug conjugates, chemical probes, and monovalent degraders, with the molecule ML 1–50 being a representative example (Fig. 1a).^17^ As with acrylamides, control of reactivity in vinyl sulfonamides is limited to variation of the amidic *N*-substituent and/or substitution of the alkene. Despite the prevalence of sulfonamides in more than 70 approved drugs,^18^ their mono-aza analogues, sulfonimidamides, have been far less explored,^19^ although they are now common in the medicinal chemistry patent literature.^20^ Potential advantages of these motifs include the presence of a stereogenic center at sulfur,^19^ and that the imidic nitrogen atom is both basic and nucleophilic, thus providing opportunities for functionalization and the potential to tune the physicochemical and biological properties of these molecules.

In a recent study we were able to demonstrate that selection of the imidic *N*-substituent in a series of sulfondiimidoyl fluorides had a marked effect on reactivity with nucleophiles at the sulfur centre (Fig. 1b),^21^ and similar trends have also been seen with sulfonimidoyl fluorides.^22^ Having shown the importance of imidic *N*-substituents on reactivity at sulfur, we speculated that we could translate this concept to vinyl sulfonimidamides, and control reactivity at the β-carbon (Fig. 1b). These new reagents would thus possess a unique additional vector to modulate reactivity and physicochemical properties compared to known warheads such as acrylamides. In addition, the stereogenic center at sulfur could also be expected to impart selectivity with chiral nucleophiles. There are a handful of alkenyl sulfonimidamides in the literature,^23^ with the first vinyl example reported by our laboratory in 2020.^24^ Additionally, a small collection of vinyl sulfonimidamides has been prepared by Bull and Armstrong using a sequence starting from β-alkoxydisulfides;^25^ these studies showcased the chemical and stereochemical stability of vinyl sulfonimidamides, although no reactivity studies were performed. The stability of sulfonimidamides in general, has been touched on in several publications.^19,26^

## Results and discussion

**Scheme 1:**
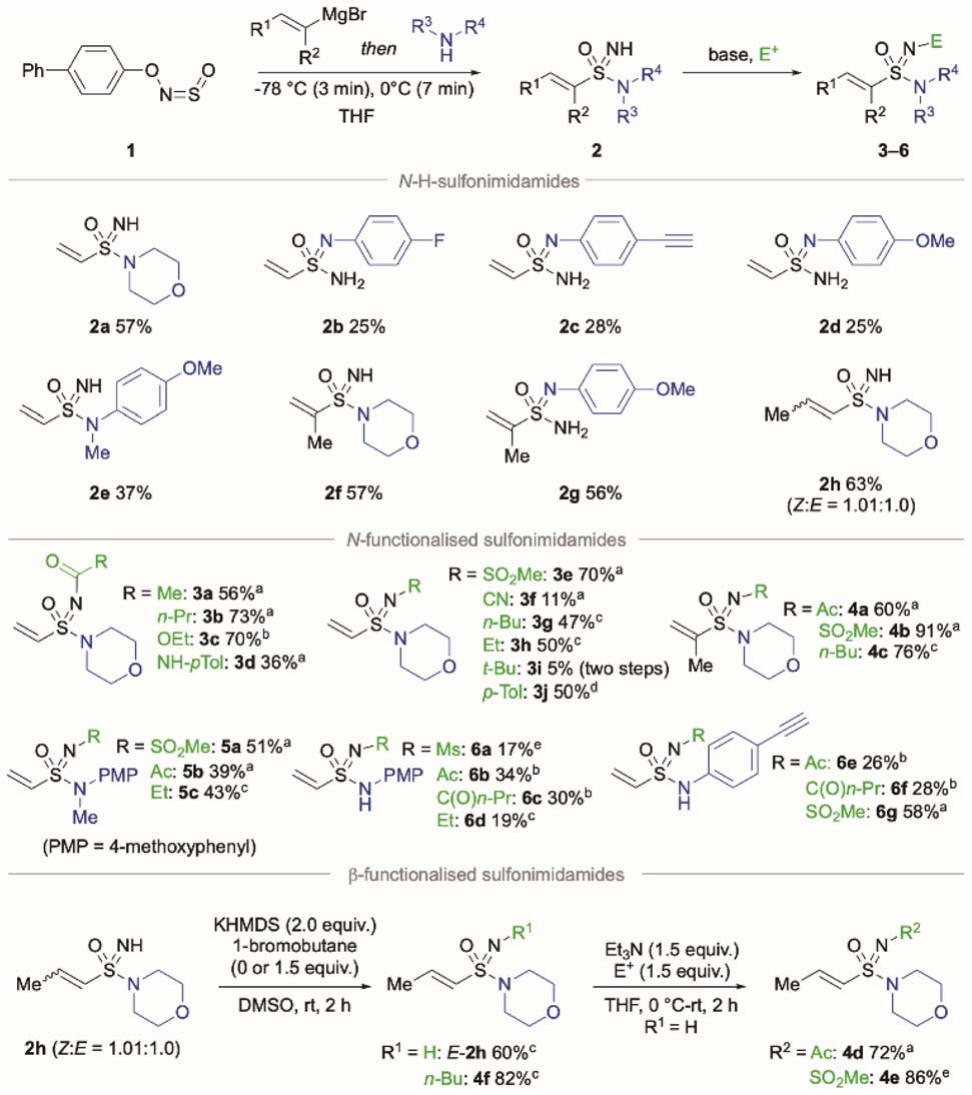
Synthesis of N–H- and *N*-functionalised sulfonimidamides from BiPhONSO. Conditions for N–H sulfonimidamides: BiPhONSO (1.0 equiv.), alkenyl Grignard (1.0 equiv.), THF, −78 °C, 3 min then amine (1.2–1.5 equiv.), −78 °C to 0 °C, 7 min. Functionalization of vinylsulfonimidamides: 1.0 equiv. of sulfonimidamide was used in all cases. ^*a*^Et_3_N (1.5 equiv.), E^+^ (1.1–1.5 equiv.), THF, 0 °C to rt, 2–20 h; ^*b*^pyridine (1.5–1.8 equiv.), E^+^ (1.1–1.5 equiv.), THF, 0 °C to rt, 3–16 h; ^*c*^KHMDS (2.0 equiv.), E^+^ (0–1.5 equiv.), DMSO, rt, 2 h; ^*d*^*p*-tolyl boronic acid (2.3 equiv.), Cu(MeCN)_4_BF_4_ (15 mol%), DMF, rt, O_2_, 24 h; ^*e*^NaH (1.2 equiv.), MsCl (1.1 equiv.), THF, 0 °C to rt, 18 h.

Using a slightly modified procedure to the earlier method reported by our laboratory,^24*a*^ vinyl sulfonimidamides 2 were prepared in one-pot using a combination of the commercially available hydroxysulfinylamine BiPhONSO (1),^27^ vinylmagnesium bromide, and a range of amines (Scheme 1), allowing the rapid assembly of the desired N–H sulfonimidamides, with modular installation of the amidic N-unit. With a series of N–H sulfonimidamides in hand, fuinctionalisation of the imidic N–H was achieved in a straightforward way. For example, treatment of morpholine derivative (2a) with base and an appropriate electrophile provided access to a variety of *N*-functionalised products including acyl (3a–b), carbonate (3c), urea (3d), sulfonyl (3e), cyano (3f) and alkyl (3g–3h) sulfonimidamides. A Cu-mediated Chan–Lam coupling was used to access *N*-aryl derivative (3j).^28^ Access to the *N-tert*-butyl derivative 3i required an alternative route proceeding from *tert*-butylsulfinylamine.^29^ Primary aniline derived compounds (2c–2d) were more challenging to functionalise, with the presence of two potential reactive N-atoms resulting in lower yields. Of note, when a mixture of geometrical isomers of β-methyl substituted sulfonimidamide (2h) was subjected to basic conditions, only the (*E*)-isomer of the product was obtained when using 1-bromobutane as the electrophile (4f). A similar isomerisation could be exploited to obtain geometrically pure (*E*)-2h; simply treating the geometrical mixture of 2h with KHMDS for 2 h at room temperature allowed the (*E*)-isomer to be obtained in 60% yield. Related base-catalysed geometric isomerisations have previously been described for sulfides, sulfoxides and sulfones, but has yet to be reported for sulfonimidamides.^30^ With a single isomer of 2h obtained, functionalisation of the imidic nitrogen was possible through treatment with triethylamine and either acetyl chloride (4d) or methanesulfonyl chloride (4e), with no isomerisation observed.

**Scheme 2:**
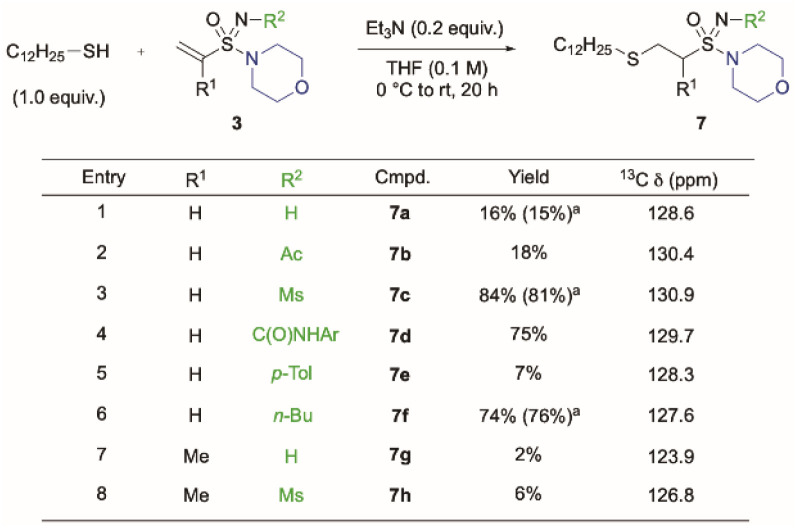
Conjugate addition reactions of alkenyl sulfonimidamides 3 with 1-dodecanethiol (full details in ESI†). ^*a*^In the presence of 1.0 equiv. of acrylamide 8.

With a broad range of *N*-functionalised sulfonimidamides available, reactivity studies toward conjugate addition of sulfur-based nucleophiles were carried out using 1-dodecanethiol (Scheme 2).^31^ The influence of the nitrogen substituent was pronounced in these reactions, with yields ranging from 7–84% for the vinyl examples. As had been previously observed with acrylamides, the ^13^C NMR shifts of the terminal vinyl-carbon provides a reasonable prediction of reactivity.^13*b*^ This is typified by *N*-sulfonyl sulfonimidamide 3e, which gave the highest yield of 84%. *N*-Butyl example 7f is an outlier in these reactions, with greatly enhanced reactivity observed under these reaction conditions, and we speculate that this may be due to the enhanced basicity of the imidic nitrogen. As expected, α-methyl substituted sulfonimidamides (7g, 7h) showed attenuated reactivity (2–6%), and this is in agreement with previous studies on substituted acrylamides.^13*c*^ To benchmark the reactivity of these new *N*-functionalised sulfonimidamides, competition reactions were performed in the presence of an acrylamide analogue, 4-acryloylmorpholine (8, 1.0 equiv.). Importantly, in these cases the yield of the obtained sulfonimidamide addition product was consistent (yields in parenthesis, 7a, 7c and 7f), demonstrating the enhanced reactivity of this functionality relative to acrylamides.

**Scheme 3:**
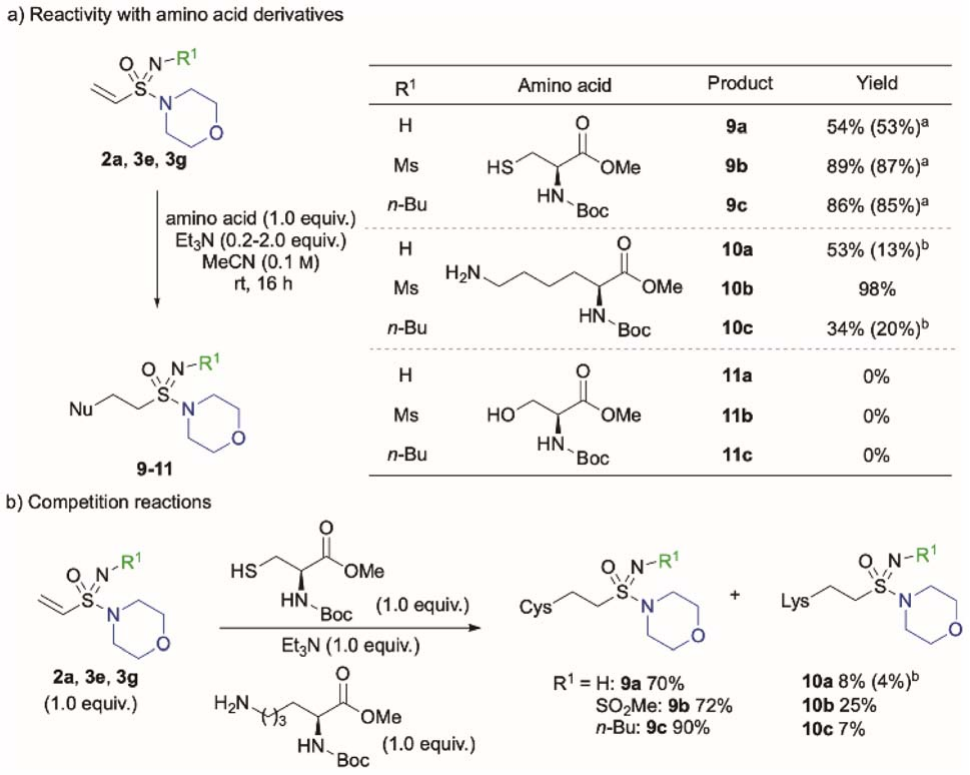
Conjugate addition reaction of vinyl sulfonimidamides 2–3 with amino acid derivatives. Reaction conditions: cysteine: Boc-(l)-Cys-OMe (1.0 equiv.), Et_3_N (1.0 equiv.), MeCN, rt, 16 h; lysine: Boc-(l)-Lys-OMe-HCl (1.0 equiv.), Et_3_N (2.0 equiv.), MeCN, rt, 16 h; serine: Boc-(l)-Ser-OMe (1.0 equiv.), Et_3_N (1.0 equiv.), MeCN, rt, 16 h. Isolated yields reported. ^*a*^Et_3_N (0.2 equiv.) was used; ^*b*^yield for double addition product.

We next examined the reactivity of vinyl sulfonimidamides against a series of protected amino acid derivatives, using the imidic N–H, *N*-Ms and *N-n*-Bu analogues as the electrophilic components (Scheme 3a).^16^ As expected, reactivity with the cysteine derivative was similar to that observed with the simple thiol, with the *N*-Ms substrate 3e, providing the corresponding addition product in high yield (9b, 89%). The *N-n*-Bu (3g) substrate was comparable (86%), while the N–H substrate 2a showed a lower reactivity (54%). These reactions gave consistent results when performed with stoichiometric or sub-stoichiometric quantities of triethylamine (0.2 equiv., yields in parenthesis). Vinyl sulfonimidamides also reacted readily with a lysine derivative; the *N*-Ms substrate (3e) providing the adduct in an excellent 98% yield (98%), while the N–H (2a) and *N-n*-butyl (3g) examples resulted in mixtures of mono- (10a and 10c) and bis-addition products. All of the substrates were unreactive towards Boc-(l)-Ser-OMe, with no desired products being observed (11) and only starting material remaining.

Given the reactivity observed with the cysteine and lysine derivatives, we next performed a series of competition experiments to probe this further. The same three vinyl sulfonimidamide derivatives (2a, 3e, 3g) were combined with a 1 : 1 mixture of protected cysteine and lysine nucleophiles and the yields of the conjugate addition products were measured (Scheme 3b). The N–H substrate reacted preferentially with the cysteine derivative (70%), with only a small amount (8%) of the lysine adduct being isolated. The more electrophilic *N*-Ms substrate still formed the cysteine adduct as the major product, but the proportion of the lysine adduct was increased to 25%. Finally, the *N*-Bu sulfonimidamide displayed good selectivity for cysteine, with only 7% of the lysine adduct being formed.

**Scheme 4:**
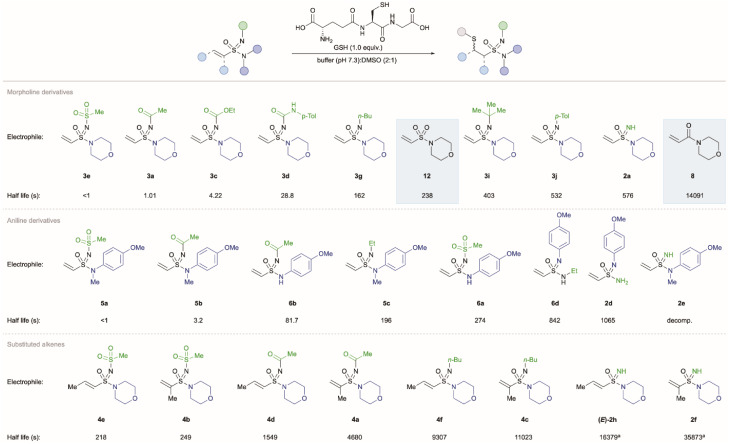
Kinetic studies of vinyl sulfonimidamides with glutathione. ^*a*^6.0 equiv. GSH used.

The reactivity of the vinyl sulfonimidamides was next examined under biologically relevant conditions using glutathione (GSH) as the nucleophile at pH 7.3 (Scheme 4).^32^ The half-life (*t*_1/2_) was determined using ^1^H NMR analysis, with the disappearance of the vinyl group monitored as a function of time (full experimental details are provided in the ESI†). Control experiments using acrylamide 8 and sulfonimidamide 2a confirmed only GSH-dependent reactivity. Due to the rapid reaction rates, the half-life for the majority of substrates was calculated using second-order kinetics. However, the half-life of unfunctionalized α-Me and β-Me-substituted sulfonimidamide derivatives were determined with pseudo-first-order kinetics using six equivalents of glutathione. In order to benchmark the reactivity of the vinyl sulfonimidamides using these reaction conditions, 4-acryloylmorpholine 8 and vinyl sulfonamide 12 were included as substrates. The half-life measured for the reaction of GSH and 4-acryloylmorpholine 8 (3.91 h) under these conditions is faster than that reported by Bauer and co-workers^33^ (13.8 h, 1 mM electrophile, 10 mM GSH, 70 mM phosphate buffer (pH = 7.4), 30% MeCN at 37 °C) and this is likely due to the differences in experimental parameters.

The unfunctionalized N–H sulfonimidamide 2a has a half-life of 576 seconds, showcasing the vastly enhanced reactivity compared with the acrylamide analogue (8, 14 091 seconds), although this is slower than the corresponding sulfonamide (12, 238 seconds). Incorporating an electron-withdrawing group on the imidic nitrogen atom of the sulfonimidamides results in significant increases in reactivity; in all cases, reactions with GSH exhibit extremely fast kinetics with half-life measurements of <1 minute. This rate of sulfonimidamide conjugation compares favourably even to the fastest cysteine conjugation reactions reported, such as those using iodoacetamide, maleimide or chlorooxime electrophiles.^34^ The half-life for mesylated derivative 3e was too fast to be determined. Reactivity of the acylated sulfonimidamide 3a could be measured, with a half-life of 1.01 seconds, which to the best of our knowledge is the shortest half-life recorded in reaction with GSH,^33^ showcasing the enhanced reactivity of these Michael-type acceptors. Both carbamate 3c (*t*_1/2_ = 4.22 s) and urea 3d (*t*_1/2_ = 28.8 s) demonstrated significantly enhanced reactivities compared to that of the sulfonamide 12. For sulfonimidamides bearing N-electron-withdrawing groups, there was a positive correlation between the rate of reaction and increase in the β-carbon ^13^C NMR shift (see ESI†). The exceptionally high reactivity of these compounds with sulfur-based nucleophiles suggests potential application in quantification of cysteine in proteomic studies and the labelling of peptides.^35^

Substrates featuring electron donating *N*-alkyl groups were also studied. Reaction of *N-n*-butyl substituted sulfonimidamide 3g proceeded at a comparably slower rate (*t*_1/2_ = 162 s), although this is still enhanced relative to sulfonamide 12. Substrate 3i, featuring the electron-donating and sterically demanding *tert*-butyl substituent, showed reduced reactivity (*t*_1/2_ = 403 s). *N*-Aryl derivative 3j had similar reaction kinetics (*t*_1/2_ = 532 s) compared to the N–H derivative 2a. The ESI† contains data for additional *N*-substituted vinyl sulfonimidamides (Table S3†).

Birkholz and co-workers have shown that substituents on the alkene of acrylamides affects reaction rates with GSH.^13*d*^ We examined this in the context of α- and β-methylated alkenyl sulfonimidamide derivatives with varying imidic *N*-substituents. Analogous to acrylamides, methyl substitution at the α-position (4a–c) greatly reduced reactivity with GSH relative to the unsubstituted vinyl derivatives. For example, *N*-Ms (4b, *t*_1/2_ = 249 s), *N*-Ac (4a, *t*_1/2_ = 4680 s) and *N-n*-Bu (4c, *t*_1/2_ = 11 023 s) derivatives all reacted orders of magnitude slower than their vinyl counterparts, and are more comparable to sulfonamide and acrylamide warheads in terms of reactivity. As expected, methyl substitution at the β-position (4d–f) similarly reduces the rate of reaction with GSH (*t*_1/2_ = 218 to 9307 s). This can be attributed to a combination of increased steric hindrance at the site of nucleophilic addition and deactivation of the electrophile through hyperconjugation. In all examples, the β-methyl sulfonimidamides are more reactive than α-methyl derivatives.

The final parameter explored was the effect of the amidic *N*-substituent on reactivity. *N*-Methylanisidine derived sulfonimidamides (5a–c) showed reactivity similar to the corresponding morpholine derivatives in their reactions with GSH. However, primary anisidine derived sulfonimidamides (6) displayed reduced reactivity relative to tertiary sulfonimidamides (5). The order of reactivities based on imidic *N*-substitution was also altered for this class of substrates; Ac > Ms > Et > NH. This lower reactivity (and scrambling of substituent effects) is in contrast to what has been observed using acrylamide substrates,^13*a*^ and is likely due to tautomeric isomerisation that is observed with *N*-aryl sulfonimidamides.

## Conclusions

We have shown that vinyl sulfonimidamides are available in a single step, and that their conversion to a variety of imidic *N*-functionalised products is straightforward. These new electrophilic fragments show good reactivity with both cysteine and lysine derived nucleophiles. Kinetic analysis of their reactions with glutathione demonstrates exceptional reactivity, relative to the corresponding sulfonamide and acrylamide derivatives, and that this reactivity is dependent on the identity of the imidic *N*-substituent. By tuning the imidic *N*-substituent in combination with alkene substitution, it is possible to achieve reactivity either above or below that of the corresponding acrylamides. Given the wide range of reactivities that are possible, combined with their modular assembly, we anticipate that vinyl sulfonimidamides should be of broad utility in medicinal and polymer applications.

## Author contributions

Y. T. W., C. B. and M. C. W. designed the study, Y. T. W. conducted the experiments on the synthesis of starting materials, optimization of conditions, substrate exploration, and product transformation. Y. T. W. performed the NMR experiments and calculations. M. C. W. directed the project. All authors wrote the manuscript.

## Conflicts of interest

The authors declare no conflicts of interest.

## Supplementary Material

SC-016-D5SC02420J-s001

## Data Availability

All data including experimental and analytical details are in the ESI.†

## References

[cit1] Singh J., Petter R. C., Baillie T. A., Whitty A. (2011). The resurgence of covalent drugs. Nat. Rev. Drug Discovery.

[cit2] Mehta N. V., Degani M. S. (2023). The expanding repertoire of covalent warheads for drug discovery. Drug Discovery Today.

[cit3] Guo Y., shuai W., Tong A., Wang Y. (2024). Advanced technologies for screening and identifying covalent inhibitors. TrAC, Trends Anal. Chem..

[cit4] Canon J., Rex K., Saiki A. Y., Mohr C., Cooke K., Bagal D., Gaida K., Holt T., Knutson C. G., Koppada N., Lanman B. A., Werner J., Rapaport A. S., San Miguel T., Ortiz R., Osgood T., Sun J. R., Zhu X., McCarter J. D., Volak L. P., Houk B. E., Fakih M. G., O'Neil B. H., Price T. J., Falchook G. S., Desai J., Kuo J., Govindan R., Hong D. S., Ouyang W., Henary H., Arvedson T., Cee V. J., Lipford J. R. (2019). The clinical KRAS(G12C) inhibitor AMG 510 drives anti-tumour immunity. Nature.

[cit5] Du H., Gao J., Weng G., Ding J., Chai X., Pang J., Kang Y., Li D., Cao D., Hou T. (2021). CovalentInDB: a comprehensive database facilitating the discovery of covalent inhibitors. Nucleic Acids Res..

[cit6] Model List of Essential Medicines, 2024, https://list.essentialmeds.org/

[cit7] Zhou W., Li H.-b., Xia C.-n., Zheng X.-m., Hu W.-x. (2009). The synthesis and biological evaluation of some caffeic acid amide derivatives: E-2-Cyano-(3-substituted phenyl)acrylamides. Biorg. Med. Chem. Lett..

[cit8] Nitsche C., Steuer C., Klein C. D. (2011). Arylcyanoacrylamides as inhibitors of the Dengue and West Nile virus proteases. Bioorg. Med. Chem..

[cit9] Fu J., Cheng K., Zhang Z.-m., Fang R.-q., Zhu H.-l. (2010). Synthesis, structure and structure–activity relationship analysis of caffeic acid amides as potential antimicrobials. Eur. J. Med. Chem..

[cit10] Sato I., Morihira K., Inami H., Kubota H., Morokata T., Suzuki K., Ohno K., Iura Y., Nitta A., Imaoka T., Takahashi T., Takeuchi M., Ohta M., Tsukamoto S.-i. (2009). Synthesis, biological evaluation, and metabolic stability of acrylamide derivatives as novel CCR3 antagonists. Bioorg. Med. Chem..

[cit11] Xu L.-z., Xu Z.-j., Zhang G.-s., Zhou K., Zhai Z.-w. (2008). Synthesis, Characterization
and Biological Activities of Novel Acrylamide Compounds. Chem. Res. Chin. Univ..

[cit12] Onda K., Shiraki R., Yonetoku Y., Momose K., Katayama N., Orita M., Yamaguchi T., Ohta M., Tsukamoto S. (2008). Synthesis and pharmacological evaluation of bis-3-(3,4-dichlorophenyl)acrylamide derivatives as glycogen phosphorylase inhibitors. Bioorg. Med. Chem..

[cit13] Flanagan M. E., Abramite J. A., Anderson D. P., Aulabaugh A., Dahal U. P., Gilbert A. M., Li C., Montgomery J., Oppenheimer S. R., Ryder T., Schuff B. P., Uccello D. P., Walker G. S., Wu Y., Brown M. F., Chen J. M., Hayward M. M., Noe M. C., Obach R. S., Philippe L., Shanmugasundaram V., Shapiro M. J., Starr J., Stroh J., Che Y. (2014). Chemical and Computational Methods for the Characterization of Covalent Reactive Groups for the Prospective Design of Irreversible Inhibitors. J. Med. Chem..

[cit14] Fell J. B., Fischer J. P., Baer B. R., Blake J. F., Bouhana K., Briere D. M., Brown K. D., Burgess L. E., Burns A. C., Burkard M. R., Chiang H., Chicarelli M. J., Cook A. W., Gaudino J. J., Hallin J., Hanson L., Hartley D. P., Hicken E. J., Hingorani G. P., Hinklin R. J., Mejia M. J., Olson P., Otten J. N., Rhodes S. P., Rodriguez M. E., Savechenkov P., Smith D. J., Sudhakar N., Sullivan F. X., Tang T. P., Vigers G. P., Wollenberg L., Christensen J. G., Marx M. A. (2020). Identification of the Clinical Development Candidate MRTX849, a Covalent KRAS(G12C) Inhibitor for the Treatment of Cancer. J. Med. Chem..

[cit15] Turnbull A. P., Ioannidis S., Krajewski W. W., Pinto-Fernandez A., Heride C., Martin A. C. L., Tonkin L. M., Townsend E. C., Buker S. M., Lancia D. R., Caravella J. A., Toms A. V., Charlton T. M., Lahdenranta J., Wilker E., Follows B. C., Evans N. J., Stead L., Alli C., Zarayskiy V. V., Talbot A. C., Buckmelter A. J., Wang M., McKinnon C. L., Saab F., McGouran J. F., Century H., Gersch M., Pittman M. S., Marshall C. G., Raynham T. M., Simcox M., Stewart L. M. D., McLoughlin S. B., Escobedo J. A., Bair K. W., Dinsmore C. J., Hammonds T. R., Kim S., Urbe S., Clague M. J., Kessler B. M., Komander D. (2017). Molecular basis of USP7 inhibition by selective small-molecule inhibitors. Nature.

[cit16] Chen H., Huang R., Li Z., Zhu W., Chen J., Zhan Y., Jiang B. (2017). Selective lysine modification of native peptides *via* aza-Michael addition. Org. Biomol. Chem..

[cit17] Lim M., Cong T. D., Orr L. M., Toriki E. S., Kile A. C., Papatzimas J. W., Lee E., Lin Y., Nomura D. K. (2024). DCAF16-Based Covalent Handle for the Rational Design of Monovalent Degraders. ACS Cent. Sci..

[cit18] Scott K. A., Njardarson J. T. (2018). Analysis of US FDA-Approved Drugs Containing Sulfur Atoms. Top. Curr. Chem..

[cit19] Chinthakindi P. K., Naicker T., Thota N., Govender T., Kruger H. G., Arvidsson P. I. (2017). Sulfonimidamides in Medicinal and Agricultural Chemistry. Angew. Chem., Int. Ed..

[cit20] (a) Chowdhury S. , Dehnhardt C. M., Focken T., Grimwood M. E., Hemeon I. W., Mckerrall S. and Sutherlin D., Substituted benzamides and methods of use thereof, WO2017/172802A1, 2017

[cit21] Ding M., Bell C., Willis M. C. (2024). The Modular Synthesis of Sulfondiimidoyl Fluorides and their Application to Sulfondiimidamide and Sulfondiimine Synthesis. Angew. Chem., Int. Ed..

[cit22] Zeng D., Ma Y., Deng W.-P., Wang M., Jiang X. (2022). Divergent sulfur(VI) fluoride exchange linkage of sulfonimidoyl fluorides and alkynes. Nat. Syn..

[cit23] Davies T. Q., Hall A., Willis M. C. (2017). One-Pot, Three-Component Sulfonimidamide Synthesis Exploiting the Sulfinylamine Reagent N-Sulfinyltritylamine, TrNSO. Angew. Chem., Int. Ed..

[cit24] Davies T. Q., Tilby M. J., Ren J., Parker N. A., Skolc D., Hall A., Duarte F., Willis M. C. (2020). Harnessing Sulfinyl Nitrenes: A Unified One-Pot Synthesis of Sulfoximines and Sulfonimidamides. J. Am. Chem. Soc..

[cit25] Craven G. B., Briggs E. L., Zammit C. M., McDermott A., Greed S., Affron D. P., Leinfellner C., Cudmore H. R., Tweedy R. R., Luisi R., Bull J. A., Armstrong A. (2021). Synthesis and Configurational Assignment of Vinyl Sulfoximines and Sulfonimidamides. J. Org. Chem..

[cit26] Frings M., Bolm C., Blum A., Gnamm C. (2017). Sulfoximines from a Medicinal Chemist's Perspective: Physicochemical and *in vitro* Parameters Relevant for Drug Discovery. Eur. J. Med. Chem..

[cit27] BiPhONSO is available from Cortex Organics, www.cortexorganics.com

[cit28] Vantourout J. C., Li L., Bendito-Moll E., Chabbra S., Arrington K., Bode B. E., Isidro-Llobet A., Kowalski J. A., Nilson M. G., Wheelhouse K. M. P., Woodard J. L., Xie S., Leitch D. C., Watson A. J. B. (2018). Mechanistic Insight Enables Practical, Scalable, Room Temperature Chan–Lam N-Arylation of N-Aryl Sulfonamides. ACS Catal..

[cit29] Yong H. K., Jai M. S. (1985). New facile synthesis of *N*-sulfinylamine derivatives using *N*,*N*′-sulfinylbisimidazole and *N*-(chlorosulfinyl)imidazole. Tetrahedron Lett..

[cit30] Kimmelma R. (1993). Structure Stability Relationships in Unsaturated Sulfur Compounds. III. A Thermodynamic and 13C NMR Spectroscopic Study of the Conformations of Vinyl Sulfones. Acta Chem. Scand..

[cit31] Wu C. H. , Shue J. H., Chen C. Y., Chien Y. C., Chen S. C., Pan C. H. and Huang C. Y., Pharmaceutical uses of sulfur containing compound, US2016009642A1, 2016

[cit32] Krezel A., Bal W. (2004). A formula for correlating pKa values determined in D_2_O and H_2_O. J. Inorg. Biochem..

[cit33] Palkowitz M. D., Tan B., Hu H., Roth K., Bauer R. A. (2017). Synthesis of Diverse *N*-Acryloyl Azetidines and Evaluation of Their Enhanced Thiol Reactivities. Org. Lett..

[cit34] Chen F. J., Zheng M., Nobile V., Gao J. (2022). Fast and Cysteine-Specific Modification of Peptides, Proteins and Bacteriophage Using Chlorooximes. Chem.–Eur. J..

[cit35] Virag D., Schlosser G., Borbely A., Gellen G., Papp D., Kaleta Z., Dalmadi-Kiss B., Antal I., Ludanyi K. (2024). A Mass Spectrometry Strategy for Protein Quantification Based on the Differential Alkylation of Cysteines Using Iodoacetamide and Acrylamide. Int. J. Mol. Sci..

