# Supplementary material for: Synthesis and functionalization of vinyl sulfonimidamides and their potential as electrophilic warheads
Source: Chem Sci. 2025 Jun 13;16(28):12860–6. doi: 10.1039/d5sc02420j (PMC12163725; doi:10.1039/d5sc02420j)
Supplement: SC-016-D5SC02420J-s001 [file SC-016-D5SC02420J-s001.pdf]

Supplementary Information (SI) for Chemical Science.
This journal is © The Royal Society of Chemistry 2025

Electronic Supplementary Information

for

Synthesis and functionalization of vinyl sulfonimidamides and their potential as electrophilic warheads

Yu Tung Wong,[a] Charles Bell,[a] and Michael C. Willis*[a]

[a]Department of Chemistry, University of Oxford, Chemistry Research Laboratory, Oxford, OX1 3TA, UK; E-mail: michael.willis@chem.ox.ac.uk

Table of contents

1. General Considerations 2

2 Synthetic Procedures and Characterisation data 3

2.1 Preparation of BiPhONSO 3

2.2 Preparation of *tert*-Butyl *N*-sulfinylamine (SI-3) 5

2.3 Preparation of vinyl *N*-H sulfonimidamides 6

2.4 Synthesis of vinyl sulfonamide 11

2.5 *N*-functionalization of sulfonimidamides 12

2.6 Conjugate addition with dodecanethiol 31

2.7 Procedure for competition reaction with 1-dodecanethiol 39

2.8 Procedure for conjugate addition reactions with amino acids 40

2.9 Procedure for competition reactions of Lys- and Cys-derivatives 45

2.10 Procedure for determination of half-life for the conjugate addition of glutathione 45

3. References 64

4. NMR spectra 65

1. General Considerations

Reactions were performed under an inert nitrogen atmosphere with anhydrous solvent unless otherwise stated. All glassware was oven dried at 200 °C and cooled to room temperature under positive pressure of nitrogen. Reactions were monitored by HPLC/TLC using aluminium backed silica plates. Plates were visualized under ultraviolet light (254 nm) and/or staining with $KMnO_4$. The cooling of reaction mixture to -78 °C was achieved using a dry ice-acetone bath.

Reagents were purchased from Sigma-Aldrich Chemical Co. Ltd., Fisher Scientific, Alfa Aesar, Acros Organics Ltd., Fluorochem Ltd., Fluka™ and were used as supplied. Grignard reagents and *n*-butyllithium were titrated against salicylaldehyde phenylhydrazone.[1]

Flash chromatography was carried out using Geduran® Si 60, 40-63 micron silica gel. 'Petrol' refers to the fraction of light petroleum ether with boiling point in the range of 40-60 °C.

^{1}H NMR spectra were obtained on Bruker AVIII400 (400 MHz), Bruker AVIIIHD 500 (500MHz) and Bruker NEO 600 spectrometer using the residual solvent as an internal standard. ^{13}C-NMR spectra were obtained on a Bruker AVIII 400 (101 MHz), Bruker AVIIIHD 500 (126 MHz) and Bruker NEO 600 (151 MHz) spectrometer using the residual solvent as an internal standard. ^{19}F-NMR spectra were obtained on a Bruker AVIII400 (376 MHz) or Bruker AVIIIHD 500 (476 MHz) spectrometer. Chemical shifts (δ) were reported in parts per million (ppm) with the multiplicities of the spectra reported as following: singlet (s); broad singlet (br. s); doublet (d); triplet (t); quartet (q); pentet (pent); sextet (sext); heptet (hept); multiplet (m); apparent (app.). Coupling constants (*J*) were given in Hertz (Hz) and rounded to the nearest 0.5 Hz.

Low resolution ESI mass spectra were recorded on a Waters LCT Premier spectrometer. High resolution mass spectrometry measurements were recorded on BioAccord LC-MS, performed on ACQUITY RDa mass spectrometer an ACQUITY I-Class PLUS UPLC System (Waters, Milford, MA, USA) coupled to an ACQUITY RDa mass spectrometer (Waters, Milford, MA, USA) equipped with an ESI probe or Bruker Daltronics MicroTOF (ESI) spectrometer by the internal service at Chemistry Research Laboratory, University of Oxford. Samples for mass spectra were prepared as 10 μg/mL solution in MeCN or MeOH (LRMS, HRMS-ESI, HRMS-LCMS).

Infrared spectra were recorded as thin films on a Bruker Tensor 27 FT-IR spectrometer.

Melting points were determined using a Stuart Scientific Melting Point Apparatus SMP1 and are reported uncorrected.

2 Synthetic Procedures and Characterisation data

2.1 Preparation of BiPhONSO

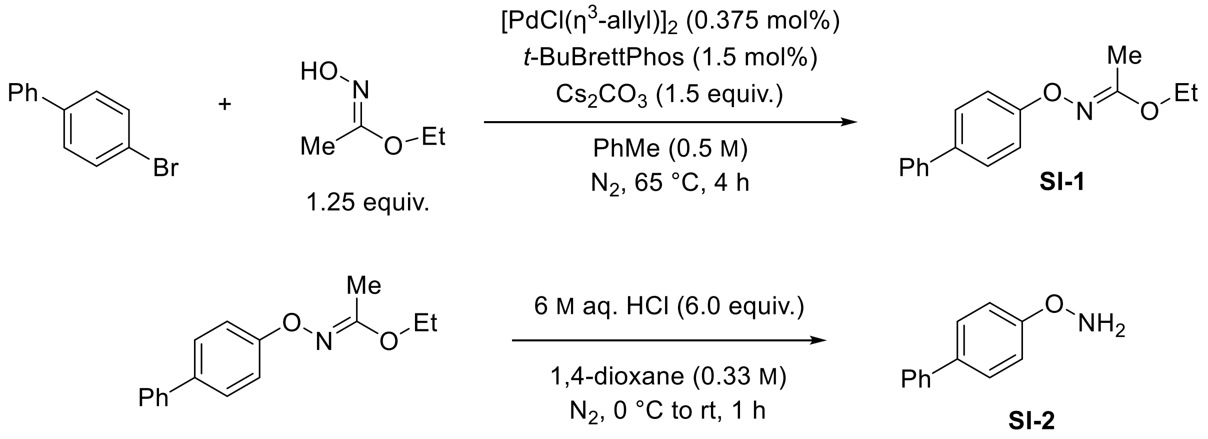

***O*-([1,1'-biphenyl]-4-yl)hydroxylamine**

Procedures adapted from T. J. Maimone, S. L. Buchwald, *J. Am. Chem. Soc*. 2010, **132**, 9990-9991.[2]

4-Bromobiphenyl (5.83 g, 25.0 mmol, 1.0 equiv.), [PdCl(η3-allyl)]$_2$ (34.3 mg, 0.094 mmol, 0.375 mol%), *t*-BuBrettPhos (182 mg, 0.375 mmol, 1.5 mol%) and Cs_2CO_3 (12.2 g, 37.5 mmol, 1.5 equiv.) were added to an oven dried round-bottomed flask. The flask was evacuated and back-filled with nitrogen three times. Anhydrous, degassed toluene (50 mL, 0.5 M) and ethyl acetohydroxamate (3.10 mL, 31.0 mmol, 1.25 equiv.) were added and the resultant solution was placed in a pre-heated oil bath at 65 °C and stirred for 4 hours. HPLC of the crude mixture showed complete conversion to product. The reaction mixture was then diluted with EtOAc, filtered through a short pad of silica and concentrated *in vacuo*. The crude mixture was used directly in the next reaction without further purification.

The flask containing the crude mixture was evacuated and back-filled with nitrogen three times and the crude mixture was dissolved in anhydrous, degassed 1,4-dioxane (75 mL, 0.33 M) and cooled to 0 °C. 6.0 M aqueous HCl (25 mL, 6 equiv.) was added dropwise. The resultant solution was stirred at 0 °C for 5 min then warmed to room temperature and stirred for 1 hour. The reaction mixture was diluted with Et_2O (250 mL) and transferred to a separating

funnel. The organic layer was washed with 1 M aqueous NaOH (300 mL) and brine (250 mL), dried over anhydrous Na_2SO_4, filtered and concentrated *in vacuo*. The crude mixture was purified by flash chromatography (*n*-hexane/EtOAc 4:1) to give the product as a white solid (3.94 g, 85% over 2 steps).

IR (thin film, ν_{max}/cm^{-1}) 3312, 3056, 3035, 1608, 1483, 1141, 833, 757, 688; **δ_H** (400 MHz, $CDCl_3$) 7.59-7.54 (m, 2H), 7.54-7.51 (m, 2H), 7.45-7.38 (m, 2H), 7.35-7.28 (m, 1H), 7.24-7.19 (m, 2H), 5.89 (br. s, 2H); **δ_C** (100 MHz, $CDCl_3$) 161.0, 141.1, 134.4, 128.9, 128.1, 126.9, 126.8, 113.6; **LRMS** *m/z* (ESI^+) $[M+H]^+$ 186.077; **HRMS** (ESI^+, *m/z* calculated for $[C_{12}H_{12}NO]^+$ 186.0913 ($[M+H]^+$), found 186.0914. Data is consistent with the literature. [2]

BiPhONSO

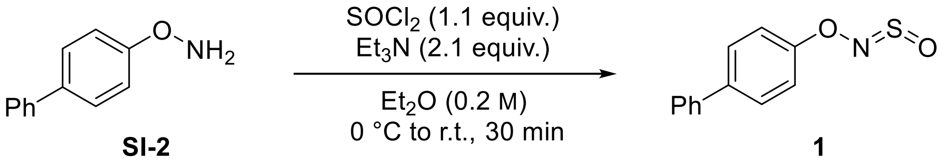

Procedure adapted from T. Q. Davies, M. J. Tilby, J. Ren, N. A. Parker, D. Skolc, A. Hall, F. Duarte, M. C. Willis, *J. Am. Chem. Soc*. 2020, **142**, 15445-15453.[3]

O-([1,1'-biphenyl]-4-yl)hydroxylamine (1.85 g, 10.0 mmol, 1.0 equiv.) was added to an oven dried round-bottom flask and evacuated and back-filled with nitrogen three times. The hydroxylamine was dissolved in anhydrous, degassed Et_2O (50 mL, 0.20 M) and cooled to 0 °C. Anhydrous triethylamine (2.90 mL, 21.0 mmol, 2.1 equiv.) was added. Thionyl chloride (0.79 mL, 11.0 mmol, 1.1 equiv.) was added dropwise and the reaction mixture was stirred at 0 °C for 15 min, then warmed to room temperature and stirred for 15 min. The reaction mixture was filtered through Celite® and washed with Et_2O (100 mL). The solution was concentrated *in vacuo* to afford BiPhONSO **1** as a gold solid (2.09 g, 90%).

IR (thin film, ν_{max}/cm^{-1}) 1601, 1484, 1208, 1156, 1030, 902, 837, 760, 687; **δ_H** (400 MHz, $CDCl_3$) 7.66-7.59 (m, 2H), 7.59-7.54 (m, 2H). 7.48-7.42 (m, 2H), 7.39-7.33 (m, 3H); **δ_C** (100 MHz, $CDCl_3$) 158.2, 140.1, 138.4, 129.0, 128.5, 127.6, 127.1, 114.9; **LRMS** *m/z* (ESI^+) $[M+Na]^+$ 264.040; **HRMS** (ESI^-), *m/z* calculated for $[C_{12}H_8NO_2S]^-$ 230.0281 ($[M-H]^-$), found 230.0276. Data is consistent with the literature.[3]

2.2 Preparation of *tert*-Butyl *N*-sulfinylamine (SI-3)

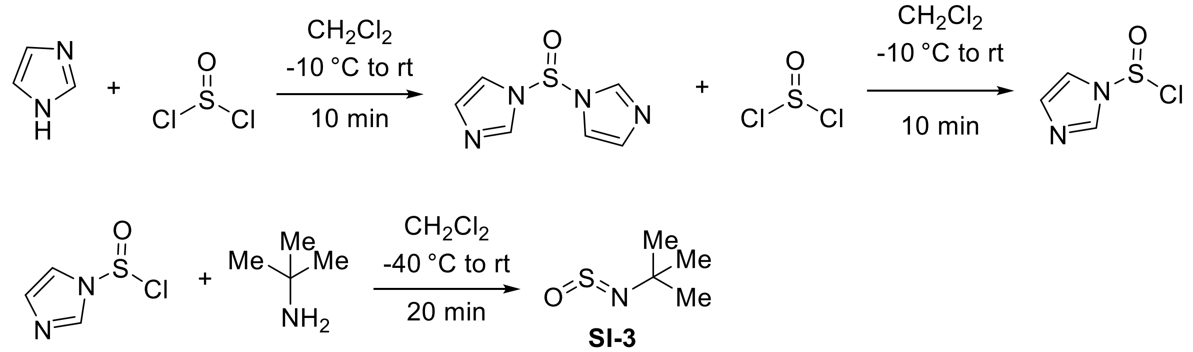

SI-3

Procedure adapted from Y. H. Kim, J. M. Shin*, Tetrahedron Lett*. 1985, **26**, 3821-3824.[4]

Imidazole (1.36 g, 20.0 mmol, 2.0 equiv.) was added to an oven dried round-bottom flask and evacuated and back filled with nitrogen three times. The imidazole was dissolved in CH_2Cl_2 (20 mL) and cooled to -10 °C. Thionyl chloride (0.36 mL, 5.00 mmol, 0.5 equiv.) was added dropwise and stirred at room temperature for 10 min. The solution was filtered through a sinter into another oven dried round-bottom flask under N_2, cooled to 10 °C and thionyl chloride (0.36 mL, 5.00 mmol, 0.5 equiv.) was added dropwise and stirred at room temperature for 10 min. The *N*-(Chlorosulfinyl)imidazole solution formed was added dropwise to a solution of *tert*-butylamine solution (1.05 mL, 10.0 mmol, 1.0 equiv.) in CH_2Cl_2 (10 mL) at -40 ℃ and stirred for 20 min at room temperature. The solution was filtered through Celite® and washed with CH_2Cl_2 (10 mL). Solvents were removed *in vacuo*, followed by distillation of the crude product (97°C, 1013 mbar) to afford *tert*-butyl *N*-sulfinylamine **SI-3** as a brown liquid (0.92 g, 63%).

IR (thin film, ν_{max}/cm^{-1}) 2953, 2927, 2870, 1466, 1199; **δ_H** (500 MHz, $CDCl_3$) 1.52 (s, 9H); **δ_C** (126 MHz, $CDCl_3$) 64.1, 30.7. Data is consistent with the literature.[4]

2.3 Preparation of vinyl *N*-H sulfonimidamides

Optimisation of reaction conditions:

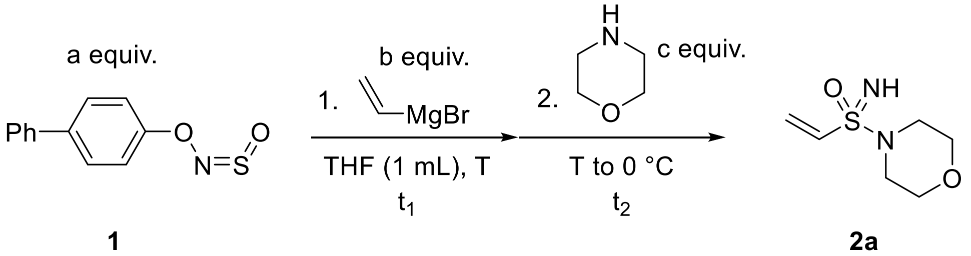

Entry	t_1 (min)	t_2 (min)	T (°C)	a	b	c	Yield %[a]
1	1	15	-78	1.0	1.0	1.5	50
2	3	15	-78	1.0	1.0	1.5	51
3	5	15	-78	1.0	1.0	1.5	47
4	3	3	-78	1.0	1.0	1.5	47
5	3	5	-78	1.0	1.0	1.5	47
6	3	7	-78	1.0	1.0	1.5	52
7	3	9	-78	1.0	1.0	1.5	52
8	3	7	-78	1.0	1.1	1.5	47
9	3	7	-78	1.0	1.0	1.0	43
10	3	7	-78	1.0	1.0	1.2	57*
11	3	7	-78	1.0	1.0	2.0	19
12	3	7	-50	1.0	1.0	1.2	49
13	3	7	-60	1.0	1.0	1.2	49
14	3	7	-78	1.1	1.0	1.32	55
15	3	7	-78	1.5	1.0	1.8	43

Table S1. Full optimization table using BiPhONSO

a: Determined by NMR yield using methyl 3,5-dinitrobenzoate

General procedure A:

The method was adapted from T. Q. Davies, M. J. Tilby, J. Ren, N. A. Parker, D. Skolc, A. Hall, F. Duarte, M. C. Willis, *J. Am. Chem. Soc*. 2020, **142**, 15445-15453.[3]

BiPhONSO **1** (34.7 mg, 0.15 mmol, 1.0 equiv.) was added to an oven-dried reaction tube. The reaction tube was evacuated and back-filled with nitrogen three times. Anhydrous THF (1.0 mL, 0.15 M) was added, and the resulting solution was cooled to -78 °C. The organometallic reagent (1.0 equiv.) was added dropwise over 30 seconds, then the reaction mixture was stirred for 3 min at -78 °C before the amine (1.2 – 1.5 equiv.) was added. The reaction mixture was then transferred to an ice-water bath at 0 °C and stirred for 7 min. The reaction was filtered through a short pad of silica, washed with EtOAc (50 mL) and concentrated *in vacuo*. The crude mixture was purified by flash chromatography to afford the desired sulfonimidamide.

4-(Vinylsulfonimidoyl)morpholine (2a)

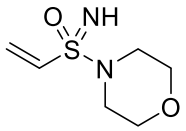

Prepared according to general procedure A, using BiPhONSO **1** (34.7 mg, 0.15 mmol, 1.0 equiv.), vinylmagnesium bromide (167 µL, 0.15 mmol, 0.90 M in THF, 1.0 equiv.) and morpholine (16 µL, 0.18 mmol, 1.2 equiv.). Purification was carried out using flash column chromatography (SiO_2, Petrol/EtOAc/MeOH, 1:3:0 to 0:1:0 to 0:98:2), to afford sulfonimidamide **2a** as a pale-yellow oil (15.0 mg, 57%).

IR (thin film, ν_{max}/cm^{-1}) 3272, 1645, 1245, 1110, 1070, 973, 738; δ_H (400 MHz, $CDCl_3$) 6.42 (dd, 1H, *J* = 10.0 Hz, 16.5 Hz), 6.20 (d, 1H, *J* = 16.5 Hz), 6.02 (d, 1H, *J* = 10.0 Hz), 3.77-3.69 (t, 4H, *J* = 5.0 Hz), 3.11-3.05 (t, 4H, *J* = 5.0 Hz), 2.38 (br. s, 1H); $\boldsymbol{\delta_C}$ (100 MHz, $CDCl_3$) 132.3, 128.6, 66.5, 46.8; **LRMS** *m/z* (ESI^+) $[M+H]^+$ 177.049; **HRMS** (ESI^+, *m/z* calculated for $[C_6H_{13}N_2O_2S]^+$ 177.0692 ($[M+H]^+$), found 177.0690. Data is consistent with literature.[3]

Note: When the reaction was performed on larger scale (0.5 mmol), the yield obtained is significantly lower (35%).

N-(4-Fluorophenyl)ethenesulfonimidamide (2b)

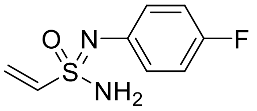

Prepared according to general procedure A, using BiPhONSO **1** (34.7 mg, 0.15 mmol, 1.0 equiv.), vinylmagnesium bromide (167 µL, 0.15 mmol, 0.90 M in THF, 1.0 equiv.) and 4-fluoroaniline (21 µL, 0.23 mmol, 1.5 equiv.). Purification was carried out using flash column chromatography (SiO_2, Petrol/EtOAc, 3:1 to 1:1.5), to afford sulfonimidamide **2b** as a pale-yellow oil (7.50 mg, 25%).

IR (thin film, v_{max}/cm^{-1}) 3219, 1500, 1301, 1213, 1045, 799; **δ_H** (500 MHz, $CDCl_3$) 7.13-7.03 (m, 2H), 6.98-6.88 (m, 2H), 6.67 (dd, 1H, *J* = 16.5 Hz, 9.5 Hz), 6.27 (d, 1H, *J* = 16.5 Hz), 5.91 (d, 1H, *J* = 9.5 Hz), 4.71 (br. s, 2H); **δ_C** (126 MHz, $CDCl_3$) 159.4 (d, $^1J_{CF}$ = 243.5 Hz), 138.1, 137.1 (d, $^4J_{CF}$ = 3.0 Hz), 126.3, 124.7(d, $^3J_{CF}$ = 10.0), 115.9 (d, $^2J_{CF}$ = 22.5 Hz); **^{19}F NMR** (470 MHz, $CDCl_3$) -119.8 (s); **LRMS** *m/z* (ESI^+) $[M+H]^+$ 201.019; **HRMS** (ESI^+, *m/z* calculated for $[C_8H_{10}FN_2OS]^+$ 201.0492 ($[M+H]^+$), found 201.0491.

N'-(4-Ethynylphenyl)ethenesulfonimidamide (2c)

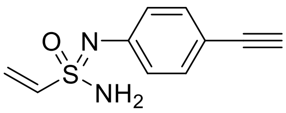

Prepared according to general procedure A, using BiPhONSO **1** (34.7 mg, 0.15 mmol, 1.0 equiv.), vinylmagnesium bromide (167 µL, 0.15 mmol, 0.90 M in THF, 1.0 equiv.) and 4-ethynylaniline (26.4 mg, 0.23 mmol, 1.5 equiv.) Purification was carried out using flash column chromatography (SiO_2, Petrol/EtOAc, 3:1 to 1:1), to afford sulfonimidamide **2c** as a pale brown oil (8.70 mg, 28%).

IR (thin film, v_{max}/cm^{-1}) 3282, 2102, 1601, 1293, 1260, 1045, 967, 841; **δ_H** (400 MHz, $CDCl_3$) 7.42-7.33 (m, 2H), 7.12-7.04 (m, 2H), 6.72 (dd, 1H, *J* = 16.5 Hz, 9.5 Hz), 6.37 (d, 1H, *J* = 16.5 Hz), 5.97 (d, 1H, *J* = 9.5 Hz), 4.77 (br.s, 2H), 3.02 (s, 1H); **δ_C** (100 MHz, $CDCl_3$) 142.7, 138.4, 133.3, 126.4, 122.8, 116.5, 83.8; 76.6; **LRMS** *m/z* (ESI^+) $[M+H]^+$ 207.051; **HRMS** (ESI^+, *m/z* calculated for $[C_8H_{10}FN_2OS]^+$ 207.0587 ($[M+H]^+$), found 207.0588.

_N'_-(4-Methoxyphenyl)ethenesulfonimidamide (2d)

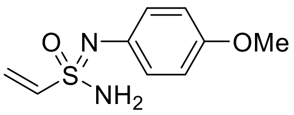

Prepared according to general procedure A, using BiPhONSO **1** (34.7 mg, 0.15 mmol, 1.0 equiv.), vinylmagnesium bromide (167 µL, 0.15 mmol, 0.90 M in THF, 1.0 equiv.) and 4-methoxyaniline (27.7 mg, 0.23 mmol, 1.5 equiv.). Purification was carried out using flash column chromatography (SiO_2, CH_2Cl_2/EtOAc, 5:1 to 3:1 to 1:1), to afford sulfonimidamide **2d** as a pale brown oil (8.01 mg, 25%).

IR (thin film, ν_{max}/cm^{-1}) 3245, 1503, 1235, 1051, 1030, 834; **δ_H** (500 MHz, $CDCl_3$) 7.12-7.04 (m, 2H), 6.83-6.76, (m, 2H), 6.65 (dd, 1H, *J* = 16.5 Hz, 9.5 Hz), 6.21 (d, 1H, *J* = 16.5 Hz), 5.86 (d, 1H, *J* = 9.5 Hz), 5.05 (br. s, 2H), 3.75 (s, 3H); **δ_C** (126 MHz, $CDCl_3$) 156.6, 137.7, 132.9, 126.1, 124.9, 114.4, 55.5; **LRMS** *m/z* (ESI$^+$) [M+H]$^+$ 213.057; **HRMS** (ESI$^+$, *m/z* calculated for $[C_9H_{12}N_2O_2S]^+$ 213.0692 ([M+H]$^+$), found 213.0694.

_N_-(4-Methoxyphenyl)-_N_-methylethenesulfonimidamide (2e)

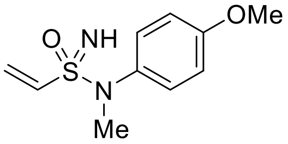

Prepared according to general procedure A, using BiPhONSO **1** (34.7 mg, 0.15 mmol, 1.0 equiv.), vinylmagnesium bromide (167 µL, 0.15 mmol, 0.90 M in THF, 1.0 equiv.) and 4-methoxy-*N*-methylaniline (30.9 mg, 0.23 mmol, 1.5 equiv.). Purification was carried out using flash column chromatography (SiO_2, CH_2Cl_2/EtOAc, 5:1 to 3:1 to 1:1), to afford sulfonimidamide **2e** as a pale-yellow oil (12.6 mg, 37%).

IR (thin film, ν_{max}/cm^{-1}) 3293, 3065, 3013, 2967, 2901, 1607, 1510, 1277, 1247, 1031, 863; **δ_H** (500 MHz, $CDCl_3$) 7.20-7.17 (m, 2H), 6.87-6.84 (m, 2H), 6.55 (dd, 1H, *J* = 16.5 Hz, 10.0 Hz) 6.20 (d, 1H, *J* = 16.5 Hz), 6.00 (d, 1H, *J* = 10.0 Hz), 3.80 (s, 3H), 3.20 (s, 3H), 2.55 (br. s, 1H); **δ_C** (126 MHz, $CDCl_3$) 158.8, 135.7, 133.4, 128.5, 127.5, 114.4, 55.6, 39.7; **HRMS** (ESI$^+$, *m/z* calculated for $[C_{10}H_{15}N_2O_2S]^+$ 227.0849 ([M+H]$^+$), found 227.0850.

4-(Prop-1-en-2-ylsulfonimidoyl)morpholine (2f)

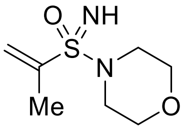

Prepared according to general procedure A, using BiPhONSO **1** (34.7 mg, 0.15 mmol, 1.0 equiv.), isopropenyl magnesium bromide (285 µL, 0.15 mmol, 0.53 M in THF, 1.0 equiv.) and morpholine (16 µL, 0.18 mmol, 1.2 equiv.). Purification was carried out using flash column chromatography (SiO_2, Petrol/EtOAc, 1:3 to 0:1), to afford sulfonimidamide **2f** as a pale-yellow oil (16.2 mg, 57%).

IR (thin film, ν_{max}/cm^{-1}) 3268, 3084, 3018, 2967, 2920, 2855, 1452, 1254, 1101, 933, 642, 543; **δ_H** (400 MHz, $CDCl_3$) 5.98 (s, 1H), 5.67 (s, 1H), 3.71 (app. s, 4H), 3.17 (app. s, 4H), 2.32 (br. s, 1H), 2.10 (s, 3H); **δ_C** (101 MHz, $CDCl_3$)143.1, 123.9, 67.0, 46.8, 19.0; **LRMS** *m/z* (ESI^+) $[M+H]^+$ 191.059; **HRMS** (ESI^+, *m/z* calculated for $[C_7H_{15}N_2O_2S]^+$ 191.0849 ($[M+H]^+$), found 191.0848.

***N'*-(4-Methoxyphenyl)prop-1-ene-2-sulfonimidamide (2g)**

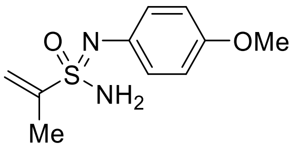

Prepared according to general procedure A, using BiPhONSO **1** (34.7 mg, 0.15 mmol, 1.0 equiv.), isopropenyl magnesium bromide (285 µL, 0.15 mmol, 0.53 M in THF, 1.0 equiv.) and 4-methoxyaniline (27.7 mg, 0.23 mmol, 1.5 equiv.). Purification was carried out using flash column chromatography (SiO_2, Petrol/EtOAc, 3:1 to 1:1 to 1:3), to afford sulfonimidamide **2g** as a pale-yellow oil (19.0 mg, 56%).

IR (thin film, ν_{max}/cm^{-1}) 3256, 3064, 2922, 2835, 1599, 1504, 1234, 1106, 1043, 836, 742; **δ_H** (500 MHz, $CDCl_3$) 7.08-7.05 (m, 2H), 6.81-6.78 (m, 2H), 6.08 (s, 1H), 5.60 (s, 1H), 4.67 (br. s, 2H), 3.76 (s, 3H), 2.14 (s, 3H); **δ_C** (126 MHz, $CDCl_3$) 156.4, 145.4, 133.7, 124.4, 123.6, 114.5, 55.6, 17.5; **HRMS** (ESI^+, *m/z* calculated for $[C_7H_{15}N_2O_2S]^+$ 227.0849 ($[M+H]^+$), found 227.0848.

4-(Prop-1-en-1-ylsulfonimidoyl)morpholine (2h)

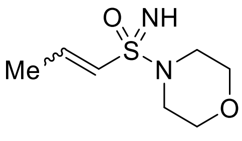

Prepared according to general procedure A, using BiPhONSO **1** (34.7 mg, 0.15 mmol, 1.0 equiv.), 1-propenyl magnesium bromide (300 µL, 0.15 mmol, 0.50 M in THF, 1.0 equiv.) and morpholine (16 µL, 0.18 mmol, 1.2 equiv.). Purification was carried out using flash column chromatography (SiO_2, Petrol/EtOAc, 1:3 to 0:1), to afford sulfonimidamide **2h** as an inseparable 1:1 mixture of cis-trans isomers, as a pale-yellow oil (18.0 mg, 63%).

IR (thin film, ν_{max}/cm^{-1}) 3268, 3056, 3024, 2961, 2918, 2855, 1630, 1454, 1292, 1111, 1069, 1003, 930, 759, 708; **δ_H** (400 MHz, $CDCl_3$) 6.76 (dq, 1H, *J* = 15.0 Hz, 7.0 Hz, trans isomer), 6.43 (dq, 1H, *J* = 11.0 Hz, 7.5 Hz, cis isomer), 6.15 (dq, 1H, *J* = 15.0 Hz, 1.5 Hz, trans isomer), 6.07 (dq, 1H, *J* = 11.0 Hz, 1.5 Hz, cis isomer), 3.75-3.71 (m, 8H, cis and trans isomers), 3.18-3.16 (m, 4H, cis isomer), 3.10-3.08 (m, 4H, trans isomer) 2.38 (br. s, 1H, cis isomer), 2.29 (br. s, 1H, trans isomer), 2.16 (dd, 3H, *J* = 7.5 Hz, 1.5 Hz, cis isomer), 1.95 (dd, 3H, *J* = 7.0 Hz, 1.5 Hz, trans isomer); **δ_C** (101 MHz, $CDCl_3$) 143.7, 142.6, 124.9, 124.5, 66.8, 66.7, 47.04, 46.97, 17.4, 14.4; **HRMS** (ESI$^+$, *m/z* calculated for $[C_7H_{14}N_2O_2SNa]^+$ 213.0668 ($[M+Na]^+$), found 213.0667.

2.4 Synthesis of vinyl sulfonamide

4-(Vinylsulfonyl)morpholine (12)

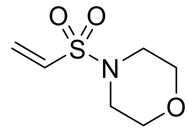

Procedure adapted from Ł. Woźniak, A. A. Rajkiewicz, L. Monsigny, A. Kajetanowicz, K. Grela, *Org. Lett,* 2020, **22**, 4970-4973.[5]

Morpholine (172 µL, 2.00 mmol, 1.0 equiv.) and triethylamine (975 µL, 7.00 mmol, 3.5 equiv.) were added to an oven dried flask that has been evacuated and back-filled with nitrogen three times. Anhydrous CH_2Cl_2 (6.0 mL, 0.33 M) was added and the solution was cooled to 0 °C. 2-Chloroethanesulfonyl chloride (209 µL, 2.00 mmol, 1.0 equiv.) was added and the solution was stirred at 0 °C for 2 hours. The reaction mixture was diluted by $CHCl_3$ (10 mL), washed

with brine (2 × 10 mL), dried over anhydrous Na_2SO_4, filtered and concentrated *in vacuo*. Purification was carried out using flash column chromatography (Petrol/EtOAc, 1:3 to 0:1), to afford the product **12** as a pale-yellow oil (263.2 mg, 74%).

IR (thin film, ν_{max}/cm^{-1}) 3105, 3058, 2985, 2899, 2864, 1456, 1349, 1261, 1161, 1115, 1075, 947, 763.1, 668; **δ_H** (500 MHz, $CDCl_3$) 6.43 (dd, 1H, *J* = 16.5 Hz, 10.0 Hz), 6.27 (d, 1H, *J* = 16.5 Hz), 6.09 (d, *J* = 10.0 Hz), 3.77-3.75 (m, 4H), 3.15-3.13 (m, 4H); **δ_C** (126 MHz, $CDCl_3$) 131.9, 129.7, 66.4, 45.8; **HRMS** (ESI$^+$, *m/z* calculated for $[C_7H_{15}N_2O_2S]^+$ 178.0532 ($[M+H]^+$), found 178.0534. Data is consistent with literature.[4]

2.5 *N*-functionalization of sulfonimidamides

***N*-(Morpholino(oxo)(vinyl)-λ^6-sulfaneylidene)acetamide (3a)**

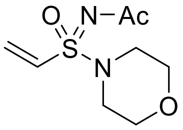

Sulfonimidamide **2a** (26.4 mg, 0.15 mmol, 1.0 equiv.) was added to an oven dried flask and the reaction flask was evacuated and back-filled with nitrogen three times. Anhydrous THF (1.5 mL, 0.10 M) was added and the solution was cooled to 0 °C. Anhydrous triethylamine (31 µL, 0.23 mmol, 1.5 equiv.) was added to the solution, followed by the addition of acetyl chloride (16 µL, 0.23 mmol, 1.5 equiv.). The reaction mixture was warmed to room temperature and stirred for 4 hours. The crude mixture was filtered through Celite®, washed with EtOAc (30 mL) and concentrated *in vacuo.* Purification was carried out using flash column chromatography (SiO_2, Petrol/EtOAc, 1:3 to 0:1), to afford the product **3a** as a colourless oil (18.3 mg, 56%).

IR (thin film, ν_{max}/cm^{-1}) 2970, 2921, 2900, 1643, 1363, 1243, 1112, 1074, 937, 843, 742; **δ_H** (400 MHz, $CDCl_3$) 6.57 (dd, 1H, *J* = 16.5 Hz, 9.5 Hz), 6.40 (d, 1H, *J* = 16.5 Hz), 6.20 (d, 1H, *J* = 9.5 Hz), 3.81-3.70 (m, 4H), 3.25-3.15 (m, 4H), 2.12 (s, 3H); **δ_C** (101 MHz, $CDCl_3$) 178.4, 132.4, 130.4, 66.4, 45.5, 27.2; **LRMS** *m/z* (ESI$^+$) $[M+H]^+$ 219.072; **HRMS** (ESI$^+$, *m/z* calculated for $[C_8H_{15}N_2O_3S]^+$ 219.0798 ($[M+H]^+$), found 219.0798.

N-(Morpholino(oxo)(vinyl)-λ^6-sulfaneylidene)butyramide (3b)

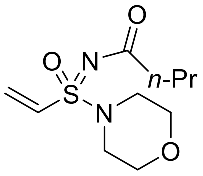

Sulfonimidamide **2a** (38.7 mg, 0.22 mmol, 1.0 equiv.) was added to an oven dried flask and the reaction flask was evacuated and back-filled with nitrogen three times. Anhydrous THF (2.2 mL, 0.10 M) was added and the solution was cooled to 0 °C. Anhydrous triethylamine (46 µL, 0.33 mmol, 1.5 equiv.) was added to the solution, followed by the addition of butyryl chloride (34 µL, 0.33 mmol, 1.5 equiv.) The reaction mixture was warmed to room temperature and stirred for 4 hours. The crude mixture was filtered through Celite®, washed with EtOAc (30 mL) and concentrated *in vacuo.* Purification was carried out using flash column chromatography (SiO_2, Petrol/EtOAc, 1:3 to 0:1), to afford the product **3b** as a colourless oil (40.0 mg, 73%).

IR (thin film, ν_{max}/cm^{-1}) 3102, 3054, 2964, 2928, 2863, 1644, 1455, 1245, 1199, 1073, 937, 847, 740; **δ_H** (400 MHz, $CDCl_3$) 6.57 (dd, 1H, J = 16.5 Hz, 9.5 Hz), 6.40 (d, 1H, J = 16.5 Hz), 6.19 (d, 1H, J = 9.5 Hz), 3.80-3.70 (m, 4H), 3.25-3.16 (m, 4H), 2.34 (t, 2H, J = 7.5 Hz), 1.64 (sext., 2H, J = 7.5 Hz), 0.93 (t, 3H, J = 7.5 Hz); **δ_C** (101 MHz, $CDCl_3$) 181.2, 132.6, 130.2, 66.4, 45.5, 42.0, 19.1, 13.9; **LRMS** *m/z* (ESI^+) $[M+H]^+$ 247.075; **HRMS** (ESI^+, *m/z* calculated for $[C_{10}H_{19}N_2O_3S]^+$ 247.1110 ($[M+H]^+$), found 247.1111.

Ethyl (Morpholino(oxo)(vinyl)- λ^6-sulfaneylidene)carbamate (3c)

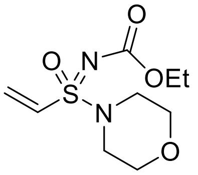

Procedure adapted from F. Izzo, M. Schäfer, P. Lienau, U. Ganzer, R. Stockman, U. Lücking, *Chem. Eur. J.* 2018, **24,** 9295-9304.[6]

Sulfonimidamide **2a** (35.3 mg, 0.20 mmol, 1.0 equiv.) was added to an oven dried flask and the reaction flask was evacuated and back-filled with nitrogen three times. Anhydrous THF (2.0 mL, 0.10 M) was added and the solution was cooled to 0 °C. Pyridine (29 µL, 0.36 mmol,

1.8 equiv.) was added to the solution, followed by the addition of ethyl chloroformate (29 µL, 0.30 mmol, 1.5 equiv.). The reaction mixture was warmed to room temperature and stirred for 16 hours. The crude mixture was filtered through Celite®, washed with EtOAc (30 mL) and concentrated *in vacuo*. Purification was carried out using flash column chromatography (SiO_2, Petrol/EtOAc, 1:1 to 1:3), to afford the product **3c** as a pale-yellow oil (34.7 mg, 70%).

IR (thin film, ν_{max}/cm^{-1}) 3054, 2979, 2903, 2861, 1672, 1245, 1111, 1071, 1021, 938, 787, 695, 654; **δ_H** (400 MHz, $CDCl_3$) 6.49 (dd, 1H, *J* = 16.5 Hz, 9.5 Hz), 6.37 (d, 1H, *J* = 16.5 Hz), 6.17 (d, 1H, *J* = 9.5 Hz); 4.10 (q, 2H, *J* = 7.5 Hz), 3.80-3.69 (m, 4H), 3.27-3.15 (m, 4H), 1.25 (t, 3H, *J* = 7.5 Hz); **δ_C** (101 MHz) 157.3, 132.3, 130.2, 66.3, 62.2, 45.7, 14.4; **LRMS** *m/z* (ESI^+) $[M+H]^+$ 249.054; **HRMS** (ESI^+, *m/z* calculated for $[C_9H_{17}N_2O_4S]^+$ 249.0904 ($[M+H]^+$), found 249.0904.

1-(Morpholino(oxo)(vinyl)- λ⁶-sulfaneylidene)-3-(*p*-tolyl)urea (3d)

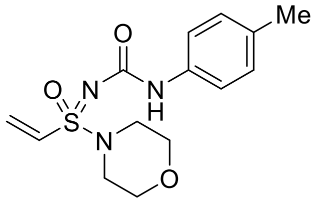

Sulfonimidamide **2a** (26.4 mg, 0.15 mmol, 1.0 equiv.) was added to an oven dried flask and the reaction flask was evacuated and back-filled with nitrogen three times. Anhydrous THF (1.5 mL, 0.10 M) was added and the solution was cooled to 0 °C. Anhydrous triethylamine (21 µL, 0.15 mmol, 1.5 equiv.) was added to the solution, followed by the addition of *p*-tolyl isocyanate (30.0 mg, 0.23 mmol, 1.5 equiv.). The reaction mixture was warmed to room temperature and stirred for 20 hours. Saturated $NH_4Cl_{(aq)}$ (3 mL) was added and EtOAc (3 × 5 mL) was used for extraction. The organic layers were washed with brine (5 mL), dried over anhydrous Na_2SO_4, filtered and concentrated *in vacuo*. Purification was carried out using flash column chromatography (SiO_2, Petrol/EtOAc, 5:1 to 1:1 to 0:1), to afford the product **3d** as a pale-yellow oil (16.9 mg, 36%).

IR (thin film, ν_{max}/cm^{-1}) 3295, 3102, 3046, 2966, 2920, 2858, 1646, 1521, 1275, 1228, 1111, 941, 850, 820; **δ_H** (400 MHz, $CDCl_3$) 7.30 (d, 2H, *J* = 8.0 Hz), 7.07 (d, 2H, *J* = 8.5 Hz), 6.90 (br. s, 1H), 6.66 (dd, 1H, *J* = 16.5Hz, 10.0 Hz), 6.40 (d, 1H, *J* = 16.5 Hz), 6.18 (d, 1H, *J* = 10.0 Hz), 3.79-3.76 (m, 4H), 3.30-3.20 (m, 4H), 2.28 (s, 3H); **δ_C** (101 MHz, $CDCl_3$) 155.7, 136.3, 132.7 (2C),

129.7, 129.5, 119.0, 66.4, 45.8, 20.9; **LRMS** *m/z* (ESI^+) $[M+H]^+$ 310.109; **HRMS** (ESI^+, *m/z* calculated for $[C_{14}H_{20}N_3O_3S]^+$ 310.1220 ($[M+H]^+$), found 310.1217.

N-(Morpholino(oxo)(vinyl)- λ⁶-sulfaneylidene)methanesulfonamide (3e)

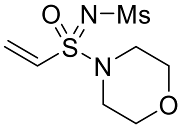

Sulfonimidamide **2a** (26.4 mg, 0.15 mmol, 1.0 equiv.) was added to an oven dried flask and the reaction flask was evacuated and back-filled with nitrogen three times. Anhydrous THF (1.5 mL, 0.10 M) was added and the solution was cooled to 0 °C. Anhydrous triethylamine (31 µL, 0.23 mmol, 1.5 equiv.) was added to the solution, followed by the addition of methanesulfonyl chloride (17 µL, 0.23 mmol, 1.5 equiv.). The reaction mixture was warmed to room temperature and stirred for 3 hours. The crude mixture was filtered through Celite®, washed with EtOAc (30 mL) and concentrated *in vacuo.* Purification was carried out using flash column chromatography (SiO_2, Petrol/EtOAc, 1:1 to 1:3 to 0:1), to afford the product **3e** as a pale-yellow oil (26.7 mg, 70%).

IR (thin film, ν_{max}/cm^{-1}) 3055, 3020, 2922, 2859, 1304, 1259, 1145, 1101, 1071, 937, 801, 760; $\boldsymbol{\delta_H}$ (400 MHz, $CDCl_3$) 6.49 (dd, 1H, *J* = 16.5 Hz, 9.0 Hz), 6.41 (d, 1H, *J* = 16.5 Hz), 6.24 (d, 1H, *J* = 9.0 Hz), 3.83-3.73 (m, 4H), 3.36-3.17 (m, 4H), 3.14-3.09 (s, 3H); $\boldsymbol{\delta_C}$ (101 MHz, $CDCl_3$) 132.1, 130.9, 66.2, 46.1, 45.0; **LRMS** *m/z* (ESI^+) $[M+H]^+$ 255.067; **HRMS** (ESI^+, *m/z* calculated for $[C_8H_{15}N_2O_3S]^+$ 255.0472 ($[M+H]^+$), found 255.0465.

N-(Morpholino(oxo)(vinyl)-λ⁶-sulfaneylidene)cyanamide (3f)

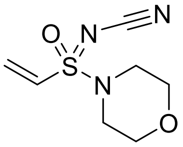

Sulfonimidamide **2a** (35.2 mg, 0.20 mmol, 1.0 equiv.) was added to an oven dried flask and the reaction flask was evacuated and back-filled with nitrogen three times. Anhydrous CH_2Cl_2 (2.0 mL, 0.10 M) was added and the solution was cooled to 0 °C. Anhydrous triethylamine (42

µL, 0.30 mmol, 1.5 equiv.) was added to the solution, followed by the addition of cyanogen bromide (80 µL, 0.24 mmol, 3.0 M in CH_2Cl_2, 1.2 equiv.). The reaction mixture was warmed to room temperature and stirred for 16 hours. The crude mixture was quenched with $NaHCO_3$ $_{(aq)}$ (10 mL) and extracted with dichloromethane (10 mL × 3). The combined organic layers were washed with brine (10 mL) and concentrated *in vacuo*. Purification was carried out using flash column chromatography (SiO_2, Petrol/EtOAc, 1:1 to 0:1), to afford the product **3f** as a colourless oil (4.4 mg, 11%).

IR (thin film, ν_{max}/cm^{-1}) 3057, 2984, 2921, 2860, 2205, 1455, 1259, 1201, 1111, 1073, 942, 844, 771, 647, 621; **δ_H** (600 MHz, $CDCl_3$) 6.49-6.35 (m, 2H), 6.30 (dd, 1H, *J* = 7.5 Hz, 1.5 Hz), 3.82-3.71 (m, 4H), 3.24-3.14 (m, 4H); **δ_C** (151 MHz, $CDCl_3$) 133.4, 129.9, 110.2, 66.1, 46.2; **LRMS** *m/z* (ESI$^+$) $[M+H]^+$ 202.061; **HRMS** (ESI$^+$, *m/z* calculated for $[C_9H_{17}N_2O_4S]^+$ 202.0645 ($[M+H]^+$), found 202.0646.

4-(*N*-Butylvinylsulfonimidoyl)morpholine (3g)

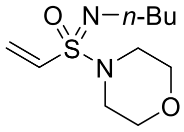

Procedure adapted from F. Izzo, M. Schäfer, P. Lienau, U. Ganzer, R. Stockman, U. Lücking, *Chem. Eur. J.* 2018, **24**, 9295-9304.[6]

Sulfonimidamide **2a** (45.8 mg, 0.26 mmol, 1.0 equiv.) was added to an oven dried flask and the reaction flask was evacuated and back-filled with nitrogen three times. Anhydrous DMSO (1.3 mL, 0.2 M) was added and bromobutane (42 µL, 0.39 mmol, 1.5 equiv.) was added, followed by the addition of KHMDS (104 mg, 0.52 mmol, 2.0 equiv.). The solution was stirred at room temperature for 2 hours. H_2O (10 mL) and EtOAc (3 × 20 mL) were used for extraction and the organic layer was washed with brine (10 mL), dried over anhydrous Na_2SO_4, filtered and concentrated *in vacuo*. Purification was carried out using flash column chromatography (SiO_2, Petrol/EtOAc, 1:1 to 0:1), to afford the product **3g** as a pale-yellow oil (28.6 mg, 47%).

IR (thin film, ν_{max}/cm^{-1}) 3054, 2958, 2927, 2857, 1453, 1278, 1255, 1152, 1113, 1068, 931, 758, 653; **δ_H** (400 MHz, $CDCl_3$) 6.40 (dd, 1H, *J* = 17.0 Hz, 10.0 Hz), 6.16 (d, 1H, *J* = 17.0 Hz), 5.97 (d, 1H, *J* = 10.0 Hz), 3.78-3.65 (m, 4H), 3.24-2.89 (m, 6H), 1.58-1.46 (m, 2H), 1.42-1.29 (m, 2H),

0.89 (t, 3H, J = 7.5 Hz); **δ_C** (101 MHz, $CDCl_3$) 132.5, 127.6, 66.6, 46.8, 41.5, 34.8, 20.5, 14.0; **LRMS** *m/z* (ESI^+) $[M+H]^+$ 233.082; **HRMS** (ESI^+), *m/z* calculated for $[C_{10}H_{21}N_2O_2S]^+$ 233.1818 ($[M+H]^+$), found 233.1818.

4-(*N*-Ethylvinylsulfonimidoyl)morpholine (3h)

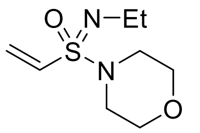

Procedure adapted from F. Izzo, M. Schäfer, P. Lienau, U. Ganzer, R. Stockman, U. Lücking. *Chem. Eur. J*, 2018, **24**, 9295-9304.[6]

Sulfonimidamide **2a** (45.8 mg, 0.26 mmol, 1.0 equiv.) was added to an oven dried flask and the reaction flask was evacuated and back-filled with nitrogen three times. Anhydrous DMSO (1.3 mL, 0.20 M) and bromoethane (30 µL, 0.39 mmol, 1.5 equiv.) was added, followed by the addition of KHMDS (103.7 mg, 0.52 mmol, 2.0 equiv.). The solution was stirred at room temperature for 2 hours. H_2O (10 mL) and EtOAc (3 × 20 mL) were used for extraction and the organic layer was washed with brine (10 mL), dried over anhydrous Na_2SO_4, filtered and concentrated *in vacuo*. Purification was carried out using flash column chromatography (SiO_2, Petrol/EtOAc, 1:2 to 0:1), to afford the product **3h** as a pale-yellow oil (26.6 mg, 50%).

IR (thin film, ν_{max}/cm^{-1}) 3052, 2967, 2917, 2895, 2857, 1453, 1293, 1245, 1152, 1111, 1084, 927, 824, 756, 650; **δ_H** (400 MHz, $CDCl_3$) 6.41 (dd, 1H, J = 17.0 Hz, 10.0 Hz), 6.17 (d, 1H, J = 17.0 Hz), 5.98 (d, 1H, J = 10.0 Hz), 3.27-3.16 (m, 1*H*), 3.12-2.96 (m, 5H), 1.19 (3H, t, J = 7.0 Hz); **δ_C** (101 MHz, $CDCl_3$) 132.4, 127.7, 66.6, 46.9, 36.6, 18.2; **LRMS** *m/z* (ESI^+) $[M+H]^+$ 205.076; **HRMS** (ESI^+, *m/z* calculated for $[C_8H_{17}N_2O_2S]^+$ 205.1005 ($[M+H]^+$), found 205.1005.

N-(*tert*-Butyl)ethenesulfinamide (SI-4)

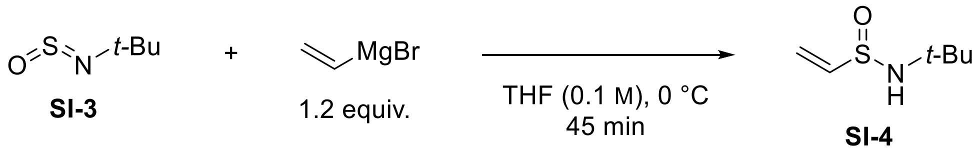

Sulfinylamine **SI-3** (238 mg, 2.00 mmol, 1.0 equiv.) was added to an oven dried flask and dissolved in anhydrous THF (2.0 mL, 0.10 M). The solution was quickly degassed and refilled with nitrogen for three times. The solution was cooled to 0 °C and vinylmagnesium bromide (2.45 mL, 0.98 M, 2.40 mmol, 1.2 equiv.) was added dropwise. The solution was stirred at 0 °C for 45 min, quenched with silica, filtered through a short pad of silica and washed with EtOAc (30 mL). The crude mixture was concentrated *in vacuo* and purified using flash column chromatography (Petrol/EtOAc 1:1 to 1:3), to afford the product **SI-4** as a pale-yellow solid (86.2 mg, 29%).

mp 48-50 °C (EtOAc)**; IR** (thin film, ν_{max}/cm^{-1}) 3146, 3053, 3012, 2969, 2921, 1735, 1605, 1367, 1235, 1054, 963, 806, 668; **δ_H** (500 MHz, $CDCl_3$) 6.59 (dd, 1H, *J* = 16.5, 9.5 Hz), 6.11 (d, 1H, *J* = 16.5 Hz), 5.87 (d, 1H , *J* = 9.5 Hz), 3.64 (br. s., 1H), 1.33 (s, 9H); **δ_C** (126 MHz, $CDCl_3$) 143.0, 122.3, 54.3, 31.1; **HRMS** (ESI$^+$, *m/z* calculated for $[C_6H_{14}NOS]^+$ 148.0791 ($[M+H]^+$), found 148.0790.

4-(*N*-(*tert*-Butyl)vinylsulfonimidoyl)morpholine (3i)

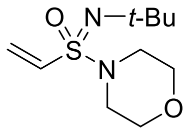

Sulfinamide **SI-4** (86.2 mg, 0.59 mmol, 1.0 equiv.) was added to an oven dried flask and the reaction flask was evacuated and back-filled with nitrogen three times. The sulfinamide **SI-4** was dissolved in anhydrous CH_2Cl_2 (5.9 mL, 1.0 M) and cooled to 0 °C. *tert*-Butyl hypochlorite (66 µL, 0.59 mmol, 1.0 equiv.) was added and the solution was stirred for 30 min. Anhydrous triethylamine (244 µL, 1.76 mmol, 3.0 equiv.) and morpholine (61 µL, 0.70 mmol, 1.2 equiv.) were added and the solution was warmed to room temperature and stirred for 4 hours. The crude mixture was filtered through Celite®, washed with EtOAc (30 mL), concentrated *in*

vacuo and purified using flash column chromatography (SiO_2, Petrol/EtOAc 1:1 to 1:3), to afford the product **3i** as a yellow oil (23.6 mg, 17%).

IR (thin film, ν_{max}/cm^{-1}) 3077, 3045, 2974, 2898, 2861, 1455, 1300, 1257, 1212, 1142, 930, 757, 651; **δ_H** (500 MHz, $CDCl_3$) 6.37 (dd, 1H, *J* = 16.5, 10.0 Hz), 6.05 (d, 1H, *J* = 16.5 Hz), 5.87 (d, 1H, 10.0 Hz), 3.76-3.69 (m, 4H), 3.08-3.06 (m, 4H), 1.35 (s, 9H); **δ_C** (126 MHz, $CDCl_3$) 133.3, 125.4, 66.8, 55.2, 47.5, 33.2; **HRMS** (ESI^+, *m/z* calculated for $[C_{10}H_{21}N_2O_2S]^+$ 233.1318 ($[M+H]^+$), found 231.1321.

4-(*N*-(*p*-Tolyl)vinylsulfonimidoyl)morpholine (3j)

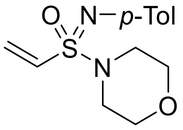

Procedure adapted from J. C. Vantourout, L. Li, E. B. Moll, S. Chabbra, K. Arrington, B. E. Bode, A. I. Llobet, J. A. Kowalski, M. G. Nilson, K. M. P. Wheelhouse, J. L. Woodard, S. Xie, D. Leitch, A. J. B. Watson. *ACS Catal*, 2018, **8**, 9560–9566[7]

Sulfonimidamide **2a** (44.1 mg, 0.25 mmol, 1.0 equiv.), *p*-tolylboronic acid (78.2 mg, 0.58 mmol, 2.3 equiv.), $Cu(MeCN)_4BF_4$ (11.8 mg, 0.038 mmol, 15 mol%) were added to an oven dried flask and the reaction flask was evacuated and back-filled with nitrogen three times. Anhydrous DMF (0.83 mL, 0.3 M) was added and the flask was changed to an oxygen environment. The reaction mixture was stirred at room temperature for 24 hours. H_2O (10 mL) was added to the vial for quenching the reaction and the flask was opened to air. The crude mixture was extracted with EtOAc (3 × 10 mL), the organic layer was washed with brine (10 mL), dried over anhydrous Na_2SO_4, filtered and concentrated *in vacuo*. Purification was carried out using flash chromatography (Petrol/EtOAc, 5:1, 2:1 to 1:1), to afford the product **3j** as a pale-yellow oil (27.9 mg, 42%).

IR (thin film, ν_{max}/cm^{-1}) 3052, 3022, 2965, 2917, 2893, 2856, 1609, 1506, 1452, 1379, 1310, 1257, 1225, 1112, 1074, 930, 822, 761, 654; **δ_H** (400 MHz, $CDCl_3$) 7.08-6.99 (m, 4 H),6.53 (dd. 1H, *J* = 16.5 Hz, 10.0 Hz), 6.31 (d, 1H, *J* = 16.5 Hz), 6.08 (d, 1H , *J* = 10.0 Hz), 3.73-3.58 (m, 4H), 3.22-3.07 (m, 4H), 2.30-2.23 (s, 3H); **δ_C** (101 MHz, $CDCl_3$) 140.3, 132.8, 131.7, 129.7, 128.3,

123.5, 66.5, 46.6, 20.9; **LRMS** *m/z* (ESI$^+$) [M+H]$^+$ 267.077; **HRMS** (ESI$^+$, *m/z* calculated for [$C_{13}H_{19}N_2O_2S$]$^+$ 267.1162 ([M+H]$^+$), found 267.1162.

***N*-(Morpholino(oxo)(prop-1-en-2-yl)-λ^6-sulfaneylidene)acetamide (4a)**

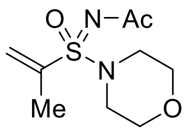

Sulfonimidamide **2f** (19.0 mg, 0.10 mmol, 1.0 equiv.) was added to an oven dried flask and the reaction flask was evacuated and back-filled with nitrogen three times. Anhydrous THF (1.0 mL, 0.10 M) was added and the solution was cooled to 0 °C. Triethylamine (21 µL, 0.15 mmol, 1.5 equiv.) was added to solution, followed by the addition of acetyl chloride (11 µL, 0.15 mmol, 1.5 equiv.). The reaction mixture was warmed to room temperature and stirred for 2 hours. The crude mixture was filtered through Celite@, washed with EtOAc (30 mL) and concentrated *in vacuo.* Purification was carried out using flash column chromatography (SiO_2, Petrol/EtOAc, 1:3 to 0:1), to afford the product **4a** as a colourless oil (14.0 mg, 60%).

IR (thin film, ν_{max}/cm^{-1}) 3106, 2974, 2916, 2899, 2862, 1648, 1453, 1364, 1256, 1113, 1073, 1043, 940, 835, 737; **δ_H** (400 MHz, $CDCl_3$) 6.18 (s, 1H), 5.85 (s, 1H), 3.76-3.74 (m, 4H), 3.27-3.24 (m, 4H), 2.12 (s, 3H), 2.07 (s, 3H); **δ_C** (101 MHz, $CDCl_3$) 178.7, 142.0, 126.4, 66.6, 45.5, 27.1, 17.0; **HRMS** (ESI$^+$, *m/z* calculated for [$C_9H_{17}N_2O_3S$]$^+$ 233.0954 ([M+H]$^+$), found 233.0945.

***N*-(Morpholino(oxo)(prop-1-en-2-yl)-λ^6-sulfaneylidene)methanesulfonamide (4b)**

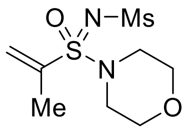

Sulfonimidamide **2f** (30.4 mg, 0.16 mmol, 1.0 equiv.) was added to an oven dried flask and the reaction flask was evacuated and back-filled with nitrogen three times. Anhydrous THF (1.6 mL, 0.10 M) was added and the solution was cooled to 0 °C. Anhydrous triethylamine (34 µL, 0.24 mmol, 1.5 equiv.) was added to solution, followed by the addition of methanesulfonyl chloride (19 µL, 0.24 mmol, 1.5 equiv.). The reaction mixture was warmed to room temperature and stirred for 2 hours. The crude mixture was filtered through Celite@, washed

with EtOAc (30 mL) and concentrated *in vacuo.* Purification was carried out using flash column chromatography (SiO_2, Petrol/EtOAc, 1:1 to 1:3 to 0:1), to afford the product **4b** as a pale-yellow oil (39.0 mg, 91%).

IR (thin film, ν_{max}/cm^{-1}) 3106, 3018, 2967, 2926, 2862, 1452, 1306, 1258, 1207, 1146, 1109, 1084, 943, 802, 747; **δ_H** (400 MHz, $CDCl_3$) 6.20 (s, 1H $_b$), 5.87 (s, 1H), 3.81-3.72 (m, 4H), 3.38-3.24 (m, 4H), 3.12 (s, 3H), 2.12 (s, 3H); **δ_C** (100 MHz, $CDCl_3$) 141.9, 126.9, 66.4, 46.1, 44.9, 16.9; **HRMS** (ESI$^+$, *m/z* calculated for $[C_8H_{17}N_2O_4S_2]^+$ 269.0624 ([M+H]$^+$), found 269.0624.

4-(*N*-Butylprop-1-en-2-ylsulfonimidoyl)morpholine (4c)

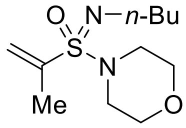

Sulfonimidamide **2f** (38.0 mg, 0.20 mmol, 1.0 equiv.) was added to an oven dried flask and the reaction flask was evacuated and back-filled with nitrogen three times. Anhydrous DMSO (2 mL, 0.10 M) and bromobutane (32 µL, 0.30 mmol, 1.5 equiv.) were added, followed by the addition of KHMDS (79.8 mg, 0.40 mmol, 2.0 equiv.). The reaction mixture was stirred for 2 hours at room temperature. H_2O (15 mL) and EtOAc (3 × 15 mL) were used for extraction and the organic layer was washed with brine (15 mL), dried over anhydrous Na_2SO_4, filtered and concentrated *in vacuo*. Purification was carried out using flash column chromatography (SiO_2, Petrol/EtOAc, 3:1 to 1:1 to 1:3), to afforded the product **4c** as a pale-yellow oil (37.5 mg, 76%).

IR (thin film, ν_{max}/cm^{-1}) 2958, 2928, 2856, 1635, 1452, 1361, 1294, 1275, 1144, 1113, 745; **δ_H** (400 MHz, $CDCl_3$) 5.91 (q, 1H, *J* = 1.0 Hz), 5.61 (q, 1H, *J* = 1.5 Hz), 3.76-3.67 (m, 4H), 3.19-3.06 (m, 5H), 2.98-2.91 (m, 1H), 2.08 (dd, 3H, *J* = 1.5 Hz, 1.0 Hz), 1.57-1.49 (m, 2H), 1.42-1.33 (m, 2H), 0.91 (t, 3H, 7.5 Hz); **δ_C** (101 MHz, $CDCl_3$) 143.0, 122.9, 67.0, 47.0, 41.9, 35.0, 20.6, 19.2, 14.0; **HRMS** (ESI$^+$, *m/z* calculated for $[C_{11}H_{23}N_2O_2S]^+$ 247.1475 ([M+H]$^+$), found 247.1474.

(*E*)-4-(Prop-1-en-1-ylsulfonimidoyl)morpholine (*E*-2h)

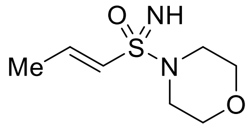

Sulfonimidamide **2h** (16.4 mg, 0.197 mmol, 1.0 equiv.) was added to an oven dried flask and the reaction flask was evacuated and back-filled with nitrogen three times. The sulfonimidamide was dissolved in anhydrous DMSO (2.0 mL, 0.99 M). KHMDS (47.1 mg, 0.394 mmol, 2.0 equiv.) was added and the solution was stirred at room temperature for 2 hours. H_2O (10 mL) and EtOAc (3 × 20 mL) were used for extraction and the organic layer was washed with brine (10 mL), dried over anhydrous Na_2SO_4, filtered and concentrated *in vacuo*. Purification was carried out using flash column chromatography (SiO_2, Petrol/EtOAc, 1:3 to 0:1), to afford the product (*E*)-**2h** as a pale-yellow oil (23.5 mg, 60%).

IR (thin film, ν_{max}/cm^{-1}) 3285, 3056, 3024, 2985, 2975, 2862, 1642, 1455, 1294, 1261, 1115, 1071, 1011, 933, 805, 710; **δ_H** (400 MHz, $CDCl_3$) 6.77 (dq, 1H, *J* = 15.0 Hz, 7.0 Hz), 6.16 (dq, 1H, *J* = 15.0 Hz, 1.5 Hz), 3.75-3.70 (m, 4H), 3.11-3.09 (m, 4H), 2.28 (br. s, 1H), 1.96 (dd, 3H, *J* = 7.0 Hz, 1.5 Hz) ; **δ_C** (101 MHz, $CDCl_3$) 143.5, 125.1, 66.7, 47.1, 17.4; **HRMS** (ESI^+, *m/z* calculated for $[C_7H_{15}N_2O_2S]^+$ 191.0849 ($[M+H]^+$), found 191.0855.

(*E*)-*N*-(Morpholino(oxo)(prop-1-en-1-yl)-λ^6-sulfaneylidene)acetamide (4d)

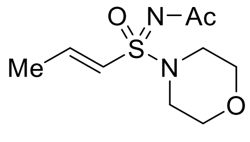

Sulfonimidamide (*E*)-**2h** (11.4 mg, 0.06 mmol, 1.0 equiv.) was added to an oven dried flask and the reaction flask was evacuated and back-filled with nitrogen three times. Anhydrous THF (0.6 mL, 0.10 M) was added and the solution was cooled to 0 °C. Anhydrous triethylamine (12 µL, 0.09 mmol, 1.5 equiv.) was added to solution, followed by the addition of methanesulfonyl chloride (6 µL, 0.09 mmol, 1.5 equiv.). The reaction mixture was warmed to room temperature and stirred for 2 hours. The crude mixture was filtered through Celite$^@$, washed with EtOAc (30 mL) and concentrated *in vacuo.* Purification was carried out using flash column chromatography (SiO_2, Petrol/EtOAc, 1:1 to 1:3 to 0:1), to afford the product **4d** as a pale-yellow oil (9.7 mg, 72%).

IR (thin film, ν_{max}/cm^{-1}) 3048, 3030, 2987, 2976, 2900, 2863, 1644, 1455, 1440, 1364, 1256, 1113, 1074, 936, 845, 732; **δ_H** (500 MHz, $CDCl_3$) 6.94 (dq, 1H, *J* = 15.0 Hz, 7.0 Hz), 6.28 (dq, 1H, *J* = 15.0 Hz, 1.5 Hz), 3.77-3.75 (m, 4H), 3.19-3.17 (m, 4H), 2.11 (s, 3H), 2.00 (dd, 3H, *J* = 7.0 Hz, 1.5 Hz); **δ_C** (126 MHz, $CDCl_3$) 178.4, 145.7, 130.0, 66.4, 45.5, 27.2, 17.7; **HRMS** (ESI$^+$, *m/z* calculated for $[C_9H_{17}N_2O_3S]^+$ 233.0954 ($[M+H]^+$), found 233.0952.

(*E*)-*N*-(Morpholino(oxo)(prop-1-en-1-yl)-λ^6-sulfaneylidene)methanesulfonamide (4e)

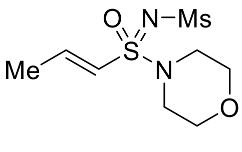

Sulfonimidamide (*E*)-**2h** (11.4 mg, 0.06 mmol, 1.0 equiv.) was added to an oven dried flask and the reaction flask was evacuated and back-filled with nitrogen three times. Anhydrous THF (0.6 mL, 0.10 M) was added and the solution was cooled to 0 °C. Anhydrous triethylamine (12 µL, 0.09 mmol, 1.5 equiv.) was added to solution, followed by the addition of methanesulfonyl chloride (7 µL, 0.09 mmol, 1.5 equiv.). The reaction mixture was warmed to room temperature and stirred for 2 hours. The crude mixture was filtered through Celite@, washed with EtOAc (30 mL) and concentrated *in vacuo.* Purification was carried out using flash column chromatography (SiO_2, Petrol/EtOAc, 1:1 to 1:3 to 0:1), to afford the product **4e** as a pale-yellow oil (13.8 mg, 86%).

IR (thin film, ν_{max}/cm^{-1}) 3058, 3030, 2978, 2960, 2918, 2864, 1629, 1456, 1311, 1261, 1149, 1113, 940, 801, 732, 635; **δ_H** (500 MHz, $CDCl_3$) 6.96 (dq, 1H, *J* = 15.0 Hz, 7.0 Hz), 6.18 (dq, 1H, *J* = 15.0 Hz, 1.5 Hz), 3.82-3.75 (m, 4H), 3.32-3.16 (m, 4H), 3.12 (s, 3H), 2.02 (dd, 3H, *J* = 7.0 Hz, 1.5 Hz); **δ_C** (126 MHz, $CDCl_3$) 146.5, 124.7, 66.2, 46.1, 44.9, 17.7; **HRMS** (ESI$^+$, *m/z* calculated for $[C_8H_{16}N_2O_4S_2Na]^+$ 291.0444 ($[M+H]^+$), found 291.0442.

(*E*)-4-(*N*-Butylprop-1-en-1-ylsulfonimidoyl)morpholine (4f)

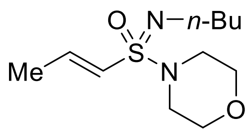

Sulfonimidamide (*E*)-**2h** (8.2 mg, 0.043 mmol, 1.0 equiv.) was added to an oven dried flask and the reaction flask was evacuated and back-filled with nitrogen three times. Anhydrous DMSO (0.5 mL, 0.10 M) and bromobutane (7 µL, 0.065 mmol, 1.5 equiv.) were added, followed by the addition of KHMDS (17.2mg, 0.086 mmol, 2.0 equiv.). The reaction mixture was stirred for 2 hours at room temperature. H_2O (15 mL) and EtOAc (3 × 15 mL) were used for extraction and the organic layer was washed with brine (15 mL), dried over anhydrous Na_2SO_4, filtered and concentrated *in vacuo*. Purification was carried out using flash column chromatography (SiO_2, Petrol/EtOAc, 3:1 to 1:1 to 1:3), to afford the product **4f** as a pale-yellow oil (8.7 mg, 82%).

IR (thin film, $ν_{max}/cm^{-1}$) 3054, 3021, 2959, 2917, 2896, 2858, 1645, 1454, 1273, 1257, 1122, 1152, 1115, 932, 818, 702; **$δ_H$** (400 MHz, $CDCl_3$) 6.71 (dq, 1H, *J* = 15.0 Hz, 7.0 Hz), 6.14 (dq, 1H, *J* = 1.5 Hz), 3.78-3.69 (m, 4H), 3.20-3.13 (m, 1H), 3.10-2.97 (m, 4H), 2.96-2.90 (m, 1H), 1.93 (dd, 3H, *J* = 7.0 Hz, 1.5 Hz), 1.54-1.49 (m, 2H), 1.42-1.33 (m, 2H), 0.91 (t, 3H, 7.5 Hz); **$δ_C$** (101 MHz, $CDCl_3$) 142.3, 125.3, 66.7, 46.9, 41.6, 39.4, 20.6, 17.3, 14.0; **HRMS** (ESI^+, *m/z* calculated for $[C_{11}H_{23}N_2O_2S]^+$ 247.1475 ($[M+H]^+$), found 247.1468.

***N*-(((4-Methoxyphenyl)(methyl)amino)(oxo)(vinyl)-λ^6-sulfaneylidene)methanesulfonamide (5a)**

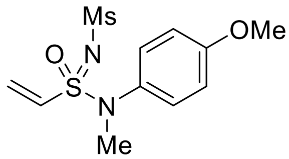

Sulfonimidamide **2e** (19.3 mg, 0.085 mmol, 1.0 equiv.) was added to an oven dried flask and the reaction flask was evacuated and back-filled with nitrogen three times. Anhydrous THF (0.85 mL, 0.10 M) was added and the solution was cooled to 0 °C. Anhydrous triethylamine (18 µL, 0.1275 mmol, 1.5 equiv.) was added to the solution, followed by the addition of methanesulfonyl chloride (10 µL, 0.1275 mmol, 1.5 equiv.). The reaction mixture was warmed

to room temperature and stirred for 2 hours. The crude mixture was filtered through Celite@, washed with EtOAc (30 mL) and concentrated *in vacuo*. Purification was carried out using flash column chromatography (SiO_2, CH_2Cl_2/EtOAc, 7:1 to 5:1 to 3:1), to afford the product **5a** as a pale-yellow oil (13.2 mg, 51%).

IR (thin film, ν_{max}/cm^{-1}) 3130, 3105, 3067, 3058, 2944, 2906,1607, 1511, 1314, 1252,883, 807; **δ_H** (500 MHz, $CDCl_3$) 7.33-7.30 (m, 2H), 6.91-6.87 (m, 2H), 6.60 (dd, 1H, *J* = 16.5 Hz, 10.0 Hz), 6.33 (d, 1H, *J* = 16.5 Hz), 6.14 (d, 1H, *J* = 10.0 Hz), 3.81 (s, 3H), 3.66 (s, 3H), 3.13 (s, 3H); **δ_C** (126 MHz, $CDCl_3$) 159.8, 133.3, 132.5, 129.8, 129.6, 114.8, 55.6, 45.0, 40.0; **HRMS** (ESI$^+$, *m/z* calculated for $[C_{11}H_{16}N_2O_4S_2Na]^+$ 327.0444 ($[M+Na]^+$), found 327.0443.

N-(((4-Methoxyphenyl)(methyl)amino)(oxo)(vinyl)-λ^6-sulfaneylidene)acetamide (5b)

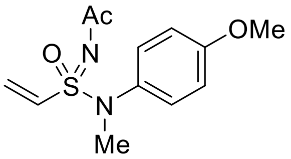

Sulfonimidamide **2e** (19.3 mg, 0.085 mmol, 1.0 equiv.) was added to an oven dried flask and the reaction flask was evacuated and back-filled with nitrogen three times. Anhydrous THF (0.85 mL, 0.10 M) was added and the solution was cooled to 0 °C. Anhydrous triethylamine (18 µL, 0.1275 mmol, 1.5 equiv.) was added, followed by the addition of acetyl chloride (9 µL, 0.1275 mmol, 1.5 equiv.), The reaction mixture was warmed to room temperature and stirred for 5 hours. The crude mixture was filtered through Celite@, washed with EtOAc (30 mL) and concentrated *in vacuo*. Purification was carried out using flash column chromatography (SiO_2, CH_2Cl_2/EtOAc, 5:1 to 3:1 to 1:1), to afford the product **5b** as a pale-yellow oil (8.9 mg, 39%).

IR (thin film, ν_{max}/cm^{-1}) 3081, 3072. 3059, 3022, 2997, 2952, 2903, 2894, 1653, 1510, 1364, 1249, 1058, 876, 795, 669; **δ_H** (500 MHz, $CDCl_3$) 7.29-7.26 (m, 2H), 6.88-6.85 (m, 2H), 6.76 (dd, 1H, *J* = 16.5 Hz, 10.0 Hz), 6.33 (d, 1H, *J* = 16.5 Hz), 6.09 (d, 1H, *J* = 10.0 Hz), 3.80 (s, 3H), 3.23 (s, 3H), 2.03 (s, 3H); **δ_C** (126 MHz, $CDCl_3$) 178.3, 159.5, 133.5, 133.3, 129.6, 129.4, 114.6, 55.6, 39.0, 27.2; **HRMS** (ESI$^+$, *m/z* calculated for $[C_{12}H_{17}N_2O_3S]^+$ 269.0954 ($[M+H]^+$), found 269.0943.

***N*'-Ethyl-*N*-(4-methoxyphenyl)-*N*-methylethenesulfonimidamide (5c)**

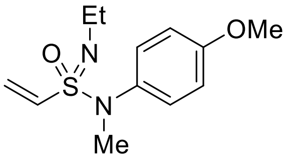

Sulfonimidamide **2e** (22.6 mg, 0.10 mmol, 1.0 equiv.) was added to an oven dried flask and the reaction flask was evacuated and back-filled with nitrogen three times. Anhydrous DMSO (1.0 mL, 0.10 M) and bromoethane (11 µL, 0.15 mmol, 1.5 equiv.) were added, followed by the addition of KHMDS (40.0 mg, 0.2 mmol, 2.0 equiv.). The reaction mixture was stirred for 2 hours at room temperature. H_2O (15 mL) and EtOAc (3 × 15 mL) were used for extraction and the organic layer was washed with brine (15 mL), dried over anhydrous Na_2SO_4, filtered and concentrated *in vacuo*. Purification was carried out using flash column chromatography (SiO_2, CH_2Cl_2/EtOAc, 7:1 to 5:1 to 3:1), afforded the product **5c** as a pale-yellow oil (11 mg, 43%).

IR (thin film, ν_{max}/cm^{-1}) 3056, 6013, 2965, 2933, 2920, 2860, 1510, 1300, 1248, 1108, 1008, 862, 745, 657; **δ_H** (400 MHz, $CDCl_3$) 7.19-7.15 (m, 2H), 6.87-6.83 (m, 2H), 6.56 (dd, 1H, *J* = 16.5 Hz, 10.0 Hz), 6.11 (d, 1H, *J* = 16.5 Hz), 5.92 (d, 1H, *J* = 10.0 Hz), 3.79 (s, 3H), 3.36 (dq, 1H, *J* = 12.5, 7.5 Hz), 3.15 (s, 3H), 3.15-3.07 (m, 1H), 1.22 (t, 3H, *J* = 7.5 Hz); **δ_C** (100 MHz, $CDCl_3$) 158.4, 136.4, 133.7, 128.1, 126.4, 114.3, 55.6, 39.5, 36.9, 18.1; **HRMS** (ESI^+, *m/z* calculated for $[C_{12}H_{19}N_2O_2S]^+$ 255.1162 ($[M+H]^+$), found 255.1156.

***N*-(*N*-(4-Methoxyphenyl)vinylsulfonimidoyl)methanesulfonamide (6a)**

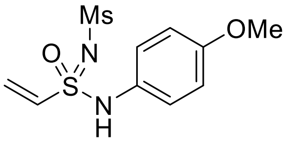

Sulfonimidamide **2d** (40.3 mg, 0.190 mmol, 1.0 equiv.) was added to an oven dried flask and the reaction flask was evacuated and back-filled with nitrogen three times. Anhydrous THF (1.9 mL, 0.1 M) was added and the solution was cooled to 0 °C. Sodium hydride (9.1 mg, 60% dispersion in mineral oil, 0.228 mmol, 1.2 equiv.) was added to solution, followed by the addition of methanesulfonyl chloride (16 µL, 0.209 mmol, 1.1 equiv.). The reaction was stirred for 15 min and warmed to room temperature and stirred for 18 hours. The crude mixture was

filtered through Celite@, washed with EtOAc (30 mL) and concentrated *in vacuo*. Purification was carried out using flash column chromatography (SiO_2, CH_2Cl_2/EtOAc 5:1 to 3:1 to 1:1), to afford the product **6a** as a pale-yellow oil (9.1 mg, 17%).

IR (thin film, ν_{max}/cm^{-1}) 3191, 3082, 3062, 3025, 2952, 2936, 2838, 1606, 1509, 1297, 1251, 1141, 1095, 1030, 971, 830, 805, 766; **δ_H** (400 MHz, $CDCl_3$) 7.22-7.15 (m, 2H), 6.88-6.82 (m, 2H), 6.72 (dd, 1H, *J* = 16.5 Hz, 10.0 Hz), 6.25 (d, 1H, *J* = 16.5 Hz), 6.08 (d, 1H, *J* = 10.0 Hz); 3.79 (s, 3H), 3.22 (s, 3H); **δ_C** (100 MHz, $CDCl_3$) 159.0, 135.0, 130.3, 127.2, 127.0, 114.9, 55.6, 44.8; **LRMS** *m/z* (ESI$^+$) [M-H]$^-$ 289.0; **HRMS** (ESI$^+$, *m/z* calculated for $[C_{10}H_{15}N_2O_4S_2]^+$ 291.0468 ($[M+H]^+$), found 291.0467.

***N*-(*N*-(4-Methoxyphenyl)vinylsulfonimidoyl)acetamide (6b)**

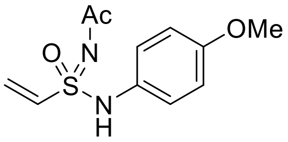

Sulfonimidamide **2d** (18.2 mg, 0.085 mmol, 1.0 equiv.) was added to an oven dried flask and the reaction flask was evacuated and back-filled with nitrogen three times. Anhydrous THF (0.85 mL, 0.10 M) was added and the solution was cooled to 0 °C. Pyridine (10 µL, 0.1275 mmol, 1.5 equiv.) was added to solution, followed by the addition of acetyl chloride (7 µL, 0.094 mmol, 1.1 equiv.). The reaction mixture was warmed to room temperature and stirred for 3 hours. The crude mixture was filtered through Celite@, washed with EtOAc (30 mL) and concentrated *in vacuo*. Purification was carried out using flash column chromatography (SiO_2, CH_2Cl_2/EtOAc, 5:1 to 3:1 to 1:1), to afford the product **6b** as a pale-yellow oil (7.3 mg, 34%).

IR (thin film, ν_{max}/cm^{-1}) 3430, 3033, 2918, 2849, 1655, 1510, 1281, 1026, 1000, 826, 765; **δ_H** (500 MHz, $CDCl_3$) 8.67 (br. s, 1H), 7.17-7.14 (m, 2H), 6.86-6.83 (m, 2H), 6.69 (dd, 1H, *J* = 16.5, 10.0 Hz), 6.20 (d, 1H, *J* = 16.5 Hz), 5.99 (d, 1H, *J* = 10.0 Hz), 3.78 (s, 3H), 2.18 (s, 3H); **δ_C** (126 MHz, $CDCl_3$) 180.0, 158.5, 133.6, 129.3, 127.5, 126.2, 114.8, 55.6, 27.2; **HRMS** (ESI$^+$, *m/z* calculated for $[C_{11}H_{15}N_2O_3S]^+$ 255.0798 ($[M+H]^+$), found 255.0793.

***N*-(*N*-(4-Methoxyphenyl)vinylsulfonimidoyl)butyramide (6c)**

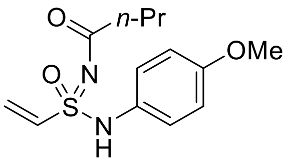

Sulfonimidamide **2d** (31.8 mg, 0.150 mmol, 1.0 equiv.) was added to an oven dried flask and the reaction flask was evacuated and back-filled with nitrogen three times. Anhydrous THF (1.5 mL, 0.10 M) was added and the solution was cooled to 0 °C. Pyridine (18 µL, 0.225 mmol, 1.5 equiv.) was added to solution, followed by the addition of butyryl chloride (17 µL, 0.165 mmol, 1.1 equiv.). The reaction mixture was warmed to room temperature and stirred for 3 hours. The crude mixture was filtered through Celite@, washed with EtOAc (30 mL) and concentrated *in vacuo*. Purification was carried out using flash column chromatography (SiO_2, Petrol/EtOAc, 5:1 to 1:1), to afford the product **6c** as a pale-yellow oil (12.7 mg, 30%).

IR (thin film, ν_{max}/cm^{-1}) 3107, 3066, 3055, 2959, 2933, 2872, 2837, 1606, 1508, 1463, 1362, 1247, 1202, 1095, 1032, 835 ; $\boldsymbol{\delta_H}$ (400 MHz, $CDCl_3$) 7.17-7.12 (m, 2H), 6.86-6.82 (m, 2H), 6.68 (dd, 1H, *J* = 16.5 Hz, 10.0 Hz), 6.19 (d, 1H, *J* = 16.5 Hz), 5.96 (d, 1H, *J* = 10.0 Hz), 3.78 (s, 3H), 2.38 (t, 2H, *J* = 7.5 Hz), 1.68 (sext, 2H, *J* = 7.5 Hz), 0.95 (t, 3H, *J* = 7.5 Hz); $\boldsymbol{\delta_C}$ (100 MHz, $CDCl_3$) 182.8, 163.2, 147.5, 133.7, 129.0, 126.1, 114.8, 55.6, 42.0, 19.1, 13.9; **LRMS** *m/z* (ESI^+) $[M+H]^+$ 283.092, $[M+Na]^+$ 305.071; **HRMS** (ESI^+, *m/z* calculated for $[C_{13}H_{19}N_2O_3S_2Na]^+$ 305.0930 ($[M+Na]^+$), found 305.0930.

***N*-Ethyl-*N*'-(4-methoxyphenyl)ethenesulfonimidamide (6d)**

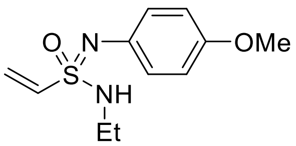

Sulfonimidamide **2d** (45.6 mg, 0.215 mmol, 1.0 equiv.) was added to an oven dried flask and the reaction flask was evacuated and back-filled with nitrogen three times. Anhydrous DMSO (2.15 mL, 0.10 M) and bromoethane (18 µL, 0.237 mmol, 1.1 equiv.) was added, followed by the addition of KHMDS (51.5 mg, 0.258 mmol, 1.2 equiv.). The solution was stirred at room temperature for 2 hours. H_2O (15 mL) and EtOAc (3 × 15 mL) were used for extraction and the organic layer was washed with brine (15 mL), dried over anhydrous Na_2SO_4, filtered and

concentrated *in vacuo*. Purification was carried out using flash chromatography (SiO_2, CH_2Cl_2/EtOAc, 7:1 to 5:1 to 3:1), to afford the product **6d** as a pale-yellow oil (7.8 mg, 19%)

IR (thin film, ν_{max}/cm^{-1}) 3289, 3106, 3060, 2980, 2935, 1509, 1246,1156, 1064, 1031, 897, 834 ; **δ_H** (500 MHz, $CDCl_3$) 7.16-7.13 (m, 2H), 6.89-6.85 (m, 2H), 6.61 (dd, 1H, *J* = 16.5 Hz, 10.0 Hz), 6.19 (d, 1H, *J* = 16.5 Hz), 5.94 (d, 1H, *J* = 10.0 Hz), 3.80 (s, 3H), 3.60 (q, 2H, *J* = 7.0 Hz), 1.07 (t, 3H, *J* = 7.0 Hz); **δ_C** (126 MHz, $CDCl_3$) 159.2, 135.3, 133.0, 130.4, 126.5, 114.5, 55.6, 46.7, 14.7; **HRMS** (ESI^+, *m/z* calculated for $[C_{11}H_{17}N_2O_2S]^+$ 241.1005 ($[M+H]^+$), found 241.0999.

***N*-(*N*-(4-ethynylphenyl)vinylsulfonimidoyl)acetamide (6e)**

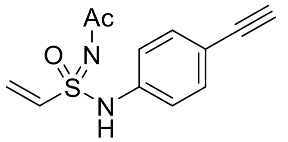

Sulfonimidamide **2c** (31.8 mg, 0.15 mmol, 1.0 equiv.) was added to an oven dried flask and the reaction flask was evacuated and back-filled with nitrogen three times. Anhydrous THF (1.5 mL, 0.10 M) was added and the solution was cooled to 0 °C. Pyridine (18 µL, 0.23 mmol, 1.5 equiv.) was added to the solution, followed by the addition of acetyl chloride (12 µL, 0.17 mmol, 1.1 equiv.). The reaction mixture was warmed to room temperature and stirred for 20 hours. The crude mixture was filtered through Celite®, washed with EtOAc (30 mL) and concentrated in *vacuo*. Purification was carried out using flash column chromatography (SiO_2, Petrol/EtOAc, 2:1 to 1:1), to afford the product **6e** as a pale-yellow oil (9.5 mg, 26%).

Note: This compound is not stable and decomposes gradually at room temperature, therefore a clean NMR spectra was not able to be obtained.

IR (thin film, ν_{max}/cm^{-1}) 3283, 3102, 3087, 3063, 2923, 2847, 2103, 1691, 1627, 1603, 1503, 1366, 1271, 1219, 1176, 1107, 972, 838, 654; **δ_H** (400 MHz, $CDCl_3$) 7.45-7.40 (m, 2H), 7.15-7.11 (m, 2H), 6.67 (dd, 1H, *J* = 16.5 Hz, 10.0 Hz), 6.31 (d, 1H, *J* = 16.5 Hz), 6.03 (d, 1H, *J* = 10.0 Hz), 3.07 (s, 1H), 2.17 (s, 3H); **δ_C** (100 MHz, $CDCl_3$) 179.5, 137.1, 134.1, 133.5, 129.2, 121.9, 119.1, 83.0, 77.8, 27.2; **LRMS** *m/z* (ESI^+) $[M+H]^+$ 249.066; **HRMS** (ESI^+, *m/z* calculated for $[C_{12}H_{13}N_2O_2S]^+$ 249.0692 ($[M+H]^+$), found 249.0694.

_N_-(_N_-(4-Ethynylphenyl)vinylsulfonimidoyl)butyramide (6f)

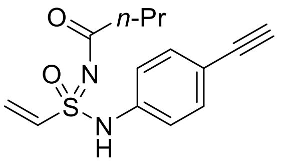

Sulfonimidamide **2c** (26.8 mg, 0.13 mmol, 1.0 equiv.) was added to an oven dried flask and the reaction flask was evacuated and back-filled with nitrogen three times. Anhydrous THF (1.3 mL, 0.10 M) was added and the solution was cooled to 0 °C. Pyridine (17 µL, 0.21 mmol, 1.6 equiv.) was added to the solution, followed by the addition of butyryl chloride (15 µL, 0.14 mmol, 1.1 equiv.). The reaction mixture was warmed to room temperature and stirred for 20 hours. The crude mixture was filtered through Celite®, washed with EtOAc (30 mL) and concentrated *in vacuo*. Purification was carried out using flash column chromatography (Petrol/EtOAc, 2:1 to 1:1), to afford the product **6f** as a pale-yellow oil (10.0 mg, 28%).

IR (thin film, ν_{max}/cm^{-1}) 3286, 3107, 3104, 3089, 2964, 2929, 2873, 2109, 1624, 1605, 1506, 1308, 1201, 1094, 1017, 838, 640; **δ_H** (400 MHz, $CDCl_3$) 7.47-7.42 (m, 2H), 7.16-7.12 (m, 2H), 6.66 (dd, 1H, *J* = 16.5 Hz, 10.0 Hz), 6.31 (d, 1H, *J* = 16.5 Hz), 6.04 (d, 1*H, J* = 10.0 Hz), 3.07 (s, 1H), 2.39 (t, 2H, *J* = 7.5 Hz), 1.68 (sext, 2H, 7.5 Hz), 0.95 (t, 2H, *J* = 7.5 Hz); **δ_C** (100 MHz, $CDCl_3$) 182.3, 136.5, 134.0, 133.6, 129.4, 121.9, 119.5, 82.9, 77.9, 42.0, 19.0, 13.8; **LRMS** *m/z* (ESI^+) $[M+H]^+$ 277.078; **HRMS** (ESI^+, *m/z* calculated for $[C_{14}H_{16}N_2O_2S]^-$ 275.0860 ($[M-H]^-$), found 275.0857.

_N_-(_N_-(4-Ethynylphenyl)vinylsulfonimidoyl)methanesulfonamide (6g)

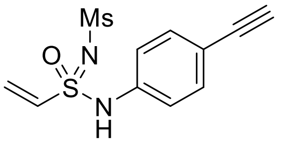

Sulfonimidamide **2c** (31.8 mg, 0.15 mmol, 1.0 equiv.) was added to an oven dried flask and the reaction flask was evacuated and back-filled with nitrogen three times. Anhydrous THF (1.5 mL, 0.10 M) was added and the solution was cooled to 0 °C. Triethylamine (23 µL, 0.23 mmol, 1.5 equiv.) was added to the solution, followed by the addition of methanesulfonyl chloride (13 µL, 0.17 mmol, 1.1 equiv.). The reaction mixture was warmed to room temperature and stirred for 20 hours. The crude mixture was filtered through Celite®, washed

with EtOAc (30 mL) and concentrated *in vacuo*. Purification was carried out using flash column chromatography (SiO_2, Petrol/EtOAc/MeOH, 1:1:0 to 1:3:0 to 0:1:0 to 0:96:4), afforded the product **6g** as a pale-yellow oil (24.7 mg, 58%).

Note: This compound is not stable and decomposes rapidly at room temperature within hours, therefore a clean NMR spectra was not able to be obtained.

IR (thin film, ν_{max}/cm^{-1}) 3279, 3104, 3055, 3041, 2934, 2359, 1601, 1502, 1289, 1259, 1095, 971, 839, 750, 661; **δ_H** (600 MHz, $CDCl_3$) 7.32 (d, 2H, *J* = 8.5 Hz), 7.03 (d, 2H, *J* = 8.5 Hz), 6.59 (dd, 1H, *J* = 16.5 Hz, 9.5 Hz, SC*H*), 6.21 (d, 1H, *J* = 16.5 Hz), 5.91 (d, 1H, *J* = 9.5 Hz), 3.65-3.34 (br s. 1H), 3.09 (s, 3H), 3.04 (s, 1H); **δ_C** (151 MHz, $CDCl_3$) 142.8, 137.0, 133.2, 127.7, 122.3, 116.2, 83.7, 79.7, 43.5; **LRMS** *m/z* (ESI^+) $[M+H]^+$ 285.035; **HRMS** (ESI^+, *m/z* calculated for$[C_{11}H_{11}N_2O_3S_2]^-$ 283.0206 ($[M-H]^-$), found 283.0212.

2.6 Conjugate addition with dodecanethiol

General procedure B:

The method was adapted from C.H. Wu, J. H. Sheu, C. Y. Chen, Y. C. Chien, S. C. Chen, C. H. Pan, C. Y. Huang. USPTO, US2016009642A1, 2016.[8]

The sulfonimidamide or acrylamide (0.100 mmol, 1.0 equiv.) was added to an oven-dried reaction flask. The flask was evacuated and back-filled with nitrogen three times. Anhydrous THF (1.0 mL, 0.1 M) and the solution was cooled to 0 °C. Anhydrous triethylamine (3 µL, 0.02 mmol, 0.2 equiv.) was added to the solution, followed by the addition of 1-dodecanthiol (24 µL, 0.10 mmol, 1.0 equiv.). The reaction flask was warmed to room temperature and stirred for 20 hours. The crude mixture was quenched with saturated $NH_4Cl_{(aq)}$ (3.0 mL), extracted with EtOAc (3 × 5.0 mL), washed with brine (5.0 mL), dried over anhydrous Na_2SO_4 and concentrated *in vacuo*. Purification was carried out using flash column chromatography to afford pure product.

Tabulation of results for conjugate addition with 1-dodecanthiol:

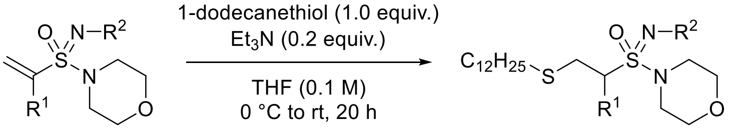

3 **7**

Entry	R^1	R^2	Cmpd.	Yield
1	H	H	**7a**	16%
2	H	Ac	**7b**	18%
3	H	Ms	**7c**	84%
4	H	C(O)NHAr	**7d**	75%
5	H	C(O)*n*-Pr	**7e**	27%
6	H	CO_2Et	**7f**	26%
7	H	p-Tol	**7g**	7%
8	H	*n*-Bu	**7h**	72%
9	H	Et	**7i**	75%
10	Me	H	**7j**	2%
11	Me	Ms	**7k**	6%
12	Me	*n*-Bu	**7l**	3%

Table S2. Detailed result for conjugate addition with 1-dodecanthiol

4-(2-(Dodecylthio)ethylsulfonimidoyl)morpholine (7a)

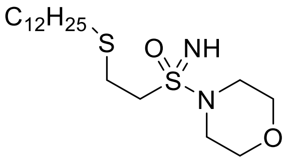

Prepared according to general procedure B, using sulfonimidamide **2a** (17.6 mg, 0.10 mmol, 1.0 equiv.). Purification was carried out using flash column chromatography (SiO_2, Petrol/EtOAc, 3:1 to 1:3 to 0:1), to afford the product **7a** as a white solid (6.0 mg, 16%).

mp 57-59 °C ($CDCl_3$) ; **IR** (thin film, ν_{max}/cm^{-1}) 3264, 2954, 2923, 2835, 1455, 1282, 1245, 1112, 1068, 935, 743, 691; **δ_H** (400 MHz, $CDCl_3$) 3.74-3.72 (m, 4H), 3.33-3.24 (m, 4H), 3.21-3.15 (m, 1*H*), 3.05-2.91 (m, 3H), 2.54 (t, 2H, *J* = 7.5 Hz), 2.17 (s, 1H), 1.63-1.52 (m, 2H), 1.39-1.32 (m, 2H), 1.30-1.19 (m, 16H), 0.89-0.84 (m, 3*H*); **δ_C** (101 MHz, $CDCl_3$) 67.0, 49.1, 46.9, 32.6, 32.0, 29.8, 29.74, 29.70, 29.63, 29.61, 29.5, 29.3, 28.9, 25.5, 22.8, 14.2; **HRMS** (ESI$^+$, *m/z* calculated for $[C_{18}H_{39}N_2O_2S_2]^+$ 379.2447 ($[M+H]^+$), found 379.2446.

N-((2-(Dodecylthio)ethyl)(morpholino)(oxo)- λ⁶-sulfaneylidene)acetamide (7b)

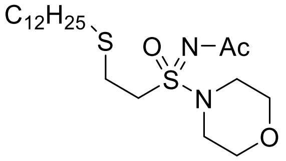

Prepared according to general procedure B, using sulfonimidamide **3a** (21.8 mg, 0.10 mmol, 1.0 equiv.). Purification was carried out using flash column chromatography (SiO_2, Petrol/EtOAc, 3:1 to 1:1 to 1:3), to afford the product **7b** as a colourless oil (7.6 mg, 18%).

IR (thin film, ν_{max}/cm^{-1}) 2954, 2923, 2853, 1644, 1456, 1361, 1256, 1235, 1114, 1071, 938, 832; **δ_H** (400 MHz, $CDCl_3$) 3.81-3.72 (m, 4H), 3.56-3.48 (m, 1*H*), 3.38-3.25 (m, 5H), 2.94-2.82 (m, *2*H), 2.55 (t, 2H, *J* = 7.5 Hz), 2.10 (s, 3H), 1.62-1.55 (m, 2H), 1.39-1.34 (m, 2H), 1.32-1.20 (m, 16H), 0.89-0.86 (m, 3H); **δ_C** (101 MHz, $CDCl_3$) 179.0, 66.6, 51.7, 46.1, 32.6, 32.1, 29.78, 29.77, 29.73, 29.65, 29.54, 29.48, 29.3, 28.9, 27.0, 24.6, 22.8, 14.3; **LRMS** *m/z* (ESI^+) $[M+Na]^+$ 443.2; **HRMS** (ESI^+, *m/z* calculated for $[C_{20}H_{41}N_2O_3S_2]^+$ 421.2553 ($[M+H]^+$), found 421.2554.

N-((2-(Dodecylthio)ethyl)(morpholino)(oxo)-λ⁶-sulfaneylidene)methanesulfonamide (7c)

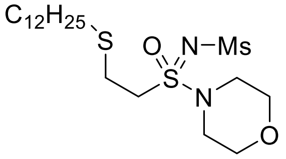

Prepared according to general procedure B, using sulfonimidamide **3e** (25.4 mg, 0.10 mmol, 1.0 equiv.). Purification was carried out using flash column chromatography (SiO_2, Petrol/EtOAc, 3:1 to 1:1 to 1:3), to afford the product **7c** as a white solid (38.4 mg, 84%).

mp 31-33°C ($CDCl_3$); IR (thin film, ν_{max}/cm^{-1}) 2924, 2853, 1456, 1309, 1242, 1147, 1111, 1081, 944, 798, 699; **δ_H** (400 MHz, $CDCl_3$) 3.84-3.73 (m, 4H), 3.52-3.41 (m, 3H), 3.36-3.22 (m, 3*H*), 3.10 (s, 3H), 2.94-2.85 (m, 2H), 2.58-2.50 (m, 2H), 1.63-1.52 (m, 2H), 1.40-1.32 (m, 2H), 1.30-1.18 (m, 16H), 0.90-0.83 (m, 3*H*); **δ_C** (101 MHz, $CDCl_3$) 66.4, 53.0, 46.5, 44.8, 32.7, 32.0, 29.73, 29.70, 29.67, 29.6, 29.5, 29.4, 29.3, 28.9, 24.5, 22.8, 14.2; **LRMS** *m/z* (ESI^-) $[M-H]^-$ 455.1; **HRMS** (ESI^+, *m/z* calculated for $[C_{19}H_{41}N_2O_4S_3]^+$ 457.2223 ($[M+H]^+$), found 457.2222.

1-((2-(Dodecylthio)ethyl)(morpholino)(oxo)-λ^6-sulfaneylidene)-3-(*p*-tolyl)urea (7d)

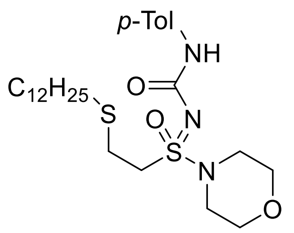

Prepared according to general procedure B, using sulfonimidamide **3d** (30.9 mg, 0.10 mmol, 1.0 equiv.). Purification was carried out using flash column chromatography (SiO_2, Petrol/EtOAc, 5:1 to 3:1), to afford the product **7d** as a white solid (38.4 mg, 75%).

mp 87-89°C (DCM); IR (thin film, ν_{max}/cm^{-1}) 3334, 3076, 3035, 2954, 2920, 2853, 1632, 1594, 1454, 1406, 1288, 1236, 1116, 950, 854, 726, 638; **δ_H** (400 MHz, $CDCl_3$) 7.30 (d, 2H, *J* = 8.0 Hz), 7.07 (d, 2H, *J* = 8.0 Hz), 6.87 (br. s, 1H,), 3.80-3.72 (m, 4H), 3.62-3.53 (m, 1H), 3.43-3.23 (m, 5H), 2.97-2.86 (m, 2H), 2.55 (t, 2H, 7.4 Hz), 2.28 (s, 3H), 1.63-1.54 (m, 2H), 1.41-1.33 (m, 2H), 1.31-1.20 (m, 16H), 0.88 (t, 3H, 6.7 Hz); **δ_C** (101 MHz, $CDCl_3$) 156.0, 136.3, 132.7, 129.5, 119.0, 66.6, 51.9, 46.3, 32.5, 32.0, 29.8, 29.73, 29.69, 29.6, 29.5, 29.4, 29.3, 28.9, 24.7, 22.8, 20.9, 14.2; **LRMS** *m/z* (ESI$^+$) [M+H]$^+$ 512.234; **HRMS** (ESI$^+$, *m/z* calculated for $[C_{26}H_{46}N_3O_3S_2]^+$ 512.2975 ([M+H]$^+$), found 512.2971.

4-(2-(Dodecylthio)-*N*-(*p*-tolyl)ethylsulfonimidoyl)morpholine (7e)

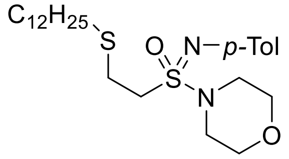

Prepared according to general procedure B, using sulfonimidamide **3j** (26.6 mg, 0.10 mmol, 1.0 equiv.). Purification was carried out using flash column chromatography (SiO_2, Petrol/EtOAc, 5:1 to 3:1 to 1:1), to afford the product **7e** as a colourless oil (3.3 mg, 7%).

IR (thin film, ν_{max}/cm^{-1}) 3075, 3027, 2923, 2853, 1610, 1507, 1310, 1259, 1208, 1113, 1044, 931, 822, 785, 637; **δ_H** (400 MHz, $CDCl_3$) 7.03-6.94 (m, 4H) 3.69-3.54 (m, 4H), 3.41-3.23 (m, 5H), 3.18-3.10 (m, 1H), 3.09-2.94 (m, 2H), 2.57 (t, 2H, *J* = 7.4 Hz), 1.65-1.56 (m, 2H), 1.42-1.34 (m, 2H), 1.31-1.20 (m, 16H), 0.88 (t, 3H, *J* = 6.7 Hz); **δ_C** (101 MHz, $CDCl_3$) 141.0, 131.6, 129.8, 123.3, 66.8, 51.5, 46.7, 32.6, 32.1, 29.81, 29.79, 29.75, 29.68, 29.67, 29.5, 29.4, 29.0, 25.4,

22.8, 20.9, 14.3; **LRMS** *m/z* (ESI$^+$) $[M+H]^+$ 469.238; **HRMS** (ESI$^+$, *m/z* calculated for $[C_{25}H_{45}N_2O_2S_2]^+$ 469.2917 ($[M+H]^+$), found 469.2916.

4-(*N*-Butyl-2-(dodecylthio)ethylsulfonimidoyl)morpholine (7f)

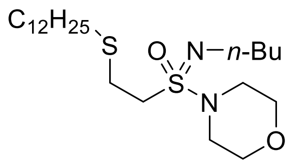

Prepared according to general procedure B, using sulfonimidamide **3g** (23.2 mg, 0.10 mmol, 1.0 equiv.). Purification was carried out using flash chromatography (SiO_2, Petrol/EtOAc, 4:1 to 1:1), to afford the product **7f** as a colourless oil (31.3mg, 72%).

IR (thin film, ν_{max}/cm^{-1}) 2956, 2924, 2854, 1455, 1362, 1330, 1295, 1281, 152, 936, 757, 630; **δ_H** (400 MHz, $CDCl_3$) 3.77-3.68 (m), 3.26-3.14 (m, 5H), 3.31-3.21 (m, 1H), 3.00-2.81 (4H), 2.55-2.51 (m, 2H), 1.61-1.52 (m, 2H), 1.51-1.44 (m, 2H), 1.40-1.32 (m, 4H), 1.30-1.21 (m, 16H), 0.93-0.85 (m, 6H); **δ_C** (101 MHz, $CDCl_3$) 67.0, 49.7, 46.8, 41.6, 34.8, 32.5, 32.1, 29.78, 29.76, 29.74, 29.72, 29.65, 29.5, 29.3, 29.0, 25.4, 22.8, 20.5, 14.3, 14.0; **LRMS** *m/z* (ESI$^+$) $[M+H]^+$ 435.211; **HRMS** (ESI$^+$, *m/z* calculated for $[C_{22}H_{47}N_2O_2S_2]^+$ 435.3073 ($[M+H]^+$), found 435.3081.

4-(1-(Dodecylthio)propan-2-ylsulfonimidoyl)morpholine (7g)

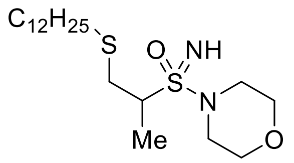

Prepared according to general procedure B, using sulfonimidamide **2f** (19.0 mg, 0.10 mmol, 1.0 equiv.). Purification was carried out using flash column chromatography (SiO_2, Petrol/EtOAc, 3:1 to 1:1 to 1:3), to afford the product **7g** as a colourless oil (0.7 mg, 2%).

IR (thin film, ν_{max}/cm^{-1}) 3277, 2923, 2853, 1652, 1455, 1362, 1256, 1114, 1068, 986, 943, 718, 676; **δ_H** (400 MHz, $CDCl_3$) 3.74-3.66 (m, 4H), 3.40-3.35 (m, 4H), 3.25-3.14 (m, 2H), 2.63-2.47 (m, 3H), 2.17 (br. s, 1H), 1.62-1.53 (m, 2H), 1.49-1.44 (m, 3H), 1.39-1.32 (m, 2H), 1.31-1.20 (m, 16H), 0.91-0.84 (m, 3H); **δ_C** (101 MHz, $CDCl_3$) 67.5, 58.3, 47.3, 33.8, 33.2, 32.1, 29.80, 29.78,

29.74, 29.70, 29.67, 29.5, 29.4, 29.0, 22.8, 14.3, 13.8; **HRMS** (ESI^+, *m/z* calculated for $[C_{19}H_{41}N_2O_2S_2]^+$ 393.2604 ($[M+H]^+$), found 393.2621.

Note: The product was isolated as a mixture of diastereomers where the dr was not determined due to the overlap of the signals.

***N*-((1-(Dodecylthio)propan-2-yl)(morpholino)(oxo)-λ⁶-sulfaneylidene)methanesulfonamide (7h)**

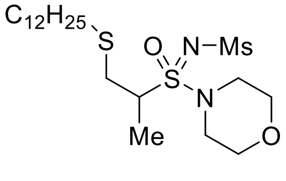

Prepared according to general procedure B, using sulfonimidamide **4b** (26.8 mg, 0.10 mmol, 1.0 equiv.). Purification was carried out using flash column chromatography (SiO_2, Petrol/EtOAc, 3:1 to 1:1 to 1:3), to afford the product **7h** as a colourless oil (2.8 mg, 6%).

IR (thin film, ν_{max}/cm^{-1}) 2954, 2923, 2853, 1457, 1311, 1258, 1147, 1112, 1075, 951, 800, 739; **δ_H** (400 MHz, $CDCl_3$) 3.85-3.69 (m, 4H), 3.55-3.35 (m, 4H), 3.32-3.21 (m, 1H), 3.18-3.07 (m, 4H), 2.63-2.45 (m, 3H), 1.62-1.44 (m, 5H), 1.44-1.31 (m, 2H), 1.31-1.20 (m, 16H), 0.90-0.83 (m, 3H); **δ_C** (101 MHz, $CDCl_3$) 66.6, 66.5, 61.3, 60.4, 47.4, 47.1, 44.9, 44.8, 33.24, 33.21, 32.3, 32.0, 31.9, 29.74, 29.72, 29.68, 29.6, 29.5, 29.4, 29.3, 28.9, 22.8, 14.2, 13.6, 12.5; **HRMS** (ESI^+, *m/z* calculated for $[C_{20}H_{42}N_2O_4S_3Na]^+$ 493.2199 ($[M+Na]^+$), found 493.2199.

Note: The product was isolated as a mixture of diastereomers where the dr was not determined due to the overlap of the signals.

***N*-((2-(Dodecylthio)ethyl)(morpholino)(oxo)-λ⁶-sulfaneylidene)butyramide (7i)**

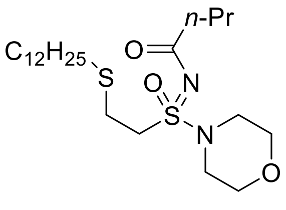

Prepared according to general procedure B, using sulfonimidamide **3b** (24.6 mg, 0.10 mmol, 1.0 equiv.). Purification was carried out using flash column chromatography (SiO_2, Petrol/EtOAc, 5:1 to 3:1 to 1:1), to afford the product **7i** as a colourless oil (12.1 mg, 27%).

IR (thin film, ν_{max}/cm^{-1}) 2958, 2924, 2854, 1643, 1457, 1258, 1235, 1198, 1114, 937, 850, 723; δ_H (400 MHz, $CDCl_3$) 3.79-3.73 (m, 4H), 3.57-3.48 (m, 1H), 3.36-3.25 (m, 5H), 2.92-2.82 (m, 2H), 2.54 (t, 2H, 7.4 Hz), 2.31 (t, 2H. 7.4 Hz), 1.68-1.54 (m, 4H), 1.41-1.33 (m, 2H), 1.32-1.22 (m, 16H), 0.94 (t, 3H, 7.4 Hz), 0.87 (t, 3H, 6.7 Hz); δ_C (101 MHz, $CDCl_3$) 181.7, 66.6, 51.8, 46.1, 41.9, 32.5, 32.0, 29.78, 29.76, 29.7, 29.6, 29.53, 29.47, 29.3, 28.9, 24.6, 22.8, 19.2, 14.3, 14.0; LRMS *m/z* (ESI^+) $[M+H]^+$ 449.243; HRMS (ESI^+, *m/z* calculated for $[C_{22}H_{45}N_2O_3S_2]^+$ 449.2866 ($[M+H]^+$), found 449.2864.

Ethyl ((2-(dodecylthio)ethyl)(morpholino)(oxo)-λ^6-sulfaneylidene)carbamate (7j)

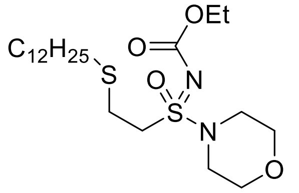

Prepared according to general procedure B, using sulfonimidamide **3c** (24.8 mg, 0.10 mmol, 1.0 equiv.). Purification was carried out using flash column chromatography (SiO_2, Petrol/EtOAc, 3:1 to 1:1 to 1:3), to afford the product **7j** as a colourless oil (11.7 mg, 26%).

IR (thin film, ν_{max}/cm^{-1}) 2955, 2924, 2853, 1670, 1456, 1254, 1114, 1019, 940, 890; **δ_H** (400 MHz, $CDCl_3$) 4.18-4.07 (m, 2H), 3.80-3.70 (m, 4H), 3.52-3.43 (m, 1H), 3.41-3.31 (m, 4H), 3.31-3.21 (m, 1H), 2.98-2.83 (2H), 2.53 (t, 2H, *J* = 7.5 Hz), 1.62-1.52 (m, 2H), 1.41-1.32 (m, 2H), 1.32-1.18 (m, 19H), 0.91-0.83 (m, 3H); **δ_C** (101 MHz, $CDCl_3$) 157.6, 66.6, 62.3, 52.3, 46.3, 32.7, 32.0, 29.8, 29.73, 29.71, 29.64, 29.58, 29.5, 29.3, 28.9, 24.6, 22.8, 14.5, 14.3; **LRMS** *m/z* (ESI^+) $[M+Na]^+$ 473.2; **HRMS** (ESI^+, *m/z* calculated for $[C_{21}H_{43}N_2O_4S_2]^+$ 451.2659 ($[M+H]^+$), found 451.2658.

4-(2-(Dodecylthio)-*N*-ethylethylsulfonimidoyl)morpholine (7k)

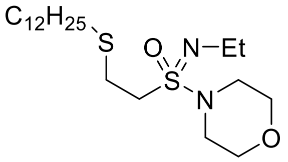

Prepared according to general procedure B, using sulfonimidamide **2h** (20.4 mg, 0.10 mmol, 1.0 equiv.). Purification was carried out using flash column chromatography (SiO_2, Petrol/EtOAc, 5:1 to 3:1 to 1:1), to afford the product **7k** as a colourless oil (30.5 mg, 75%).

IR (thin film, ν_{max}/cm^{-1}) 2956, 2924, 2853, 1453, 1295, 1251, 1155, 1114, 1068, 934, 757, 686; **δ_H** (400 MHz, $CDCl_3$) 3.77-3.67 (m, 4H), 3.27-3.15 (m, 5H), 3.15-3.07 (m, 1H), 3.00-2.84 (m, 4H), 2.55-2.48 (m, 2H), 1.62-1.51 (m, 2H,), 1.39-1.30 (m, 2H), 1.30-1.20 (m, 16H), 1.14 (t, 3H, J = 7.2 Hz) 0.90-0.81 (m, 3H); **δ_C** (101 MHz, $CDCl_3$) 66.9, 49.6, 46.8, 36.7, 32.5, 32.0, 29.8, 29.73, 29.72, 29.70, 29.6, 29.5, 29.3, 28.9, 25.3, 22.8, 18.1, 14.2; **LRMS** *m/z* (ESI^+) $[M+H]^+$ 407.225; **HRMS** (ESI^+, *m/z* calculated for $[C_{20}H_{43}N_2O_2S_2]^+$ 407.2760 ($[M+H]^+$), found 407.2755.

4-(*N*-Butyl-1-(dodecylthio)propan-2-ylsulfonimidoyl)morpholine (7l)

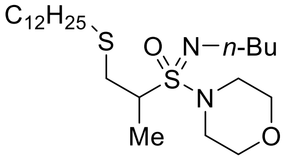

Prepared according to general procedure B, using sulfonimidamide **4c** (23.3 mg, 0.10 mmol, 1.0 equiv.). Purification was carried out using flash column chromatography (SiO_2. Petrol/EtOAc, 7:1 to 3:1 to 1:1), to afford the product **7l** as a colourless oil (1.3 mg, 3%).

IR (thin film, ν_{max}/cm^{-1}) 2956, 2924, 2853, 1456, 1374, 1297, 1212, 1149, 1068, 943, 733; **δ_H** (400 MHz, $CDCl_3$) 3.74-3.64 (m, 4H), 3.37-3.24 (m, 5H), 3.21-3.12 (m, 1H), 3.12-3.03 (dt, 1H, *J* = 12.1 Hz, 6.8 Hz), 2.88-2.77 (dt, 1H, *J* = 12.1 Hz, 6.8 Hz), 2.59-2.45 (m, 3H), 1.61-1.52 (m, 2H,), 1.52-1.44 (m, 2H) 1.41-1.31 (m, 7H), 1.31-1.21 (m, 16H, C_8H_{16}), 0.93-0.83 (m, 6H); **δ_C** (101 MHz, $CDCl_3$) 67.4, 58.5, 47.1, 41.8, 34.9, 33.7, 33.0, 32.1, 29.79, 29.77, 29.74, 29.70, 29.66, 29.5, 29.4, 29.0, 22.8, 20.5, 14.3, 14.1, 13.5; **LRMS** *m/z* (ESI^+) $[M+H]^+$ 449.214; **HRMS** (ESI^+, *m/z* calculated for $[C_{23}H_{49}N_2O_2S_2]^+$ 449.3230 ($[M+H]^+$), found 449.3223.

Note: The product was isolated as a mixture of diastereomers where the dr was not determined due to the overlap of the signals.

3-(Dodecylthio)-1-morpholinopropan-1-one (7m)

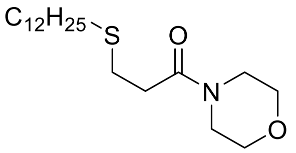

Prepared according to general procedure B, using 4-acryloylmorpholine **8** (13 µL, 0.10 mmol, 1.0 equiv.). Purification was carried out using flash column chromatography (SiO_2, Petrol/EtOAc, 3:1 to 1:1 to 0:1), to afford the product **7m** as a colourless oil (2.1 mg, 6%).

IR (thin film, ν_{max}/cm^{-1}) 2923, 2852, 1650, 1433, 1299, 1208, 1116, 1026, 911, 852; **δ_H** (400 MHz, $CDCl_3$) 3.71-3.65 (m, 4H), 3.60-3.64 (m, 2H), 3.49-3.45 (m, 2H), 2.85-2.80 (m, 2H),2.61-2.57 (m, 2H), 2.56-2.52 (m, 2H), 1.62-1.56 (m, 2H), 1.39-1.33 (m, 2H), 1.29-1.23 (m, 16H), 0.88 (t, 3H, J = 7.0 Hz); **δ_C** (101 MHz, $CDCl_3$) 162.0, 67.0, 66.8, 42.2, 33.7, 32.8, 32.1, 29.83, 29.81, 29.76, 29.7, 29.5, 29.4, 29.1, 27.6, 22.8, 14.3; **LRMS** *m/z* (ESI$^+$) $[M+H]^+$ 344.195; **HRMS** (ESI$^+$, *m/z* calculated for $[C_{19}H_{38}NO_2S]^+$ 344.2618 ($[M+H]^+$), found 344.2614.

2.7 Procedure for competition reaction with 1-dodecanethiol

The sulfonimidamide (0.10 mmol, 1.0 equiv.) was added to an oven-dried reaction flask. The flask was evacuated and back-filled with nitrogen three times. 4-Acryloylmorpholine **8** (13 µL, 0.100 mmol, 1.0 equiv.) was added and the mixture was dissolved in anhydrous THF (1.0 mL) and the solution was cooled to 0 °C. Anhydrous triethylamine (3 µL, 0.02 mmol, 0.2 equiv.) was added to the solution, followed by the addition of 1-dodecanethiol (24 µL, 0.10 mmol, 1.0 equiv.). The reaction flask was warmed to room temperature and stirred for 20 hours. The crude mixture was quenched with $NH_4Cl_{(aq)}$ (3.0 mL), extracted with EtOAc (3 × 5.0 mL), washed with brine (5.0 mL), dried over anhydrous Na_2SO_4, filtered and concentrated *in vacuo*. Purification was carried out using flash column chromatography to afford the products with matching analytical data to those reported above.

2.8 Procedure for conjugate addition reactions with amino acids

General procedure C:

Method adapted from H. Chen, R. Huang, Z. Li, W. Zhu, J. Chen, Y. Zhan, B. Jiang, *Org. Biomol. Chem.*, 2017, **15**, 7339-7345).[9]

The respective sulfonimidamide (1.0 equiv.) was added to an oven dried reaction flask. The flask was evacuated and back-filled with nitrogen three times. Anhydrous MeCN (0.1 M) was added, followed by the addition of anhydrous triethylamine (0.2 - 2.0 equiv.) and amino acid (1.0 equiv.). The reaction mixture was stirred at room temperature for 16 hours. The crude mixture was quenched with $NaHCO_3$(aq), extracted with EtOAc, washed with brine, dried over anhydrous Na_2SO_4, filtered and concentrated *in vacuo*. Purification was carried out using flash column chromatography to afford the product.

Methyl *N*-(*tert*-butoxycarbonyl)-*S*-(2-(morpholine-4-sulfonimidoyl)ethyl)-*L*-cysteinate (9a)

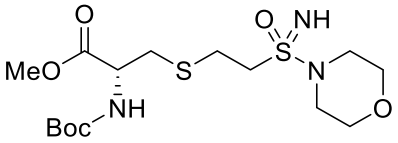

9a

Prepared according to general procedure C, using sulfonimidamide **2a** (15.8 mg, 0.09 mmol, 1.0 equiv.), anhydrous triethylamine (12.5 µL, 0.09 mmol, 1.0 equiv.), Boc-Cys-OMe (21.1 mg, 0.09 mmol, 1.0 equiv.) and MeCN (0.9 mL). Purification was carried out using flash column chromatography (SiO_2, Petrol/EtOAc, 1:3 to 0:1), to afford the product **9a** as a colourless oil (20 mg, 54%, mixture of diastereomers).

IR (thin film, ν_{max}/cm^{-1}) 3320, 3282, 2978, 1747, 1713, 1519, 1367, 1248, 1169, 1114, 939, 738; **δ_H** (500 MHz, $CDCl_3$) 5.37 (d, 1H, *J* = 7.9 Hz), 4.55 (br. s, 1H), 3.79 (s, 3H), 3.74 (t, 4H, *J* = 4.6 Hz), 3.32-3.25 (m, 4H), 3.19 (ddd, 1H, *J* = 14.1 Hz, 7.1 Hz, 3.9 Hz), 3.05-2.95 (m, 5H), 2.16 (br. s, 1H), 1.45 (s, 9 H); **δ_C** (126 MHz, $CDCl_3$) 171.4, 155.3, 80.5, 67.0, 66.7, 53.5, 52.9, 48.98, 48.96, 47.0, 46.9, 35.19, 35.16, 28.4, 26.28, 26.25; **HRMS** (ESI$^+$, *m/z* calculated for $[C_{15}H_{30}N_3O_6S_2]^+$ 412.1571 ($[M+H]^+$), found 412.1571.

Note: The product was isolated as a mixture of diastereomers where the dr was not determined due to the overlap of the signals.

Methyl *N*-(*tert*-butoxycarbonyl)-*S*-(2-(*N*-(methylsulfonyl)morpholine-4-sulfonimidoyl)ethyl)-*L*-cysteinate (9b)

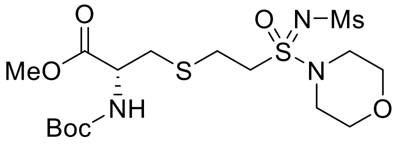

9b

Prepared according to general procedure C, using sulfonimidamide **3e** (25.4 mg, 0.10 mmol, 1.0 equiv.), anhydrous triethylamine (14 µL, 0.10 mmol, 1.0 equiv.), Boc-Cys-OMe (23.5 mg, 0.10 mmol, 1.0 equiv.) and MeCN (1.0 mL). Purification was carried out using flash column chromatography (SiO_2, Petrol/EtOAc, 1:1 to 1:3 to 0:1), to afford the product **9b** as a colourless oil (36.6 mg, 89%, mixture of diastereomers).

IR (thin film, ν_{max}/cm^{-1}) 3380, 2981, 1747, 1713, 1517, 1367, 1247, 1149, 1112, 951, 737; **δ_H** (500 MHz, $CDCl_3$) 5.41-5.26 (m, 1H), 4.57-4.41 (m, 1H), 3.82-3.74 (m, 7H), 3.46-3.25 (m, 6H), 3.08 (s, 3H), 3.05-3.00 (m, 1 H), 2.97-2.89 (m, 3 H), 1.43 (s, 9H); **δ_C** (126 MHz, $CDCl_3$) 171.2, 155.2, 80.6, 66.3, 53.5, 53.4, 52.9, 52.7, 52.6, 46.4, 44.8. 35.2, 28.4, 25.2, 25.0; **HRMS** (ESI^+, *m/z* calculated for $[C_{16}H_{32}N_3O_8S_3]^+$ 490.1346 ($[M+H]^+$), found 490.1338.

Note: The product was isolated as a mixture of diastereomers where the dr was not determined due to the overlap of the signals.

Methyl *N*-(*tert*-butoxycarbonyl)-*S*-(2-(*N*-butylmorpholine-4-sulfonimidoyl)ethyl)-*L*-cysteinate (9c)

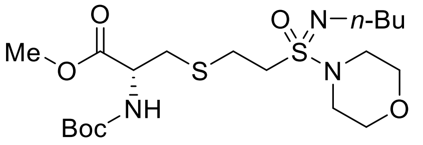

9c

Prepared according to general procedure C, using sulfonimidamide **3g** (18.6 mg, 0.08 mmol, 1.0 equiv.), anhydrous triethylamine (11.1 µL, 0.08 mmol, 1.0 equiv.), Boc-Cys-OMe (18.8 mg, 0.08 mmol, 1.0 equiv.) and MeCN (0.8 mL). Purification was carried out using flash column chromatography (SiO_2, Petrol/EtOAc, 1:1 to 1:3 to 0:1), to afford the product **9c** as a colourless oil (32.9 mg, 88%, mixture of diastereomers).

IR (thin film, ν_{max}/cm^{-1}) 3344, 2958, 1749, 1716, 1517, 1455, 1364, 1255, 1167, 1068, 937, 736; **δ_H** (400 MHz, $CDCl_3$) 5.43-5.37 (m, 1H), 4.56-4.52 (m, 1H), 3.77 (s, 3H), 3.76-3.69 (m, 4H), 3.25-3.14 (m, 5 H), 3.08-2.89 (m, 6H), 2.86-2.81 (m, 1H), 1.50-1.46 (m, 2H), 1.44 (s, 9 H), 1.38-1.31 (m, 2H), 0.89 (t, 3H, *J* = 7.3 Hz); **δ_C** (101 MHz, $CDCl_3$) 171.4, 155.3, 80.0, 66.9, 53.49, 53.45, 52.8, 49.5, 49.4, 46.7, 41.6, 35.2, 35.1, 34.8, 34.7, 28.4, 26.21, 26.18, 20.5, 14.0; **HRMS** (ESI$^+$, *m/z* calculated for $[C_{19}H_{38}N_3O_6S_2]^+$ 468.2197 ($[M+H]^+$), found 468.2206.

Note: The product was isolated as a mixture of diastereomers where the dr was not determined due to the overlap of the signals.

Methyl *N*^{2}-(*tert*-butoxycarbonyl)-*N*^{6}-(2-(morpholine-4-sulfonimidoyl)ethyl)-*L*-lysinate (10a) & methyl *N*^{2}-(*tert*-butoxycarbonyl)-*N*^{6},*N*^{6}-bis(2-(morpholine-4-sulfonimidoyl)ethyl)-*L*-lysinate (10aa)

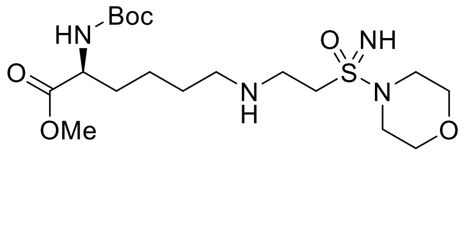

10a

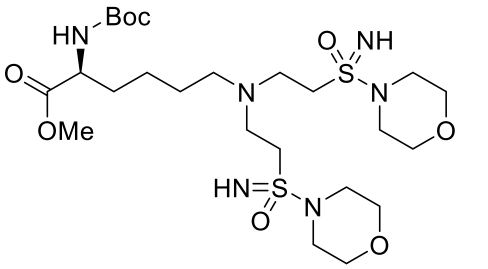

10aa

Prepared according to general procedure C, using sulfonimidamide **2a** (15.8 mg, 0.09 mmol, 1.0 equiv.), anhydrous triethylamine (25 µL, 0.18 mmol, 2.0 equiv.), Boc-Lys-OMe·HCl (26.7 mg, 0.09 mmol, 1.0 equiv.) and MeCN (0.9 mL). Purification was carried out using flash column chromatography (SiO_2, CH_2Cl_2/MeOH, 50:1 to 20:1 to 10:1), to afford the product **10a** as a colourless oil (20.8 mg, 53%, mixture of diastereomers) and **10aa** as a colourless oil (7.2 mg, 13%, mixture of diastereomers).

10a **IR** (thin film, ν_{max}/cm^{-1}) 3220, 2980, 1743, 1712, 1521, 1368, 1250, 1168, 1048, 940; **δ_H** (500 MHz, $CDCl_3$) 5.17-5.10 (m, 1H), 4.30-4.25 (m, 1H), 3.91-3.73 (m, 8 H), 3.47 (s, 1H), 3.40-3.33 (m, 1H), 3.30-3.26 (m, 3H), 3.25-3.19 (m, 1H), 3.11-2.96 (m, 1H), 2.87-2.67 (m, 2H), 1.81-1.77 (m, 1H), 1.69-1.57 (m, 3H), 1.43-1.39 (m, 12 H), 1.27-1.19 (m, 1H); **δ_C** (126 MHz, $CDCl_3$) 173.4, 155.5, 80.2, 67.7, 66.9, 53.5, 53.3, 52.5, 52.4, 49.1, 49.0, 47.5, 46.9, 46.3, 44.4, 43.8, 32.6, 29.8, 29.5, 29.1, 28.5, 23.0; **HRMS** (ESI$^+$, *m/z* calculated for $[C_{18}H_{36}N_4O_6SNa]^+$ 459.2248 ($[M+Na]^+$), found 459.2251.

Note: The product was isolated as a mixture of diastereomers where the dr was not determined due to the overlap of the signals.

10aa IR (thin film, v_{max}/cm^{-1}) 3303, 2979, 1742, 1713, 1522, 1455, 1393, 1250, 1114, 1047, 939; **δ_H** (500 MHz, $CDCl_3$) 5.15-5.08 (m, 1H), 4.28 (d, 1H, *J* = 7.4 Hz), 3.77-3.67 (m, 11H), 3.33-3.23 (m, 6H), 3.18-2.97 (m, 5H), 2.95-2.87 (m, 2H), 2.52-2.44 (m, 4H), 2.30 (br. s, 1H), 1.80 (br. s, 2H), 1.66-1.61 (m, 1H), 1.55-1.48 (m, 2H), 1.44 (s, 9H), 1.37-1.31 (m, 1H), 1.25-1.24 (m, 2H); **δ_C** (126 MHz, $CDCl_3$) 173.4, 155.6, 80.1, 67.1, 67.05, 67.05, 66.97, 53.83, 53.75, 53.7, 53.5, 53.4, 52.6, 52.4, 50.8, 47.9, 47.91, 47.89, 47.87, 46.86, 46.85, 45.9, 45.6, 43.3, 32.7, 32.1, 29.8, 28.5, 26.8, 23.1 ; **HRMS** (ESI$^+$, *m/z* calculated for $[C_{24}H_{49}N_6O_8S_2]^+$ 613.3048 ($[M+H]^+$), found 613.3069.

Note: The product was isolated as a mixture of diastereomers where the dr was not determined due to the overlap of the signals.

Methyl *N*^{2}-(*tert*-butoxycarbonyl)-*N*^{6}-(2-(*N*-(methylsulfonyl)morpholine-4-sulfonimidoyl)ethyl)-*L*-lysinate (10b)

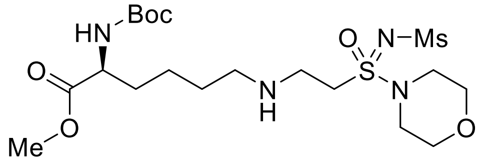

10b

Prepared according to general procedure C, using sulfonimidamide **3e** (25.4 mg, 0.10 mmol, 1.0 equiv.), anhydrous triethylamine (28 µL, 0.20 mmol, 2.0 equiv.), Boc-Lys-OMe.HCl (29.7 mg, 0.10 mmol, 1.0 equiv.) and MeCN (1.0mL). Purification was carried out using flash column chromatography (SiO_2, CH_2Cl_2/MeOH, 50:1 to 20:1 to 10:1), to afford the product **10b** as a colourless oil (50.4 mg, 98%, mixture of diastereomers).

IR (thin film, v_{max}/cm^{-1}) 3351, 3334, 2979, 1742, 1709, 1521, 1456, 1304, 1250, 1164, 1111, 945, 736; **δ_H** (500 MHz, $CDCl_3$) 5.15-5.10 (m, 1H), 4.29-4.24 (m, 1H), 3.82-3.73 (m, 4H), 3.71 (s, 3H), 3.46-3.28 (m, 5H), 3.26-3.19 (m, 1H), 3.11-3.04 (m, 5H), 2.58 (t, 2H, *J* = 7.0 Hz), 1.82-1.73 (m, 1H), 1.66-1.57 (m, 1H), 1.53-1.30 (m, 14H); **δ_C** (126 MHz, $CDCl_3$) 173.3, 155.5, 80.0, 66.3, 53.3, 52.6, 52.5, 52.4, 49.3, 46.3, 44.8, 43.1, 32.6, 29.5, 28.4, 23.1; **HRMS** (ESI$^+$, *m/z* calculated for $[C_{19}H_{39}N_4O_8S_2]^+$ 515.2204 ($[M+H]^+$), found 515.2205.

Note: The product was isolated as a mixture of diastereomers where the dr was not determined due to the overlap of the signals.

Methyl *N*[2]-(*tert*-butoxycarbonyl)-*N*[6]-(2-(*N*-butylmorpholine-4-sulfonimidoyl)ethyl)-*L*-lysinate (10c) & methyl *N*[2]-(*tert*-butoxycarbonyl)-*N*[6],*N*[6]-bis(2-(*N*-butylmorpholine-4-sulfonimidoyl)ethyl)-*L*-lysinate (10cc)

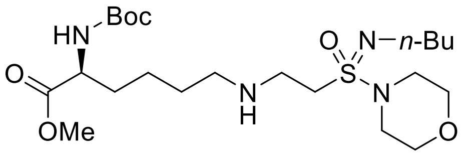

10c

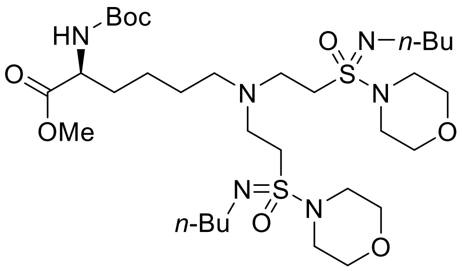

10cc

Prepared according to general procedure C, using sulfonimidamide **3g** (18.6 mg, 0.08 mmol, 1.0 equiv.), anhydrous triethylamine (22.3 µL, 0.16 mmol, 2.0 equiv.), Boc-Lys-OMe.HCl (23.7 mg, 0.08 mmol, 1.0 equiv.) and MeCN (0.8 mL). Purification was carried out using flash column chromatography (SiO_2, CH_2Cl_2/MeOH, 50:1 to 20:1 to 10:1), to afford the product **10c** as a colourless oil (13.4 mg, 34%, mixture of diastereomers) and **10cc** as a colourless oil (11.6 mg, 11.6%, mixture of diastereomers).

10c IR (thin film, v_{max}/cm^{-1}) 3368, 3344, 2957, 2936, 1746, 1714, 1520, 1456, 1392, 1298, 1257, 1169, 940, 738; **δ_H** (500 MHz, $CDCl_3$) 5.11-5.08 (m, 1H), 4.28 (d, 1H, *J* = 7.4 Hz), 3.77-3.69 (m, 7H), 3.49 (br. s, 1H), 3.30-3.17 (m, 6H), 3.10-3.04 (m, 1H), 2.92-2.68 (m, 4H), 1.81 (br. s, 1H), 1.68-1.61 (m, 3H), 1.50-1.31 (m, 16 H), 0.91 (t, 3H, *J* = 7.3Hz); **δ_C** (126 MHz, $CDCl_3$) 173.3, 155.6, 80.1, 66.8, 53.3, 52.5, 48.6, 46.8 46.7, 43.4, 41.3, 34.71, 34.69, 32.6, 28.5, 23.0, 20.5, 14.0; **HRMS** (ESI^+, *m/z* calculated for $[C_{22}H_{45}N_4O_6S]^+$ 493.3054 ($[M+H]^+$), found 493.3056.

Note: The product was isolated as a mixture of diastereomers where the dr was not determined due to the overlap of the signals.

10cc IR (thin film, v_{max}/cm^{-1}) 3368, 2958, 1746, 1714, 1519, 1455, 1336, 1274, 1154, 1069, 938, 737; **δ_H** (500 MHz, $CDCl_3$) 5.08 (d, 1H, *J* = 8.4 Hz), 4.28 (d, 1H, *J* = 6.6 Hz), 3.77-3.70 (m, 11H), 3.24-3.15 (m, 8 H), 3.08-2.98 (m, 6 H), 2.89-2.81 (m, 4 H), 2.44 (br. s, 2H), 1.77 (br. s, 4 H), 1.51-1.45 (m, 6H), 1.44 (s, 9 H), 1.39-1.31 (m, 6 H), 0.92-0.88 (m, 6H); **δ_C** (126 MHz, $CDCl_3$)

173.4, 155.5, 80.0, 66.9, 53.7, 53.5, 52.4, 47.7, 47.6, 46.8, 46.1, 46.0, 41.6, 34.8, 32.8, 28.5, 23.1, 20.5, 14.0; **HRMS** (ESI$^+$, *m/z* calculated for $[C_{32}H_{65}N_6O_8S_2]^+$ 725.4300 ($[M+H]^+$), found 725.4305.

Note: The product was isolated as a mixture of diastereomers where the dr was not determined due to the overlap of the signals.

2.9 Procedure for competition reactions of Lys- and Cys-derivatives

The respective sulfonimidamide (0.1 mmol, 1.0 equiv.) was added to an oven dried reaction flask. The flask was evacuated and back-filled with nitrogen three times. Anhydrous CH_3CN (1.0 mL, 0.1 M) was added, followed by the addition of anhydrous triethylamine (0.1 mmol, 1.0 equiv.). Boc-Lys-OMe (0.1 mmol, 1.0 equiv.) and Boc-Cys-OMe (0.1 mmol, 1.0 equiv.) were dissolved in anhydrous CH_3CN (0.3 mL) and added to the solution containing sulfonimidamide. The reaction mixture was stirred at room temperature for 16 hours. The crude mixture was quenched with $NaHCO_3$(aq) (3.0 mL), extracted with EtOAc (3 x 5 mL), washed with brine, dried over anhydrous Na_2SO_4, filtered and concentrated *in vacuo*. Purification was carried out using flash column chromatography (SiO_2, CH_2Cl_2/MeOH, 50:1 to 20:1 to 10:1), to afford the products with matching analytical data to those reported above.

2.10 Procedure for determination of half-life for the conjugate addition of glutathione

General procedure D:

The rate determination reaction was conducted using nuclear magnetic resonance (NMR) spectroscopy. ^{1}H NMR spectra were obtained on a Bruker AVIIIHD 500 (500MHz) using 5 mm NMR tubes at 20 °C. The spectra were recorded using zg60 pulse program with a spectral width of 10000 Hz and a relaxation time of 1 second. Due to the extremely fast reaction rate of some reactive substrates, for majority of the substates, each acquisition consisted of 1 scan with an increasing interval time from 0 second to 120 seconds for a total elapsed time of 2 hours. Consumption of starting material was determined by monitoring the disappearance of alkene proton signals (6.0 to 6.5 ppm) relative to a sodium acetate internal standard.

Reaction was conducted in a mixture of buffer solution and deuterated DMSO (Buffer:$(CD_3)_2SO$ = 2:1). A relatively high $(CD_3)_2SO$ ratio was employed due to the insolubility of less polar substrates in the buffer solution.

Buffer solution containing 0.0648 mol/L disodium hydrogen phosphate and 0.0153 mol/L potassium dihydrogen phosphate at pH 7.3[10] was prepared using deuterium oxide and stored at 20 °C. The prepared buffer solution had a shelf life of one month. The stock solution of sodium acetate (0.1 M) was prepared using the buffer solution. Glutathione stock solution (0.1 M) was freshly prepared before each experiment, stored under argon and used within 20 minutes to minimise the effect of background oxidation of glutathione. The sulfonimidamide was dissolved in $(CD_3)_2SO$ to form a stock solution (0.1 M).

The procedure for the kinetic study was as follows: sulfonimidamide stock solution (100 µL, 0.1 M in $(CD_3)_2SO$, 0.01 mmol, 1.0 equiv.) and sodium acetate solution (100 µL, 0.01 mmol, 1.0 equiv.) were added sequentially to the NMR tube. $(CD_3)_2SO$ (0.3 mL) and buffer solution were added (0.5 mL) to give an overall volume of 0.8 mL. Locking and shimming of the reaction mixture was performed, and a spectrum was obtained to check the concentration of electrophile before the addition of corresponding amount of glutathione solution (0.01 mmol, 1.0 equiv., 0.1 mL) such that the acquisition could be started right after the addition of glutathione.

For the majority of substrates, the half-life was determined using second order kinetics with 1.0 equivalents of glutathione used as the nucleophile. The consumption of starting material was measured and plotted as the inverse against time. The half-life was calculated through the equation obtained from the best-fitted line. An example plot is shown below for compound **2a** (**Figure S1**).

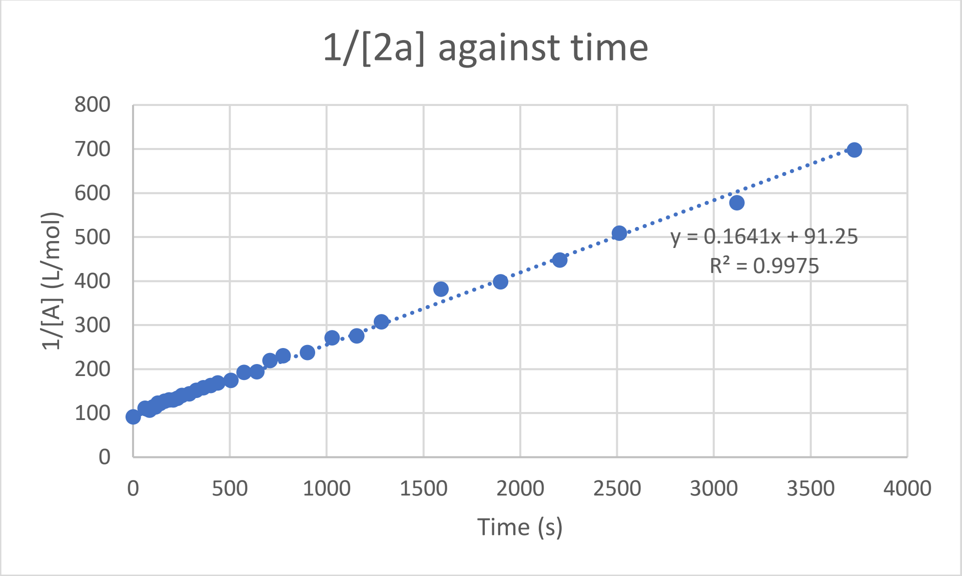

Figure S1: 1/[2a] against time

For substrates with much lower reactivity, such as **2f** and (*E*)-**2h**, half-life was calculated through pseudo-first order kinetic using excess glutathione (6.0 equiv.). The concentration of starting material was plotted as natural log and the half-life was calculated through the best-fitted line obtained. An example plot is shown below for compound **2f** (**Figure S2**).

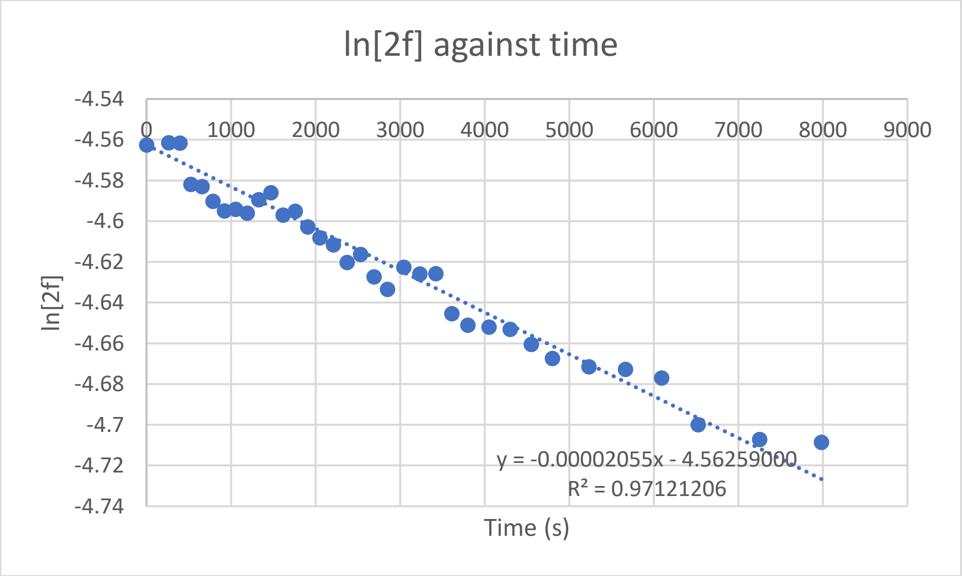

Figure S2: ln[2f] against time

Tabulation of half-lives for all substrates:

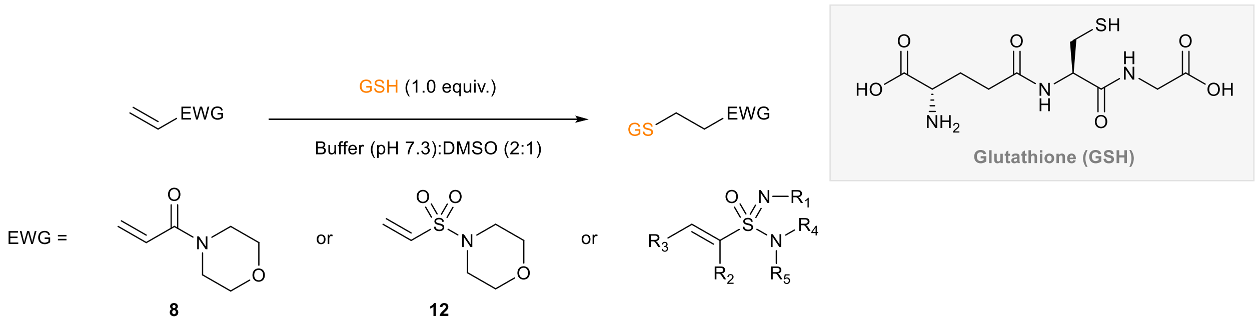

Compound	R_1	R_2	R_3	NR_4R_5	Kinetics	Half-life (s)
**2a**	H	H	H	Morpholine	Second order	576
**3a**	Ac	H	H	Morpholine	Second order	1.01
**3b**	Butyryl	H	H	Morpholine	Second order	9.88
**3c**	CO_2Et	H	H	Morpholine	Second order	4.22
**3d**	*p*-Tolyl urea	H	H	Morpholine	Second order	28.8
**3e**	Ms	H	H	Morpholine	Second order	n.d.
**3g**	*n*-Bu	H	H	Morpholine	Second order	162
**3h**	Et	H	H	Morpholine	Second order	161
**3i**	*t*-Bu	H	H	Morpholine	Second order	403
**3j**	*p*-Tol	H	H	Morpholine	Second order	532
**2d**	H	H	H	PMP	Second order	1065
**6a**	Ms	H	H	PMP	Second order	274
**6b**	Ac	H	H	PMP	Second order	81.7
**6d**	Et	H	H	PMP	Second order	842
**2e**	H	H	H	NMe-PMP	Second order	n.d.
**5a**	Ms	H	H	NMe-PMP	Second order	n.d
**5b**	Ac	H	H	NMe-PMP	Second order	3.20
**5c**	Et	H	H	NMe-PMP	Second order	196
**2f**	H	Me	H	Morpholine	Pseudo-first order	35873
**4a**	Ac	Me	H	Morpholine	Second order	4680
**4b**	Ms	Me	H	Morpholine	Second order	249
**4c**	*n*-Bu	Me	H	Morpholine	Second order	11023
(*E*)-**2h**	H	H	Me	Morpholine	Pseudo-first order	16379
**4d**	Ac	H	Me	Morpholine	Second order	1549

**4e**	Ms	H	Me	Morpholine	Second order	218
**4f**	*n*-Bu	Me	Me	Morpholine	Second order	9307
**8**	-	-	-	-	Second order	14259
**12**	-	-	-	-	Second order	232

Table S3: Half-life calculated for different substrates

For sulfonimidamides with appended electron-withdrawing groups, there was a positive correlation between the rate of reaction and increase in the β-carbon 13C NMR shift (**Figure S3**)

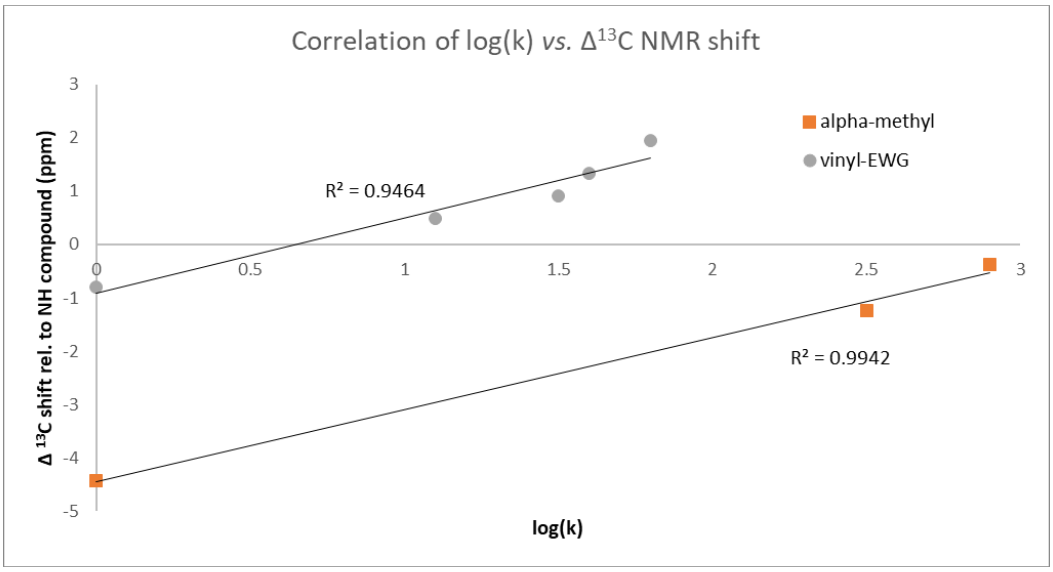

Figure S3. Correlation of log(k) versus Δ13C NMR shift

4-Acryloylmorpholine-GSH adduct (13a)

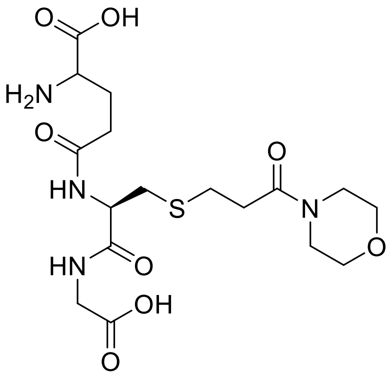

z

Prepared according to general procedure D, using 4-acryloylmorpholine **8**, except that each acquisition consisted of 4 scans with a relaxation time of 50 seconds, 1200 seconds interval time and the total elapsed time was 6 hours. The half-life was calculated using the data within the first 3.5 hours. The half-life calculated from two separate experiments were 13923 seconds and 14595 seconds respectively, with an averaged value of 14259 seconds.

HRMS (ESI$^+$), *m/z* calculated for $[C_{17}H_{27}N_4O_8SD_2]^+$ 451.1826 ($[M\text{-}H+2D]^+$), found 451.1824.

Sulfonimidamide-GSH Adduct (13b)

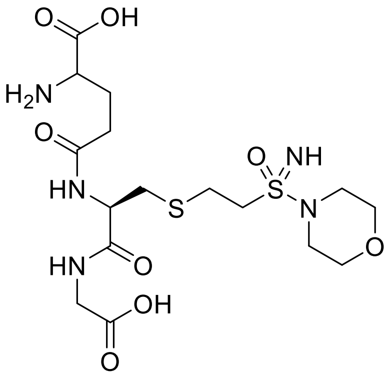

Prepared according to general procedure D, using 4-(vinylsulfonimidoyl)morpholine **2a**. The half-life calculated from three experiments were 584 seconds, 588 seconds and 556 seconds respectively, the average half-life calculated was 576 seconds (standard deviation 17 seconds).

δ_H (500 MHz, D_2O) 4.64 (dd, 1H, *J* = 8.5 Hz, 5.2Hz), 3.98 (s, 2H), 3.85-3.77 (m, 5H), 3.49-3.41 (m, 2H), 3.38-3.28 (m, 4H), 3.16-3.12 (m, 1H), 3.05-2.91 (m, 3H), 2.55 (m, 2H), 2.18 (m, 2H);

δ_C (126 MHz, $CDCl_3$) 174.8, 173.7, 173.6, 172.4, 66.5, 53.9, 52.94, 52.88, 49.2, 49.1, 46.1, 41.7, 33.02, 32.96, 31.2, 26.0, 24.84, 24.76; **HRMS** (ESI^+), *m/z* calculated for $[C_{16}H_{28}N_5O_8S_2D]^+$ 485.1593 ($[M+D]^+$), found 485.1594.

Sulfonimidamide-GSH Adduct (13c)

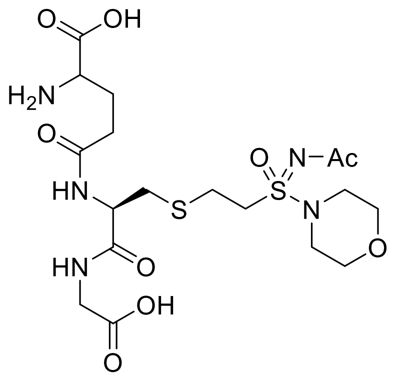

Prepared according to general procedure D, using *N*-(morpholino(oxo)(vinyl)-λ^6-sulfaneylidene)acetamide **3a**. The half-life calculated from two experiments were 1.06 second and 0.96 second respectively, the average half-life calculated was 1.01 seconds.

HRMS (ESI^+), *m/z* calculated for $[C_{18}H_{32}N_5O_9S_2]^+$ 526.1636 ($[M+H]^+$), found 526.1639.

Sulfonimidamide-GSH Adduct (13d)

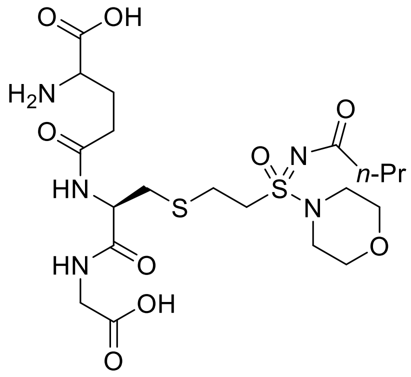

Prepared according to general procedure D, using *N*-(morpholino(oxo)(vinyl)-λ^6-sulfaneylidene)butyramide **3b**. The half-life calculated from two experiments were 8.36 seconds and 11.4 seconds respectively, the average half-life calculated was 9.88 seconds.

HRMS (ESI^+), *m/z* calculated for $[C_{20}H_{34}N_5O_9S_2D_2]^+$ 556.2075 ($[M-H+2D]^+$), found 556.2079.

Sulfonimidamide-GSH Adduct (13e)

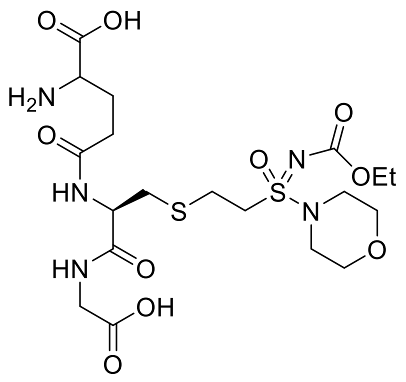

Prepared according to general procedure D, using ethyl (morpholino(oxo)(vinyl)-λ^6-sulfaneylidene)carbamate **3c**. The half-life calculated from two experiments were 3.95 seconds and 4.48 seconds respectively, the average half-life calculated was 4.22 seconds.

HRMS (ESI$^+$), *m/z* calculated for $[C_{19}H_{32}N_5O_{10}S_2D_2]^+$ 558.1867 ($[M-H+2D]^+$), found 558.1866.

Sulfonimidamide-GSH Adduct (13f)

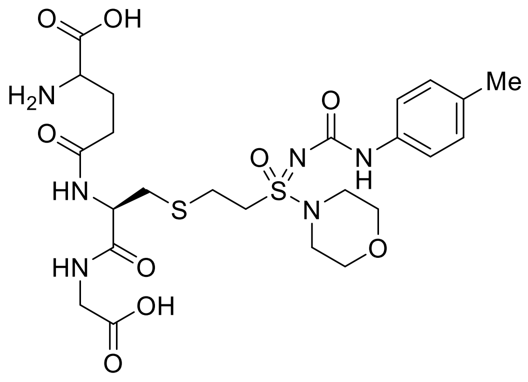

Prepared according to general procedure D, using 1-(morpholino(oxo)(vinyl)-λ^6-sulfaneylidene)-3-(p-tolyl)urea **3d**. The half-life calculated from two experiments were 26.5 seconds and 31.0 seconds respectively, the average half-life calculated was 28.8 seconds.

HRMS (ESI$^+$), *m/z* calculated for $[C_{24}H_{35}N_6O_9S_2D_2]^+$ 619.2184 ($[M-H+2D]^+$), found 619.2191.

Sulfonimidamide-GSH Adduct (13g)

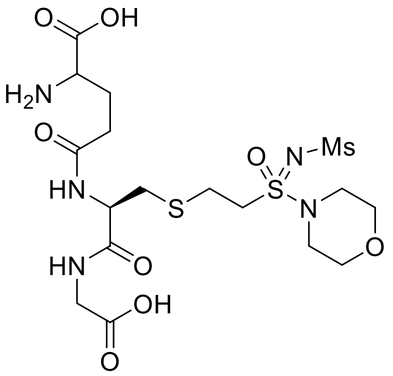

Prepared according to general procedure D, using *N*-(morpholino(oxo)(vinyl)-λ^6-sulfaneylidene)butyramide **3e**. The reaction was too fast to accurately determine the half-life.

HRMS (ESI$^+$), *m/z* calculated for $[C_{17}H_{31}N_5O_{10}S_3D]^+$ 563.1369 ($[M+D]^+$), found 563.1361.

Sulfonimidamide-GSH Adduct (13h)

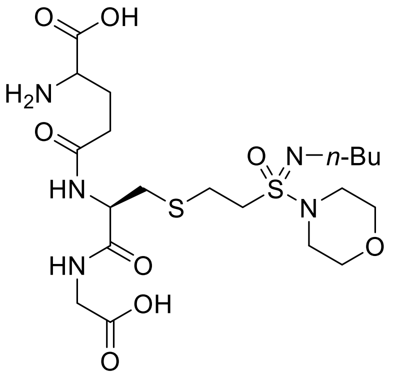

Prepared according to general procedure D, using 4-(*N*-butylvinylsulfonimidoyl)morpholine **3g**. The half-life calculated from three experiments were 167 seconds ,172 seconds and 148 seconds respectively, the average half-life calculated was 162 seconds.

HRMS (ESI$^+$), *m/z* calculated for $[C_{20}H_{38}N_5O_8S_2]^+$ 540.2156 ($[M+H]^+$), found 540.2160.

Sulfonimidamide-GSH Adduct (13i)

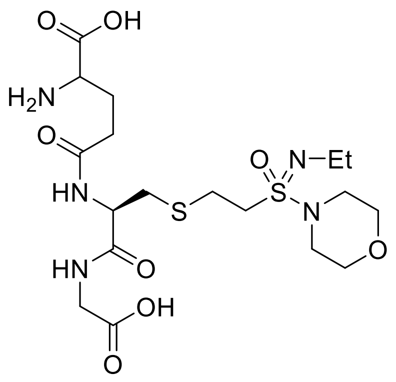

Prepared according to general procedure D, using 4-(*N*-ethylvinylsulfonimidoyl)morpholine **3h**. The half-life calculated from two experiments were 146 seconds and 176 seconds respectively, the average half-life calculated was 161 seconds.

HRMS (ESI$^+$), *m/z* calculated for $[C_{18}H_{32}N_5O_8S_2D_2]^+$ 514.1969 ($[M\text{-}H\text{+}2D]^+$), found 514.1959.

Sulfonimidamide-GSH Adduct (13j)

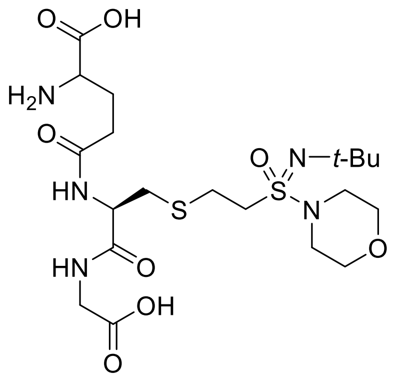

Prepared according to general procedure D, using 4-(*N*-(tert-butyl)vinylsulfonimidoyl)morpholine **3i**. The half-life calculated from two experiments were 415 seconds and 391 seconds respectively, the average half-life calculated was 403 seconds.

HRMS (ESI$^+$), *m/z* calculated for $[C_{20}H_{37}N_5O_8S_2Na]^+$ 562.1976 ($[M\text{+}Na]^+$), found 562.1977.

Sulfonimidamide-GSH Adduct (13k)

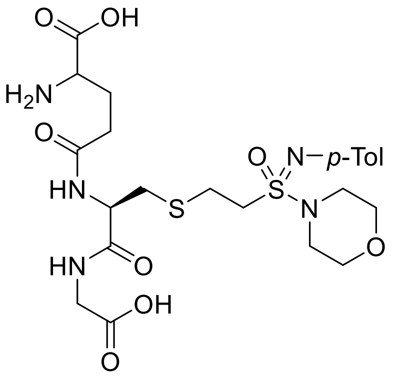

Prepared according to general procedure D, using 4-(*N*-(p-tolyl)vinylsulfonimidoyl)morpholine **3j**. The half-life calculated from two experiments were 501 seconds and 562 seconds respectively, the average half-life calculated was 532 seconds.

HRMS (ESI$^+$), *m/z* calculated for $[C_{23}H_{34}N_5O_8S_2D_2]^+$ 576.2125 ($[M\text{-}H+2D]^+$), found 576.2116.

Sulfonimidamide-GSH Adduct (13l)

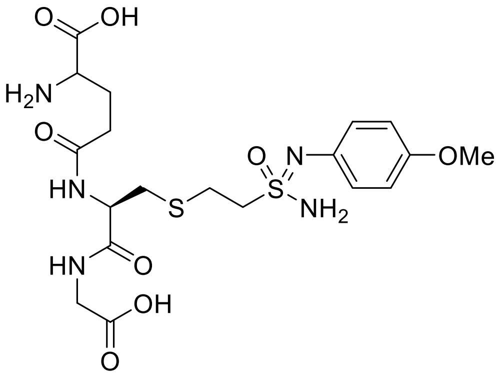

Prepared according to general procedure D, using *N*'-(4-methoxyphenyl)ethenesulfonimidamide **2d**. The half-life calculated from two experiments were 1006 seconds and 1123 seconds respectively, the average half-life calculated was 1065 seconds.

HRMS (ESI$^+$), *m/z* calculated for $[C_{19}H_{29}N_5O_8S_2D]^+$ 521.1593 ($[M+D]^+$), found 521.1600.

Sulfonimidamide-GSH Adduct (13m)

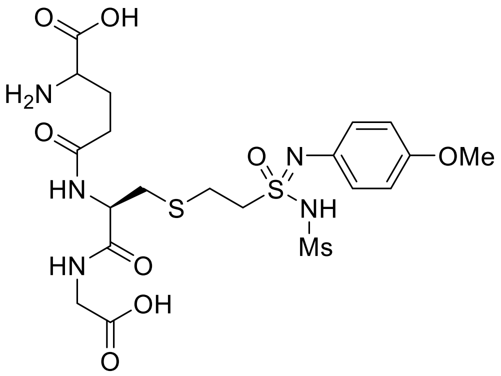

Prepared according to general procedure D, using *N*-(*N*-(4-methoxyphenyl)vinylsulfonimidoyl)methanesulfonamide **6a**. The half-life calculated from two experiments were 284 seconds and 264 seconds respectively, the average half-life calculated was 274 seconds.

HRMS (ESI$^+$), *m/z* calculated for $[C_{20}H_{31}N_5O_{10}S_3D]^+$ 599.1369 ($[M+D]^+$), found 599.1369.

Sulfonimidamide-GSH Adduct (13n)

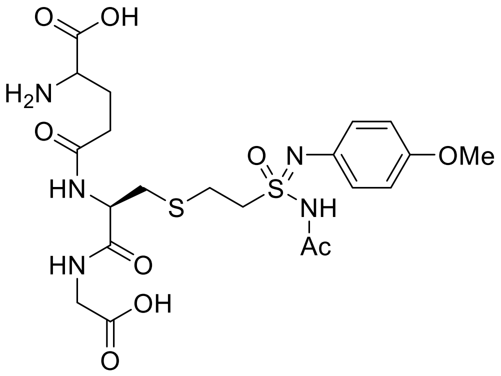

Prepared according to general procedure D, using *N*-(*N*-(4-methoxyphenyl)vinylsulfonimidoyl)acetamide **6b**. The half-life calculated from two experiments were 72.7 seconds and 90.7 seconds respectively, the average half-life calculated was 81.7 seconds.

HRMS (ESI$^+$), *m/z* calculated for $[C_{21}H_{31}N_5O_9S_2D]^+$ 563.1699 ($[M+D]^+$), found 563.1717.

Sulfonimidamide-GSH Adduct (13o)

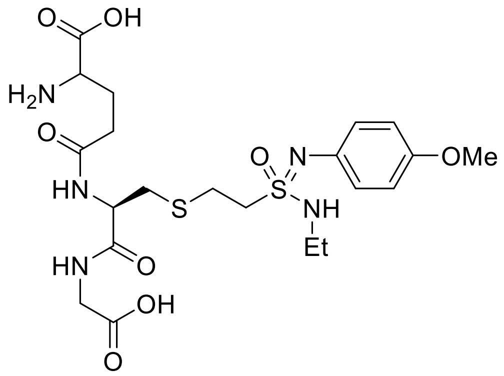

Prepared according to general procedure D, using *N*-ethyl-*N*'-(4-methoxyphenyl)ethenesulfonimidamide **6d**. The half-life calculated from two experiments were 827 seconds and 860 seconds respectively, the average half-life calculated was 842 seconds.

HRMS (ESI$^+$), *m/z* calculated for $[C_{21}H_{33}N_5O_8S_2D]^+$ 549.1906 ($[M+D]^+$), found 549.1913.

Sulfonimidamide-GSH Adduct (13p)

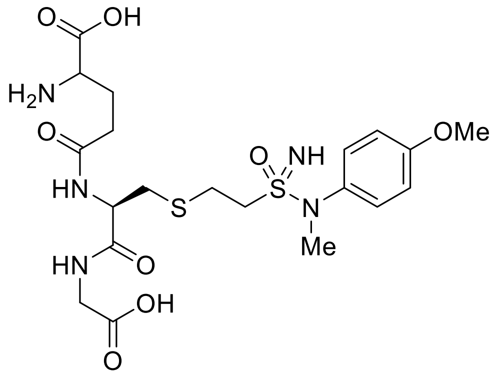

Prepared according to general procedure D, using *N*-(4-methoxyphenyl)-*N*-methylethenesulfonimidamide **2e**. Decomposition was observed when the substrate **2e** was added into the buffer-DMSO mixture thus the half-life of the conjugate addition could not be determined.

HRMS (ESI$^+$) not found.

Sulfonimidamide-GSH Adduct (13q)

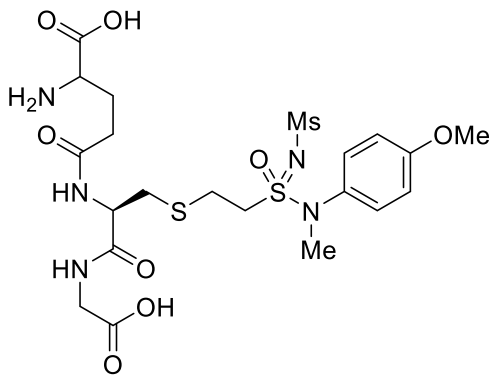

Prepared according to general procedure D, using *N*-(((4-methoxyphenyl)(methyl)amino)(oxo)(vinyl)-λ^6-sulfaneylidene)methanesulfonamide **5a**. The reaction was too fast to accurately determine the half-life.

HRMS (ESI$^+$), *m/z* calculated for $[C_{21}H_{31}N_5O_{10}S_3D_2K]^+$ 652.1147 ($[M-2H+2D+K]^+$, found 652.1146.

Sulfonimidamide-GSH Adduct (13r)

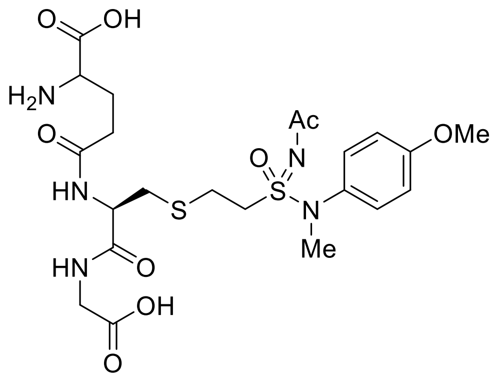

Prepared according to general procedure D, using *N*-(((4-methoxyphenyl)(methyl)amino)(oxo)(vinyl)-λ^6-sulfaneylidene)acetamide **5b**. The half-life calculated from two experiments were 3.46 seconds and 2.93 seconds respectively, the average half-life calculated was 3.20 seconds.

HRMS (ESI$^+$), *m/z* calculated for $[C_{22}H_{34}N_5O_9S_2]^+$ 576.1793 ($[M+H]^+$), found 576.1797.

Sulfonimidamide-GSH Adduct (13s)

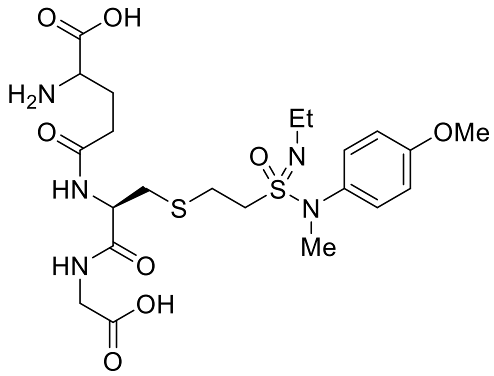

Prepared according to general procedure D, using *N'*-ethyl-*N*-(4-methoxyphenyl)-*N*-methylethenesulfonimidamide **5c**. The half-life calculated from two experiments were 192 seconds and 200 seconds respectively, the average half-life calculated was 196 seconds.

HRMS (ESI$^+$), *m/z* calculated for $[C_{22}H_{33}N_5O_8S_2D_2Na]^+$ 586.1945 ($[M-H+2D+Na]^+$), found 586.1942.

Sulfonimidamide-GSH Adduct (13t)

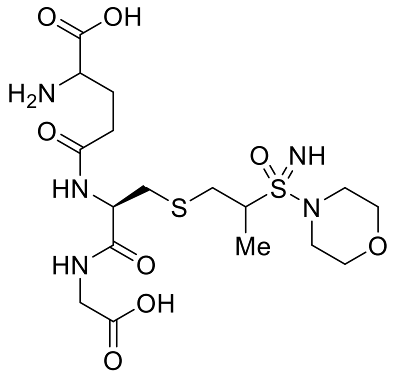

Prepared according to general procedure D, using 4-(prop-1-en-2-ylsulfonimidoyl)morpholine **2f** except that 6 equiv. of glutathione was used, and the half-life was determined using pseudo-first-order kinetics. The half-life calculated from two experiments were 33095 seconds and 38650 seconds respectively, the average half-life calculated was 35873 seconds.

HRMS (ESI$^+$), *m/z* calculated for $[C_{17}H_{31}N_5O_8S_2D]^+$ 499.1750 ($[M+D]^+$), found 499.1741.

Sulfonimidamide-GSH Adduct (13u)

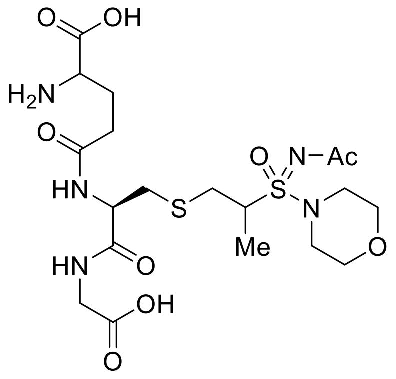

Prepared according to general procedure D, using *N*-(morpholino(oxo)(prop-1-en-2-yl)-λ^6-sulfaneylidene)acetamide **4a**. The half-life calculated from two experiments were 4738 seconds and 4621 seconds respectively, the average half-life calculated was 4680 seconds.

HRMS (ESI$^+$), *m/z* calculated for $[C_{19}H_{33}N_5O_9S_2D]^+$ 541.1855 ($[M+D]^+$), found 541.1853.

Sulfonimidamide-GSH Adduct (13v)

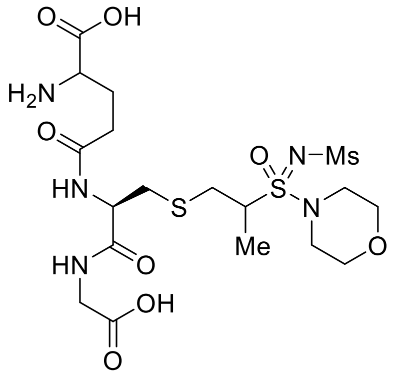

Prepared according to general procedure D, using *N*-(morpholino(oxo)(prop-1-en-2-yl)-λ^6-sulfaneylidene)methanesulfonamide **4b**. The half-life calculated from two experiments were 230 seconds and 268 seconds respectively, the average half-life calculated was 249 seconds.

HRMS (ESI$^+$), *m/z* calculated for $[C_{18}H_{34}N_5O_{10}S_3]^+$ 576.1462 ($[M+H]^+$), found 576.1463.

Sulfonimidamide-GSH Adduct (13w)

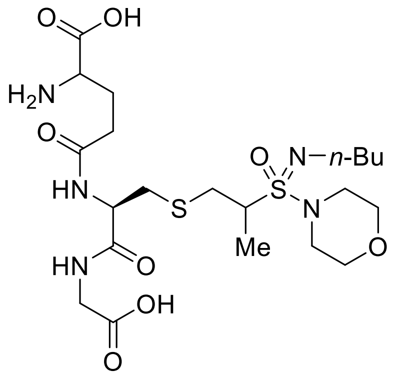

Prepared according to general procedure D, using 4-(*N*-butylprop-1-en-2-ylsulfonimidoyl)morpholine **4c**. The half-life calculated from two experiments were 11143 seconds and 10902 seconds respectively, the average half-life calculated was 11023 seconds.

HRMS (ESI$^+$), *m/z* calculated for $[C_{21}H_{40}N_5O_8S_2]^+$ 554.2313 ($[M+H]^+$), found 554.2318.

Sulfonimidamide-GSH Adduct (13x)

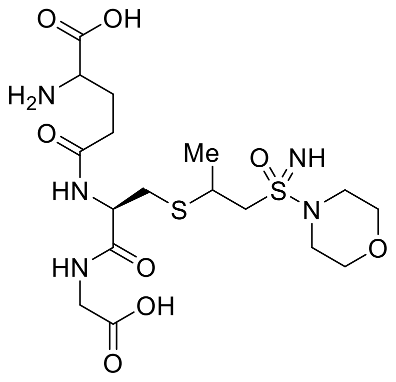

Prepared according to general procedure D, using (*E*)-4-(prop-1-en-1-ylsulfonimidoyl)morpholine **trans-2h** except that 6 equiv. of glutathione was used, and the half-life was determined using pseudo-first-order kinetics. The half-life calculated from two experiments were 16989 seconds and 15768 seconds respectively, the average half-life calculated was 16379 seconds.

HRMS (ESI$^+$), *m/z* calculated for $[C_{17}H_{31}N_5O_8S_2D]^+$ 499.1750 ($[M+D]^+$), found 499.1754.

Sulfonimidamide-GSH Adduct (13y)

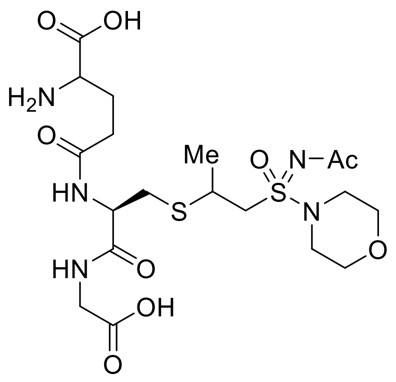

Prepared according to general procedure D, using (*E*)-*N*-(morpholino(oxo)(prop-1-en-1-yl)-λ^6-sulfaneylidene)acetamide **4d**. The half-life calculated from two experiments were 1450 seconds and 1647 seconds respectively, the average half-life calculated was 1549 seconds.

HRMS (ESI$^+$), *m/z* calculated for $[C_{19}H_{33}N_5O_9S_2D]^+$ 541.1855 ($[M+D]^+$), found 541.1862.

Sulfonimidamide-GSH Adduct (13z)

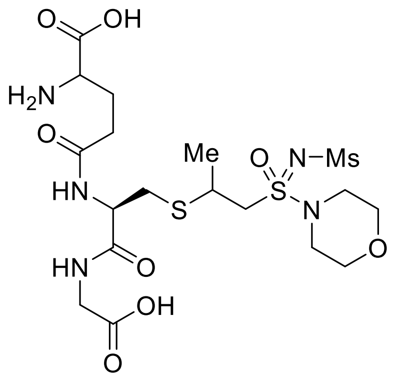

Prepared according to general procedure D, using (*E*)-*N*-(morpholino(oxo)(prop-1-en-1-yl)-λ^6-sulfaneylidene)methanesulfonamide **4e**. The half-life calculated from two experiments were 221 seconds and 215 seconds respectively, the average half-life calculated was 218 seconds.

HRMS (ESI$^+$), *m/z* calculated for $[C_{18}H_{32}N_5O_{10}S_3DNa]^+$ 599.1345 ($[M+D+Na]^+$), found 599.1347.

Sulfonimidamide-GSH Adduct (13aa)

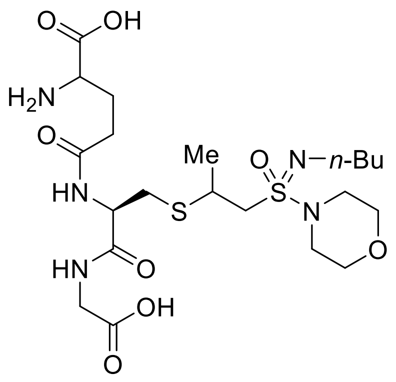

Prepared according to general procedure D, using (*E*)-4-(*N*-butylprop-1-en-1-ylsulfonimidoyl)morpholine **4f**. The half-life calculated from two experiments were 9016 seconds and 9598 seconds respectively, the average half-life calculated was 9307 seconds.

HRMS (ESI^+), *m/z* calculated for $[C_{21}H_{37}N_5O_8S_2D_2Na]^+$ 578.2258 ($[M\text{-}H+2D+Na]^+$), found 578.2257.

Sulfonamide-GSH Adduct (13ab)

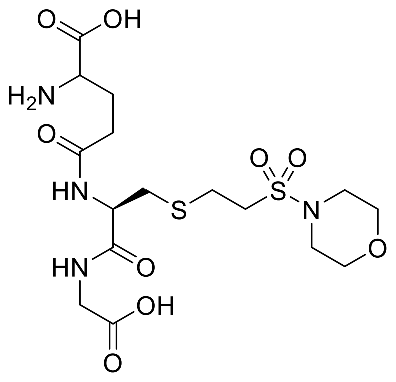

Prepared according to general procedure D, using 4-(vinylsulfonyl)morpholine **12**. The half-life calculated from two experiments were 226 second and 237 second respectively, the average half-life calculated was 232 seconds.

HRMS (ESI^+), *m/z* calculated for $[C_{16}H_{29}N_4O_9S_2]^+$ 485.1371 ($[M+H]^+$), found 485.1369.

3. References

1 B. E. Love, E. G. Jones. The use of Salicylaldehyde Phenylhydrazone as an Indicator for the Titration of Organometallic Reagents. *J. Org. Chem.* 1999, **64**, 10, 3755-3756.

2 Pd-Catalyzed O-Arylation of Ethyl Acetohydroximate: Synthesis of *O*-Arylhydroxylamines and Substituted Benzofurans. *J. Am. Chem. Soc.* 2010, **132**, 9990-9991.

3 T. Q. Davies, M. J. Tilby, J. Ren, N. A. Parker, D. Skolc, A. Hall, F. Duarte, M. C. Willis. Harnessing Sulfinyl Nitrenes: A Unified One-Pot Synthesis of Sulfoximines and Sulfonimidamides. *J. Am. Chem. Soc*. 2020, **142**, 15445-15453.

4 Y. H. Kim, J. M. Shin. New facile synthesis of N-sulfinylamine derivatives using N.N'-sulfinylbisimidazole and N-(chlorosulfinyl)imidazole. *Tetrahedron Lett*. 1985, **26**, 3821-3824.

5 Ł. Woźniak, A. A. Rajkiewicz, L. Monsigny, A. Kajetanowicz, K. Grela. Preparation of Functionalized α,β-unsaturated Sulfonamides via Olefin Cross-Metathesis. *Org. Lett.* 2020, **22**, 4970-4973.

6 F. Izzo, M. Schäfer, P. Lienau, U. Ganzer, R. Stockman, U. Lücking. *Chem. Eur. J*. 2018., **24**, 9295-9304

7 J. C. Vantourout, L. Li, E. B. Moll, S. Chabbra, K. Arrington, B. E. Bode, A. I. Llobet, J. A. Kowalski, M. G. Nilson, K. M. P. Wheelhouse, J. L. Woodard, S. Xie, D. Leitch, A. J. B. Watson. *ACS Catal*. 2018, **8**, 9560–9566

8 C.H. Wu, J. H. Sheu, C. Y. Chen, Y. C. Chien, S. C. Chen, C. H. Pan, C. Y. Huang. Pharmaceutical Uses Of Sulfur-Containing Compound. *USPTO*, US2016009642A1, 2016.

9 H. Chen, R. Huang, Z. Li, W. Zhu, J. Chen, Y. Zhan, B. Jiang. Selective lysine modification of native peptides via aza-Michael addition. *Org. Biomol. Chem*., 2017, **15**, 7339-7345.

10 A. Krężel, W. Bal. A formula for correlating pKa values determined in D2O and H2O. *J. Inorg. Biochem*, 2004, **98**, 161-166.

4. NMR spectra

O-([1,1'-biphenyl]-4-yl)hydroxylamine (SI-2), BiPhONH2

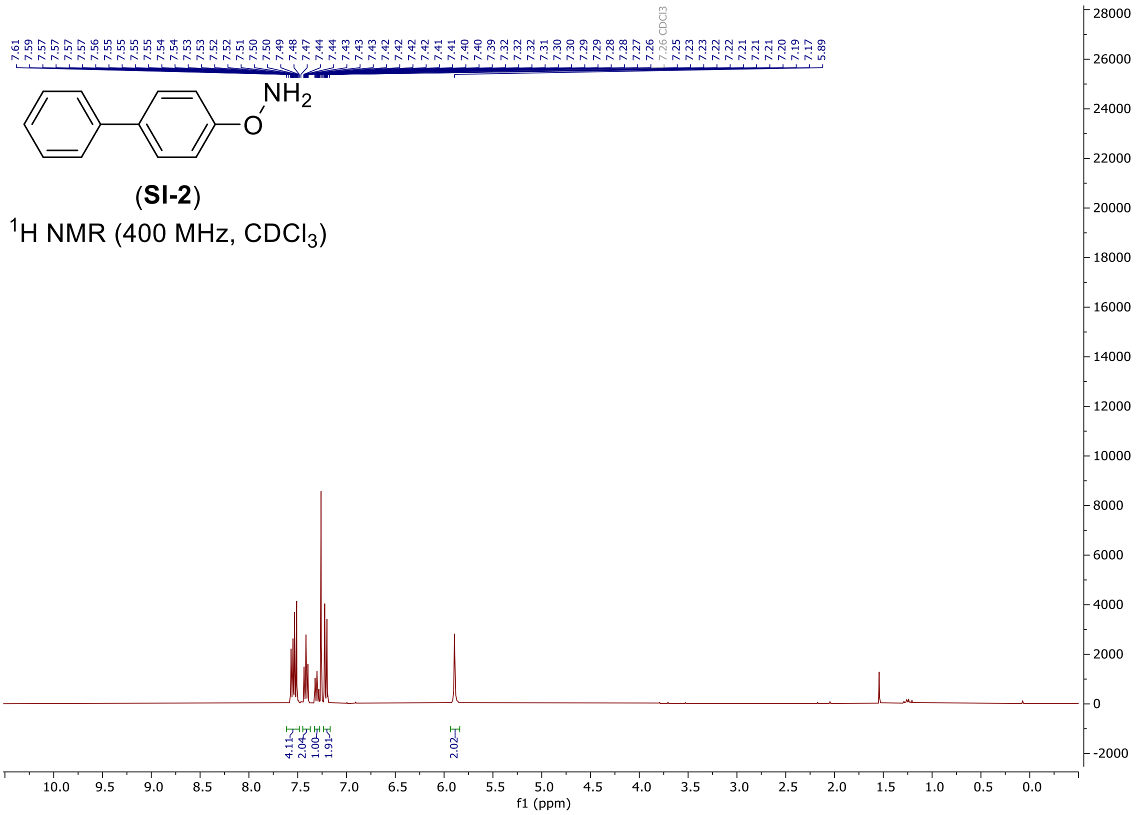

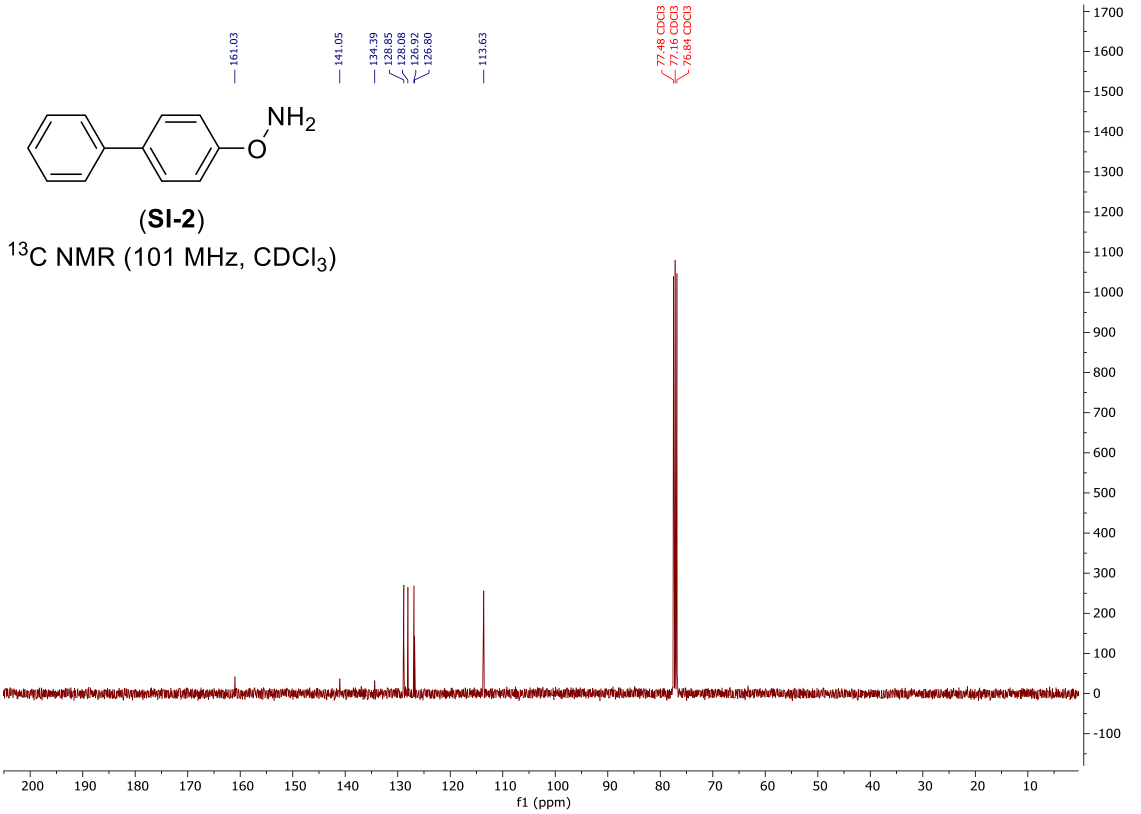

(([1,1'-Biphenyl]-4-yloxy)imino)-lλ⁴-sulfanone, BiPhONSO (1)

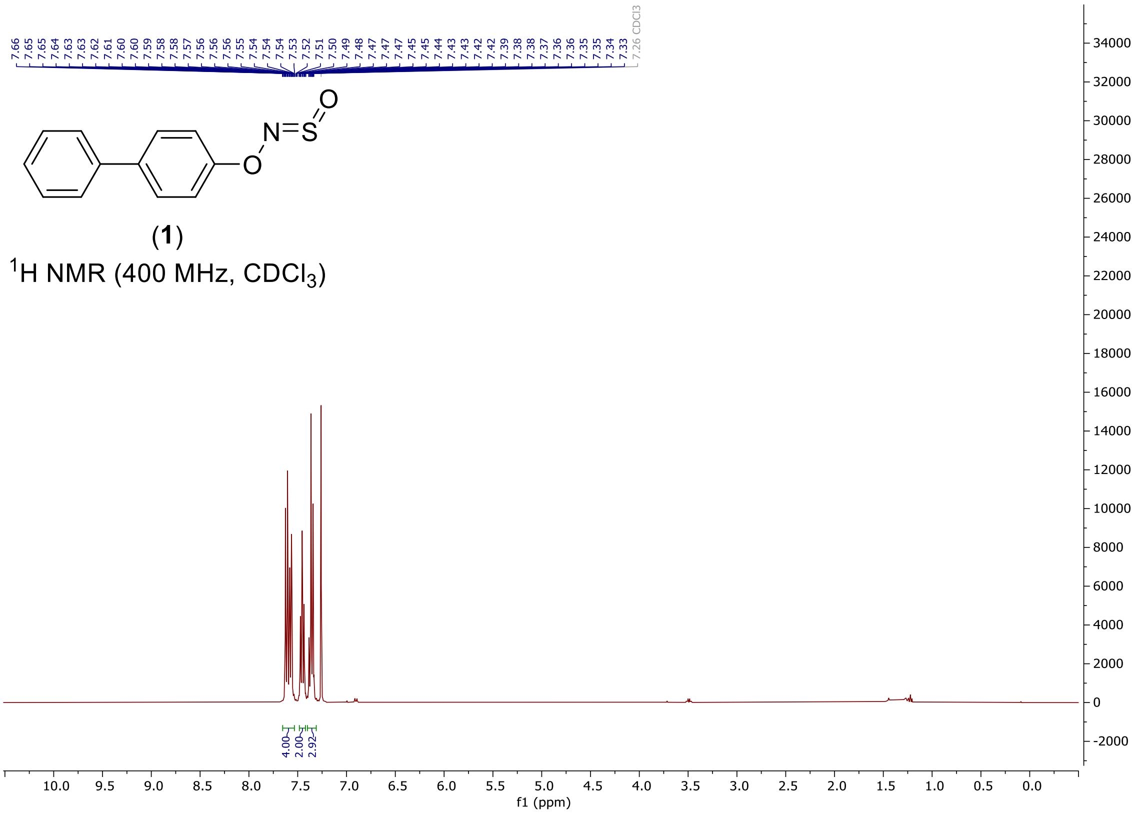

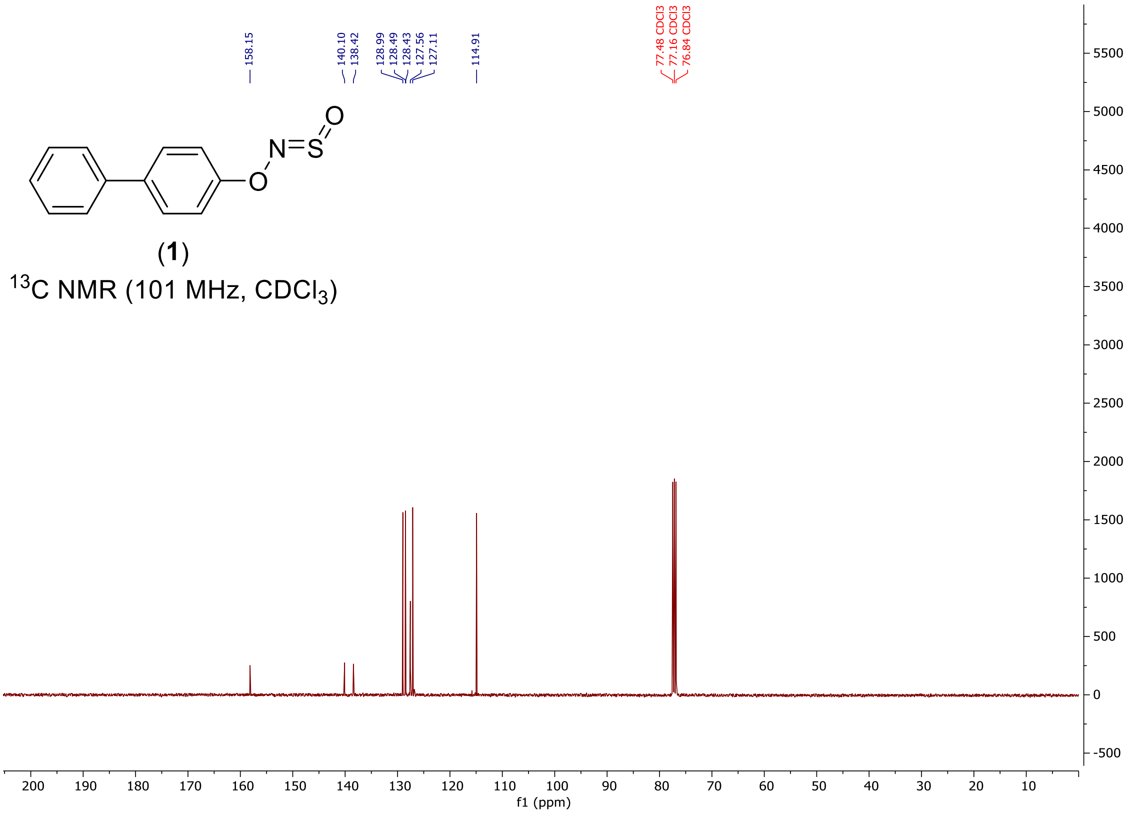

(*tert*-Butylimino)-λ⁴-sulfanone(SI-3)

Tert-butyl N-sulfinylamine

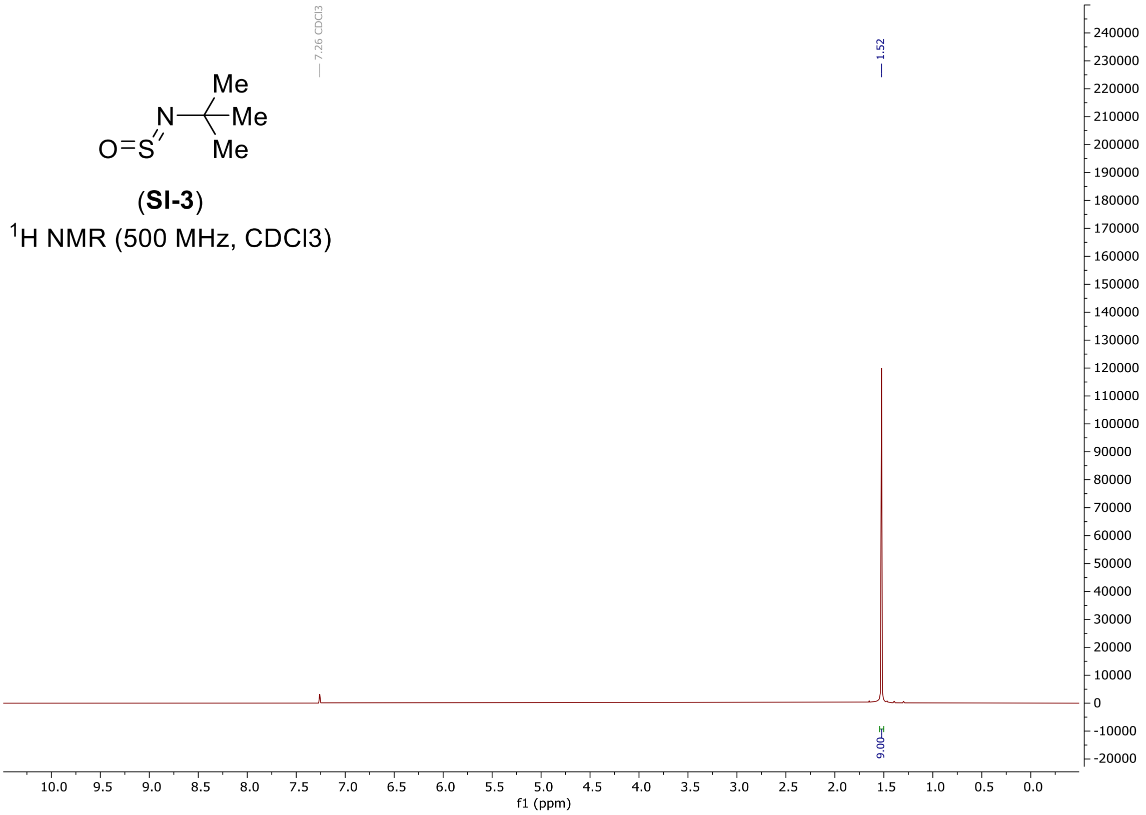

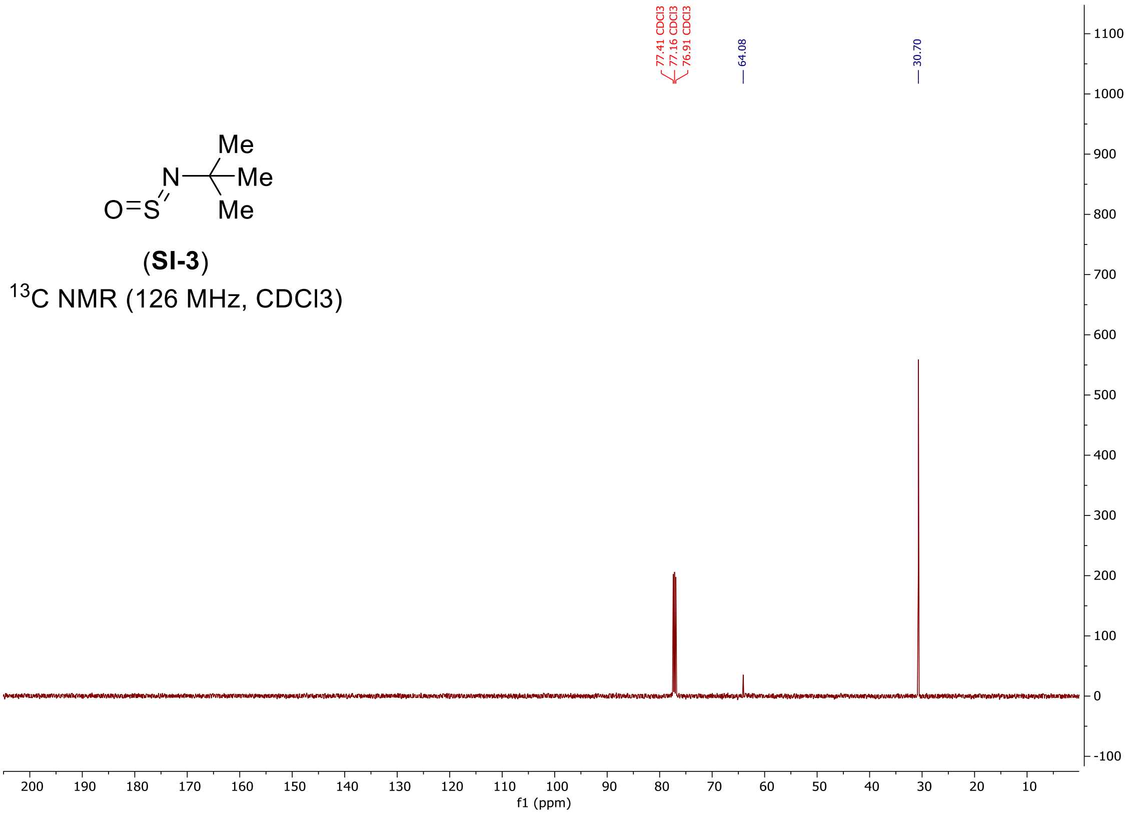

4-(Vinylsulfonimidoyl)morpholine (2a)

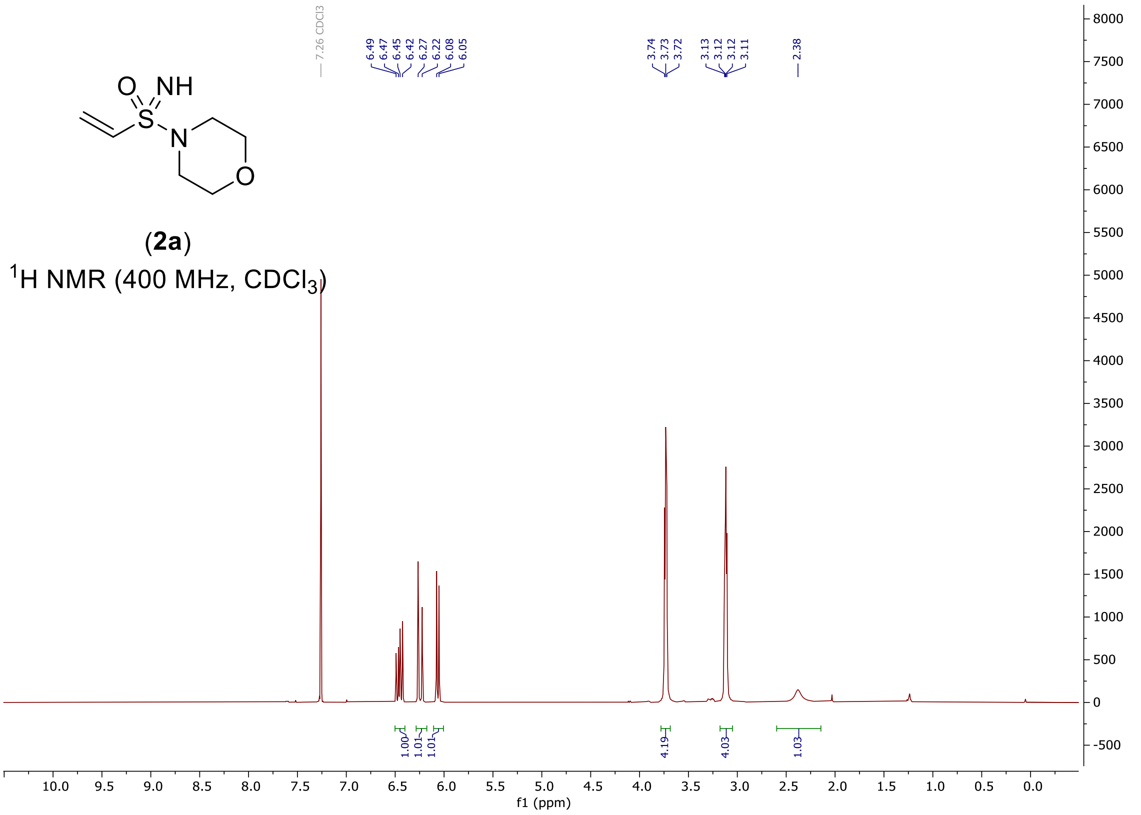

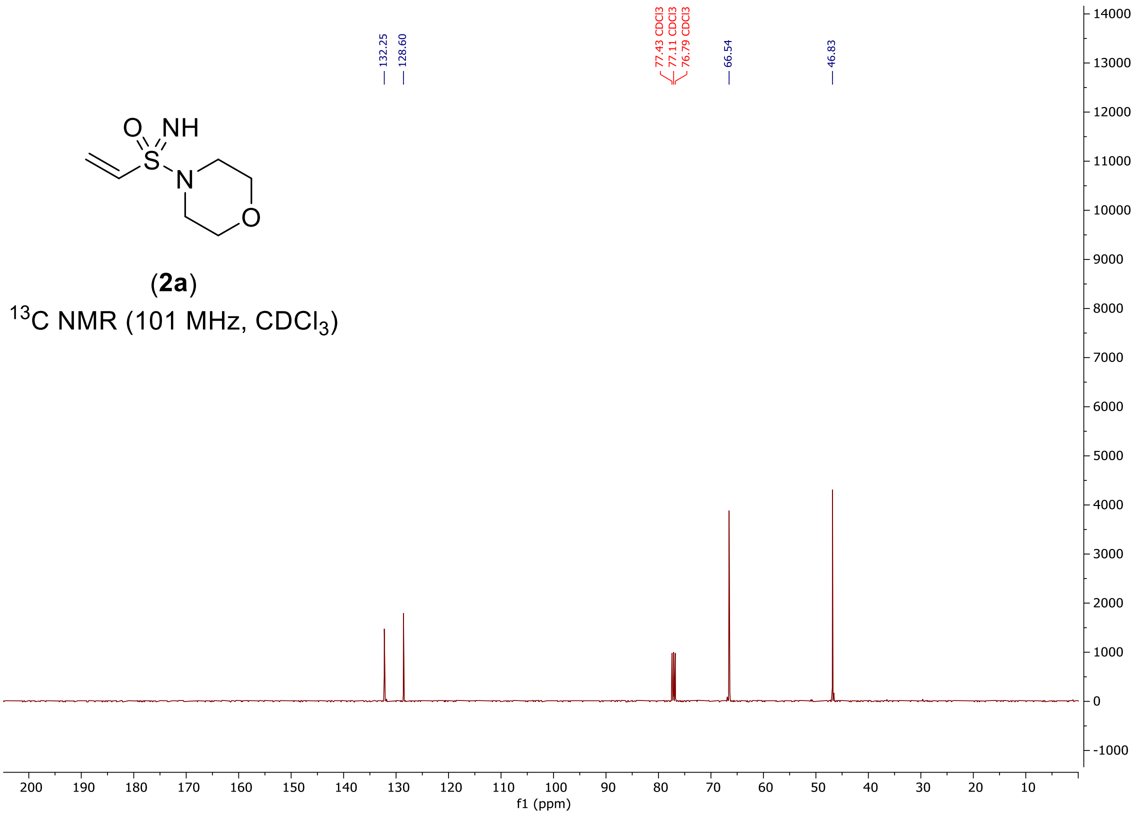

***N*-(4-Fluorophenyl)ethenesulfonimidamide (2b)**

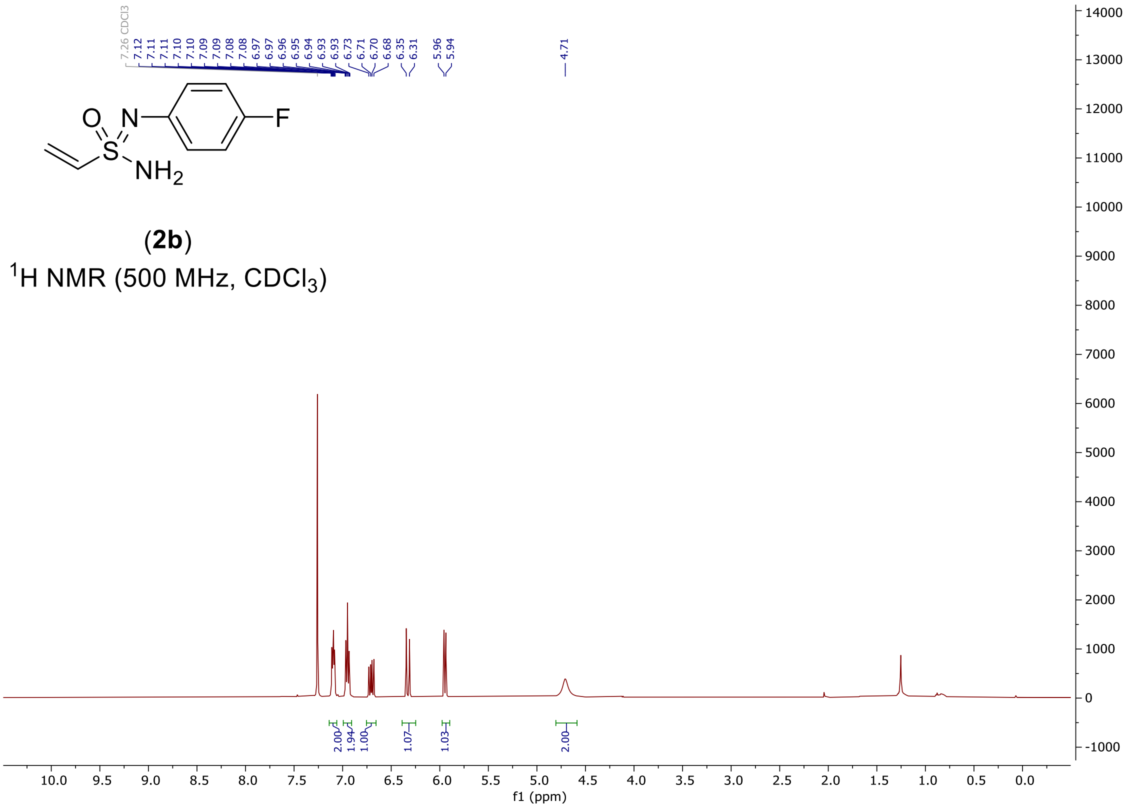

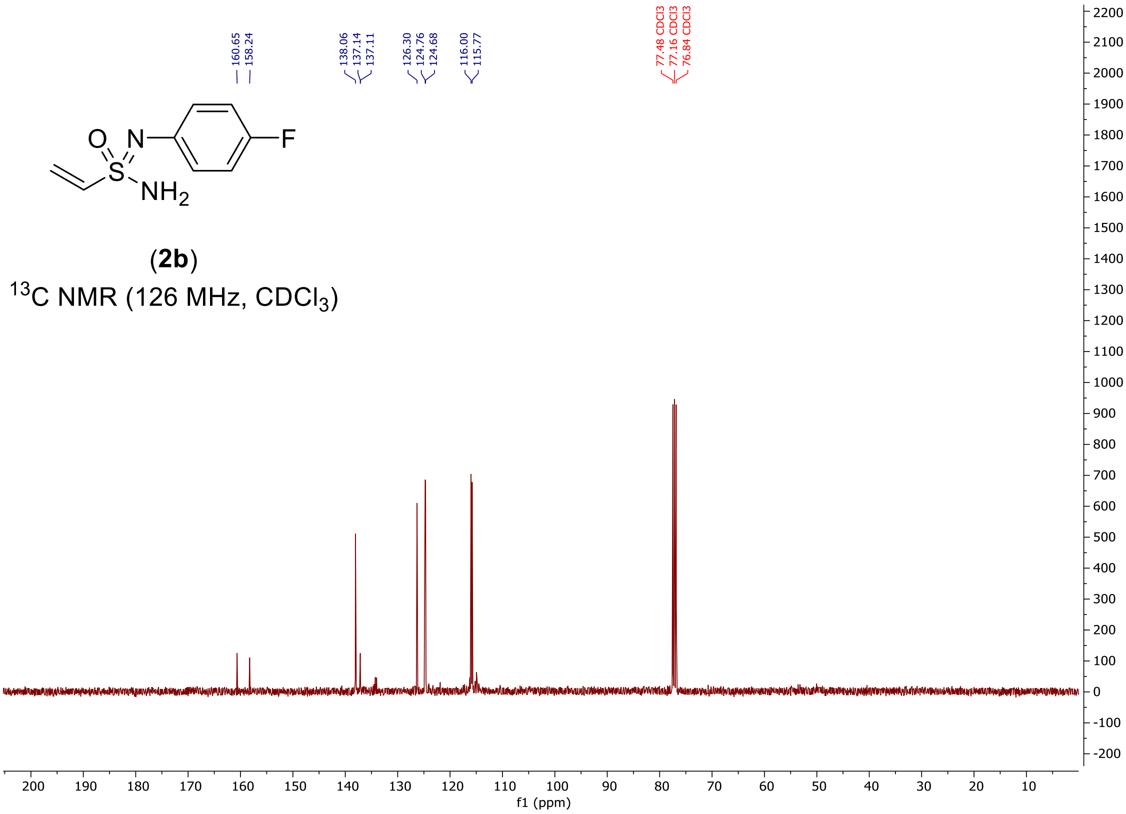

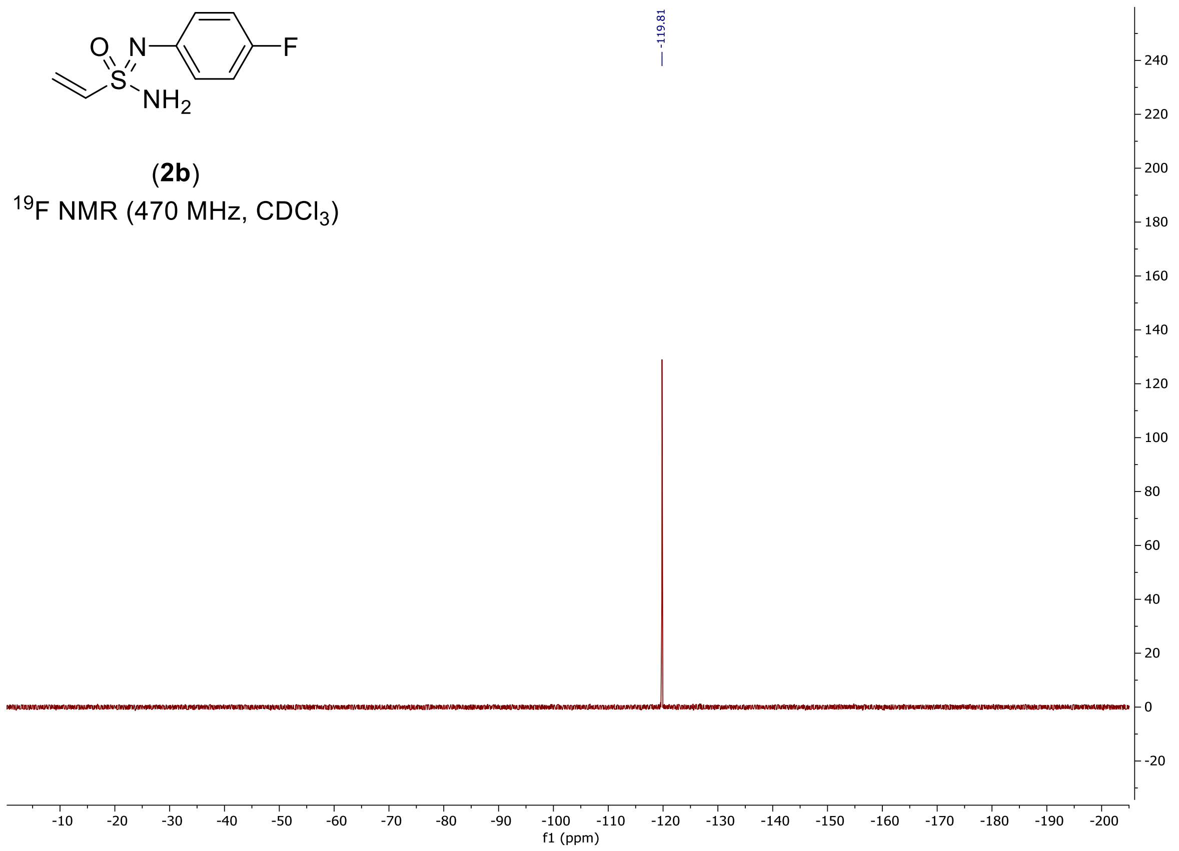

N'-(4-Ethynylphenyl)ethenesulfonimidamide (2c)

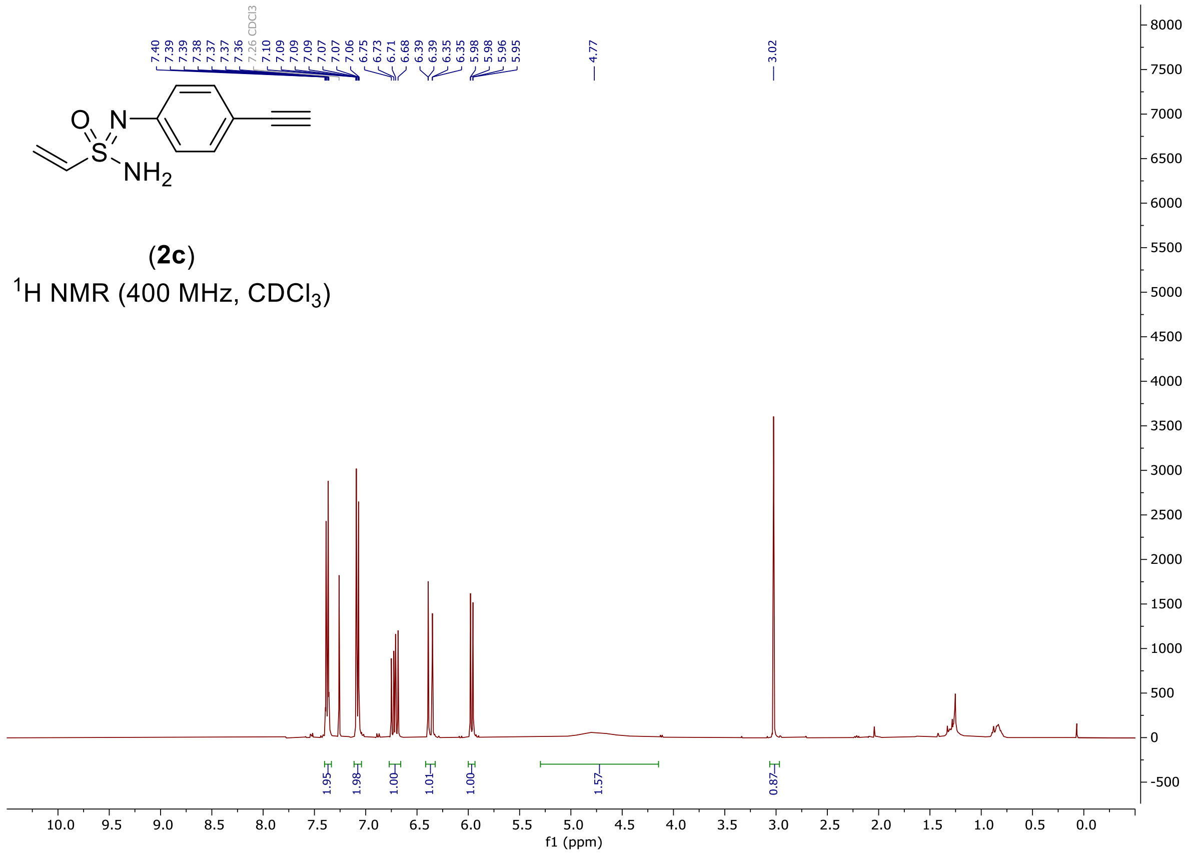

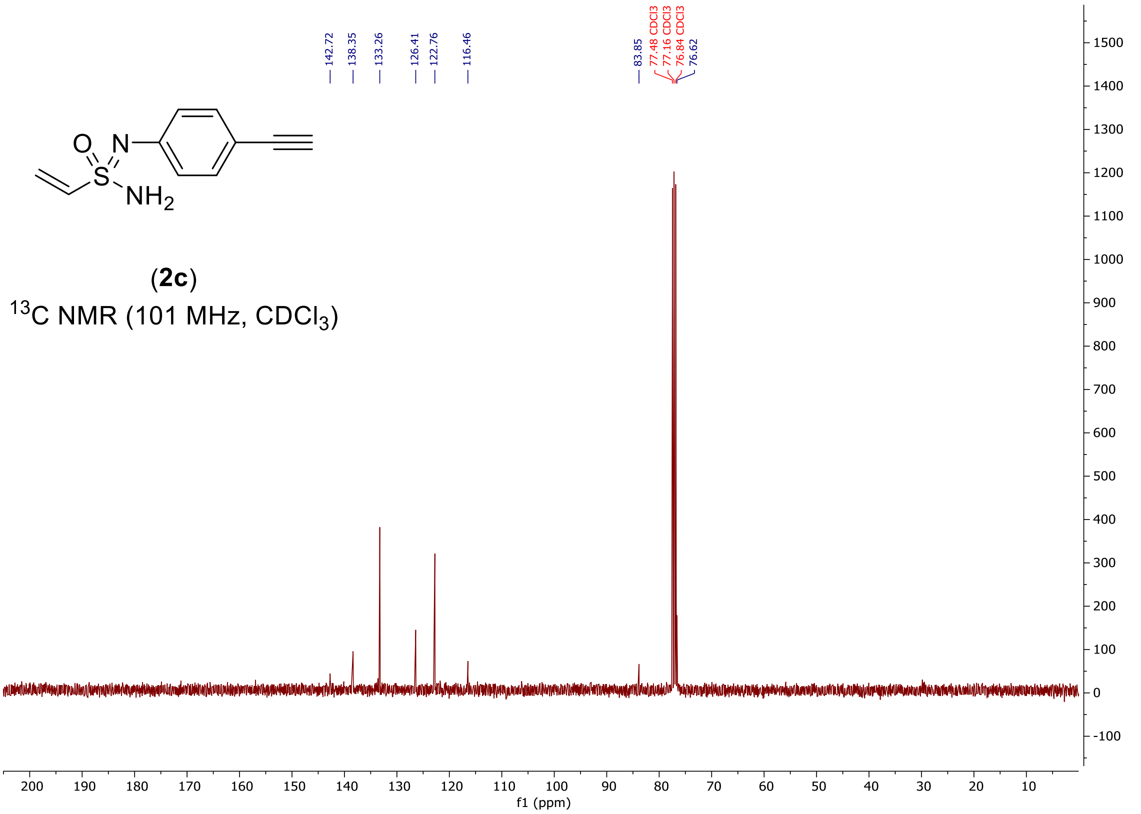

N'-(4-Methoxyphenyl)ethenesulfonimidamide (2d)

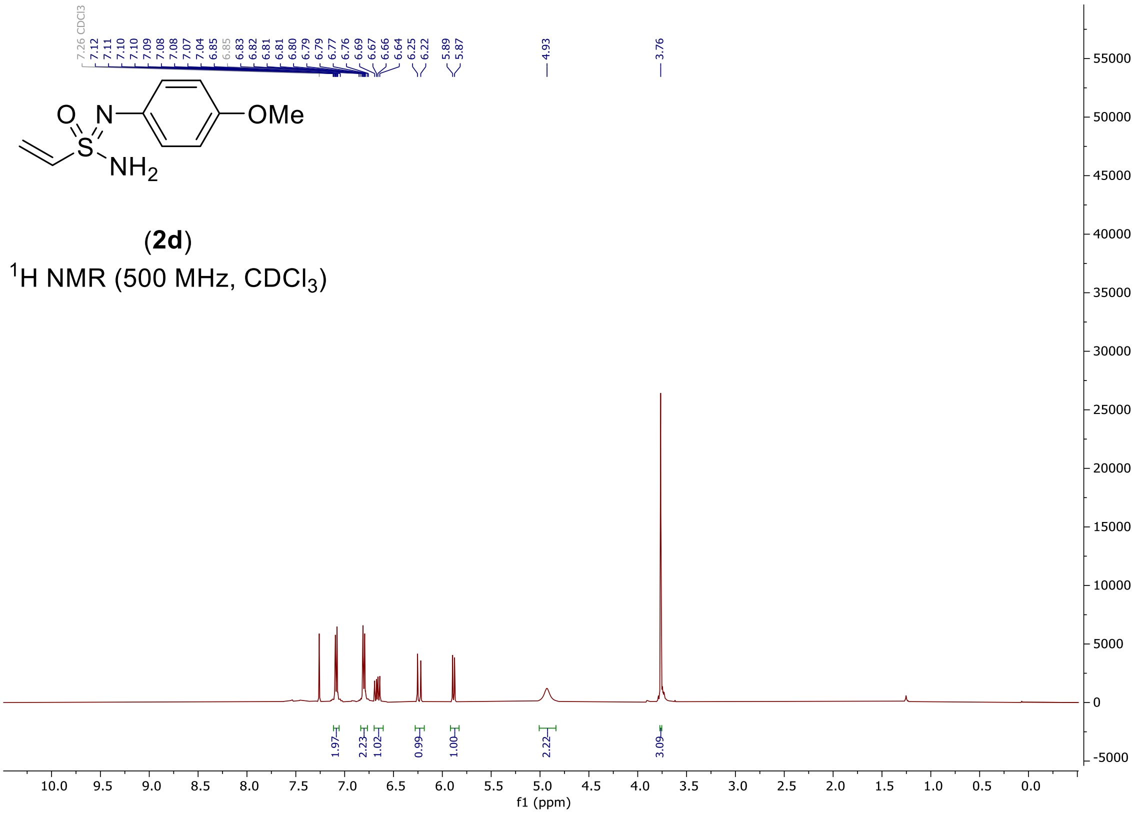

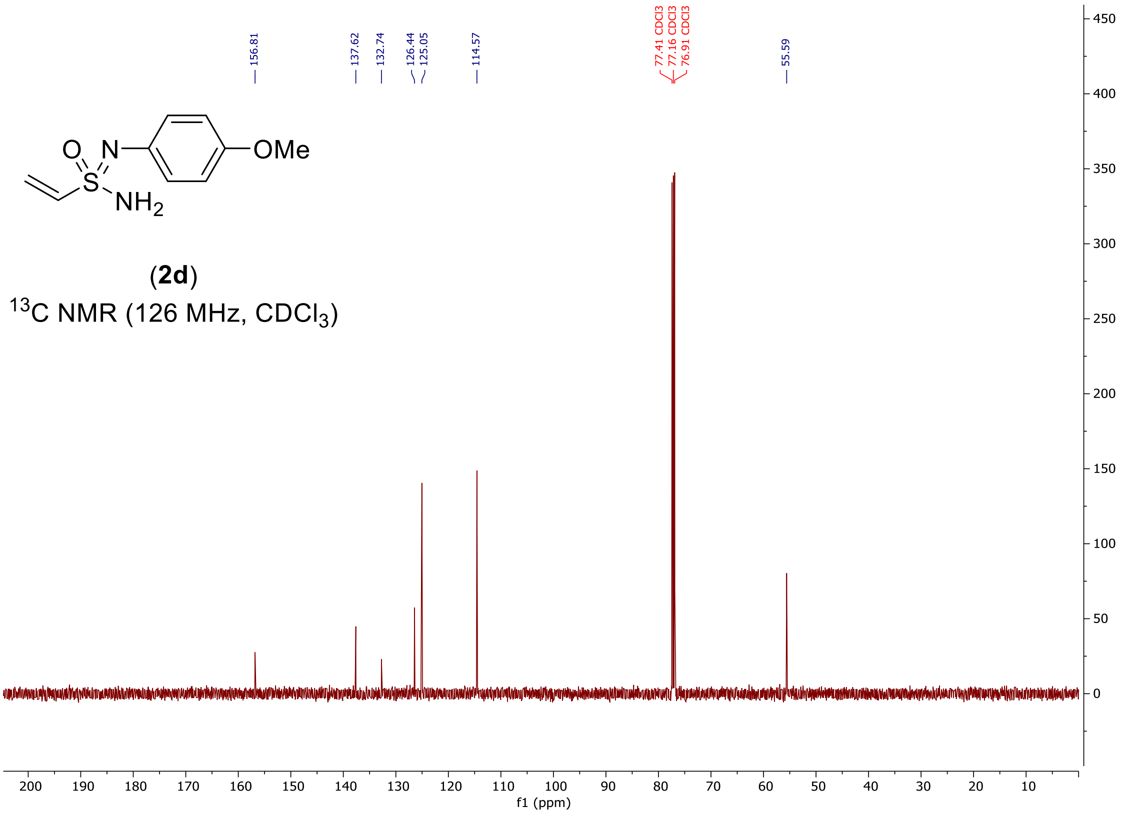

N-(4-Methoxyphenyl)-*N*-methylethenesulfonimidamide (2e)

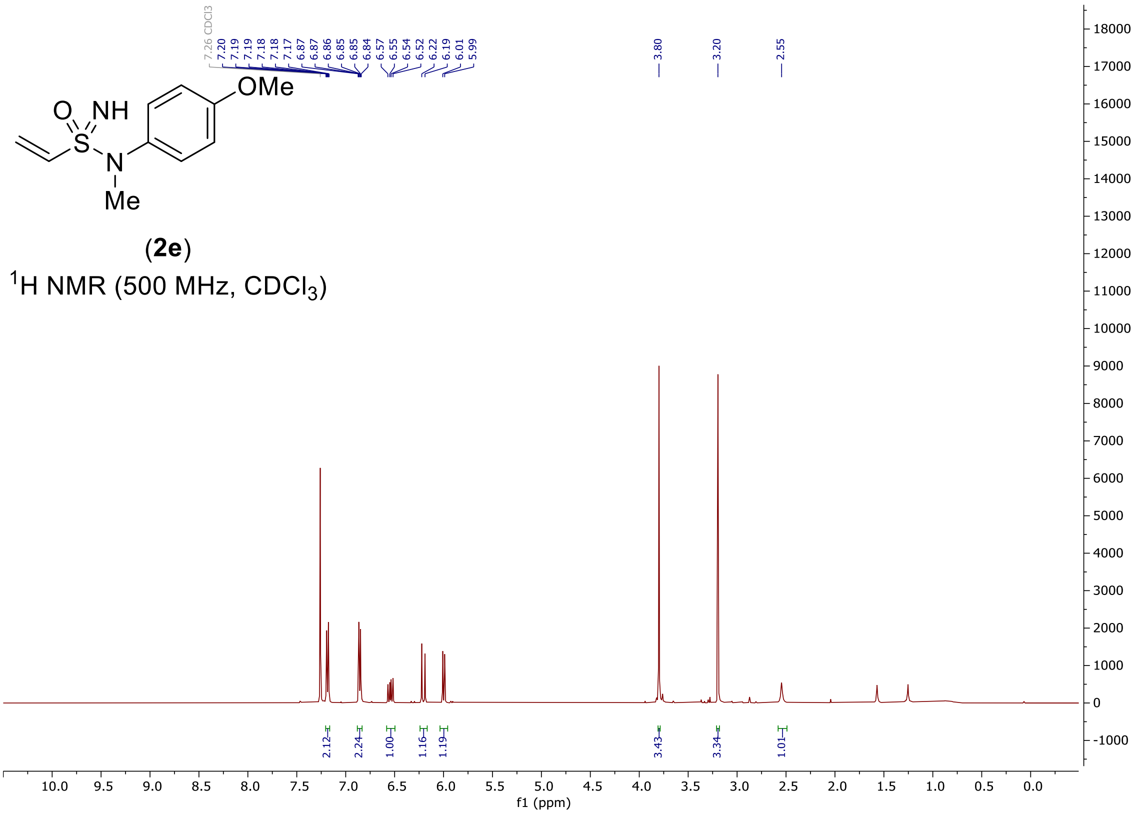

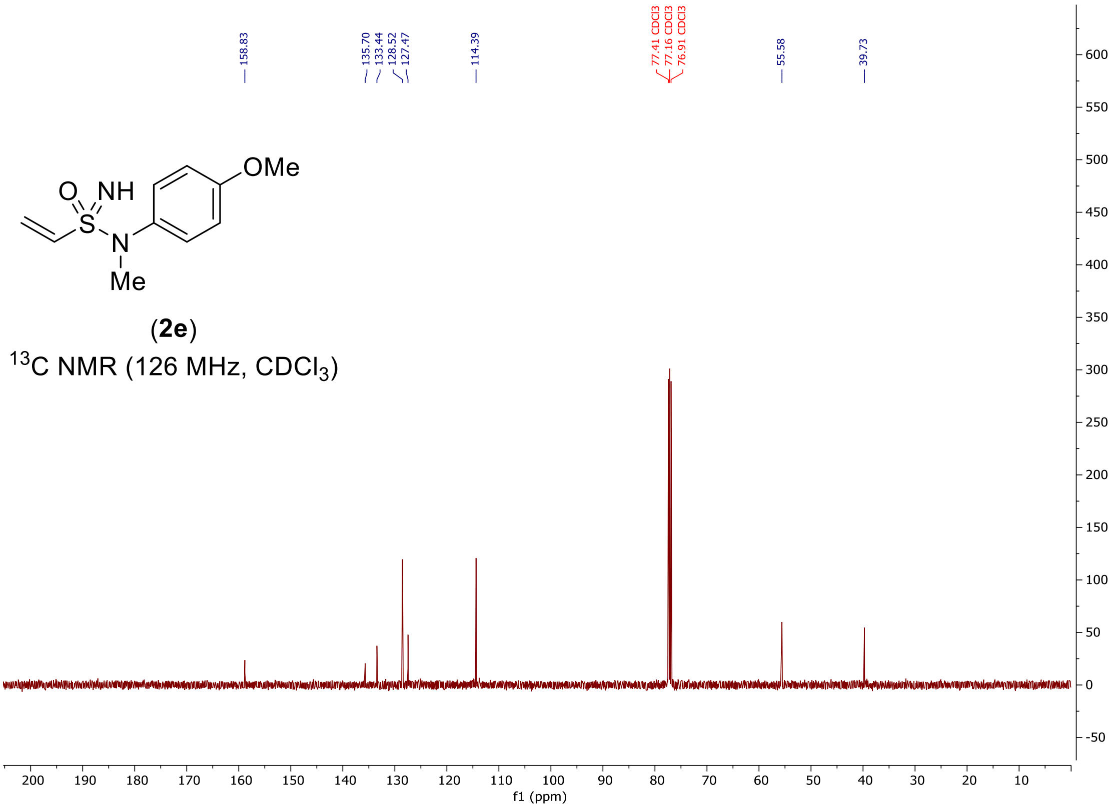

4-(Prop-1-en-2-ylsulfonimidoyl)morpholine (2f)

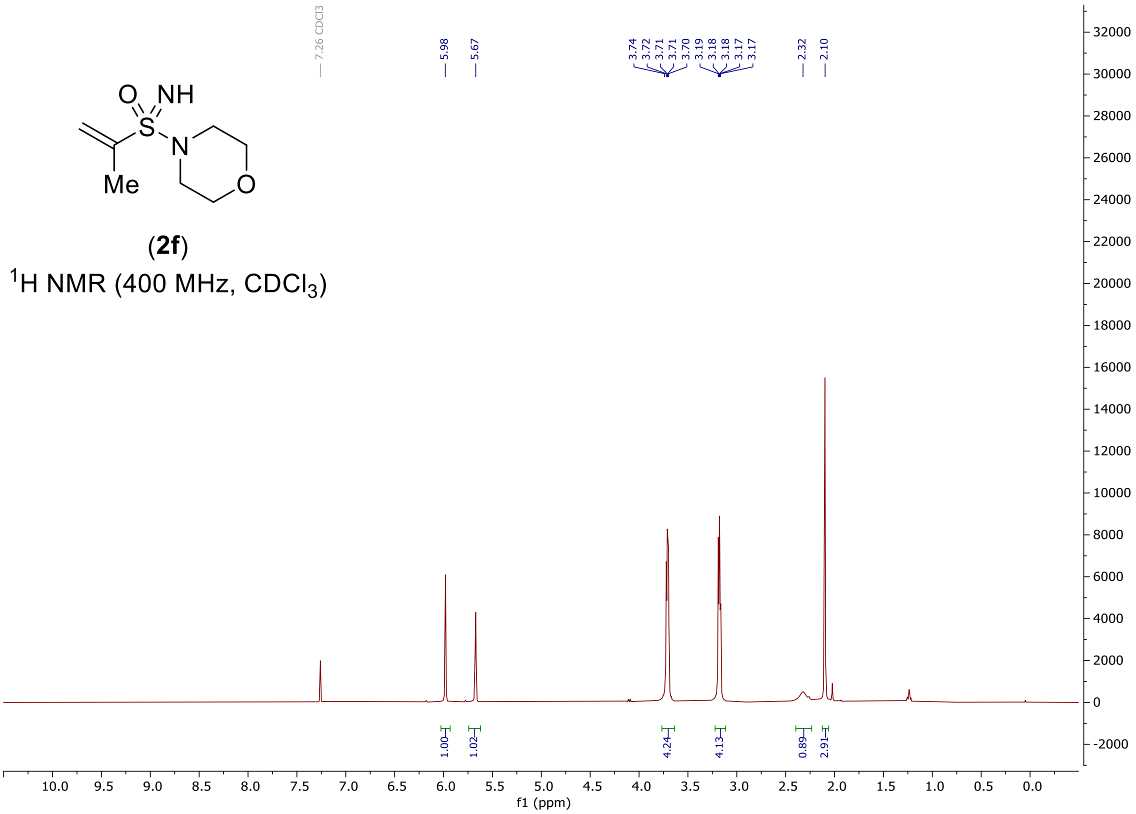

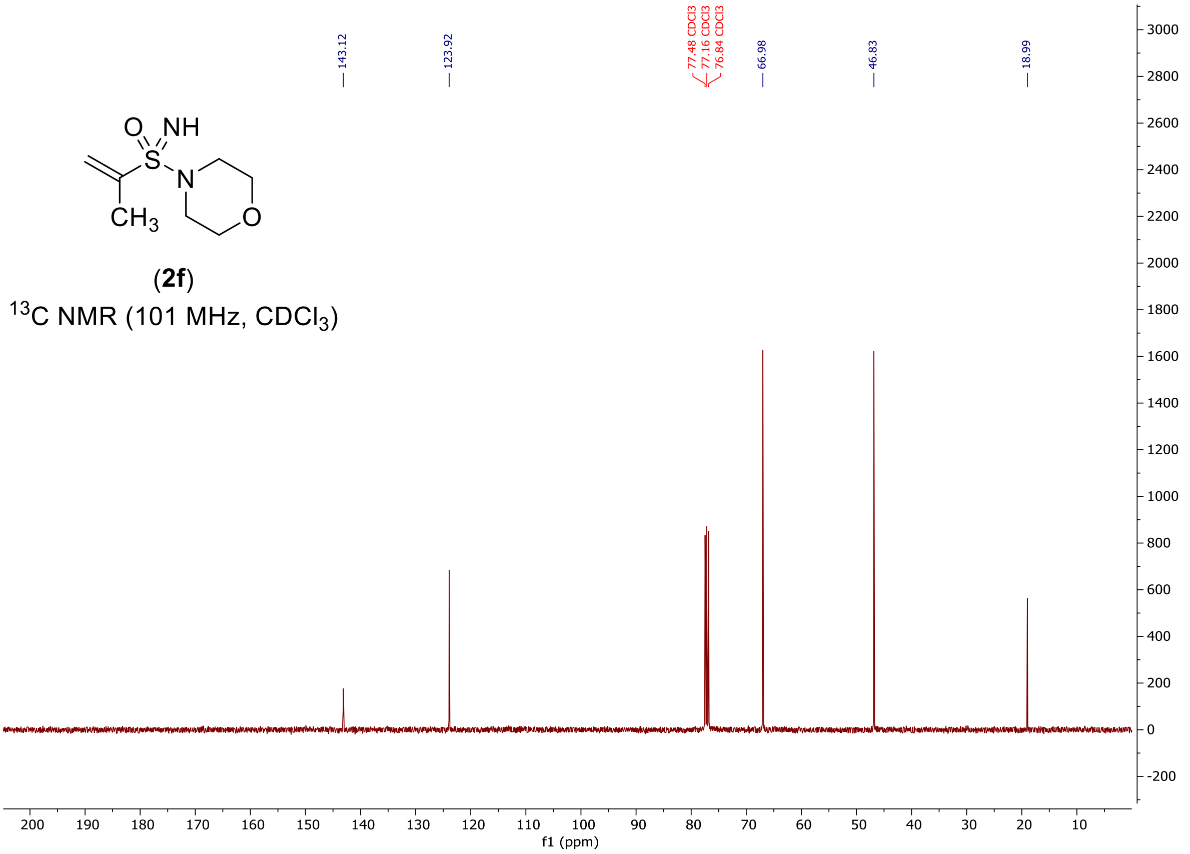

N'-(4-Methoxyphenyl)prop-1-ene-2-sulfonimidamide (2g)

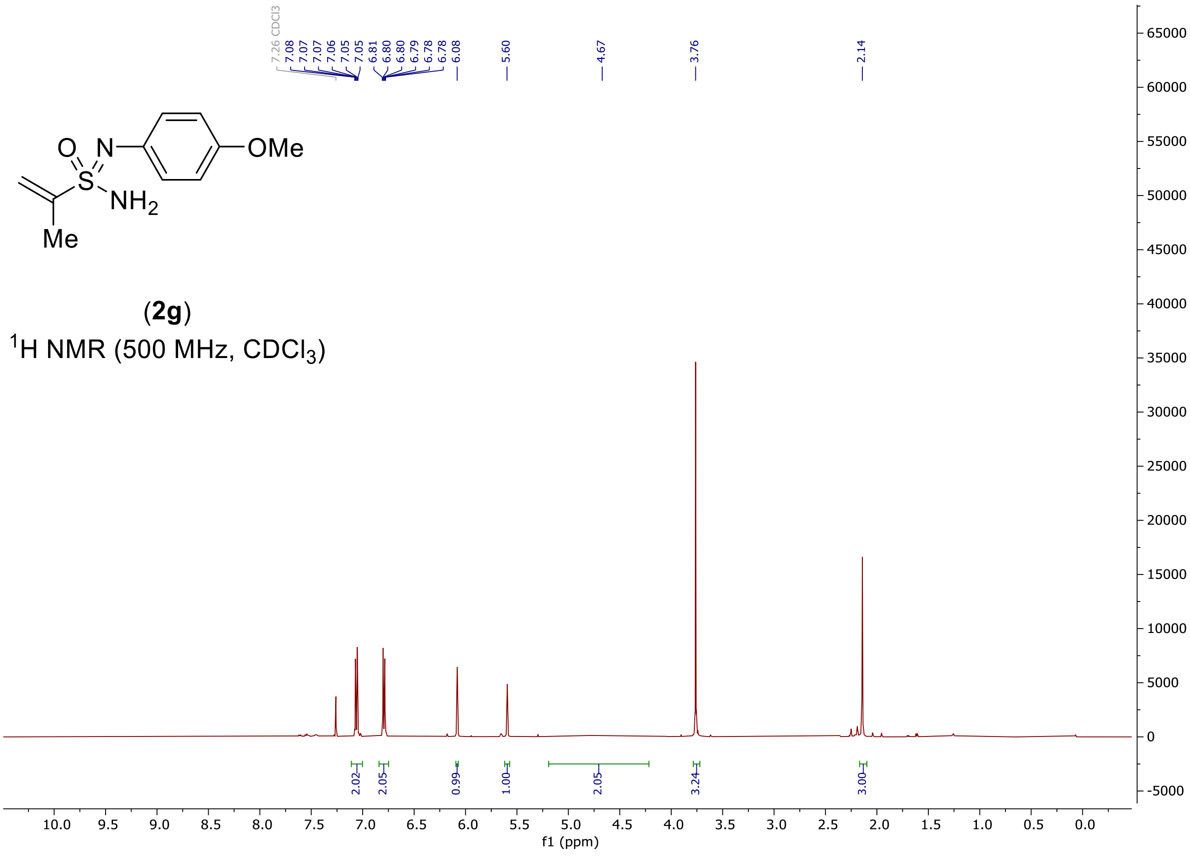

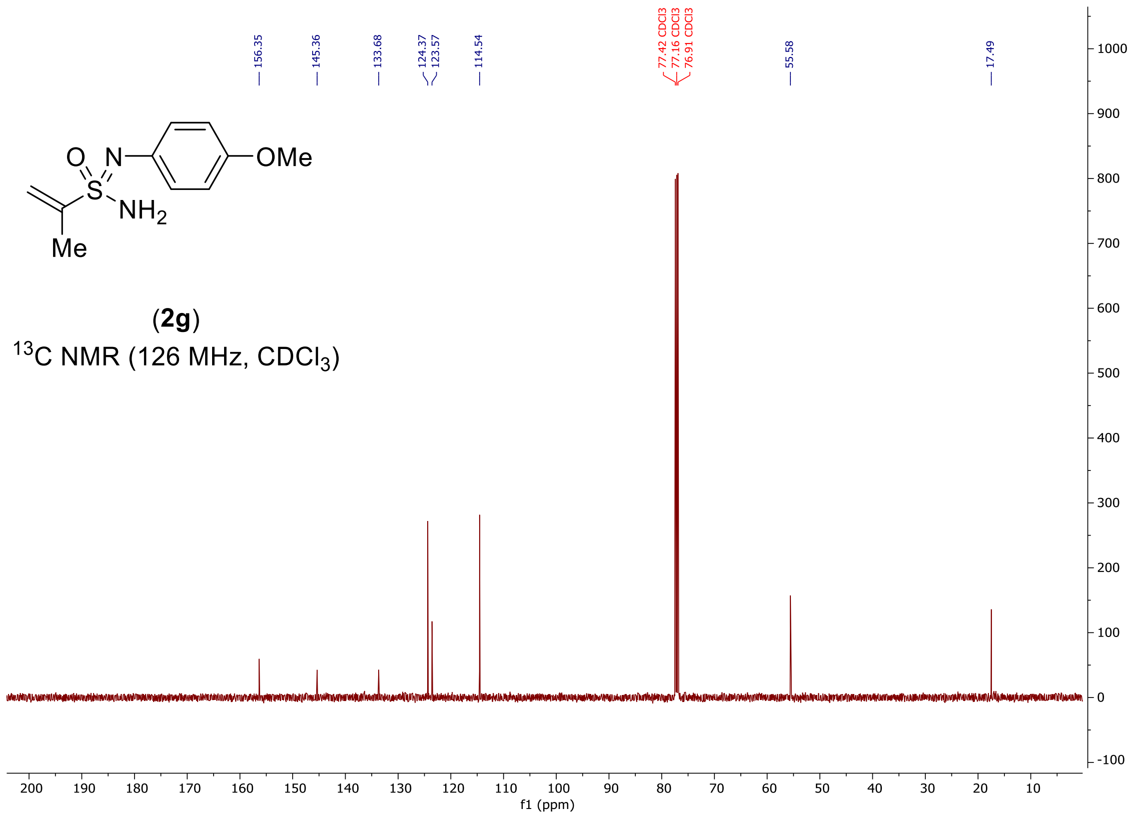

4-(Prop-1-en-1-ylsulfonimidoyl)morpholine (2h)

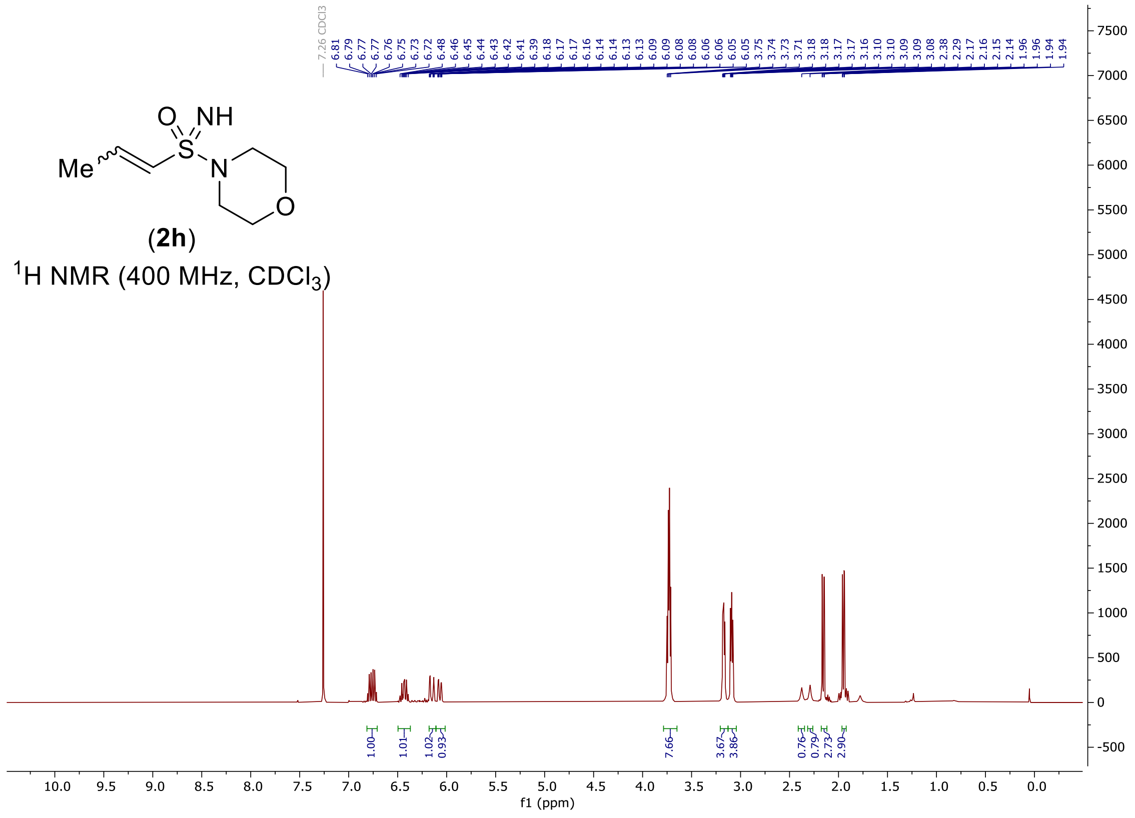

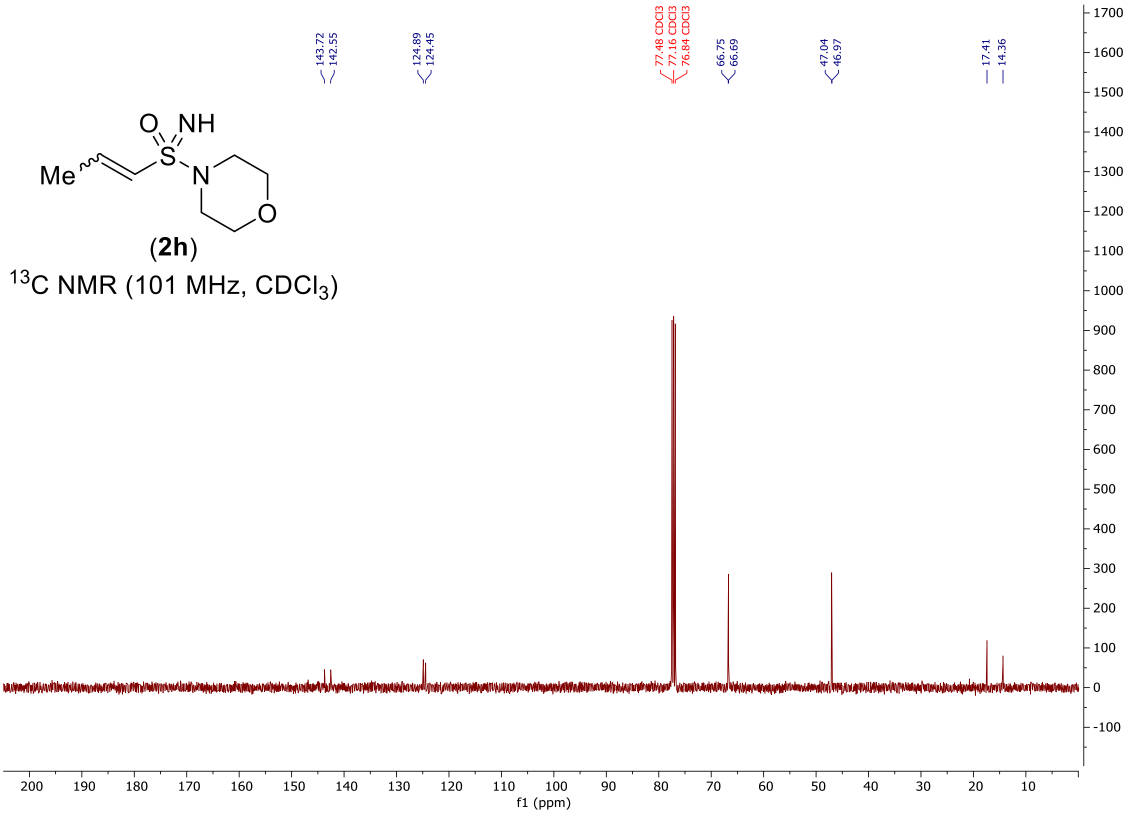

(**2h**)

^{13}C NMR (101 MHz, $CDCl_3$)

4-(Vinylsulfonyl)morpholine (12)

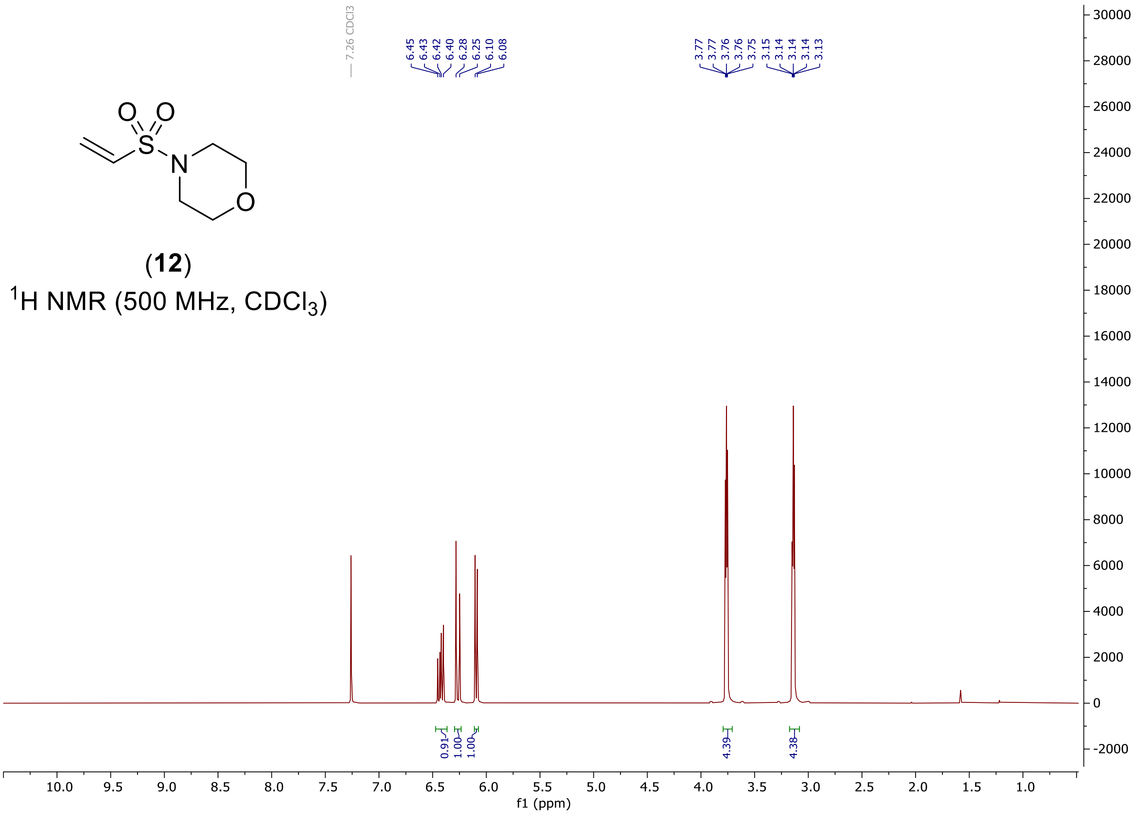

(**12**)

^{1}H NMR (500 MHz, $CDCl_3$)

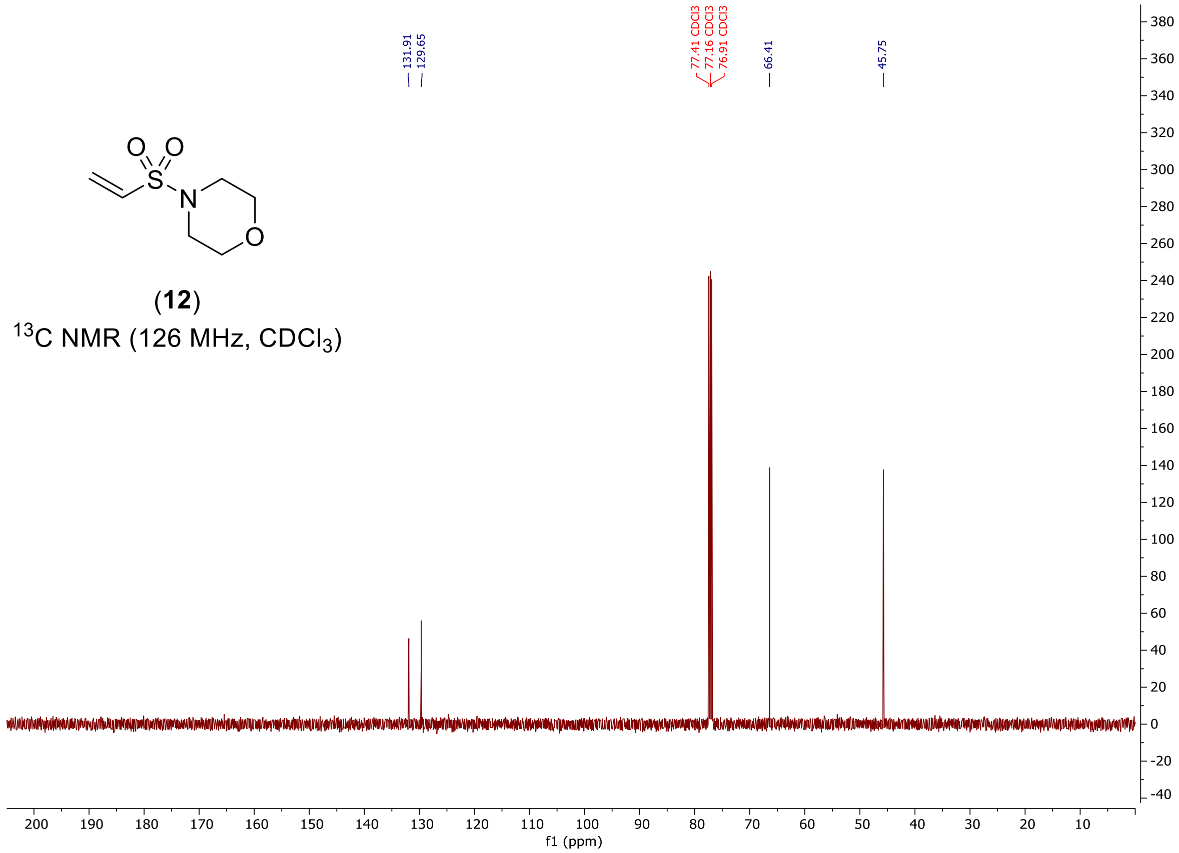

N-(Morpholino(oxo)(vinyl)-λ^6-sulfaneylidene)acetamide (3a)

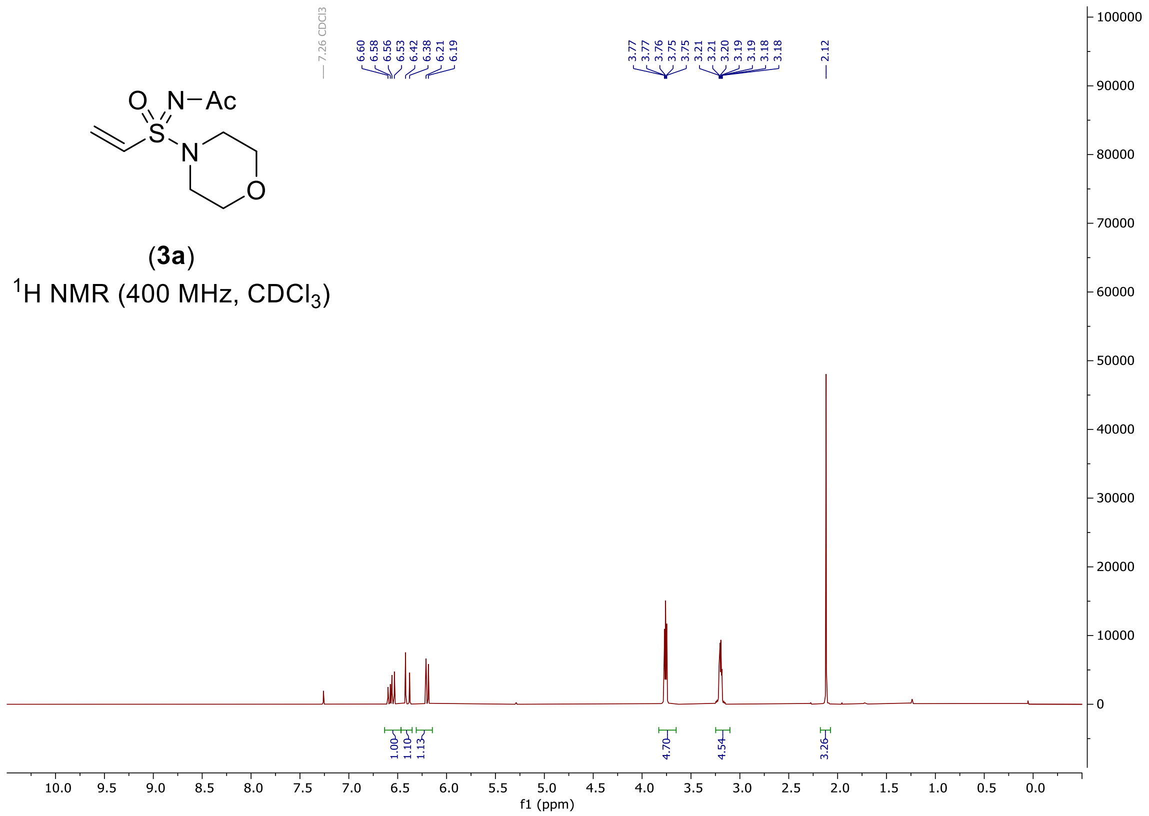

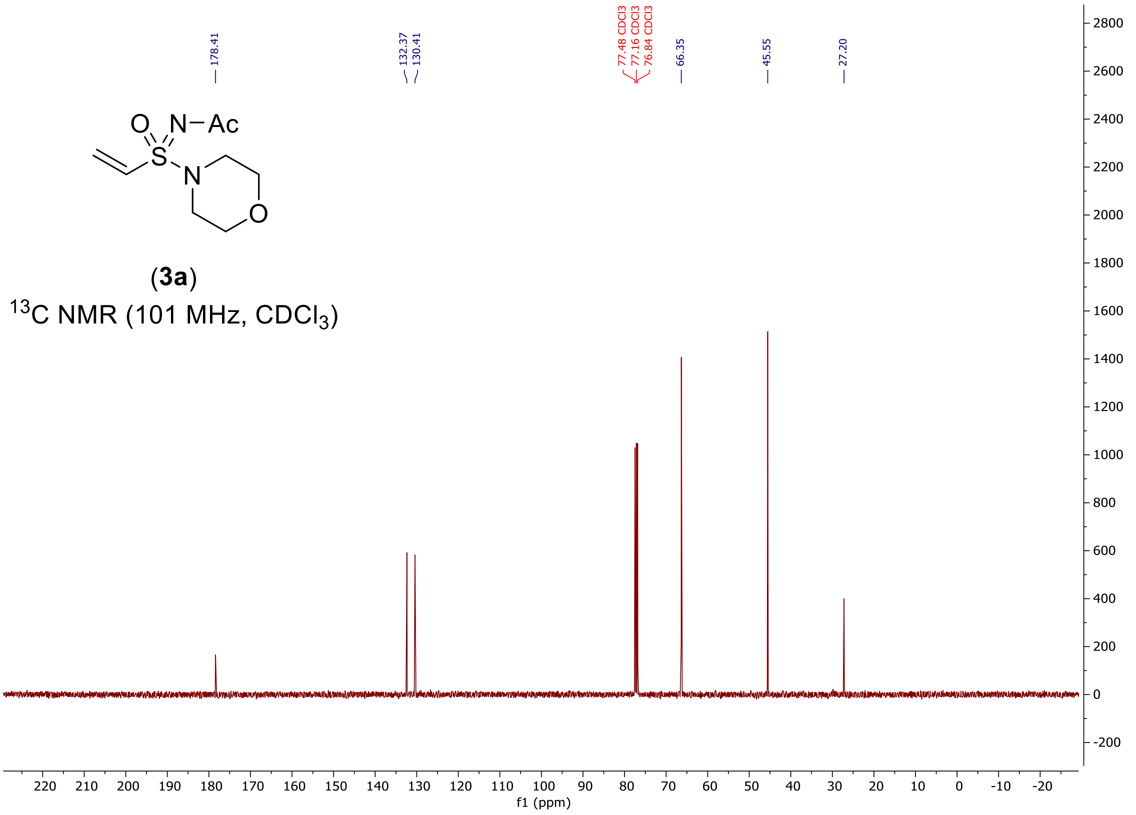

N-(Morpholino(oxo)(vinyl)-λ^6-sulfaneylidene)butyramide (3b)

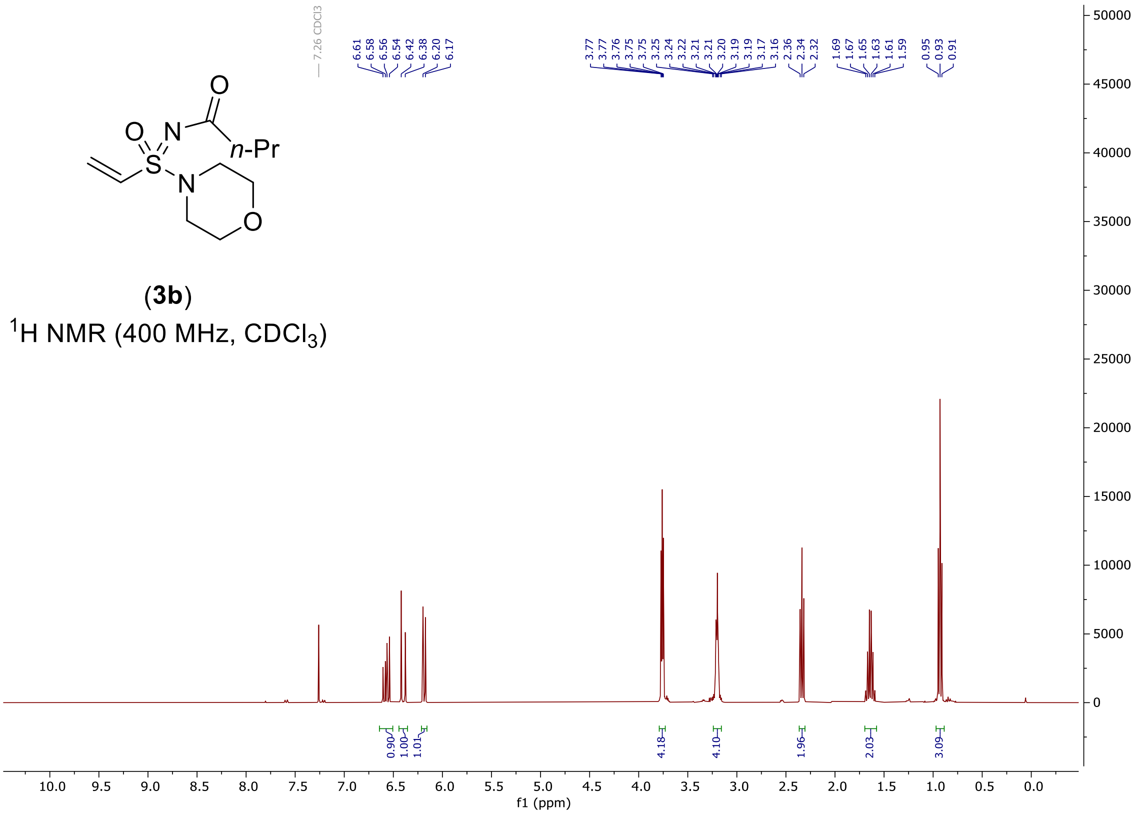

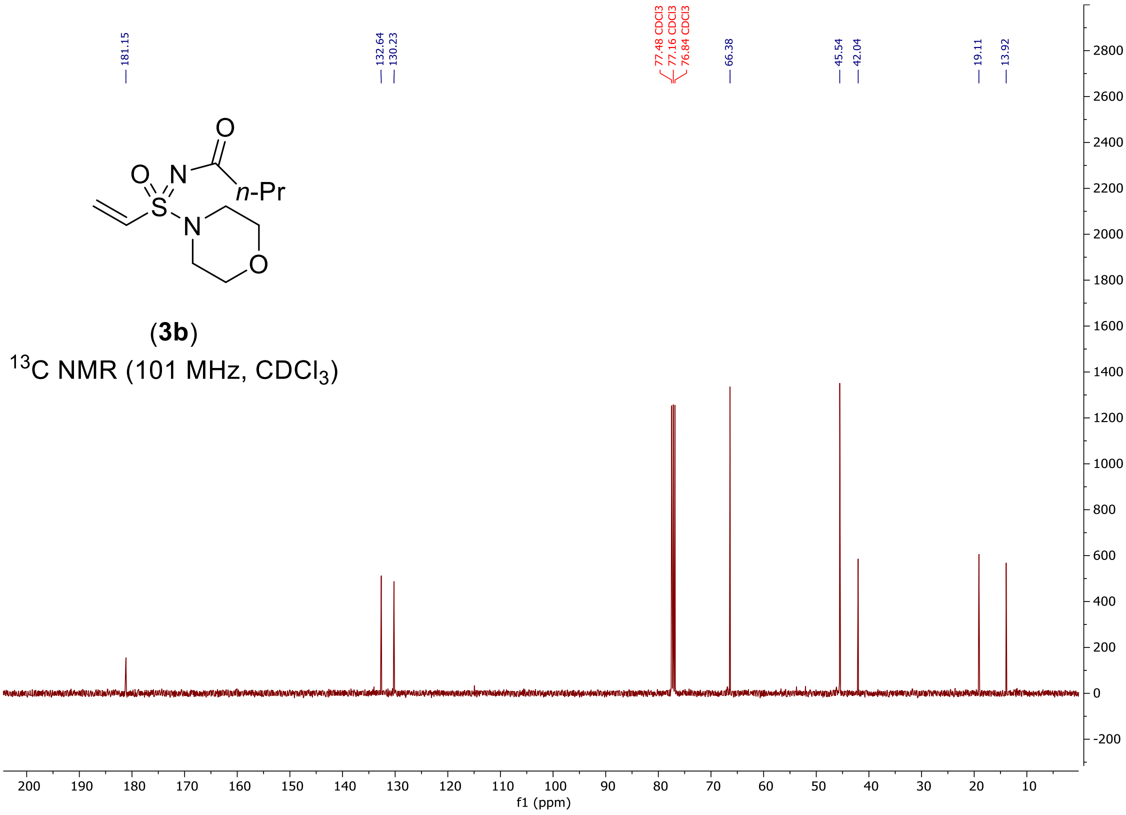

(**3b**)
^{13}C NMR (101 MHz, $CDCl_3$)

Ethyl (morpholino(oxo)(vinyl)- λ^6-sulfaneylidene)carbamate (3c)

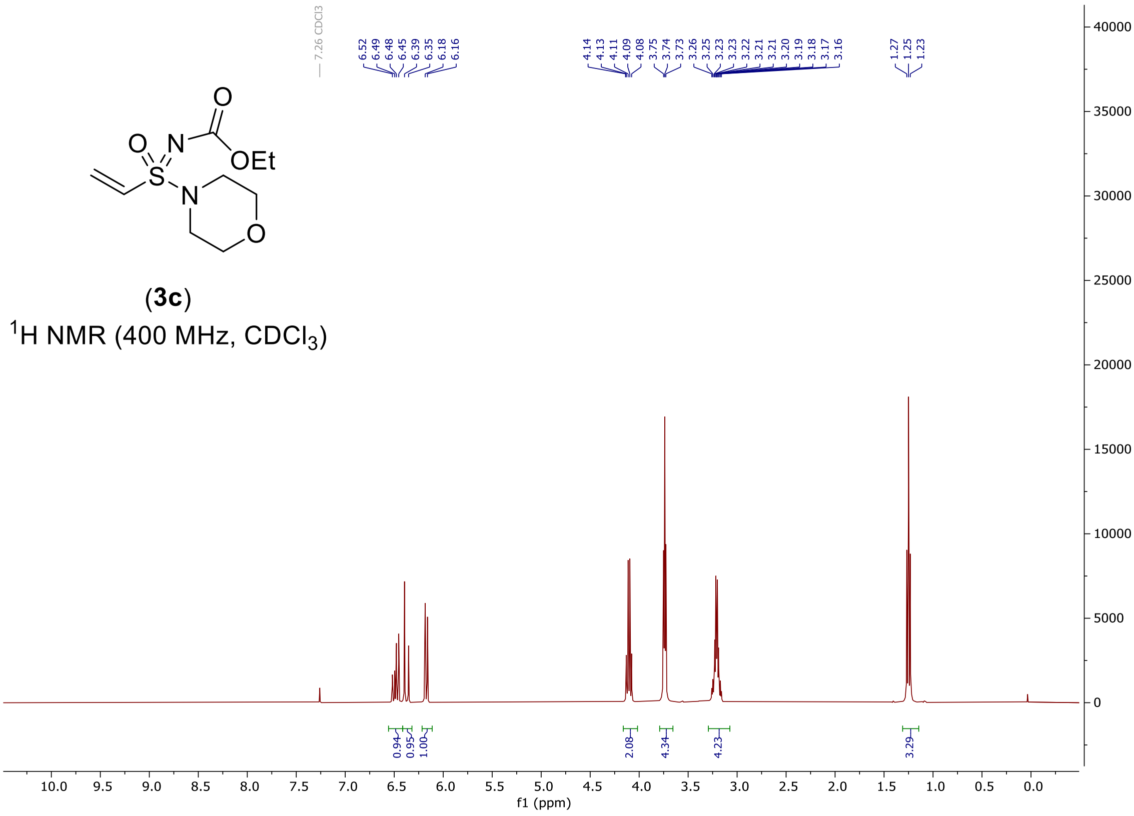

(**3c**)
^1H NMR (400 MHz, $CDCl_3$)

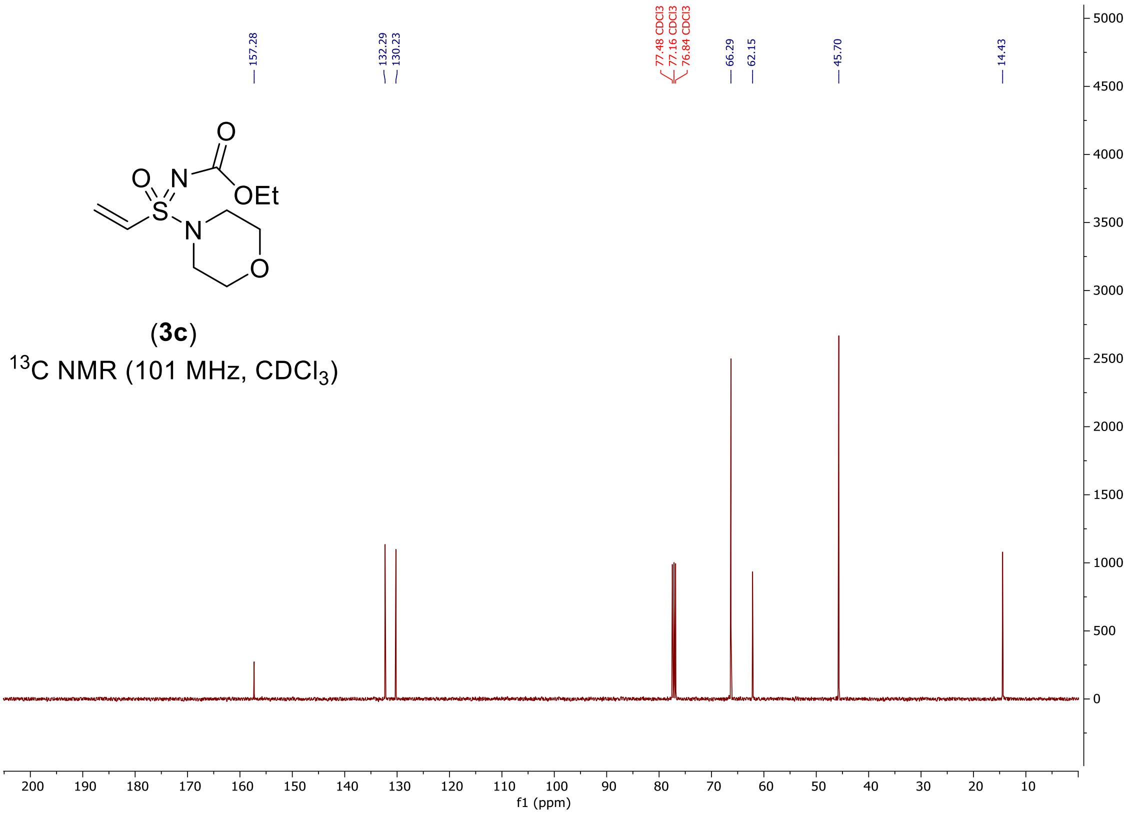

1-(Morpholino(oxo)(vinyl)- λ⁶-sulfaneylidene)-3-(*p*-tolyl)urea (3d)

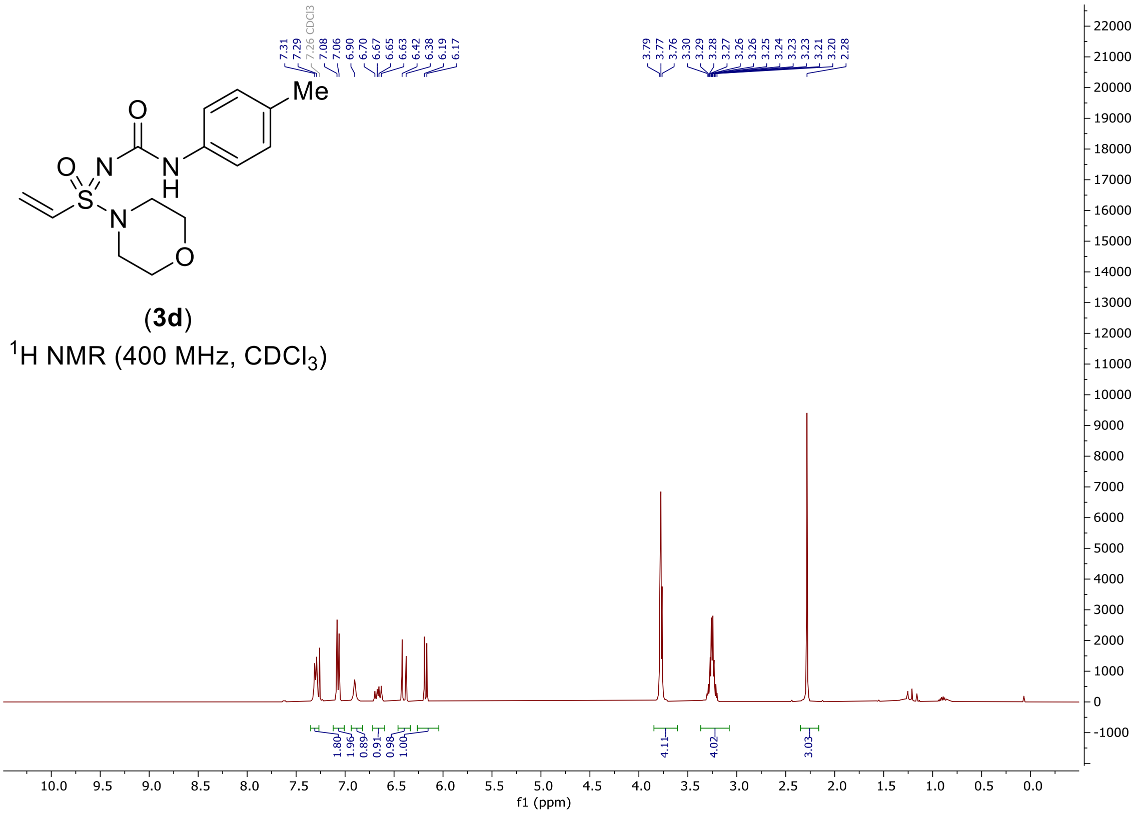

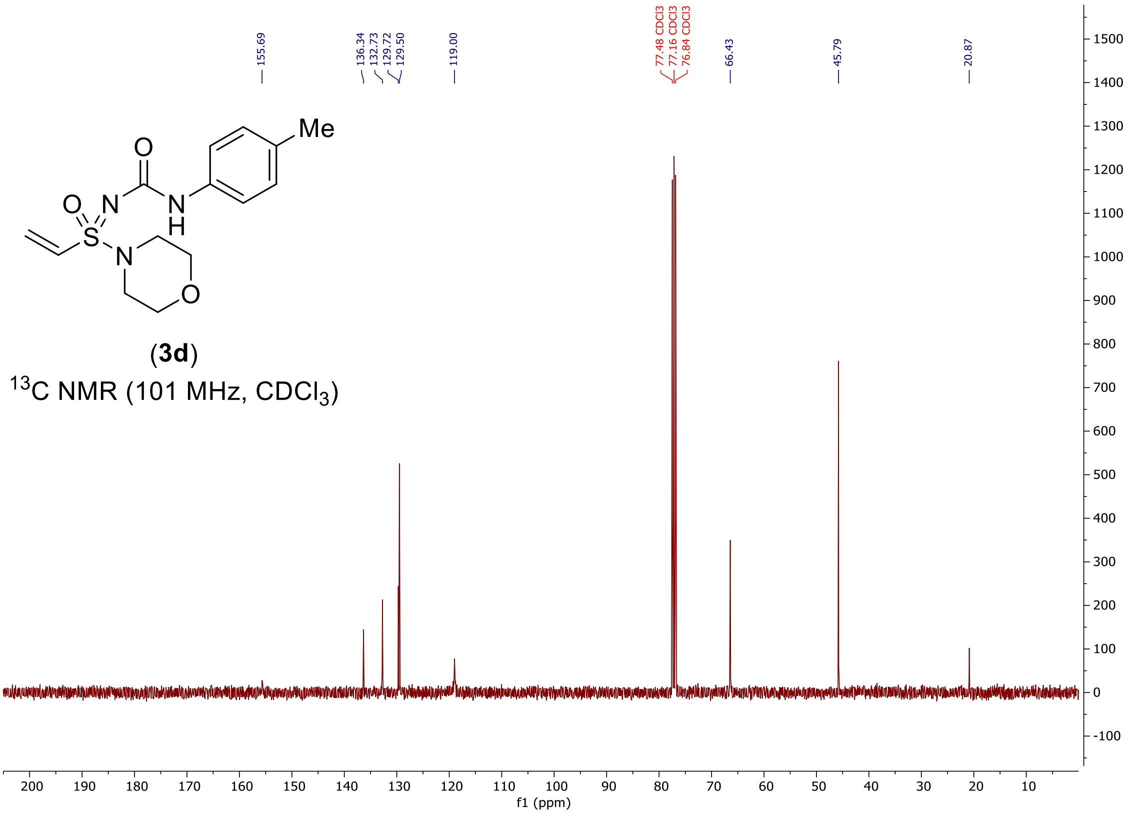

N-(Morpholino(oxo)(vinyl)- λ^{6}-sulfaneylidene)methanesulfonamide (3e)

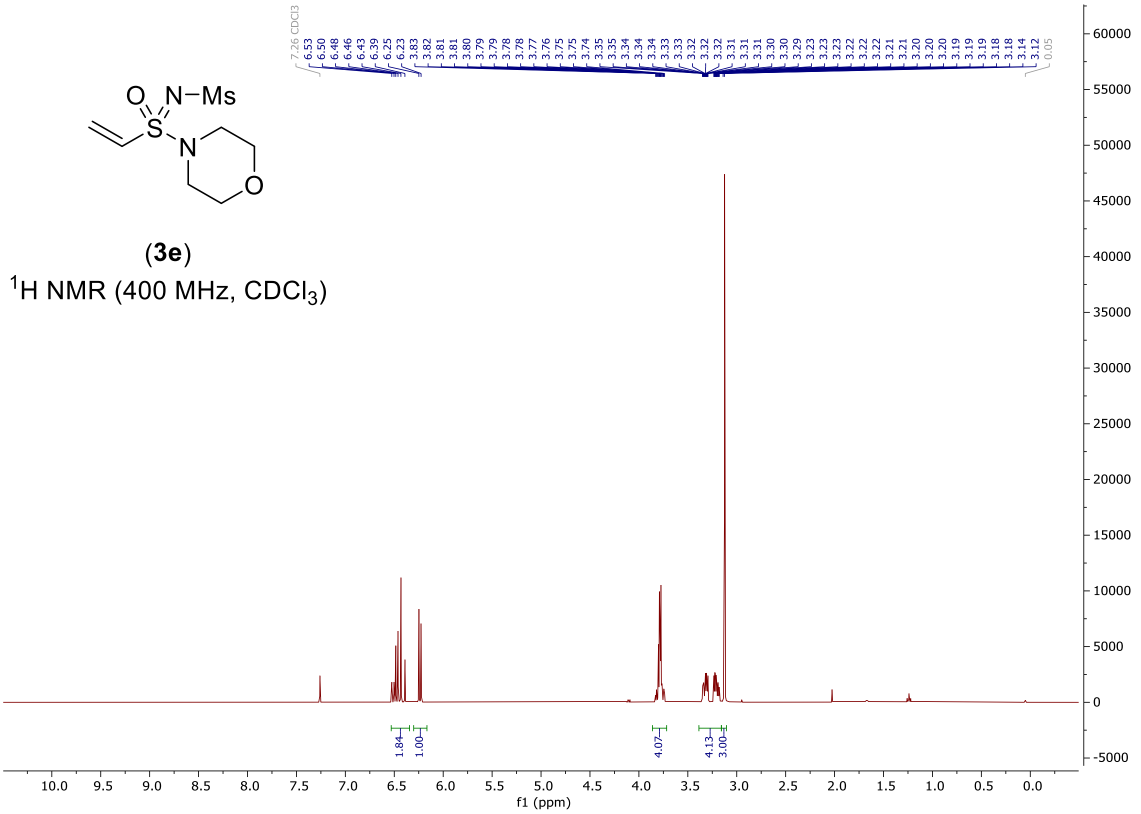

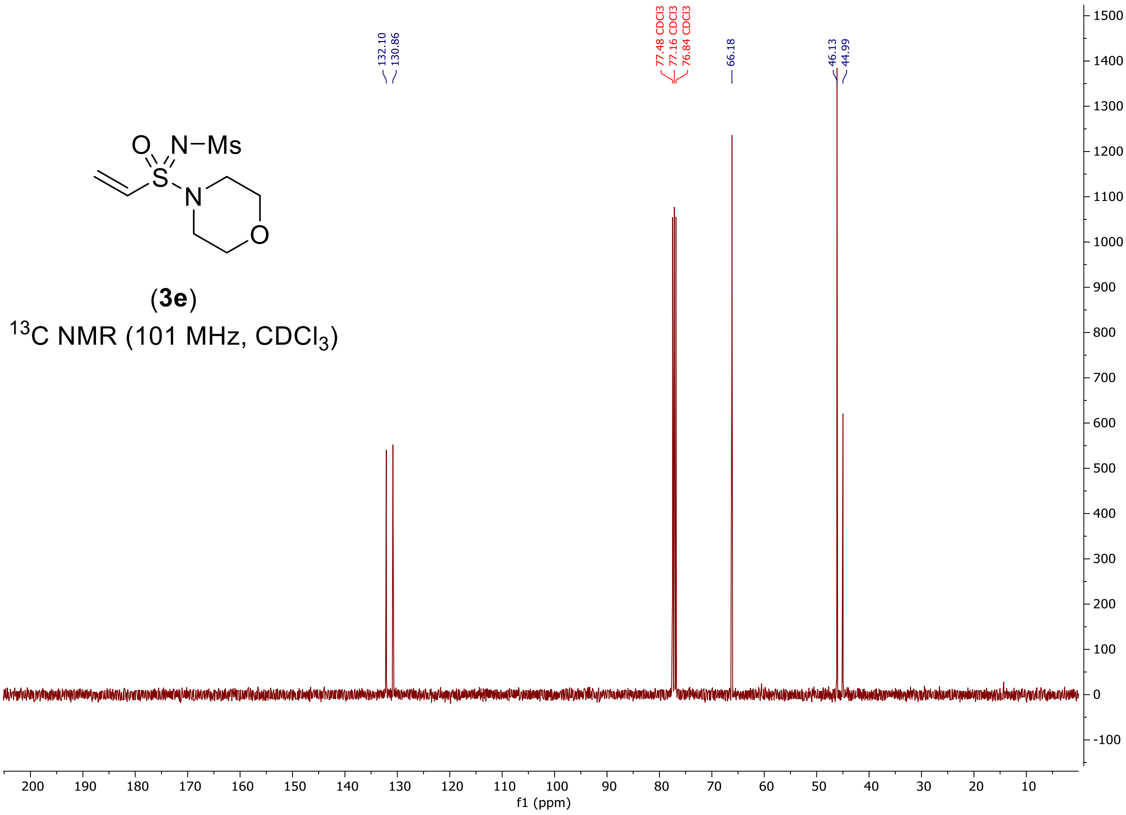

N-(morpholino(oxo)(vinyl)- λ^6-sulfaneylidene)cyanamide (3f)

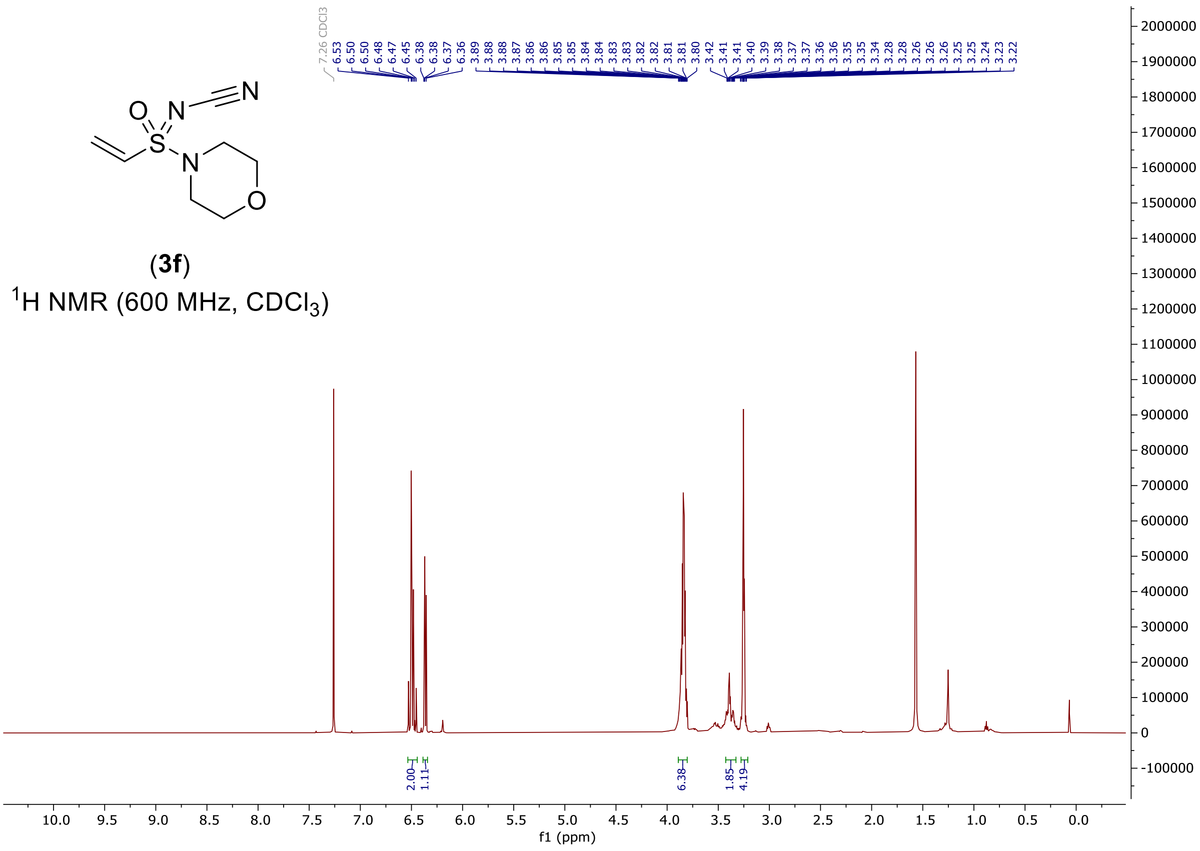

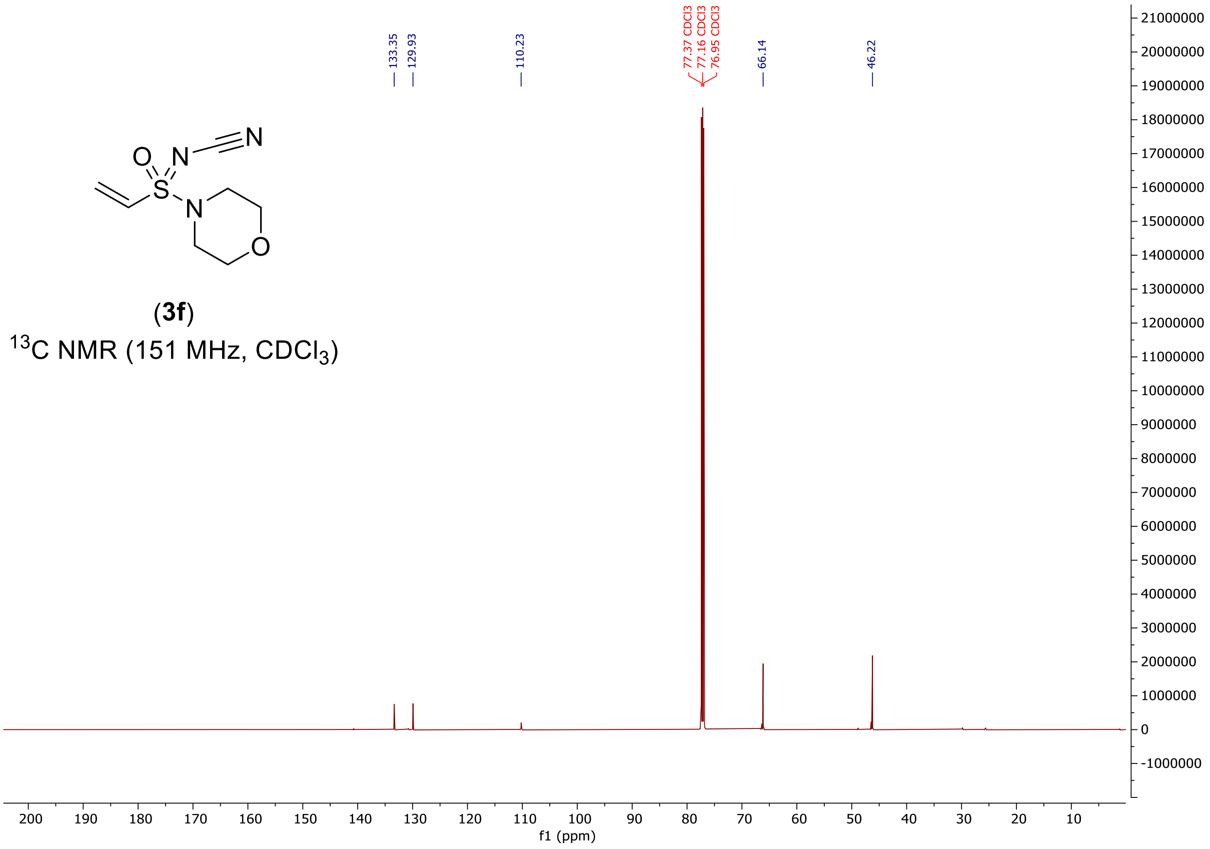

4-(*N*-Butylvinylsulfonimidoyl)morpholine (3g)

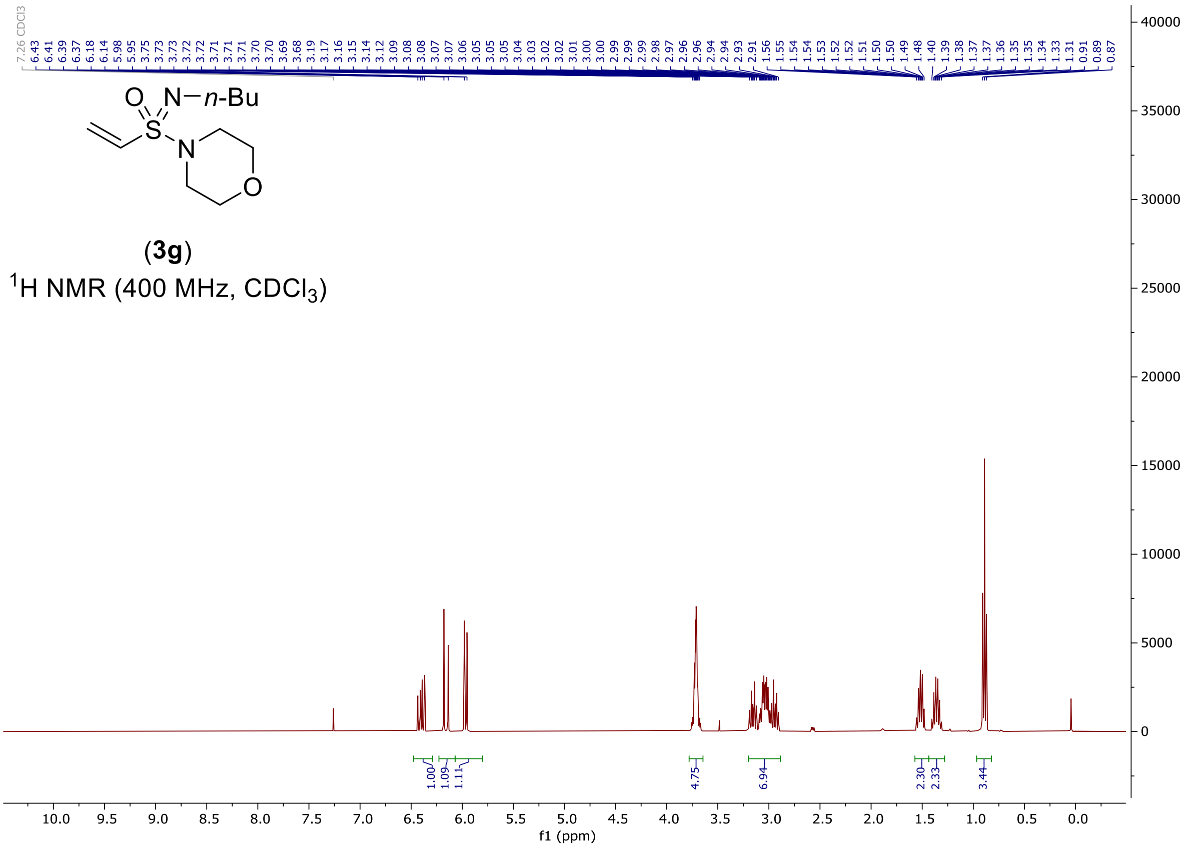

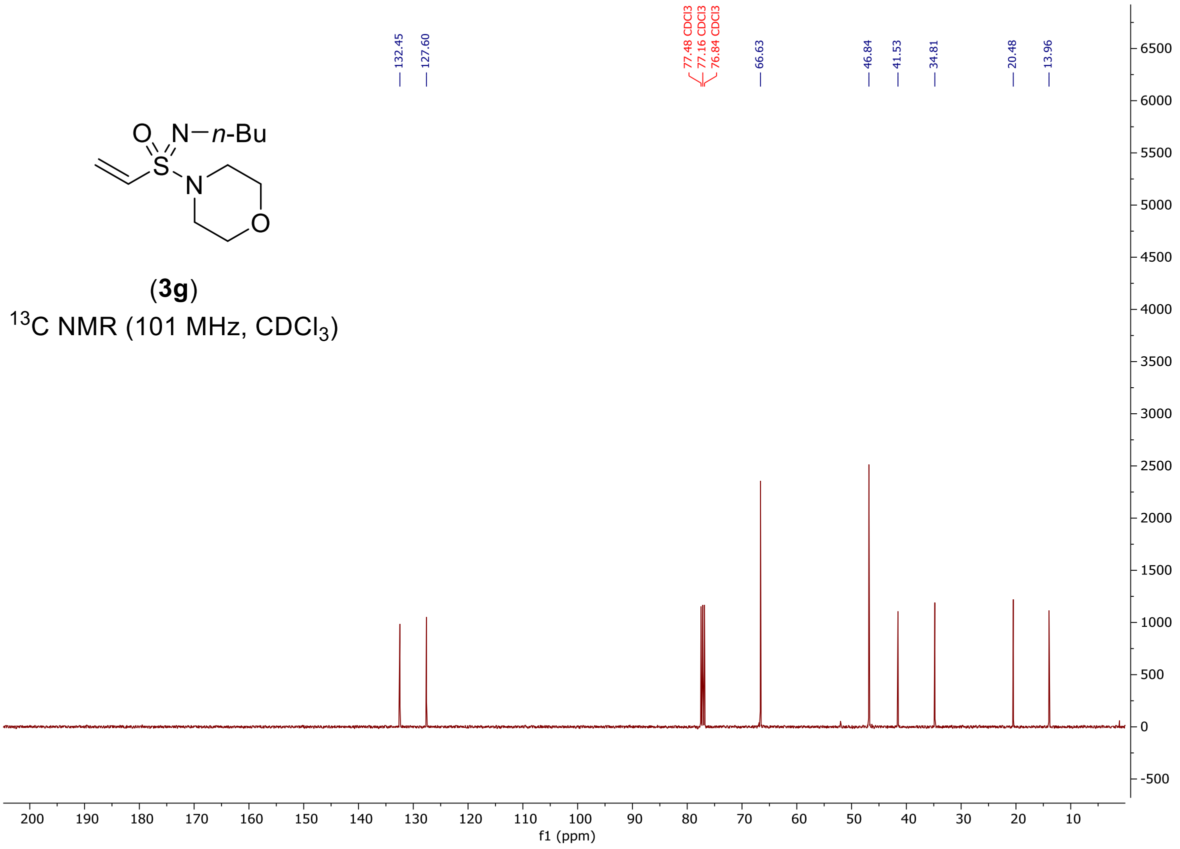

4-(*N*-Ethylvinylsulfonimidoyl)morpholine (3h)

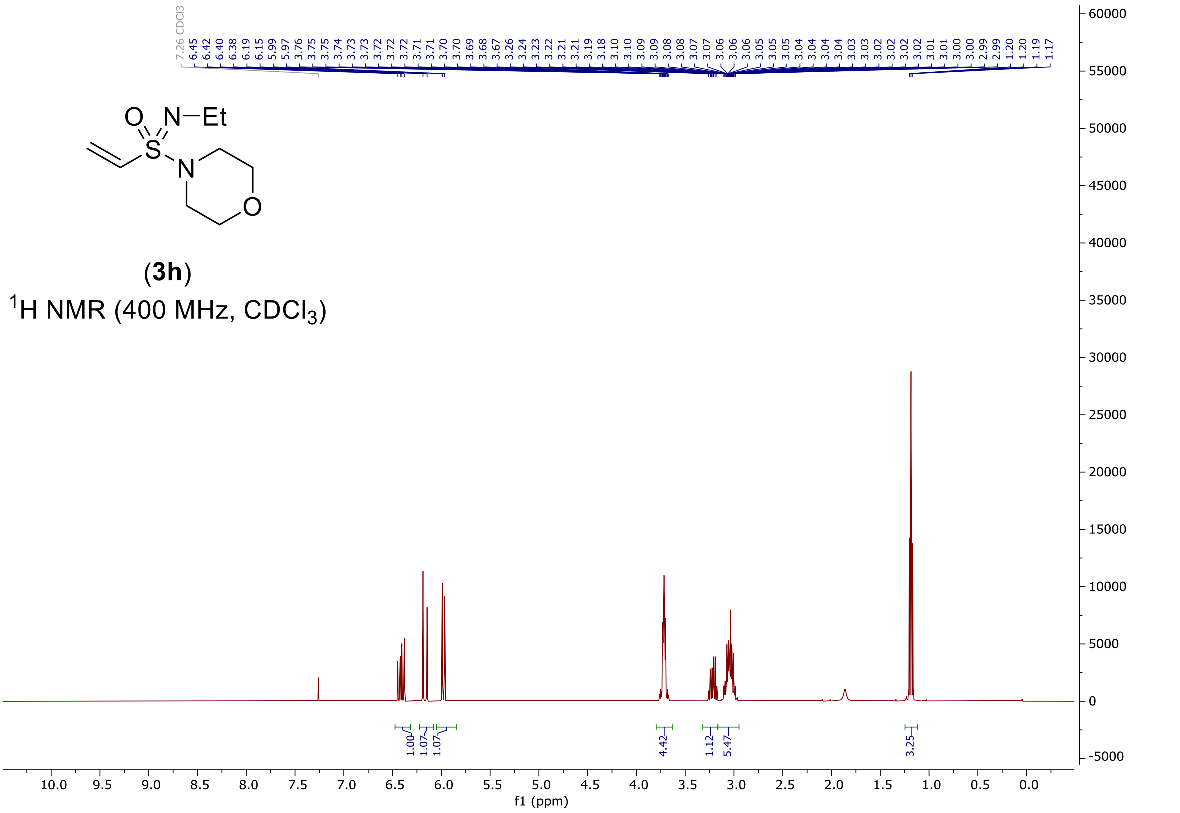

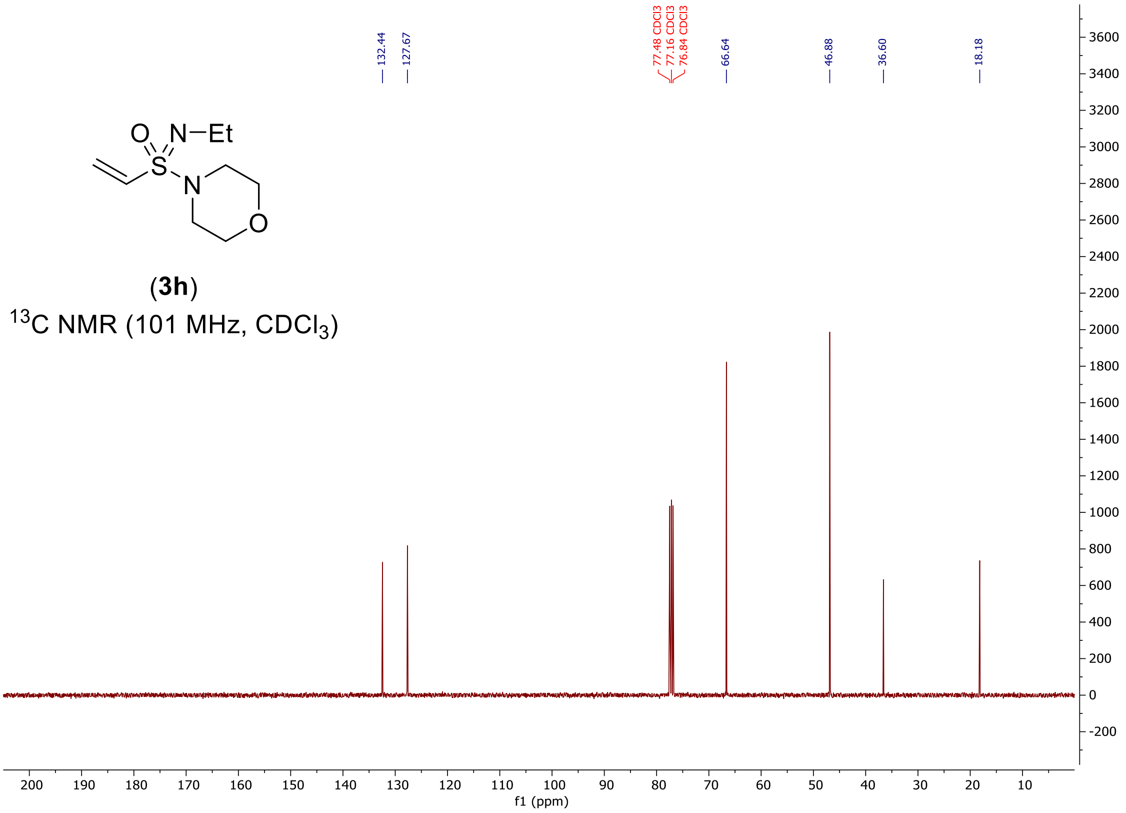

N-(*tert*-Butyl)ethenesulfinamide (SI-4)

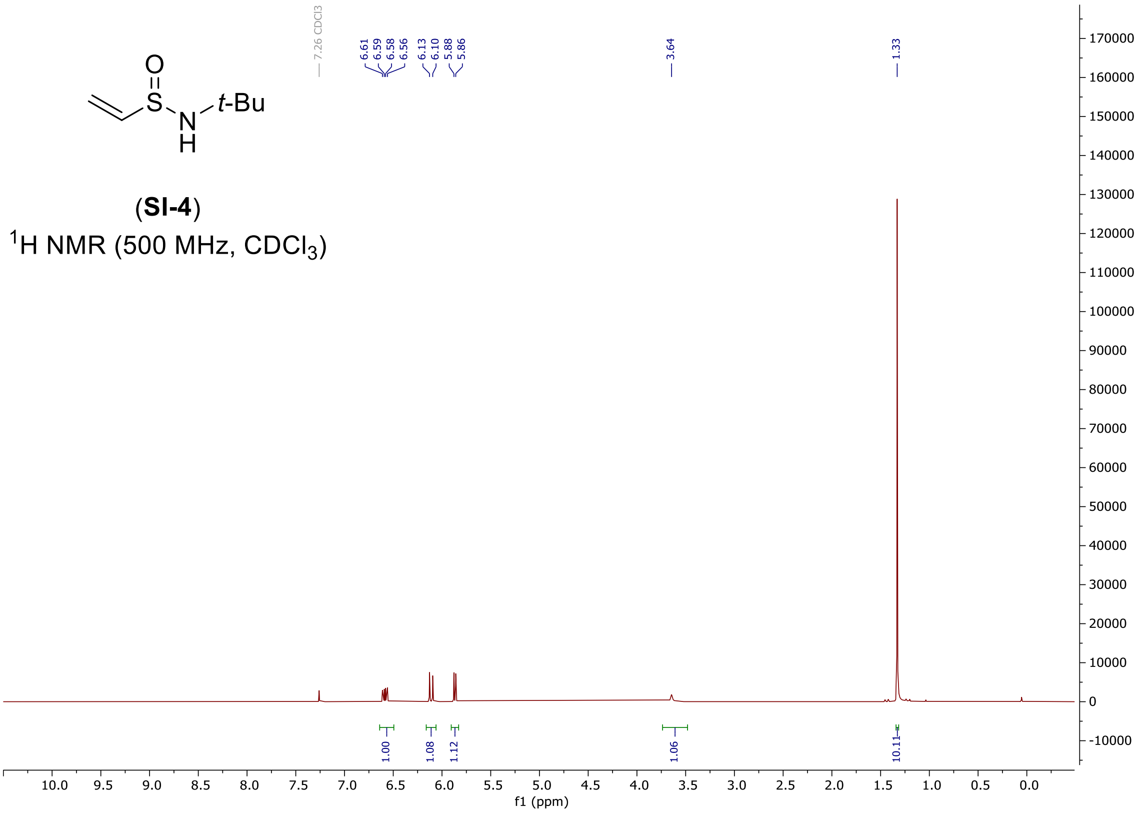

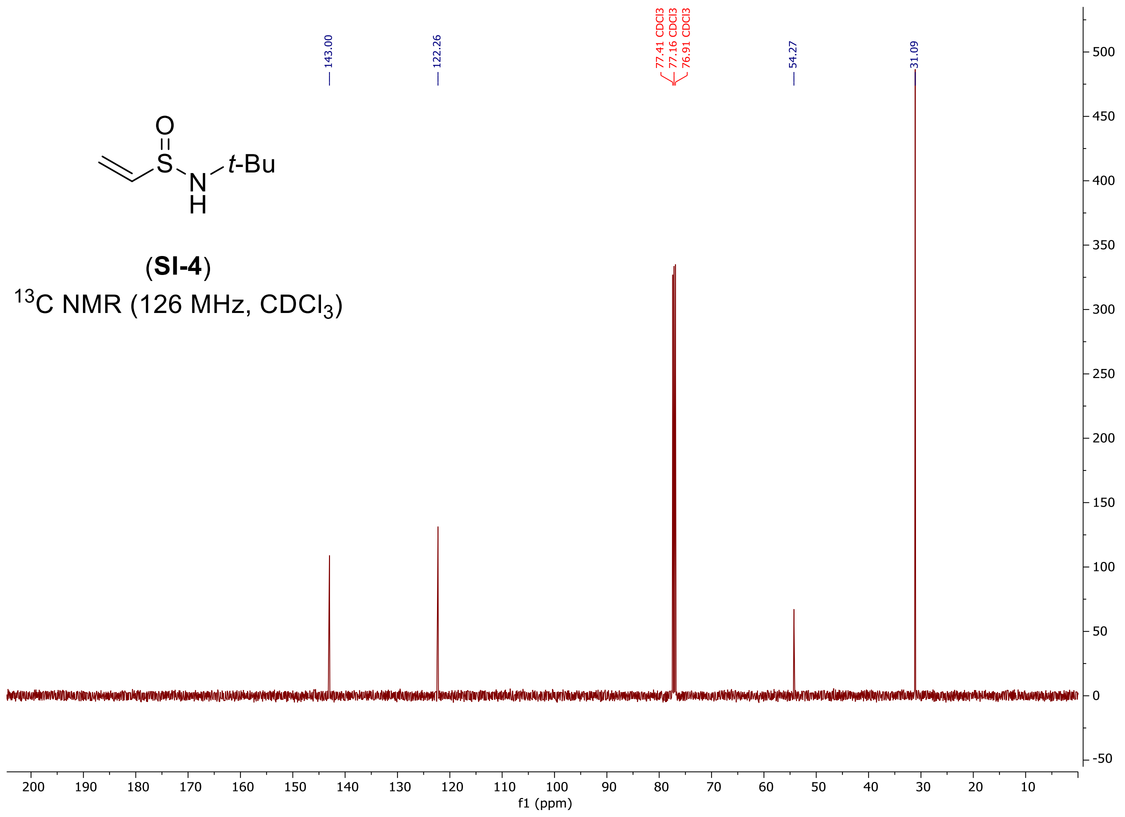

4-(*N*-(*tert*-Butyl)vinylsulfonimidoyl)morpholine (3i)

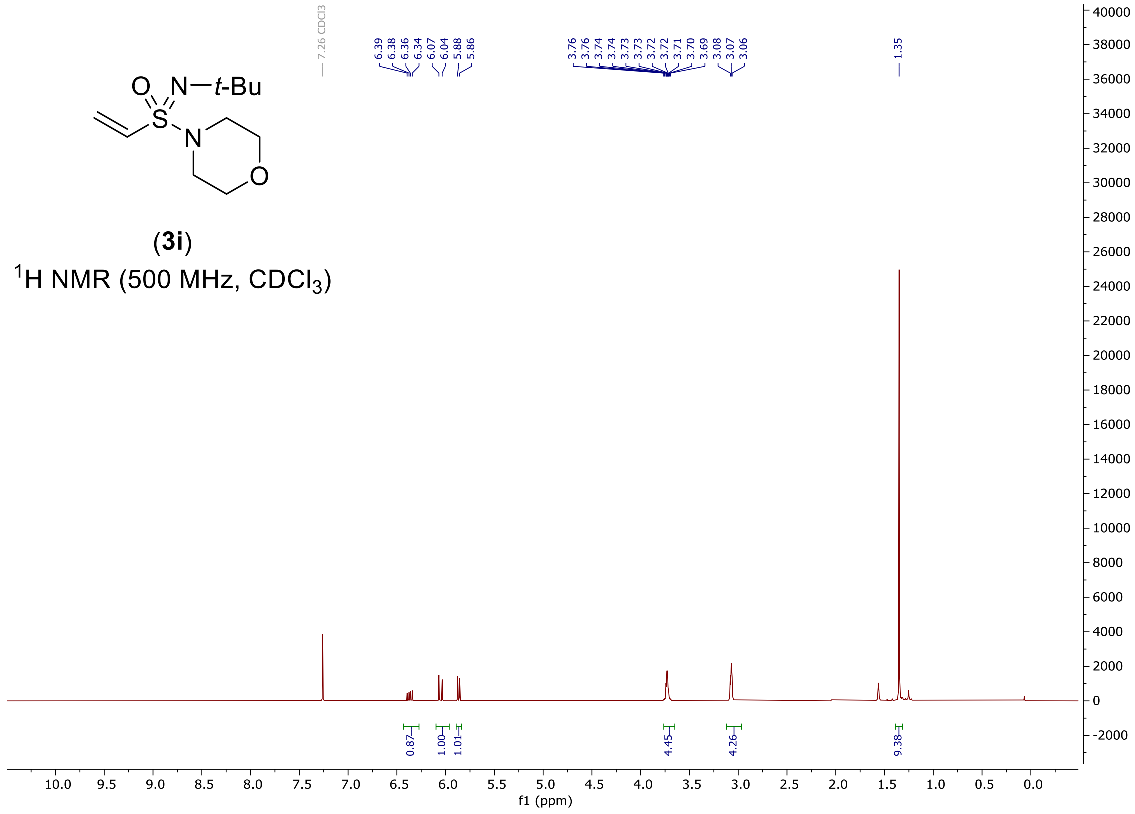

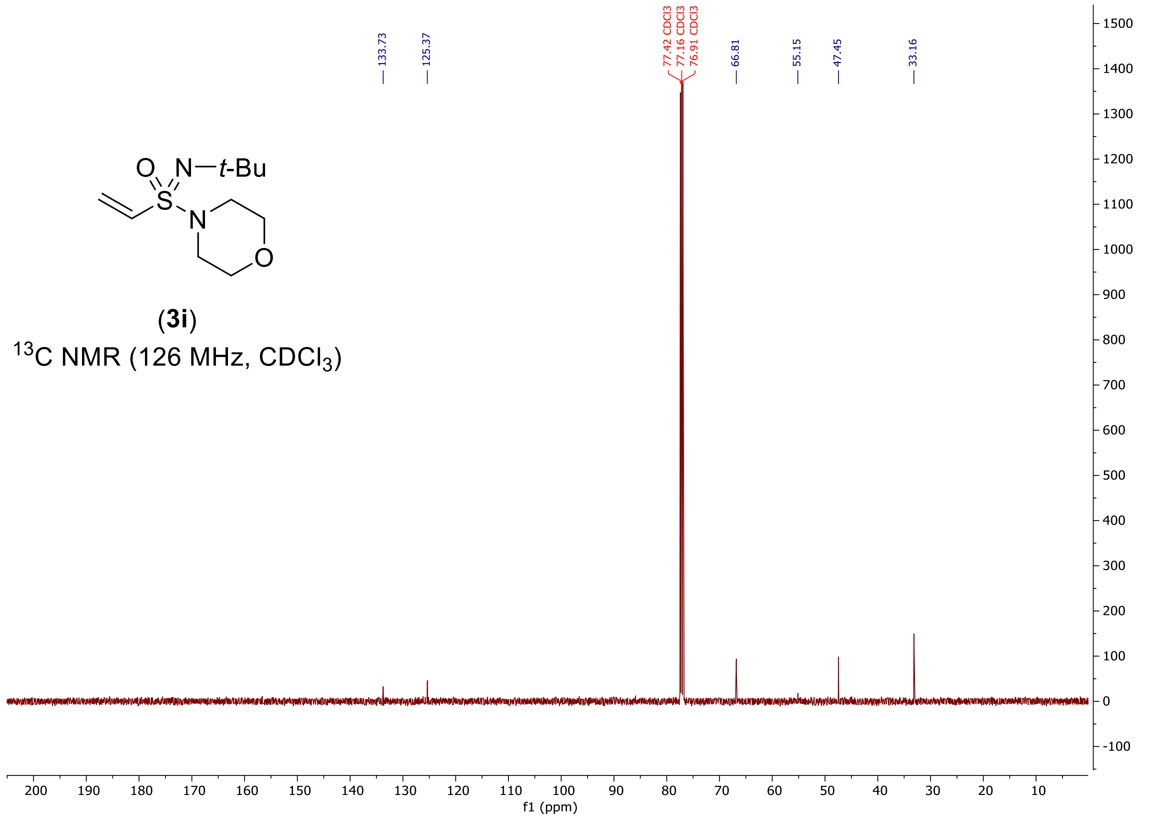

4-(*N*-(*p*-Tolyl)vinylsulfonimidoyl)morpholine (3j)

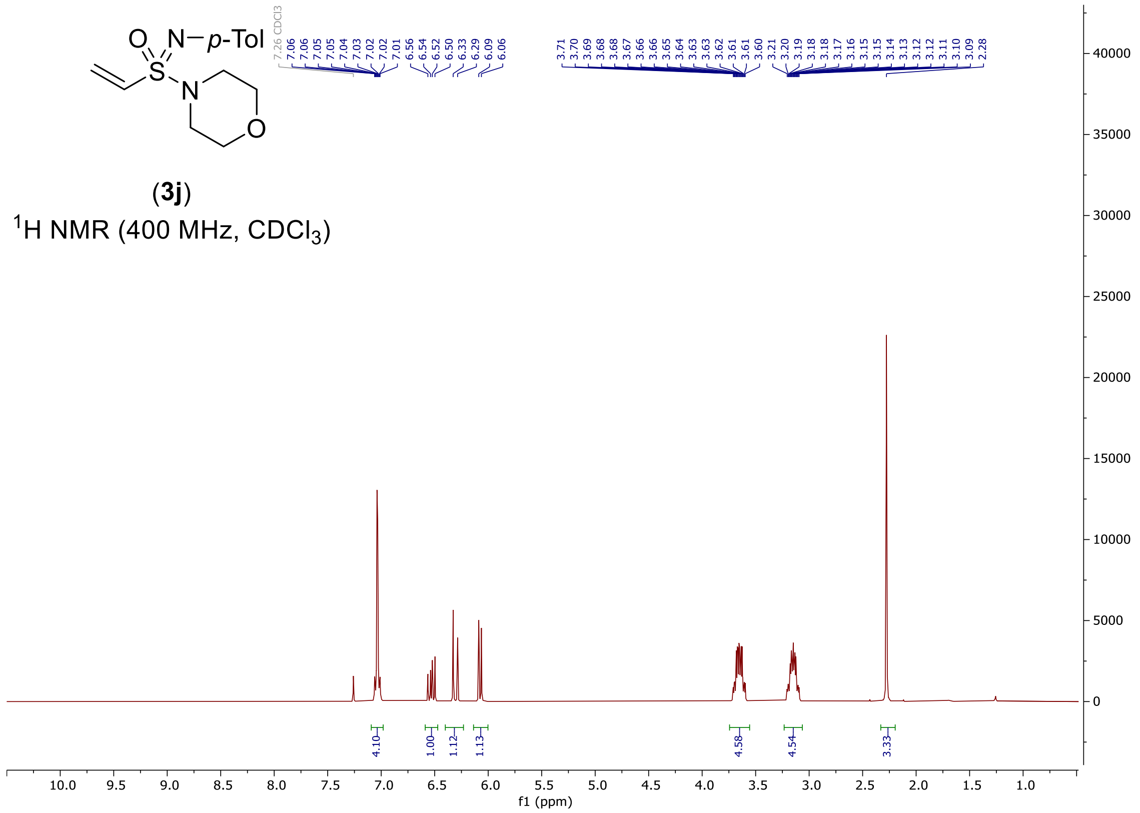

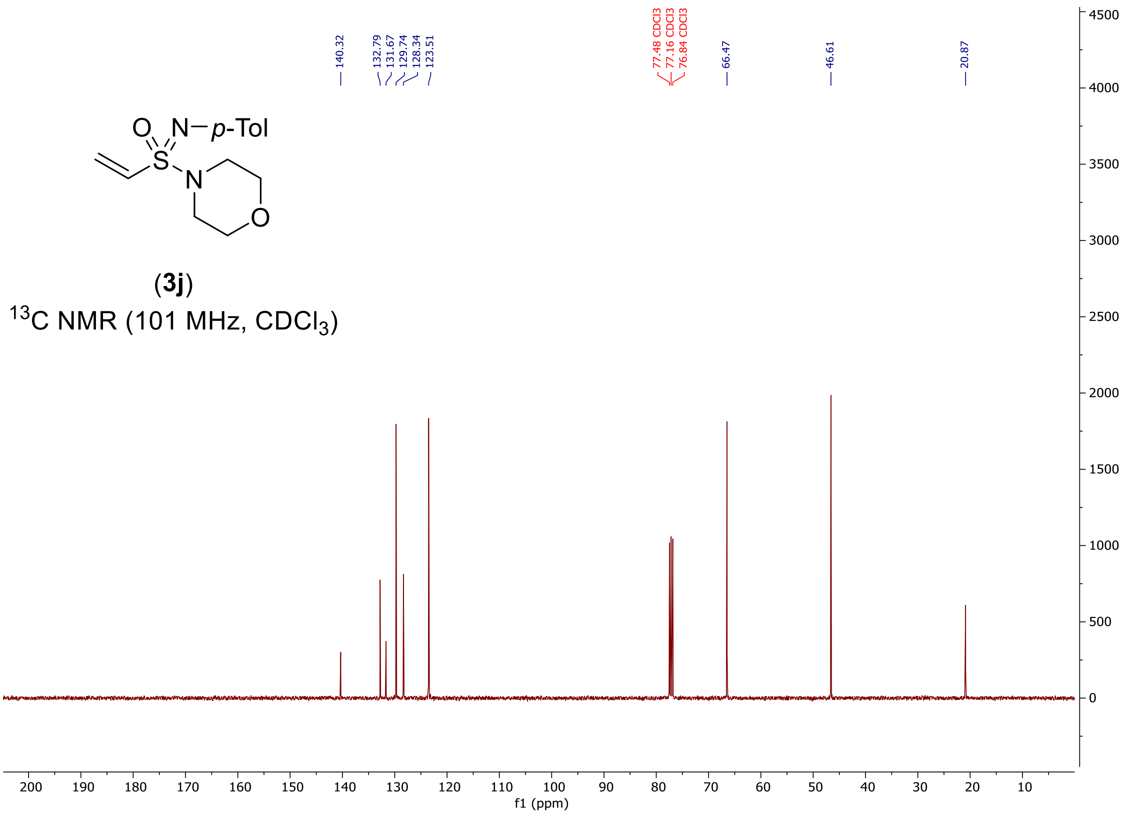

N-(morpholino(oxo)(prop-1-en-2-yl)-l6-sulfaneylidene)acetamide (4a)

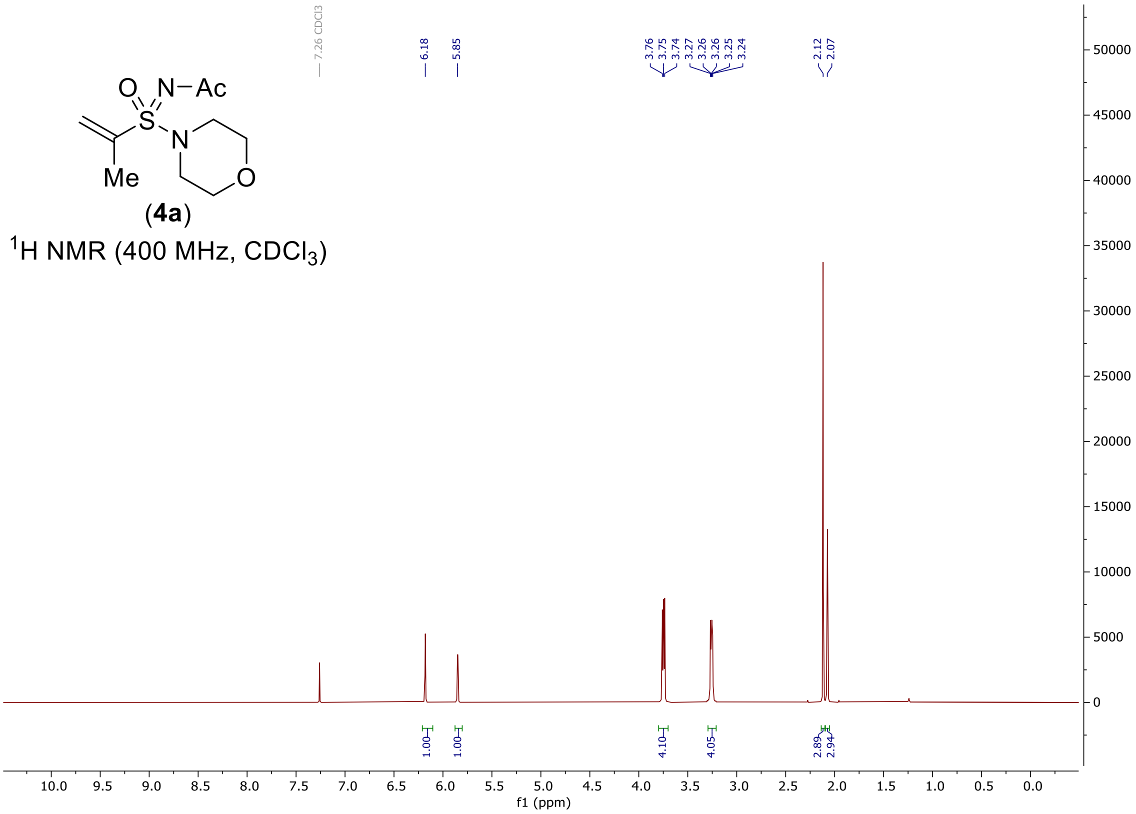

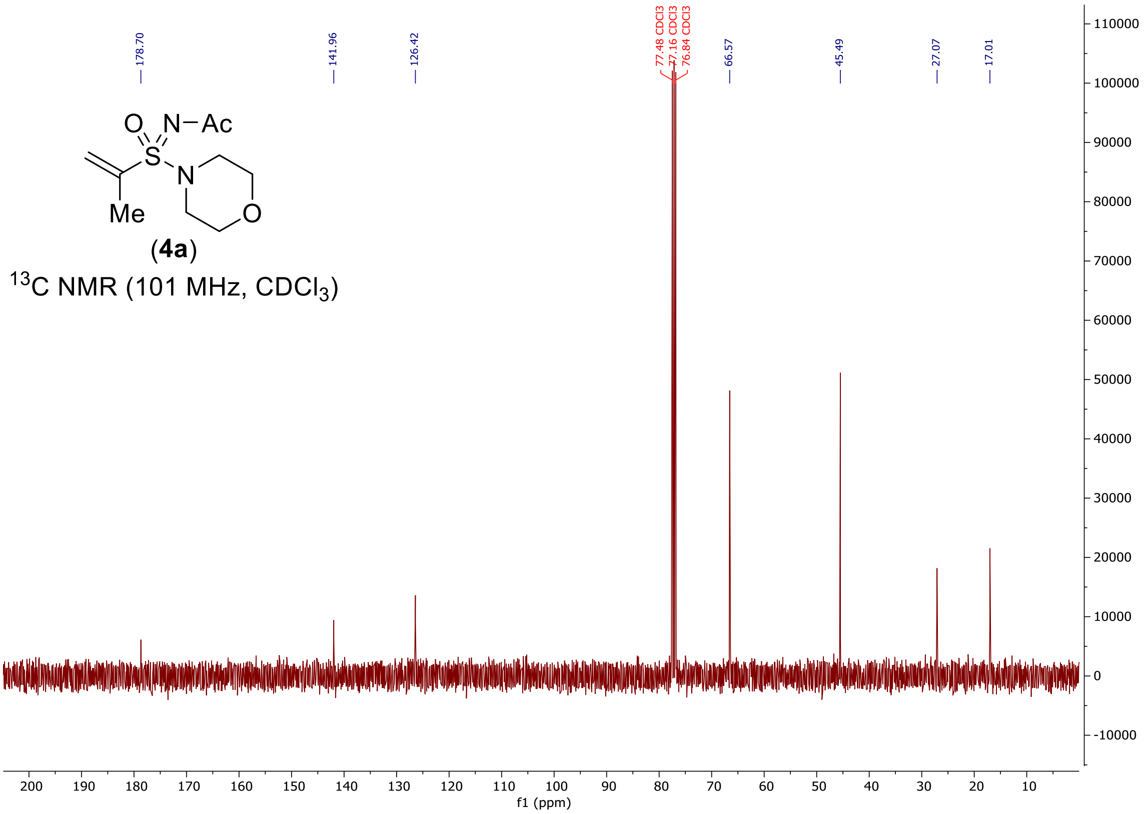

N-(morpholino(oxo)(prop-1-en-2-yl)-l6-sulfaneylidene)methanesulfonamide (4b)

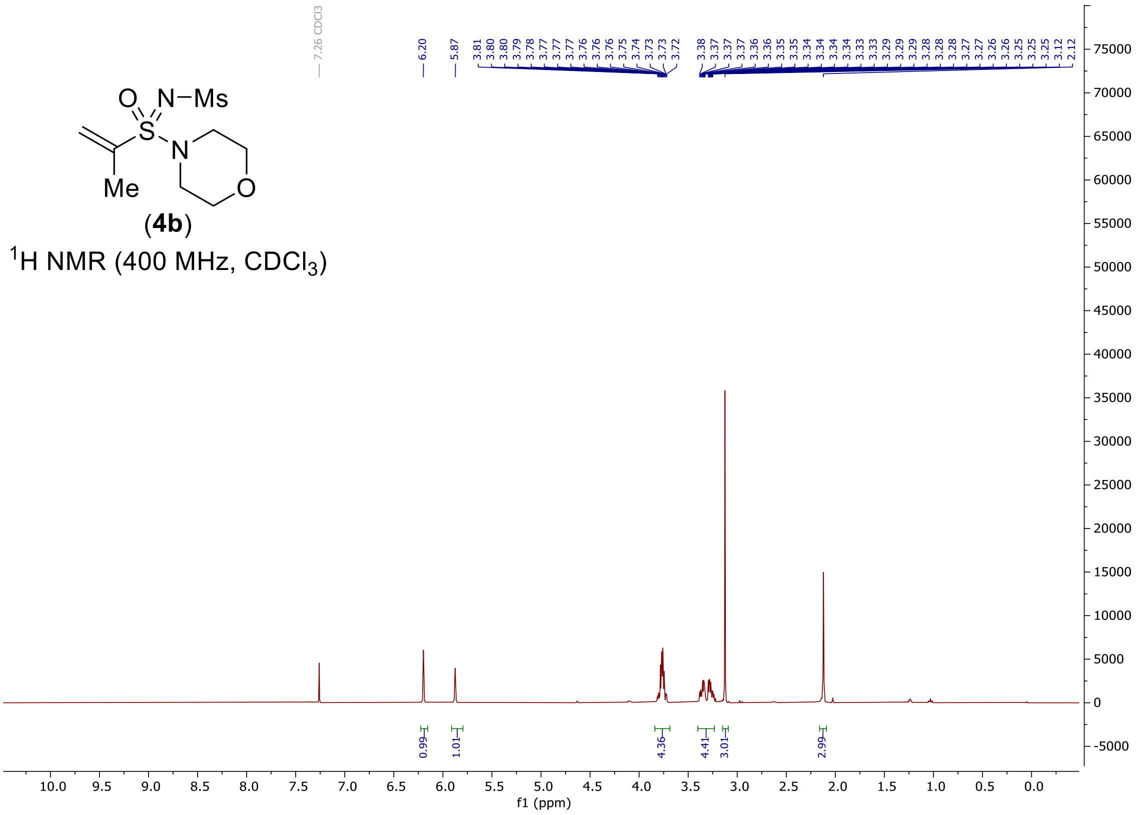

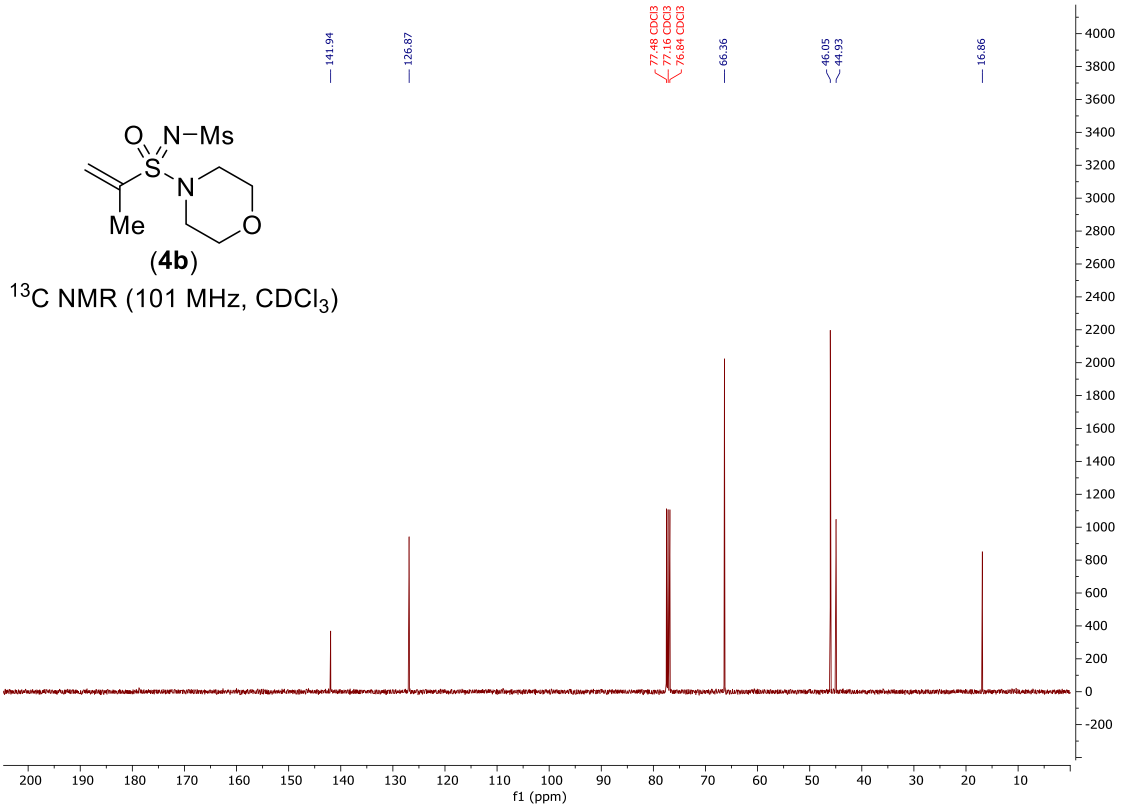

4-(*N*-butylprop-1-en-2-ylsulfonimidoyl)morpholine (4c)

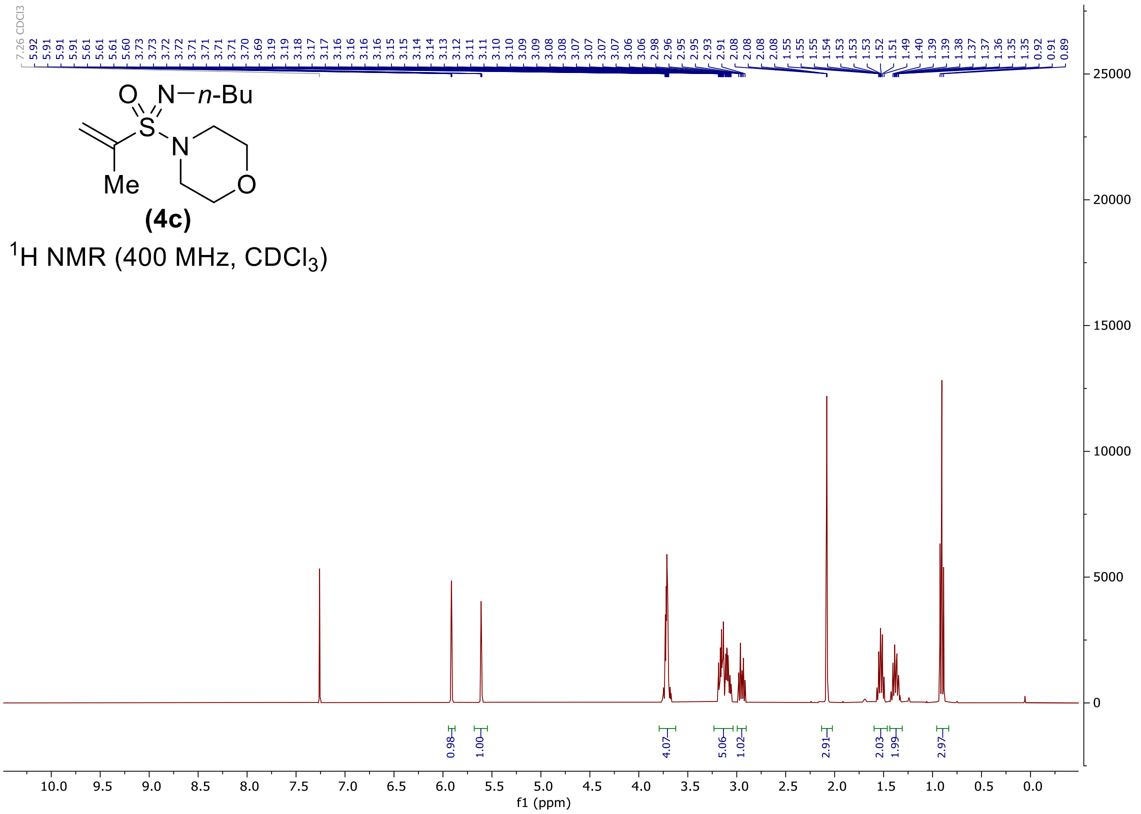

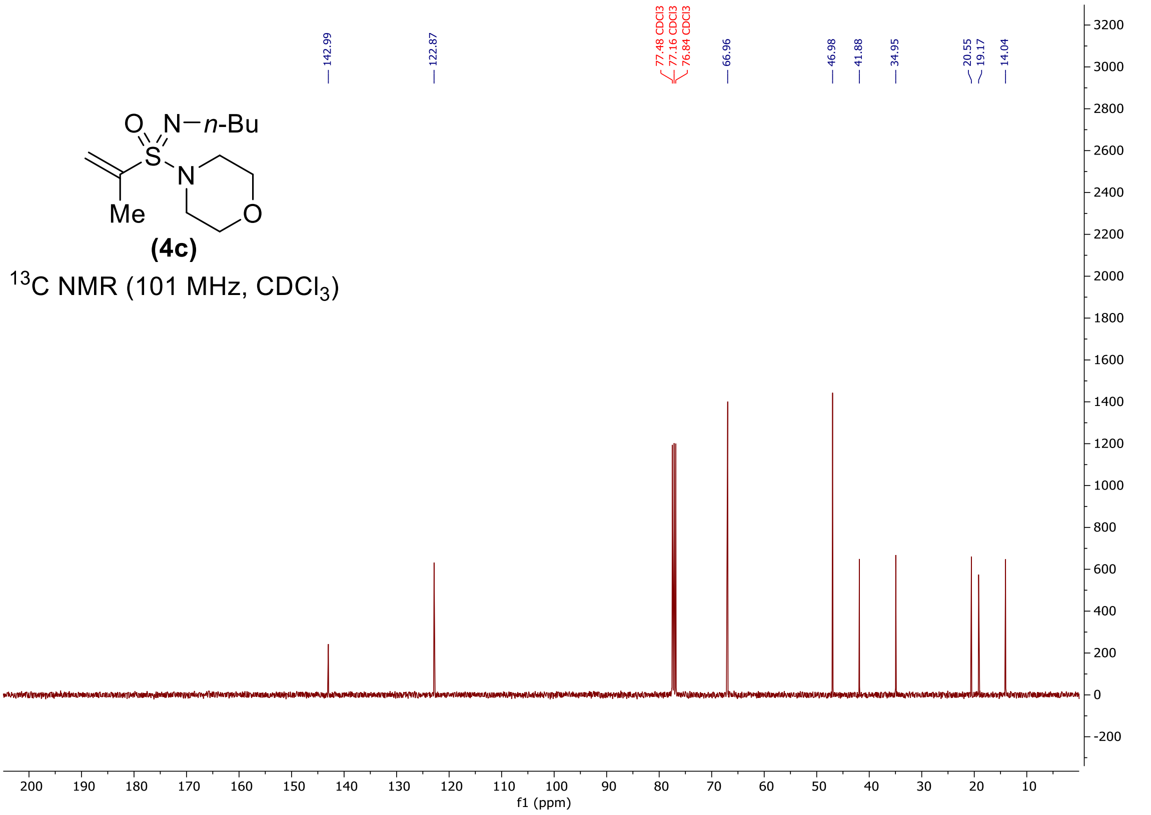

(*E*)-4-(prop-1-en-1-ylsulfonimidoyl)morpholine (*E*-2h)

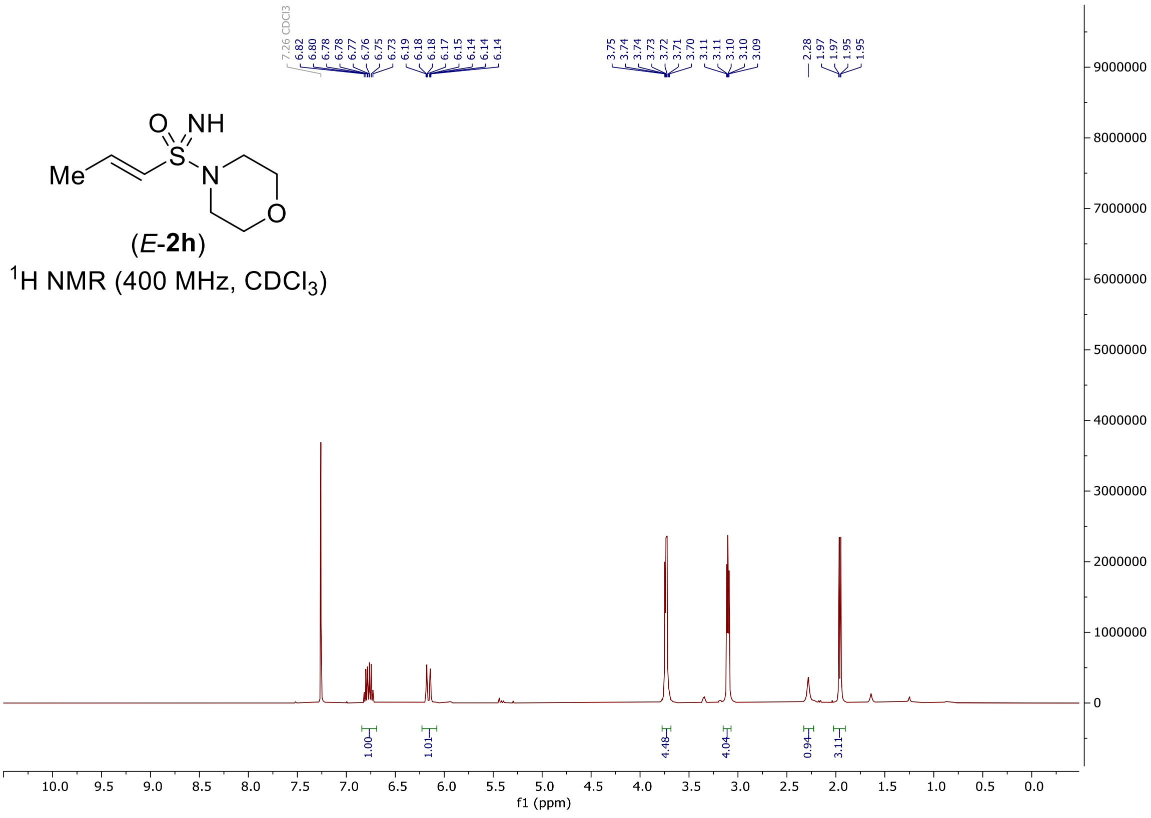

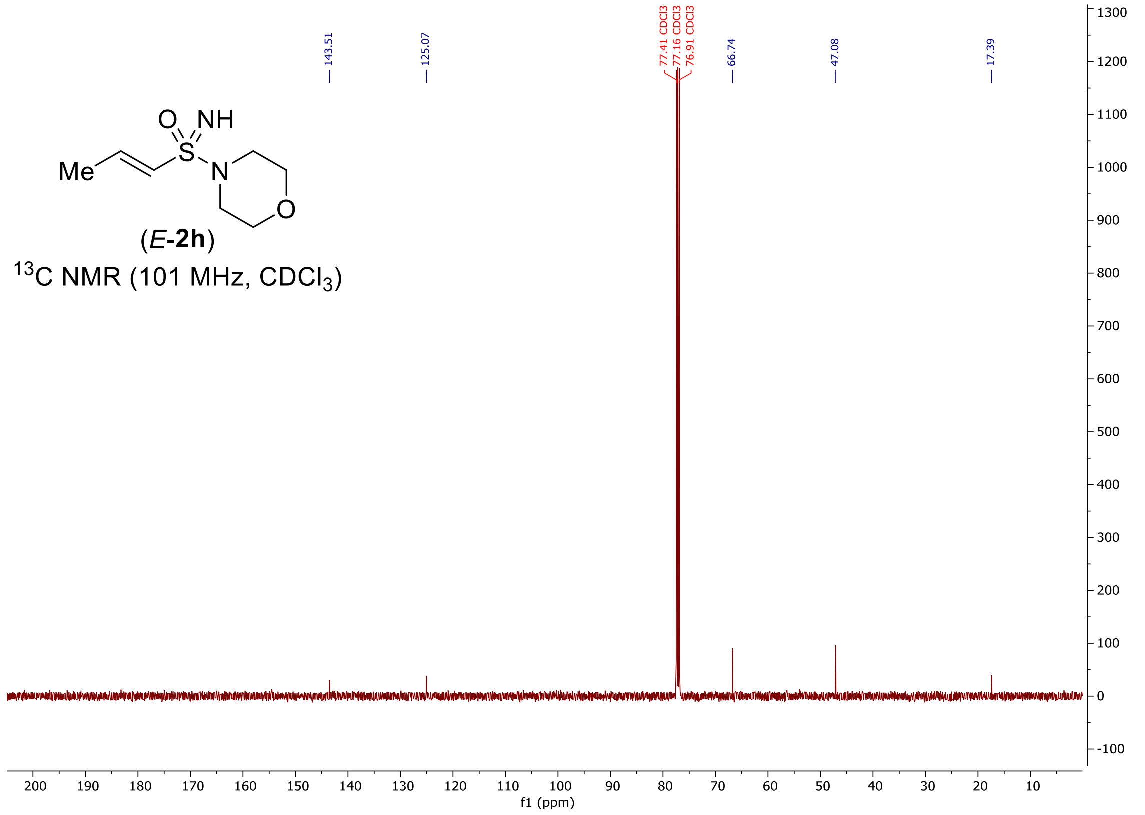

(*E*)-*N*-(morpholino(oxo)(prop-1-en-1-yl)-l6-sulfaneylidene)acetamide (4d)

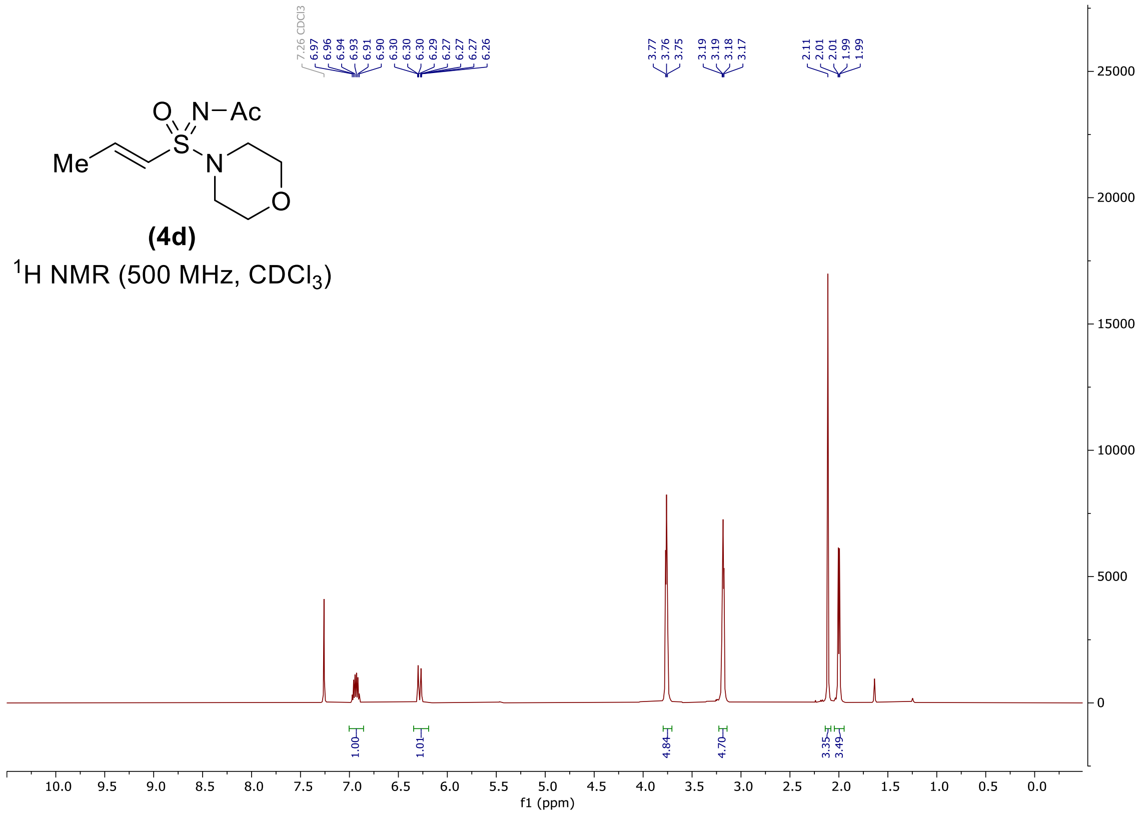

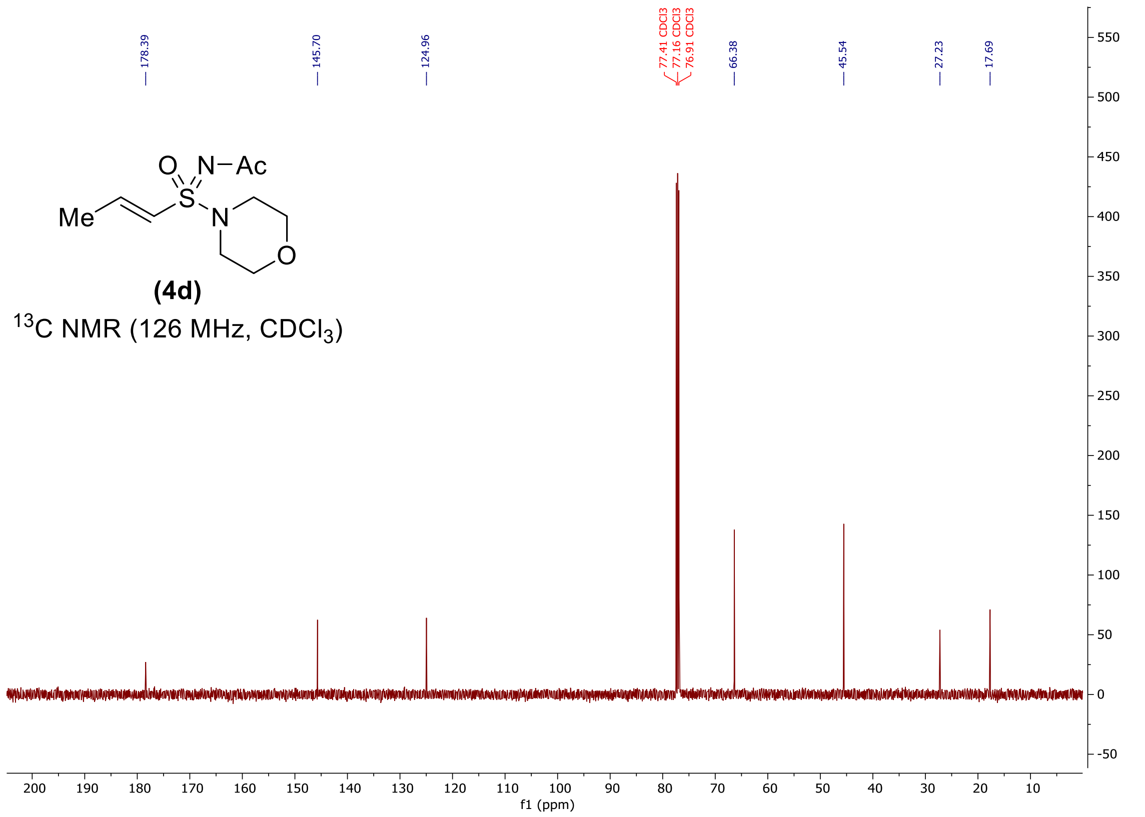

(*E*)-*N*-(morpholino(oxo)(prop-1-en-1-yl)-l6-sulfaneylidene)methanesulfonamide (4e)

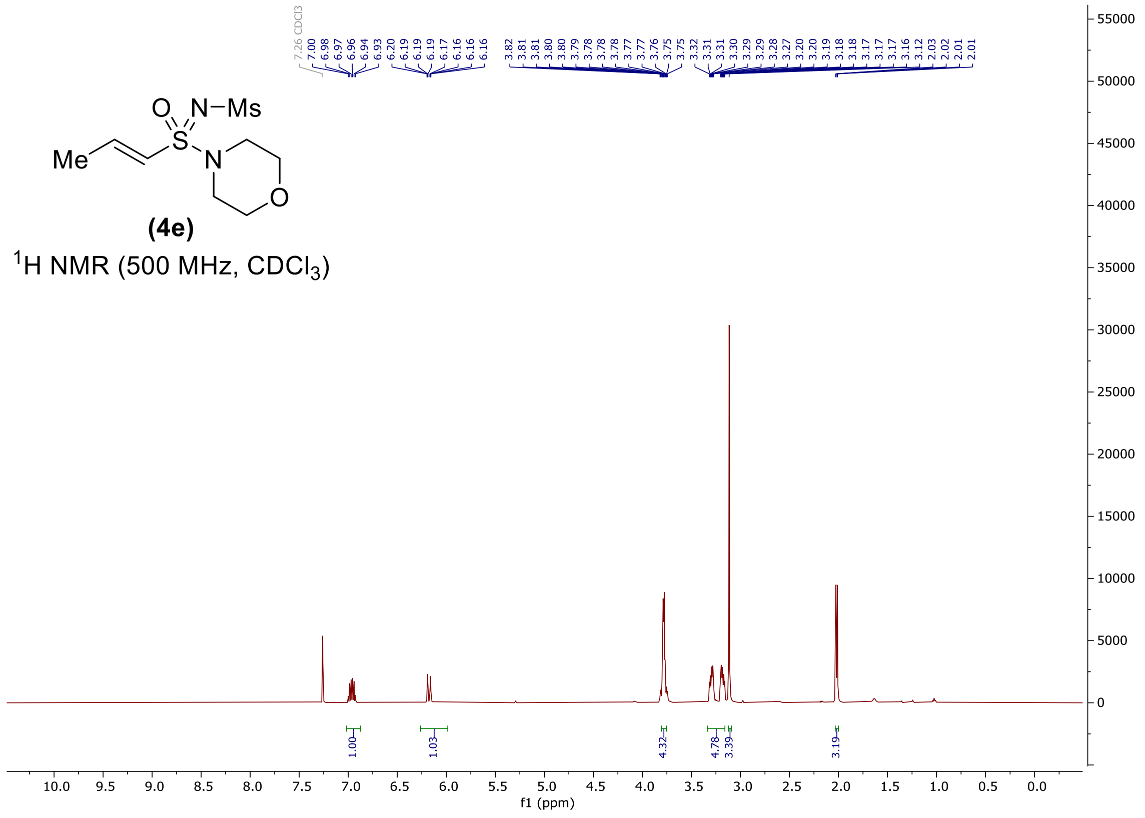

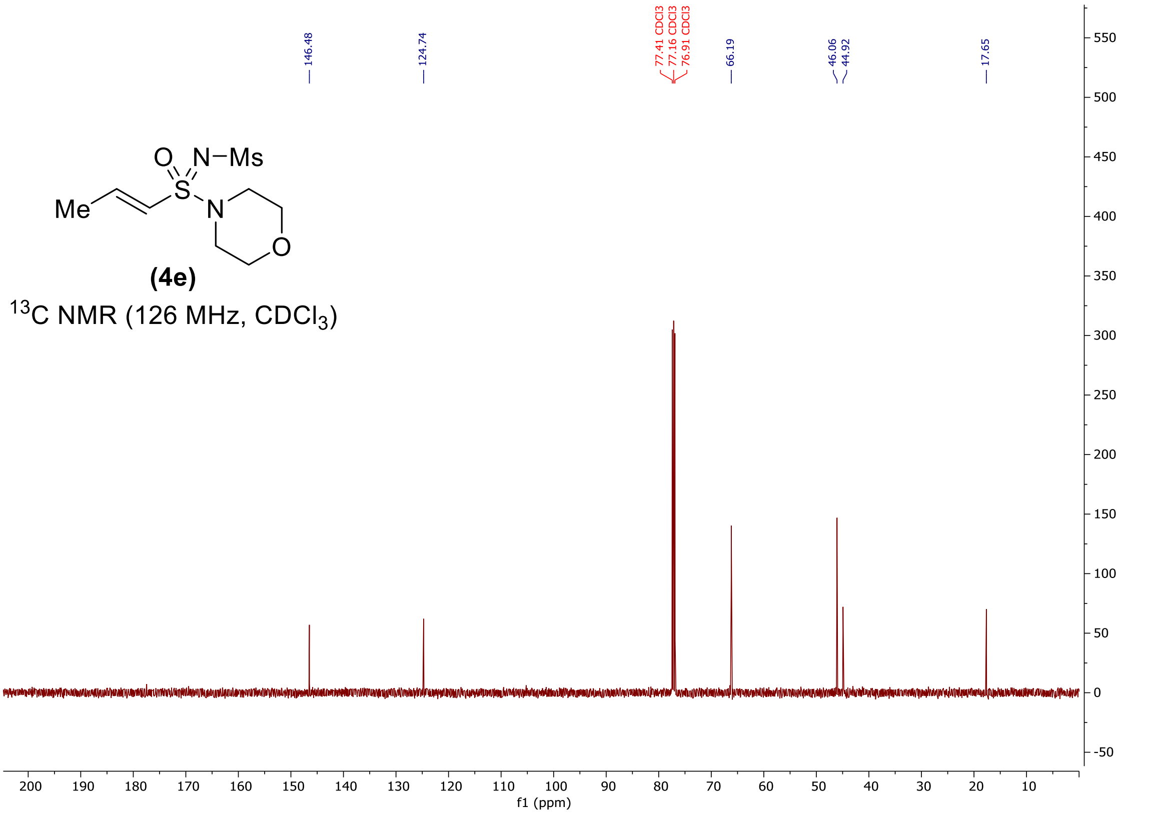

(*E*)-4-(*N*-butylprop-1-en-1-ylsulfonimidoyl)morpholine (4f)

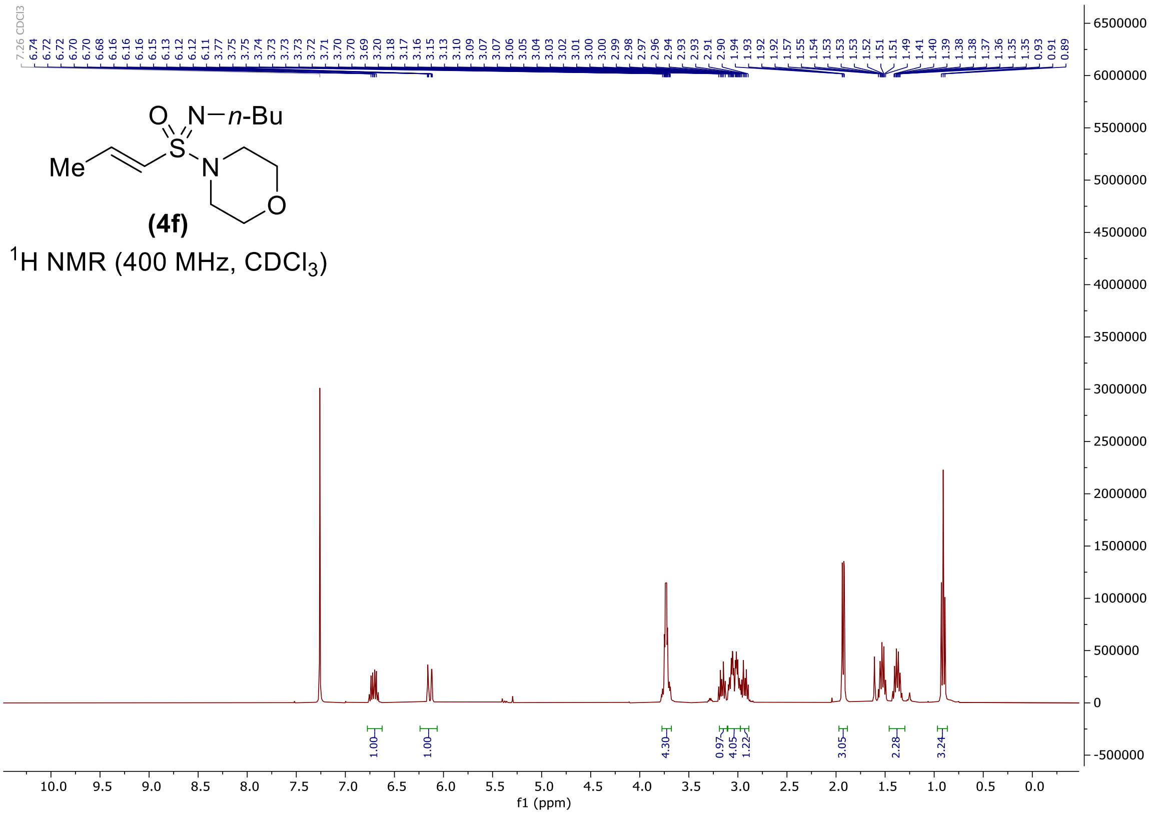

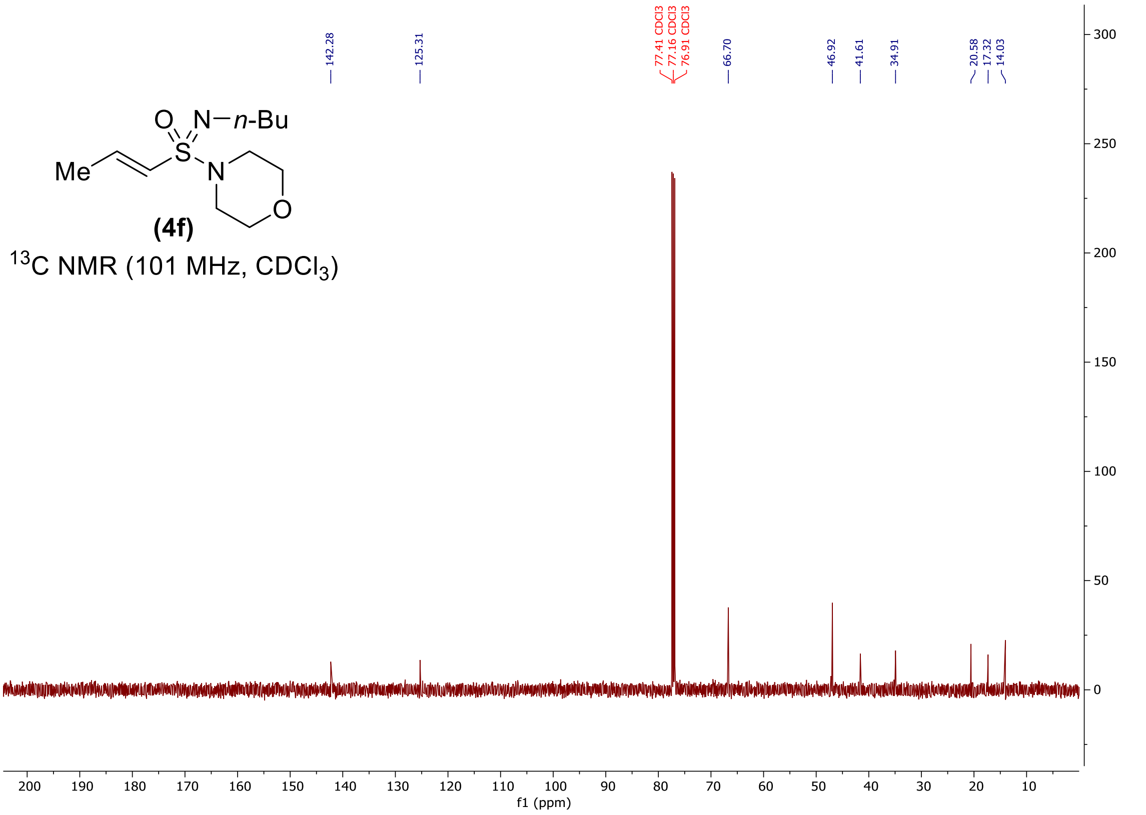

N-(((4-methoxyphenyl)(methyl)amino)(oxo)(vinyl)-l6-sulfaneylidene)methanesulfonamide (5a)

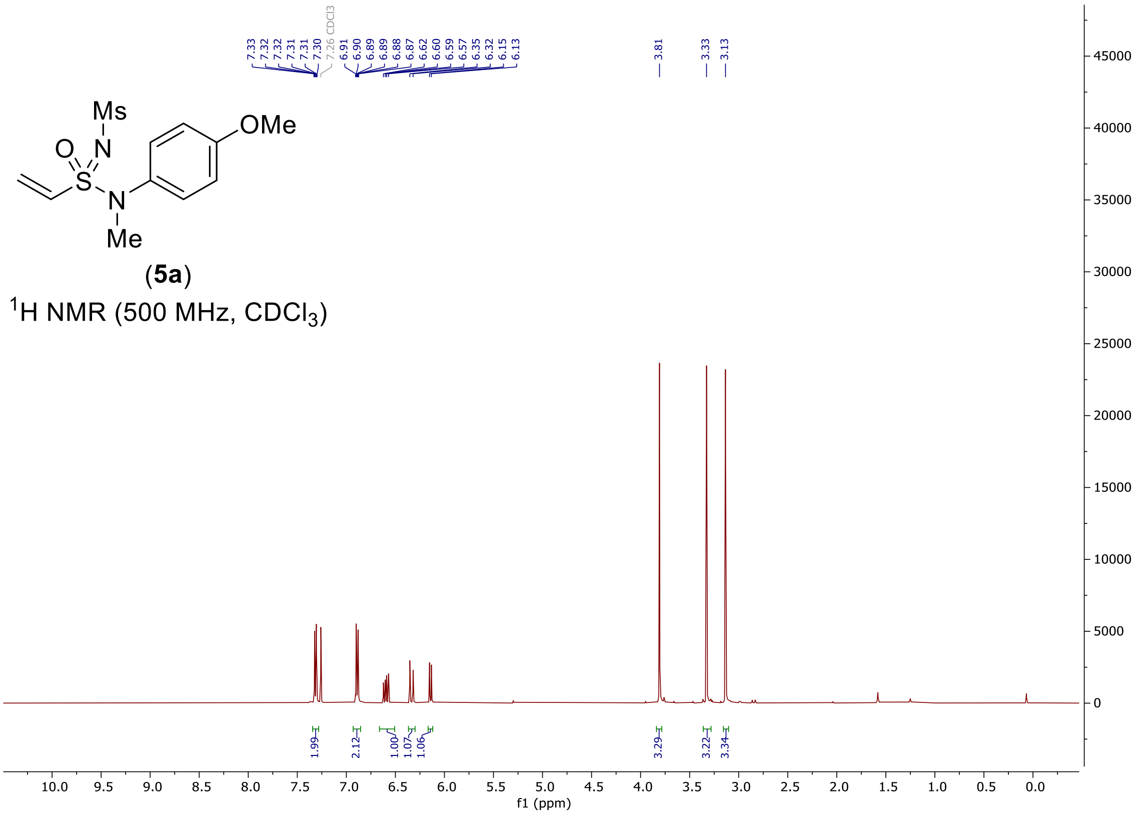

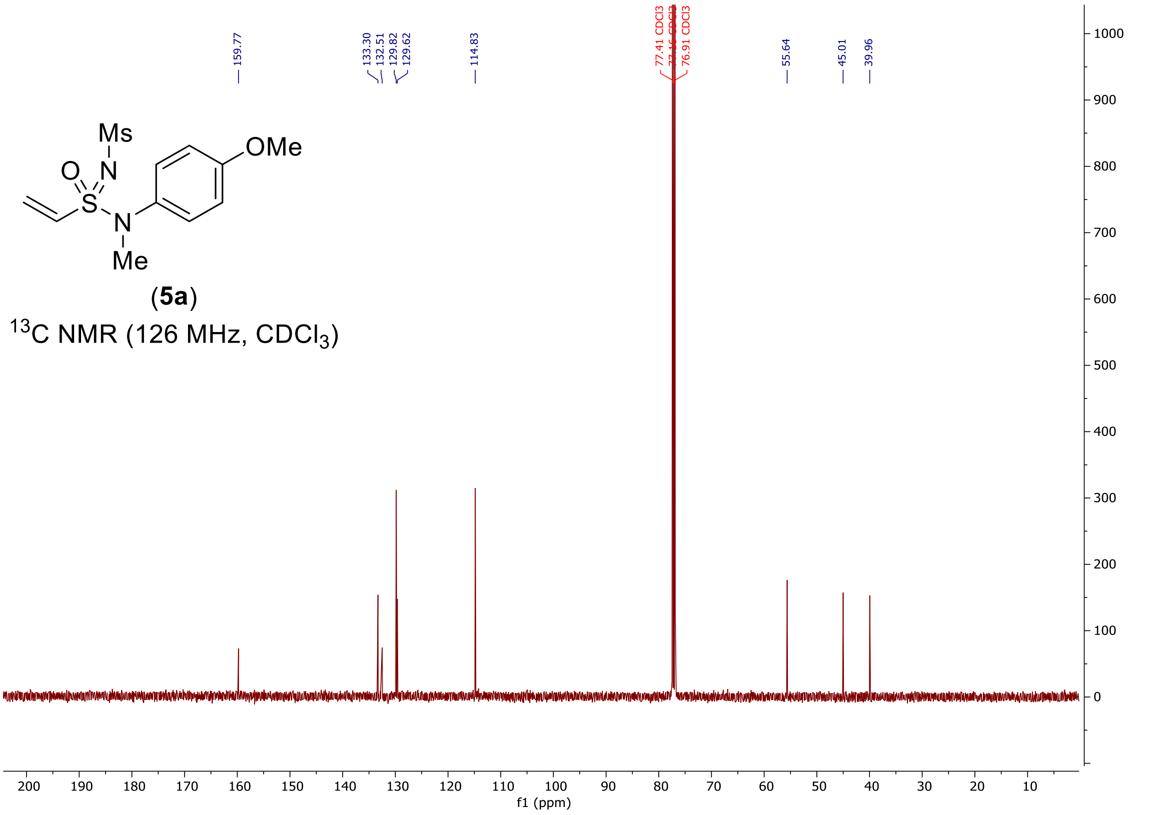

N-(((4-methoxyphenyl)(methyl)amino)(oxo)(vinyl)-l6-sulfaneylidene)acetamide (5b)

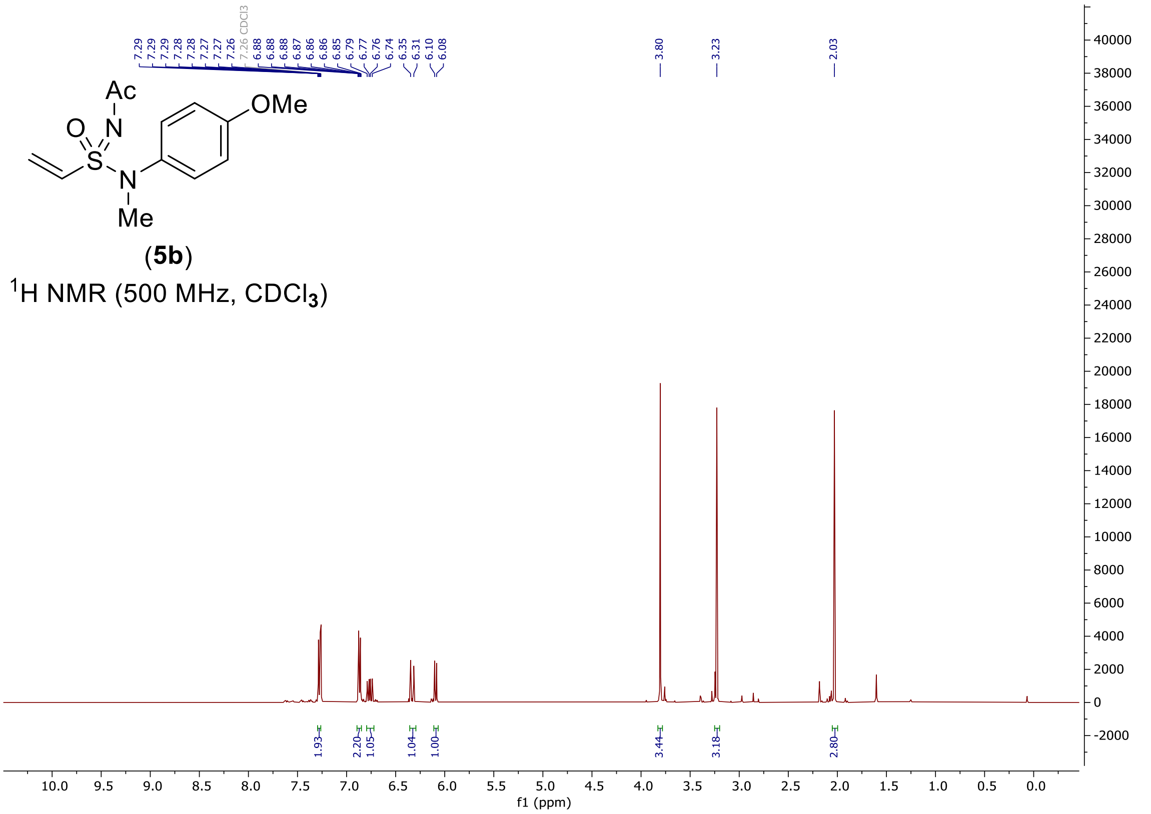

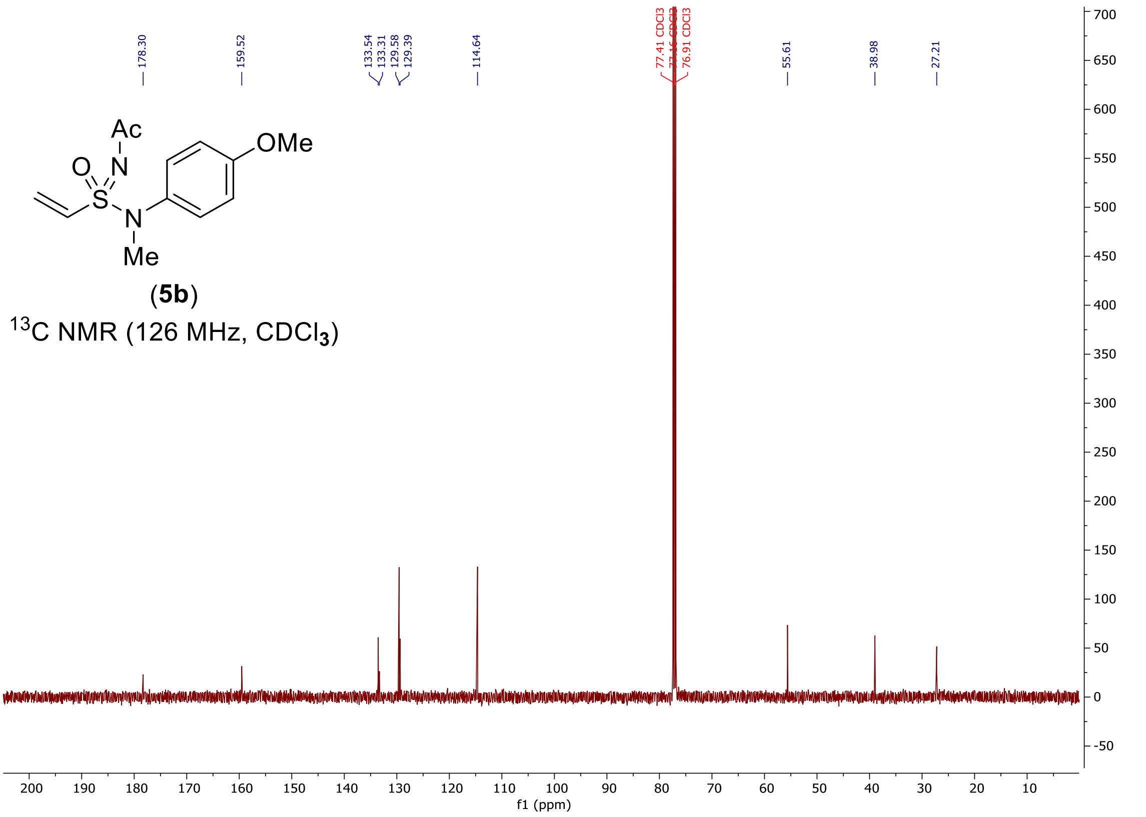

N'-ethyl-*N*-(4-methoxyphenyl)-N-methylethenesulfonimidamide (5c)

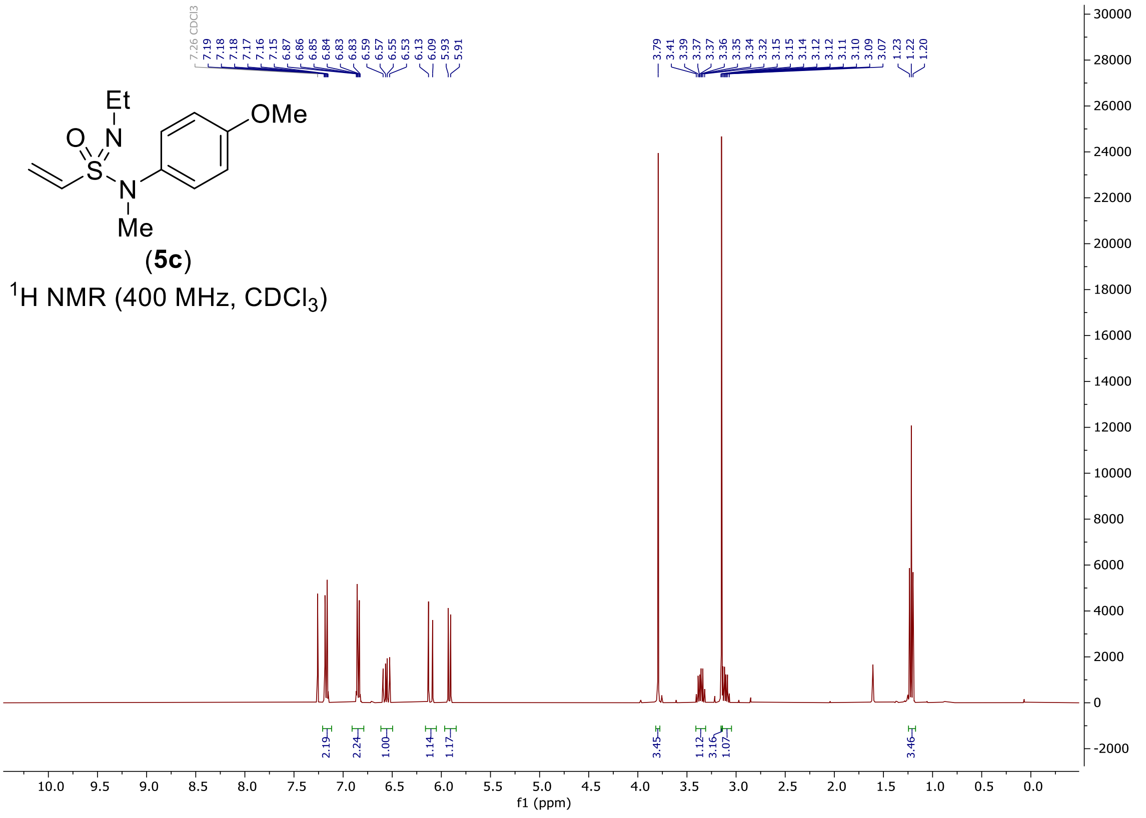

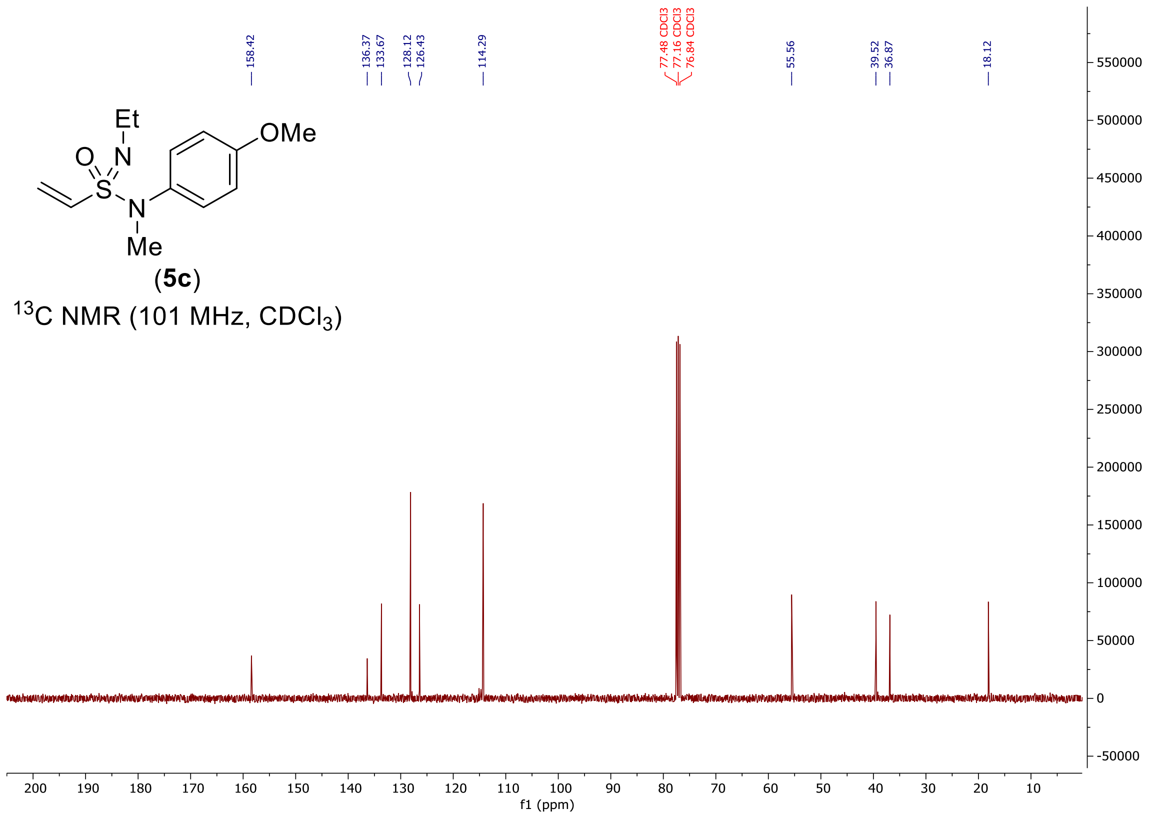

***N*-(*N*-(4-methoxyphenyl)vinylsulfonimidoyl)methanesulfonamide (6a)**

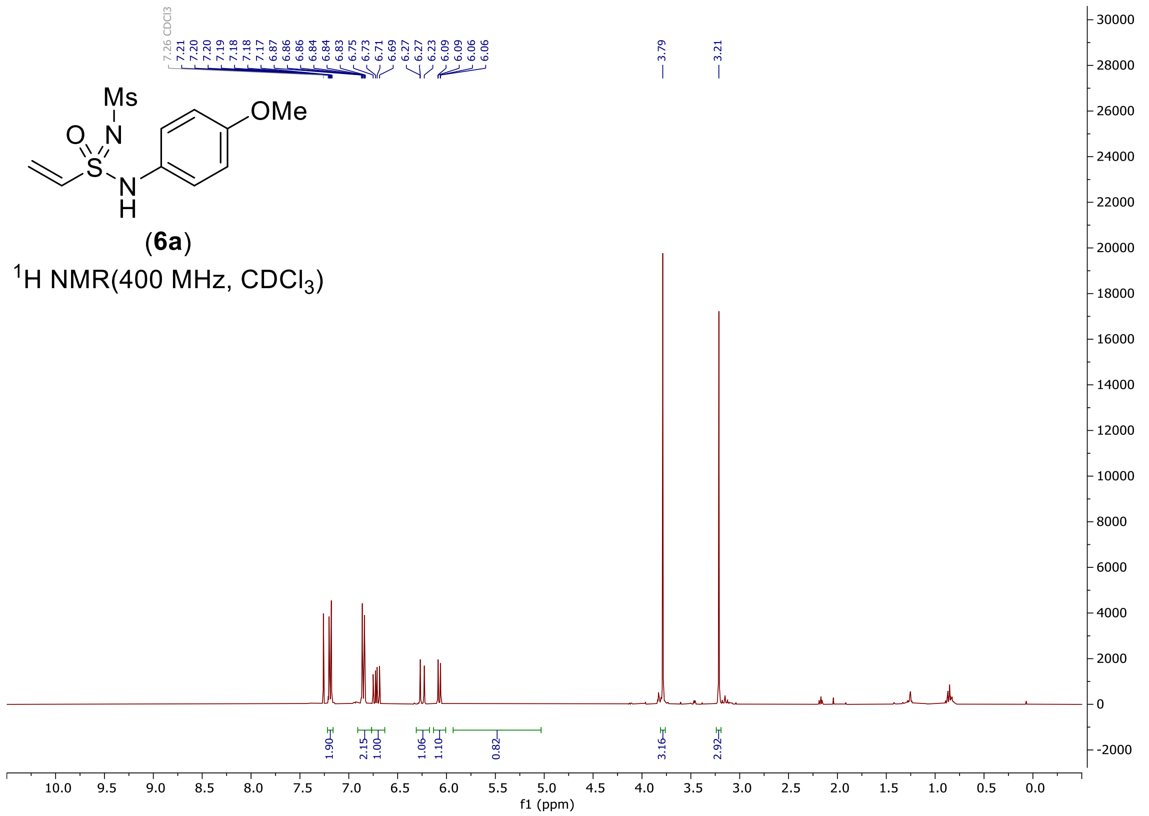

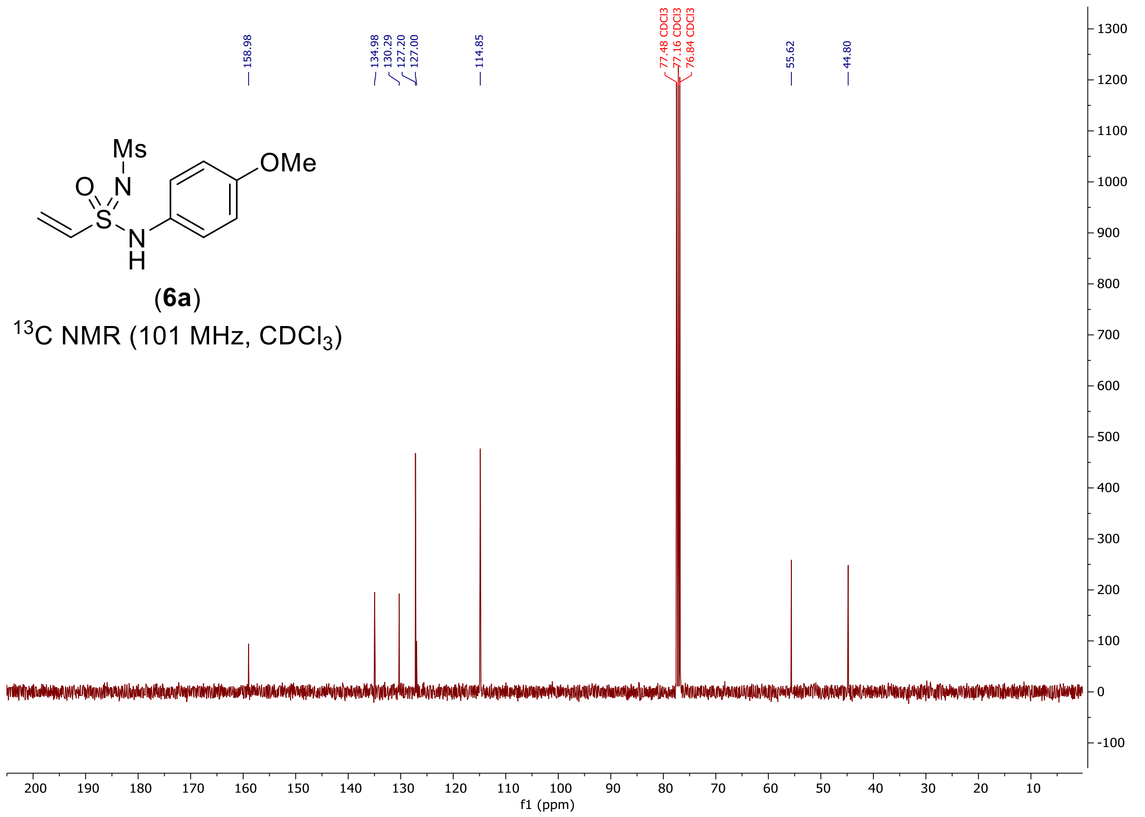

N-(*N*-(4-methoxyphenyl)vinylsulfonimidoyl)acetamide (6b)

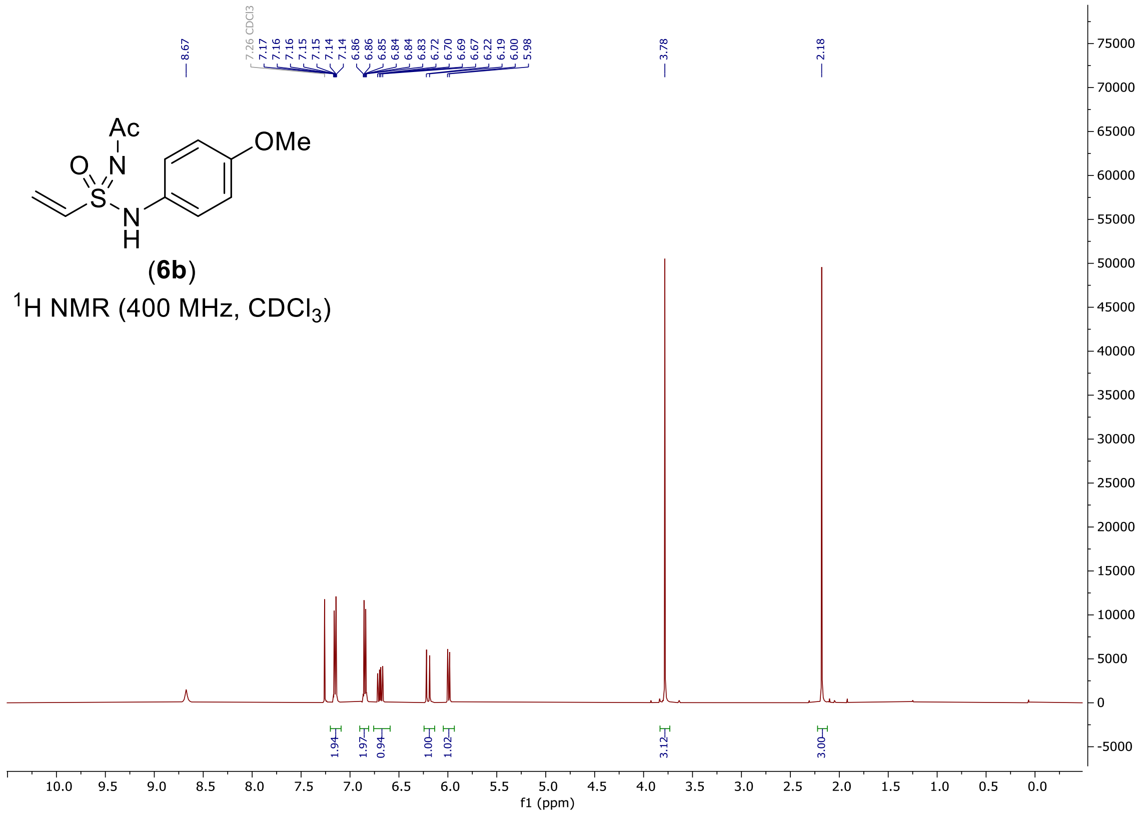

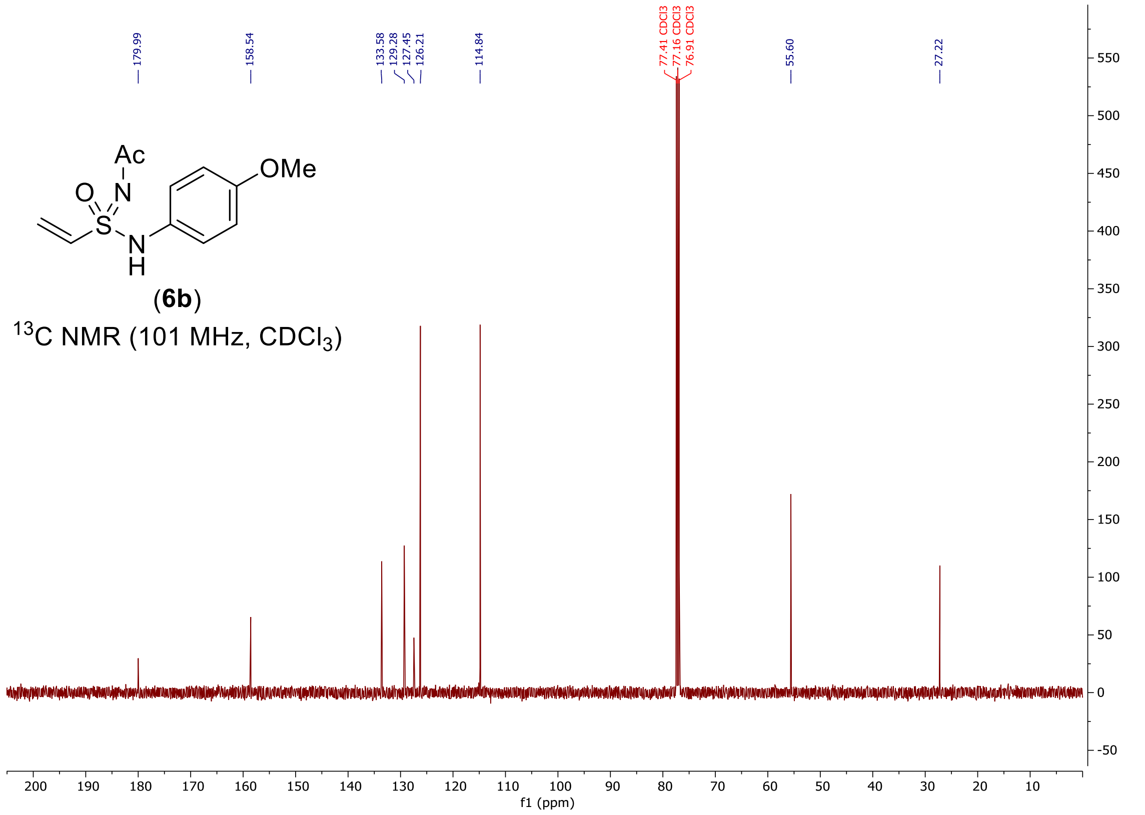

N-(*N*-(4-methoxyphenyl)vinylsulfonimidoyl)butyramide (6c)

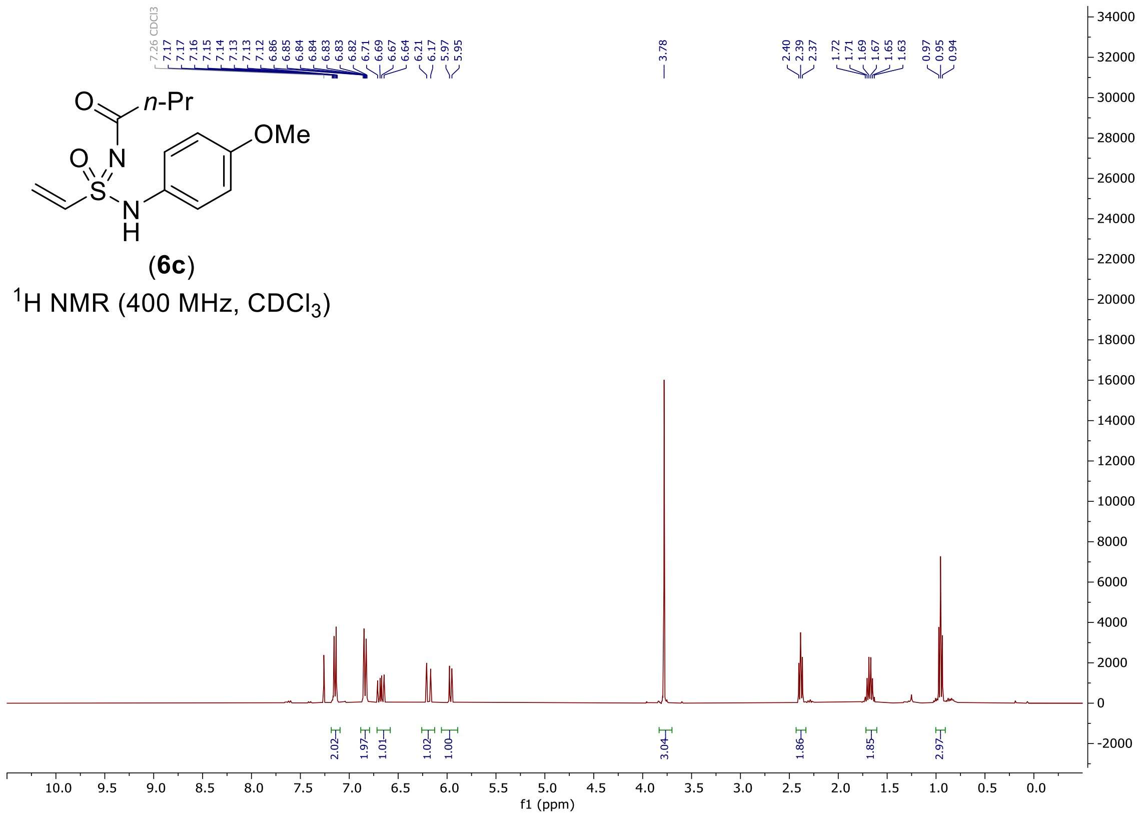

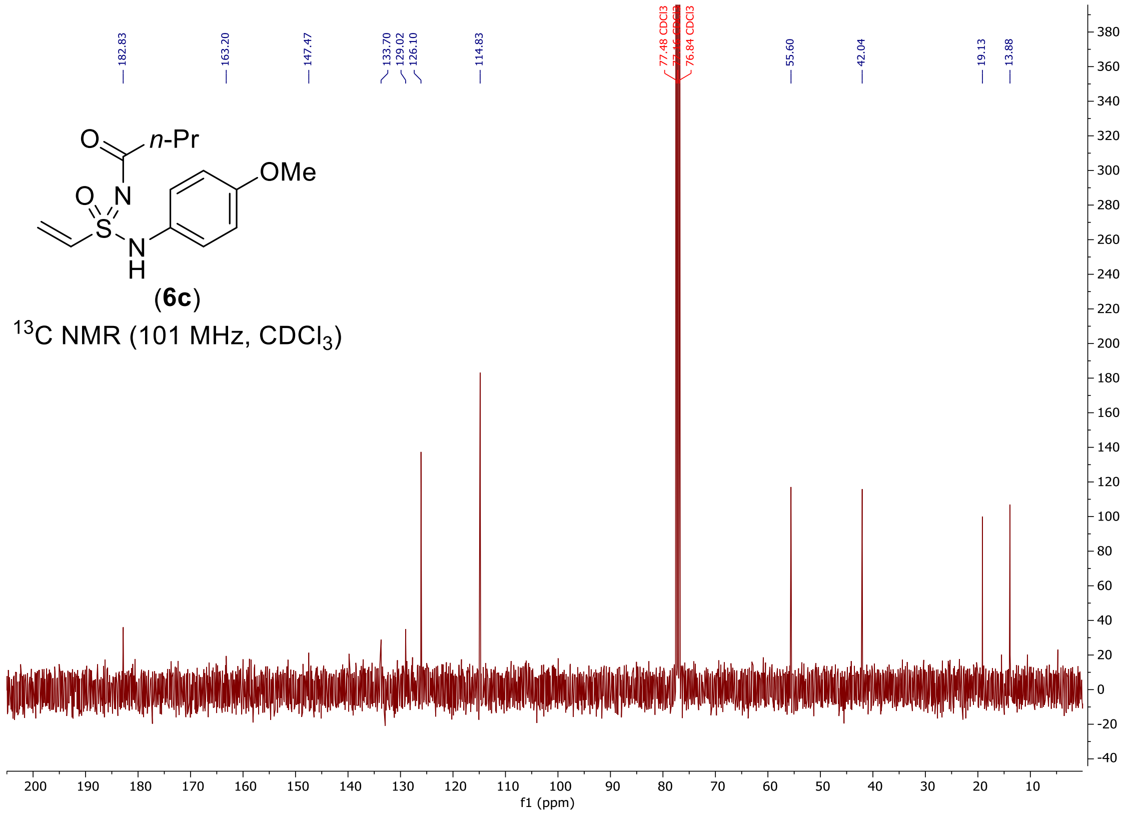

N-ethyl-N'-(4-methoxyphenyl)ethenesulfonimidamide (6d)

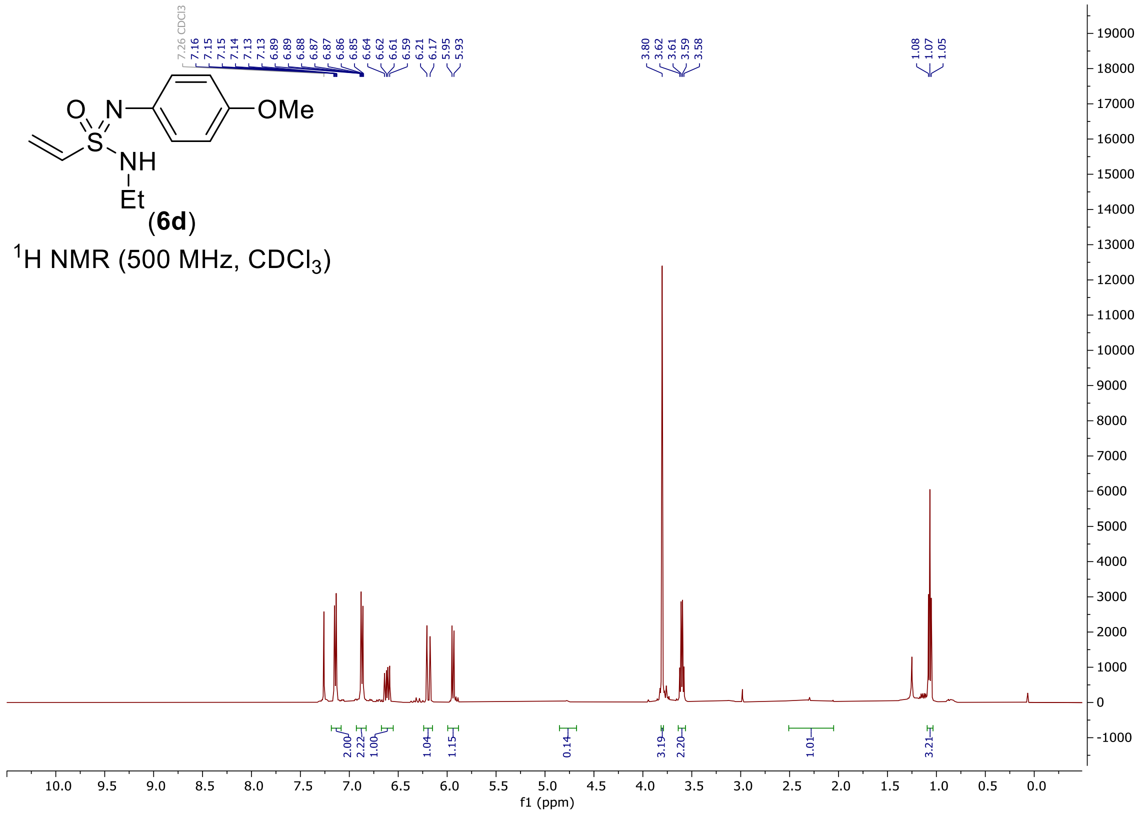

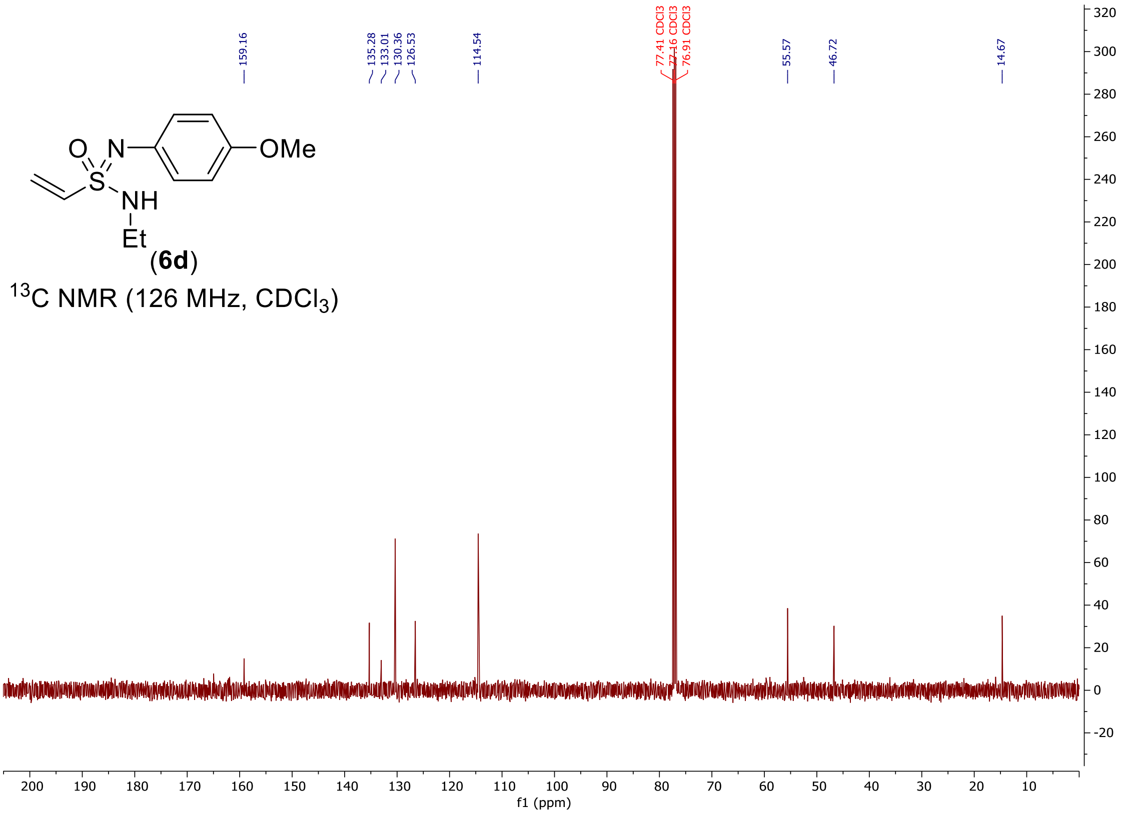

N-(*N*-(4-Ethynylphenyl)vinylsulfonimidoyl)acetamide (6e)

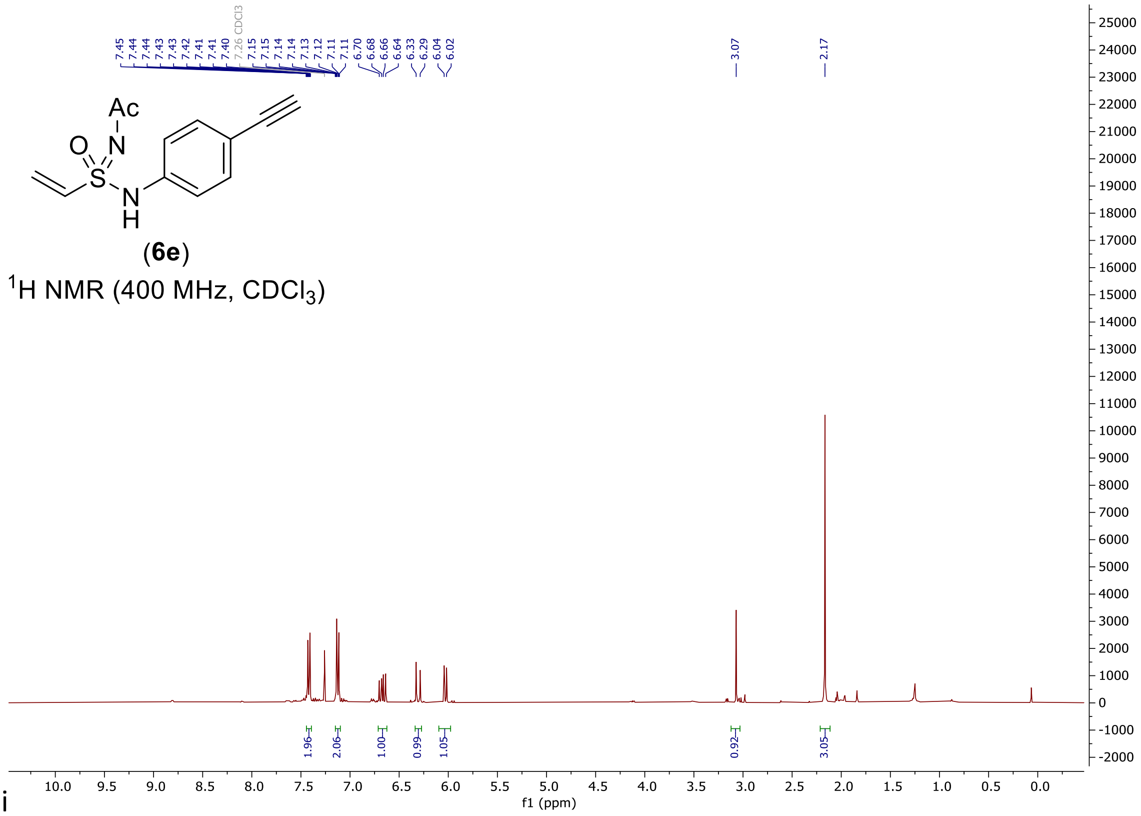

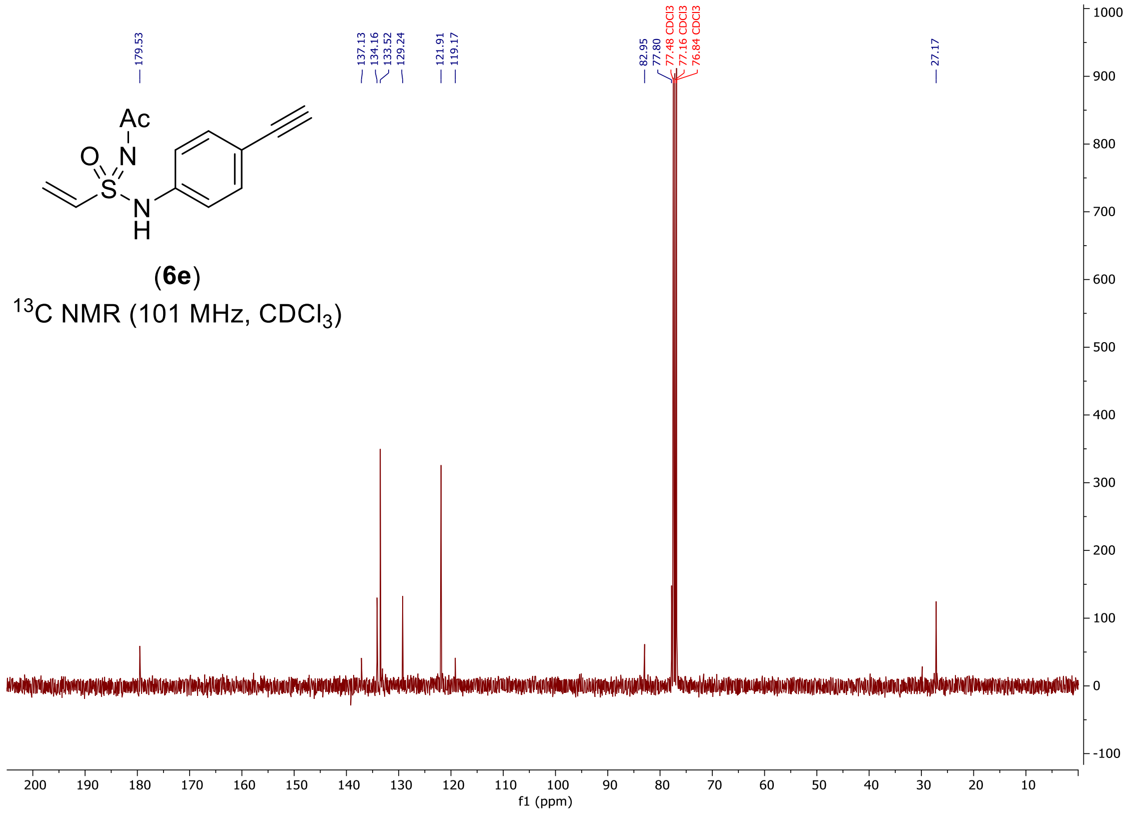

N-(*N*-(4-Ethynylphenyl)vinylsulfonimidoyl)butyramide (6f)

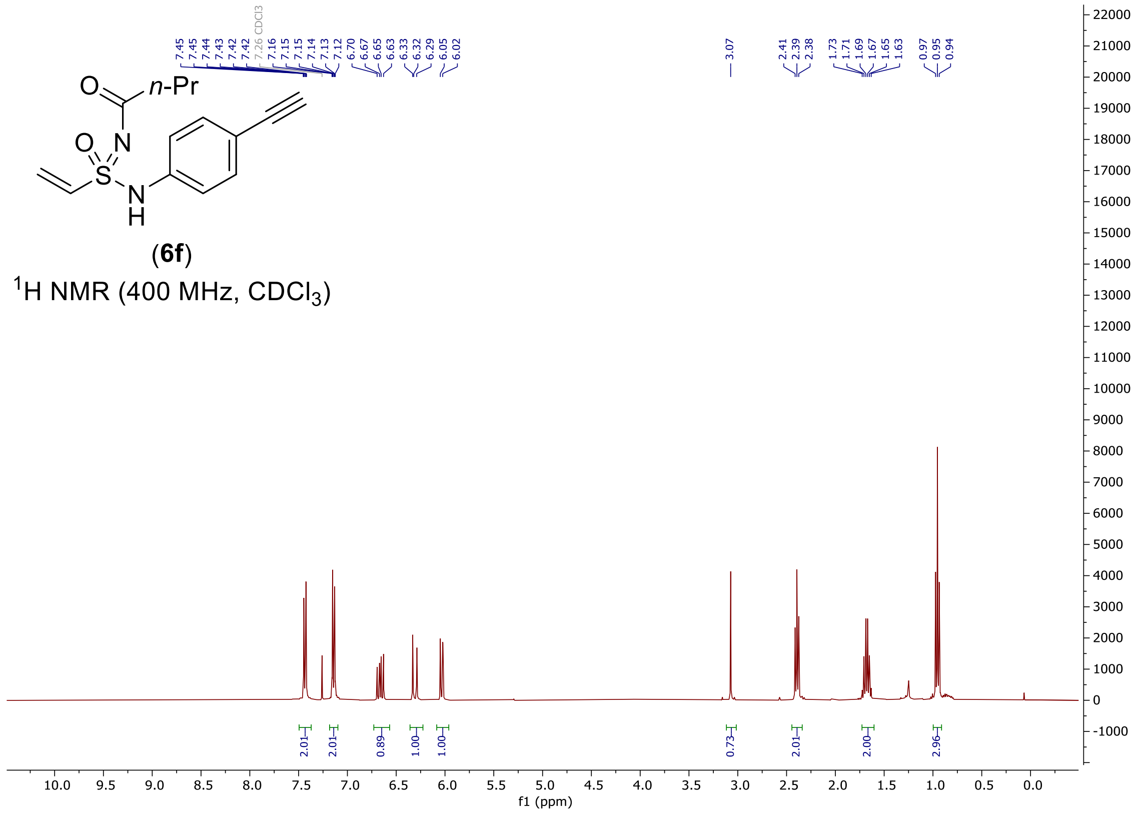

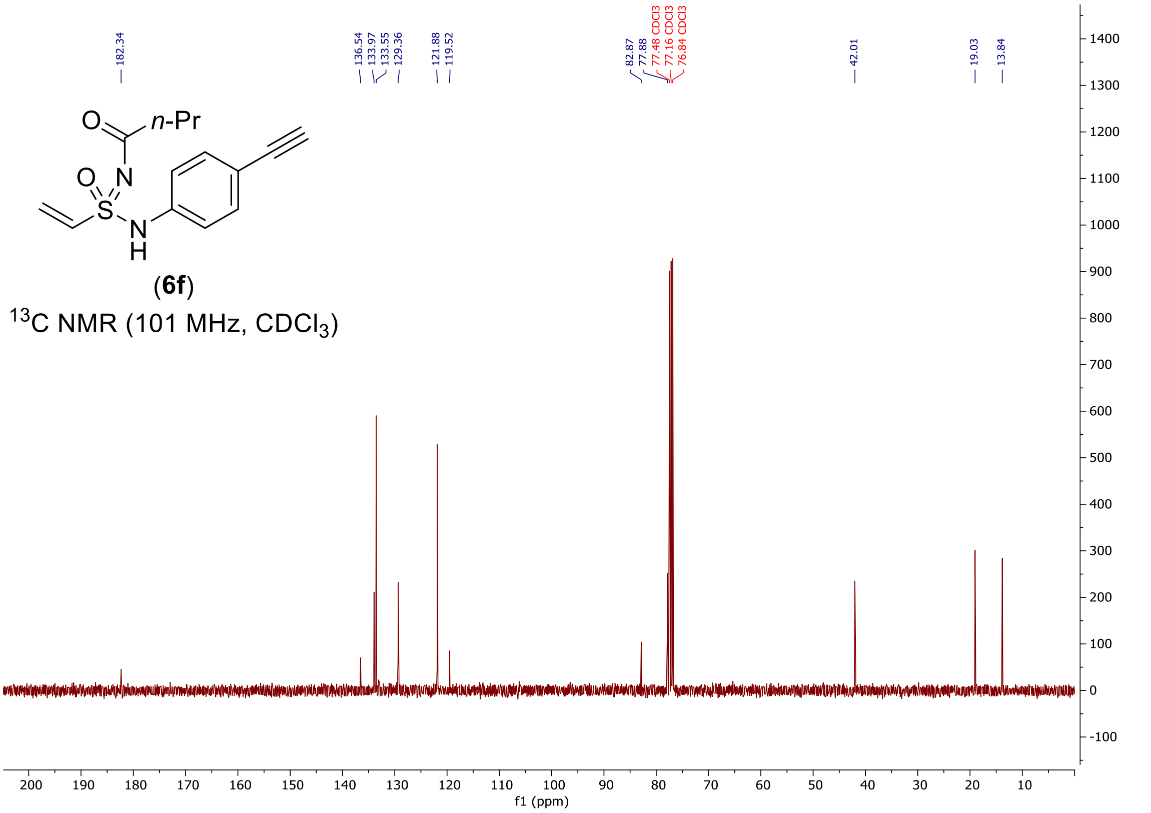

N-(*N*-(4-Ethynylphenyl)vinylsulfonimidoyl)methanesulfonamide (6g)

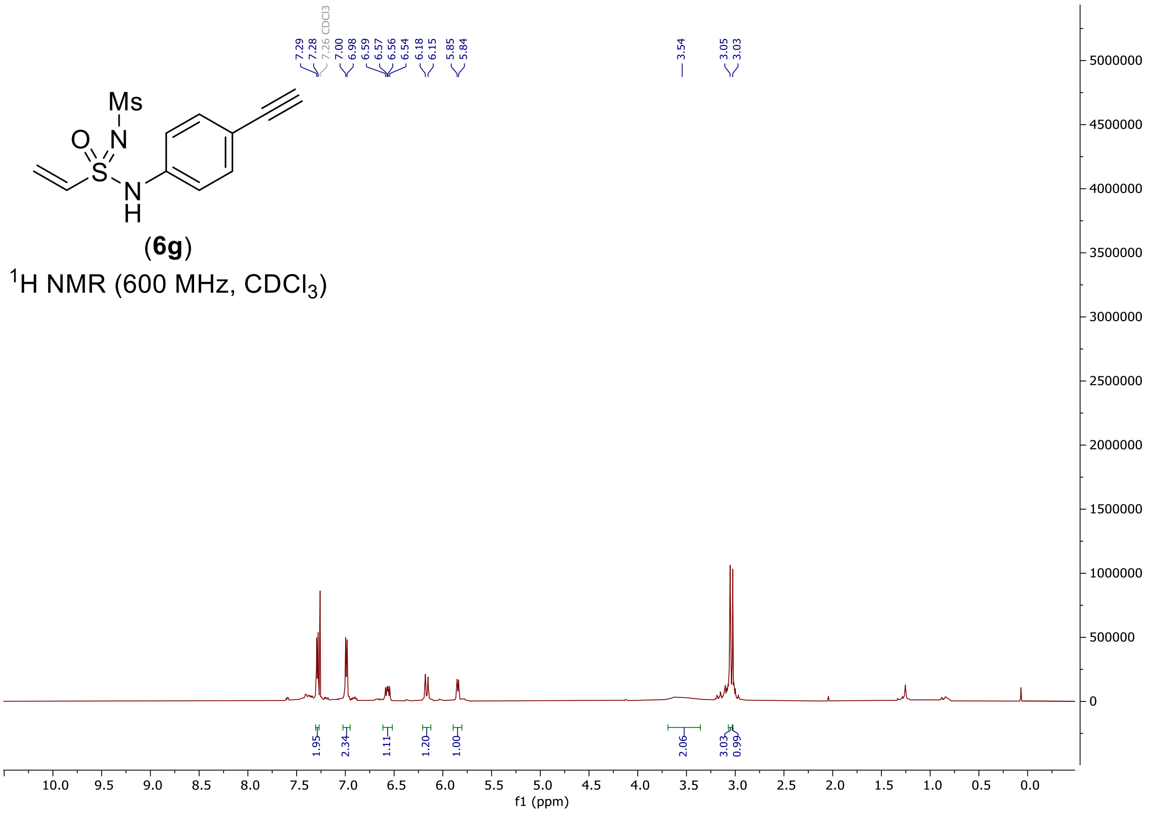

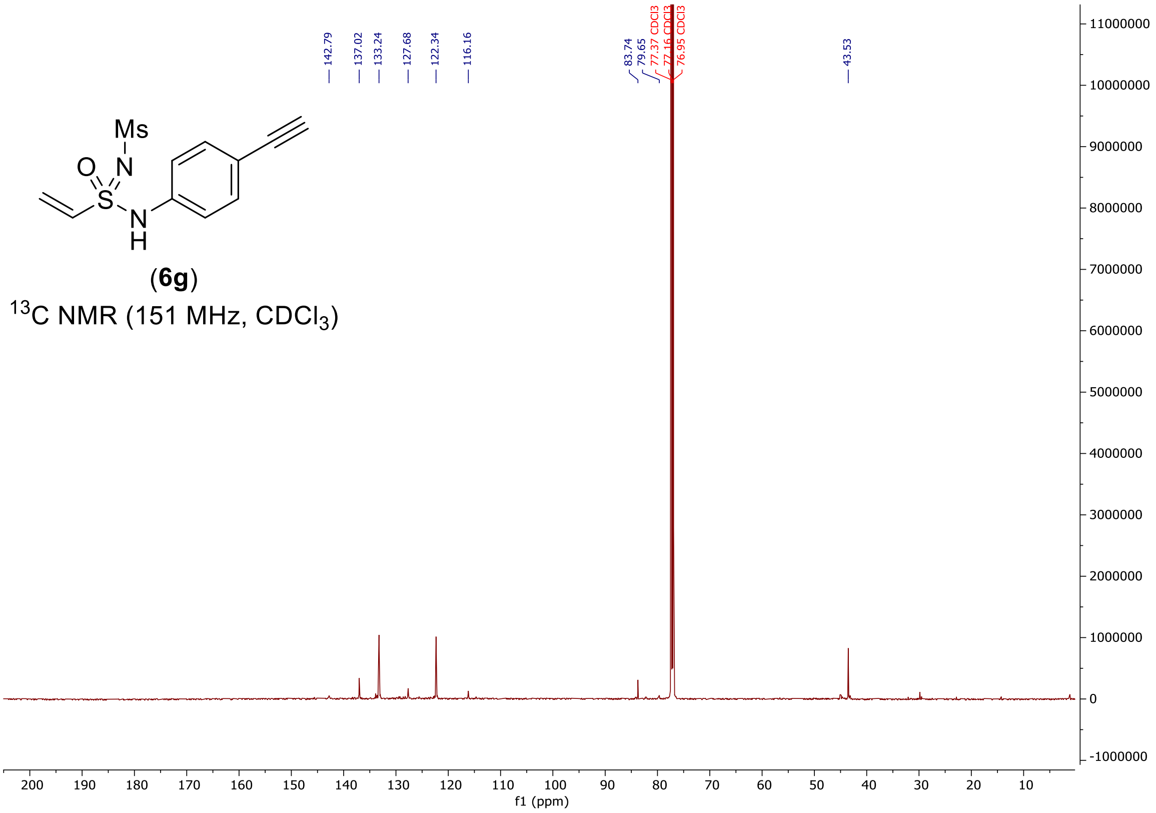

4-(2-(dodecylthio)ethylsulfonimidoyl)morpholine (7a)

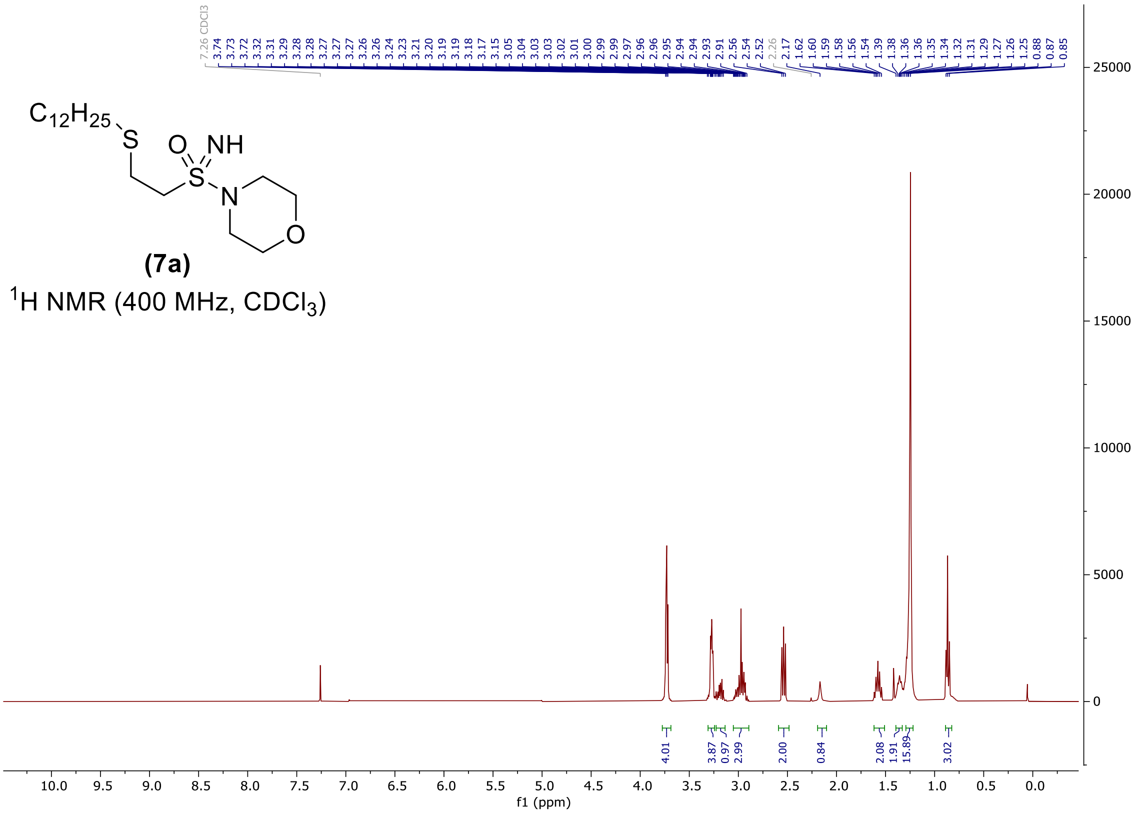

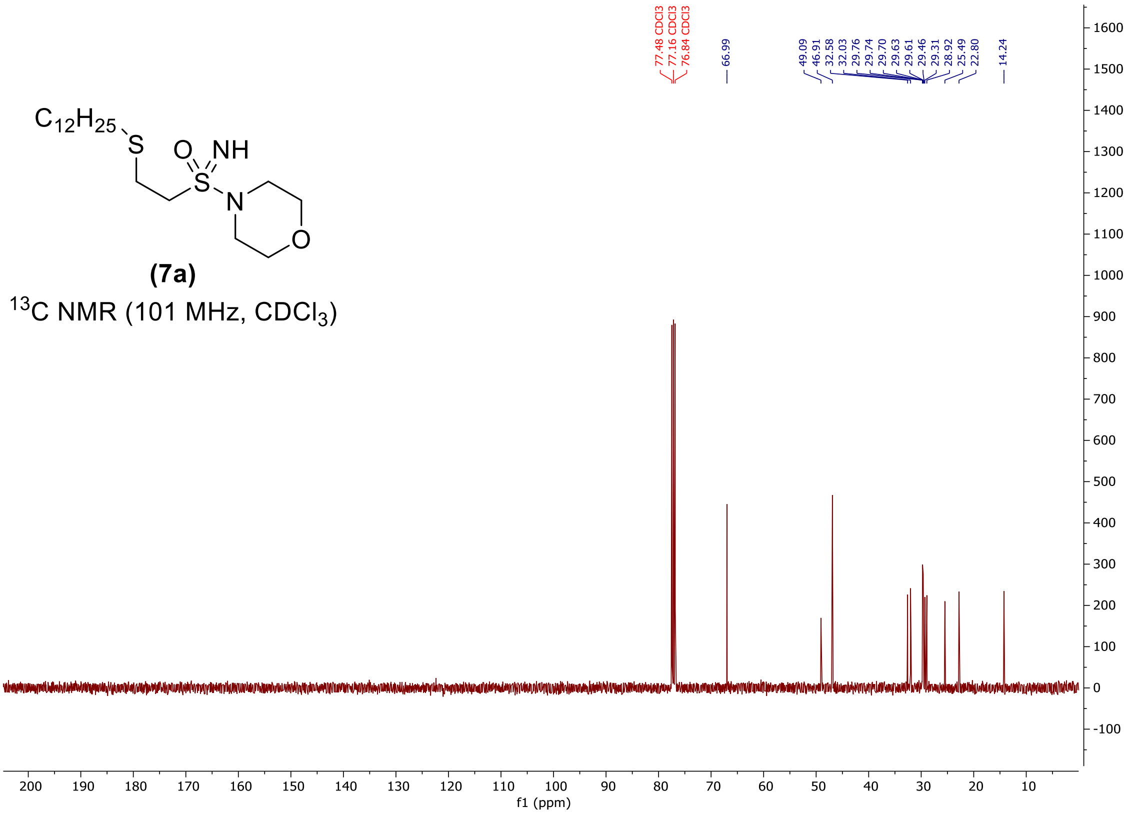

N-((2-(dodecylthio)ethyl)(morpholino)(oxo)- λ⁶-sulfaneylidene)acetamide (7b)

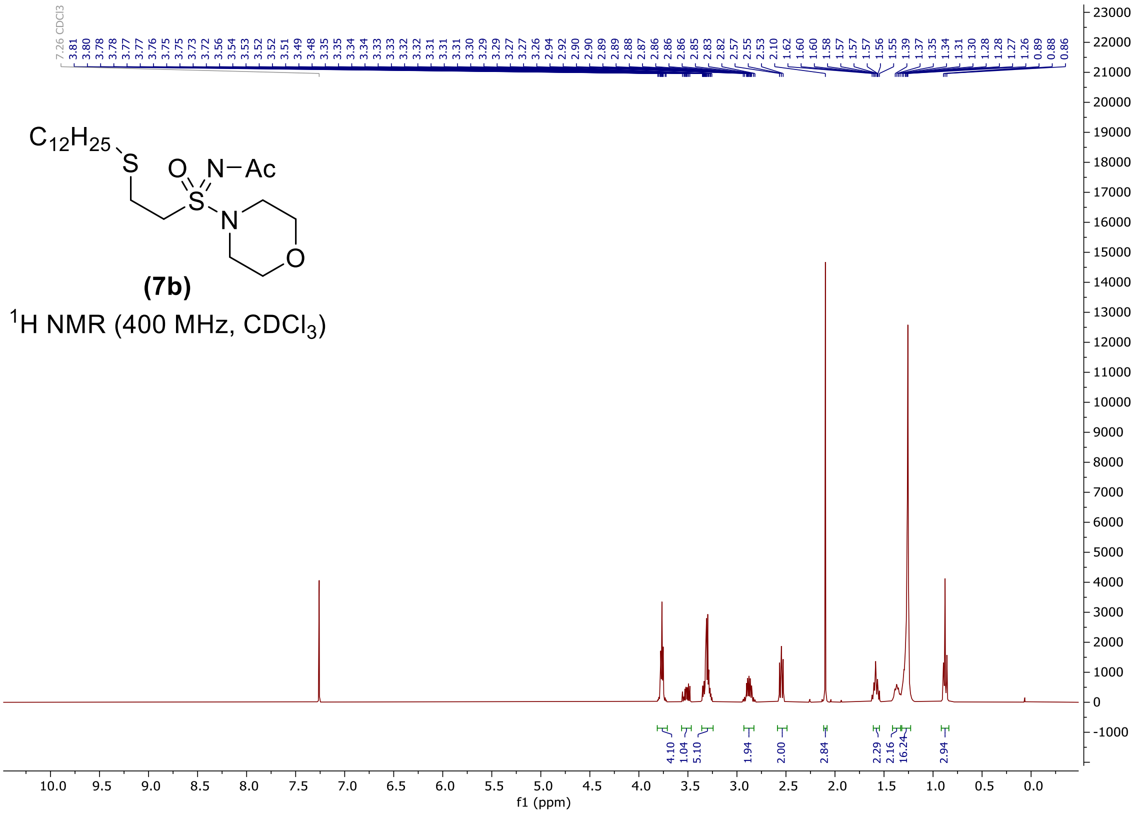

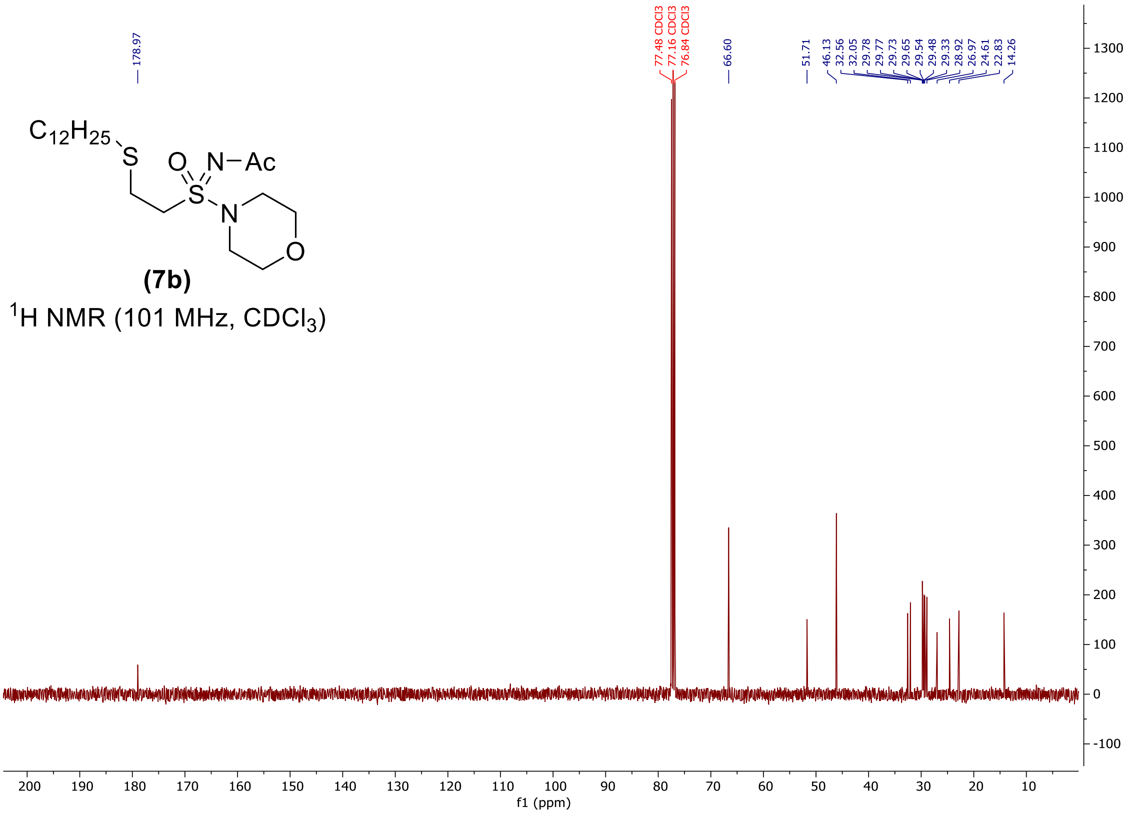

N-((2-(dodecylthio)ethyl)(morpholino)(oxo)- λ^6-sulfaneylidene)methanesulfonamide (7c)

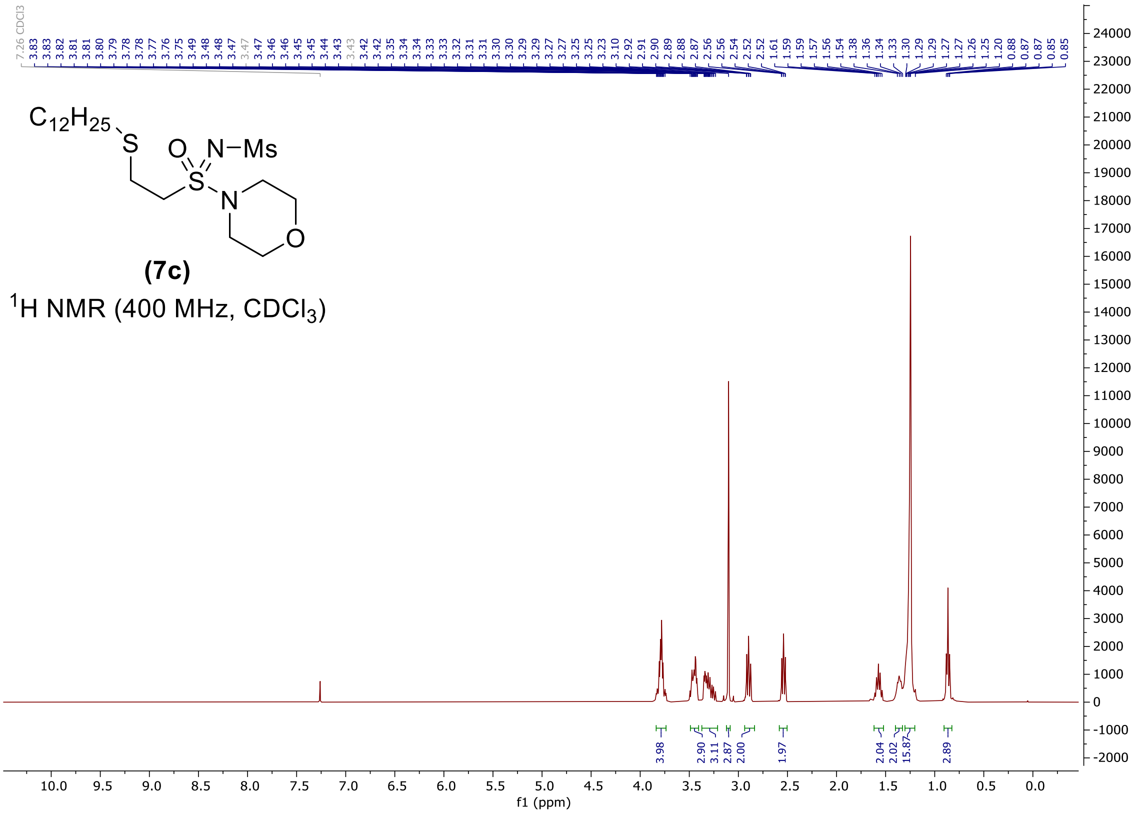

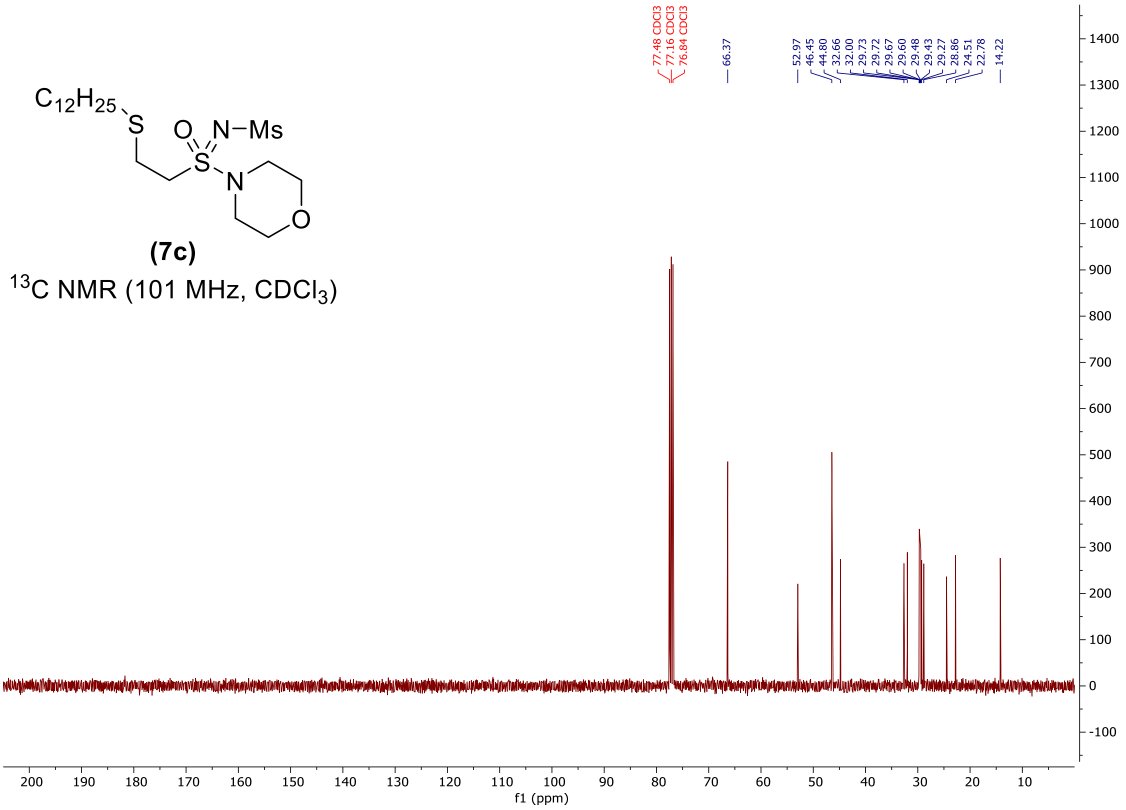

1-((2-(dodecylthio)ethyl)(morpholino)(oxo)-l6-sulfaneylidene)-3-(p-tolyl)urea (7d)

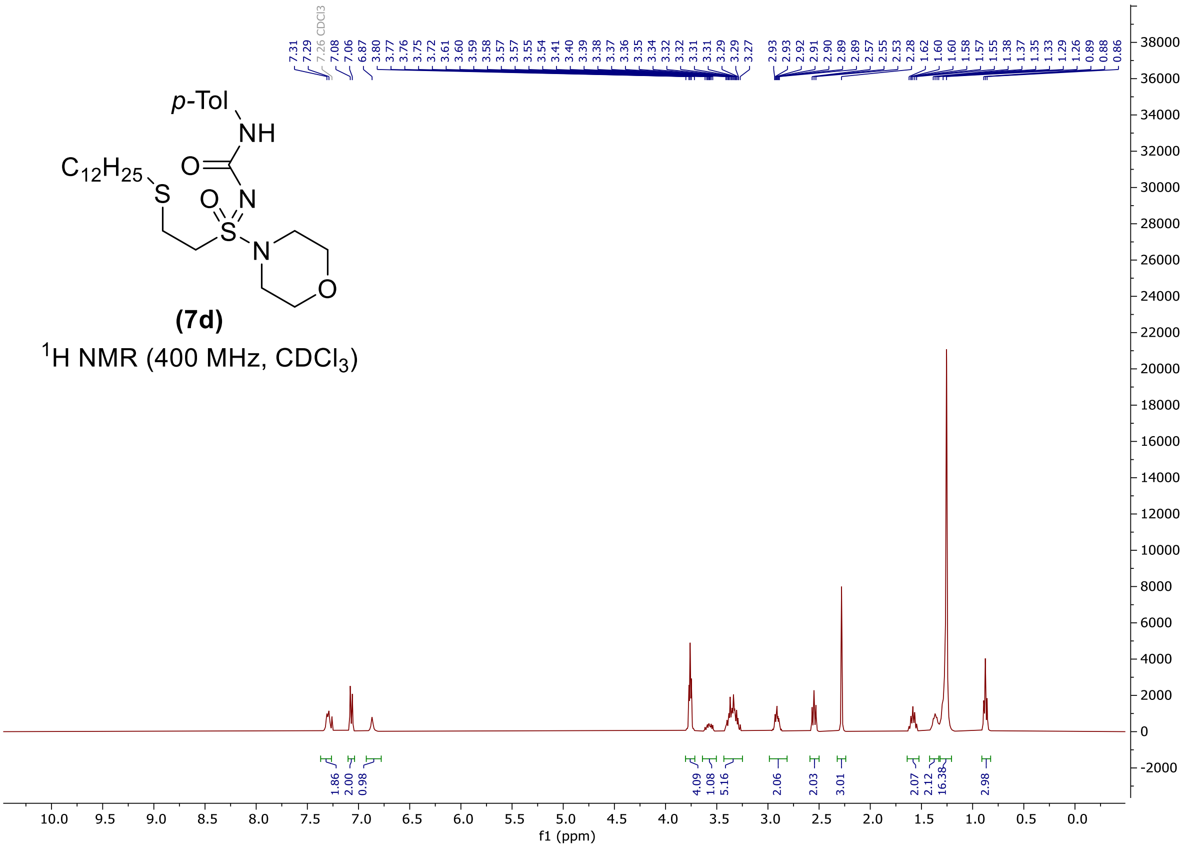

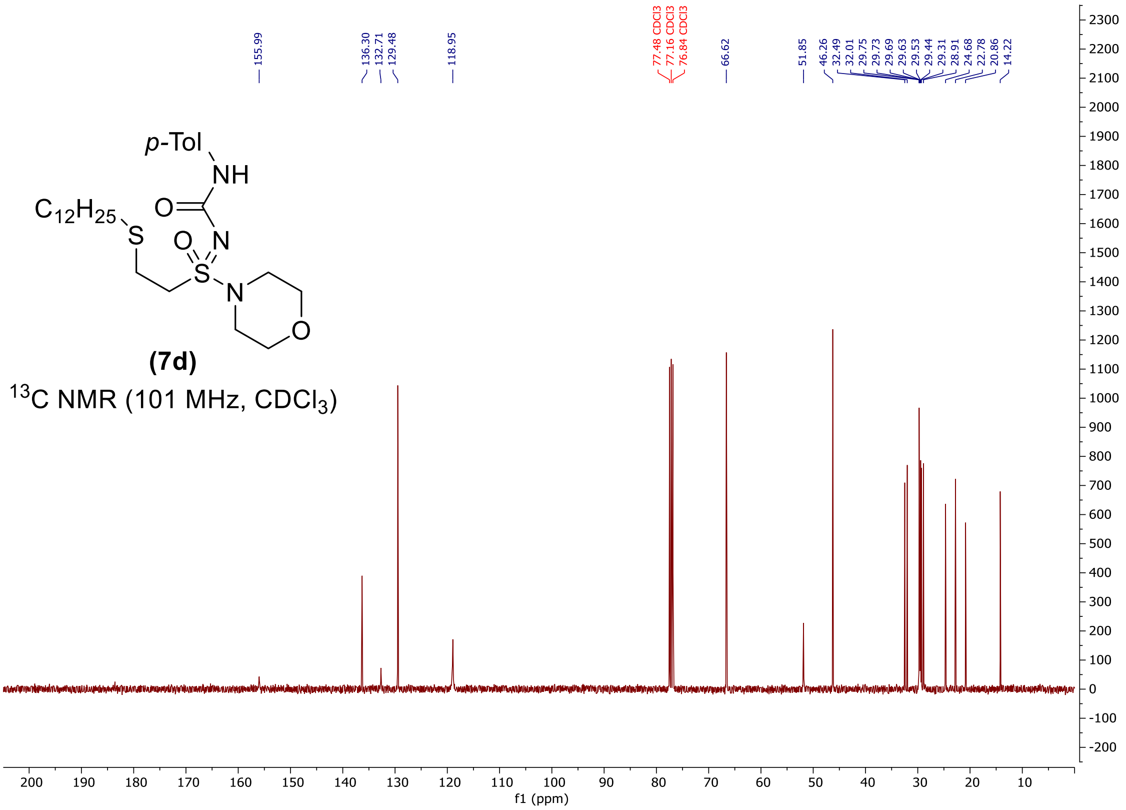

4-(2-(dodecylthio)-*N*-(p-tolyl)ethylsulfonimidoyl)morpholine (7e)

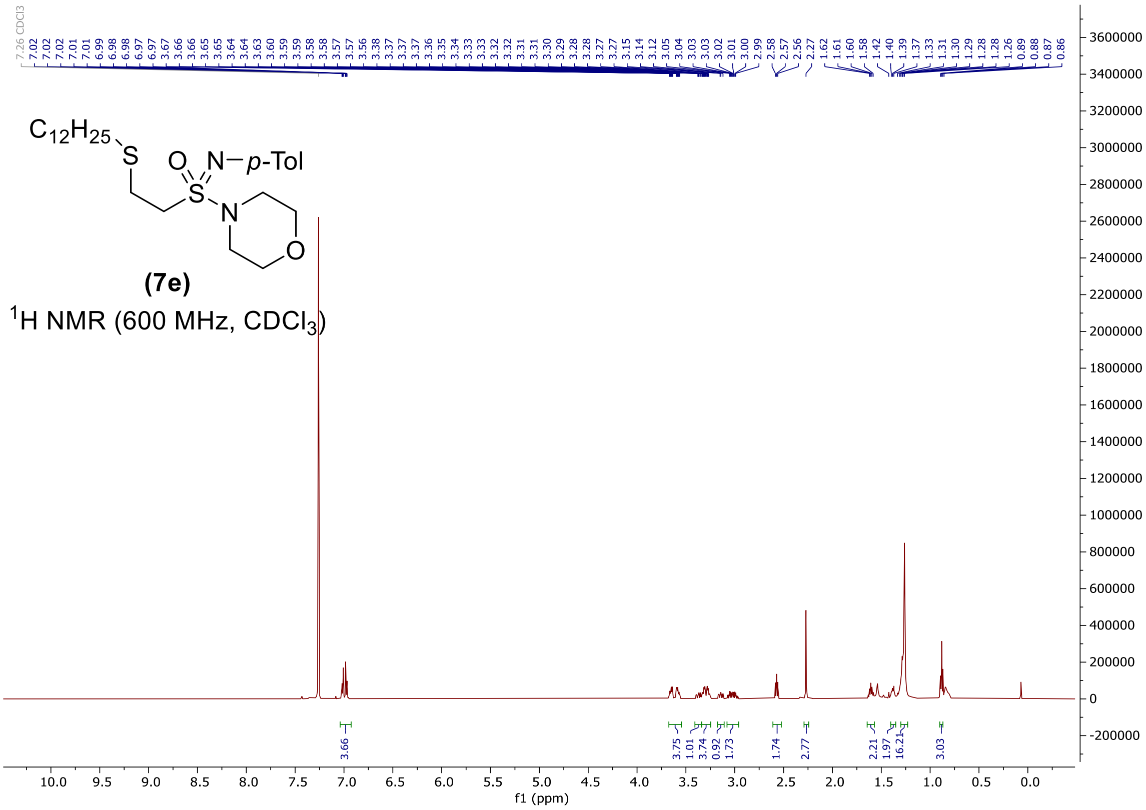

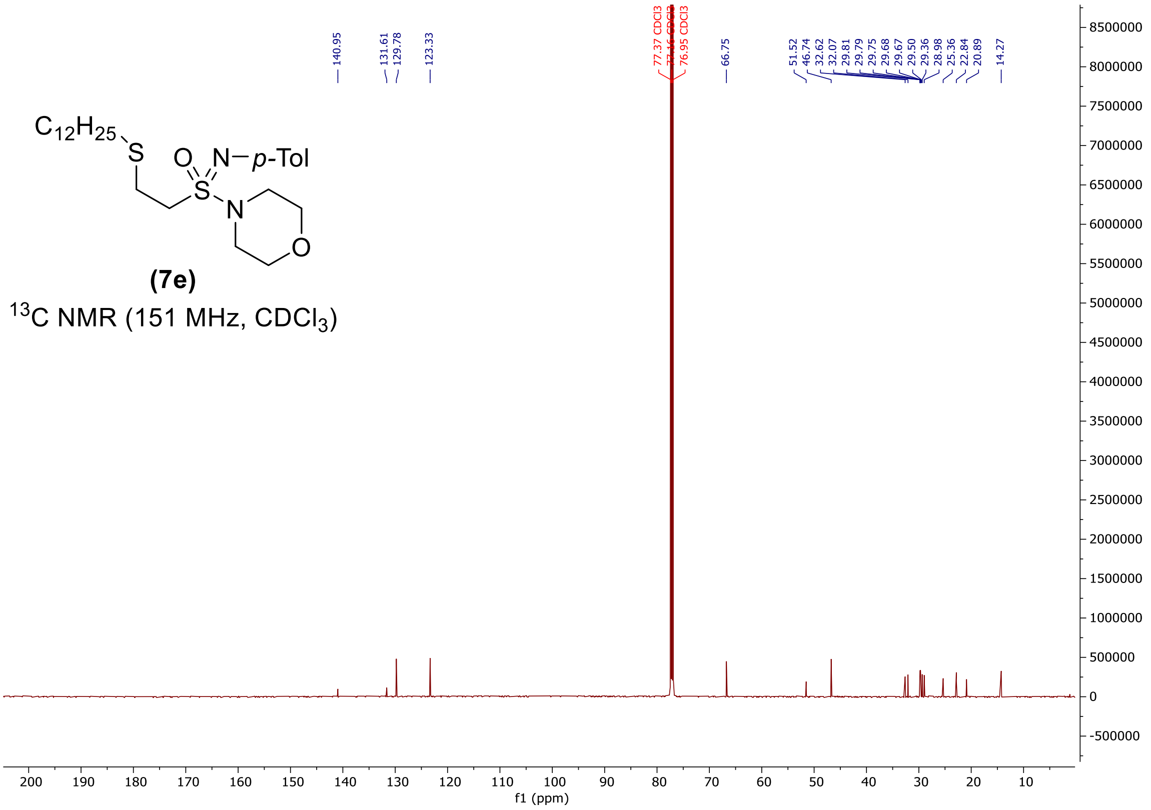

4-(*N*-butyl-2-(dodecylthio)ethylsulfonimidoyl)morpholine (7f)

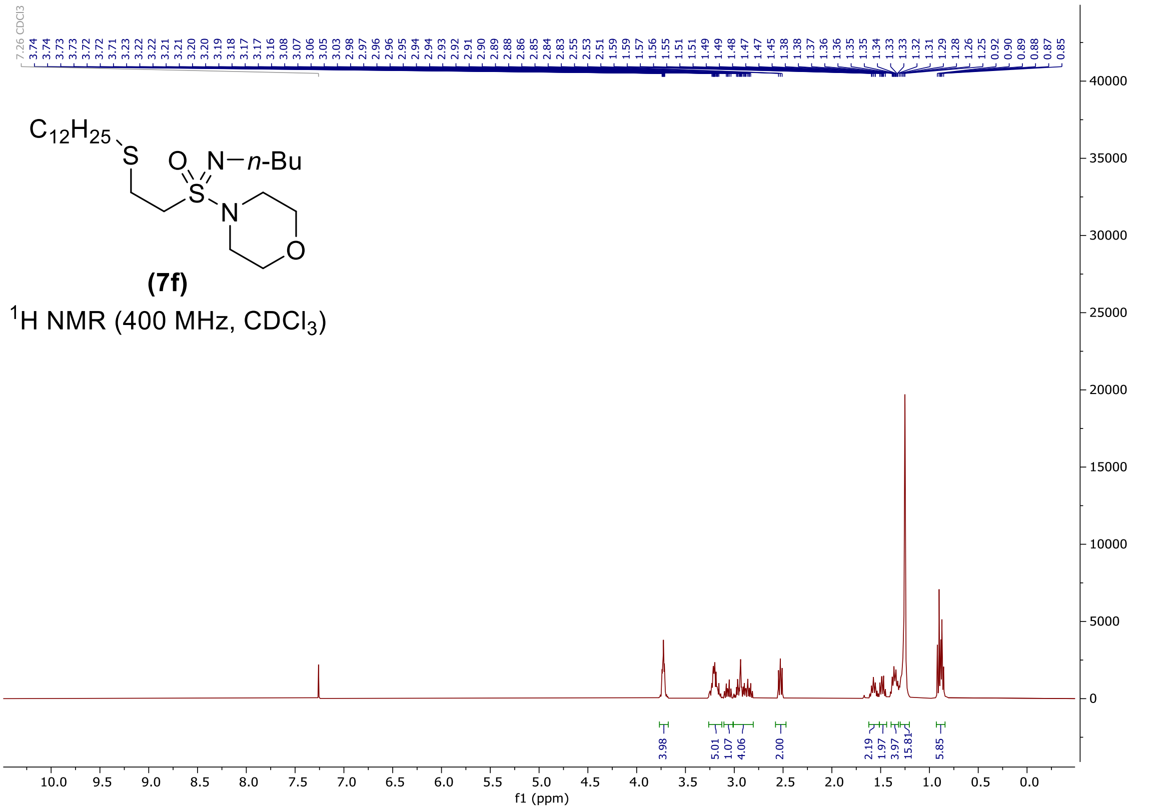

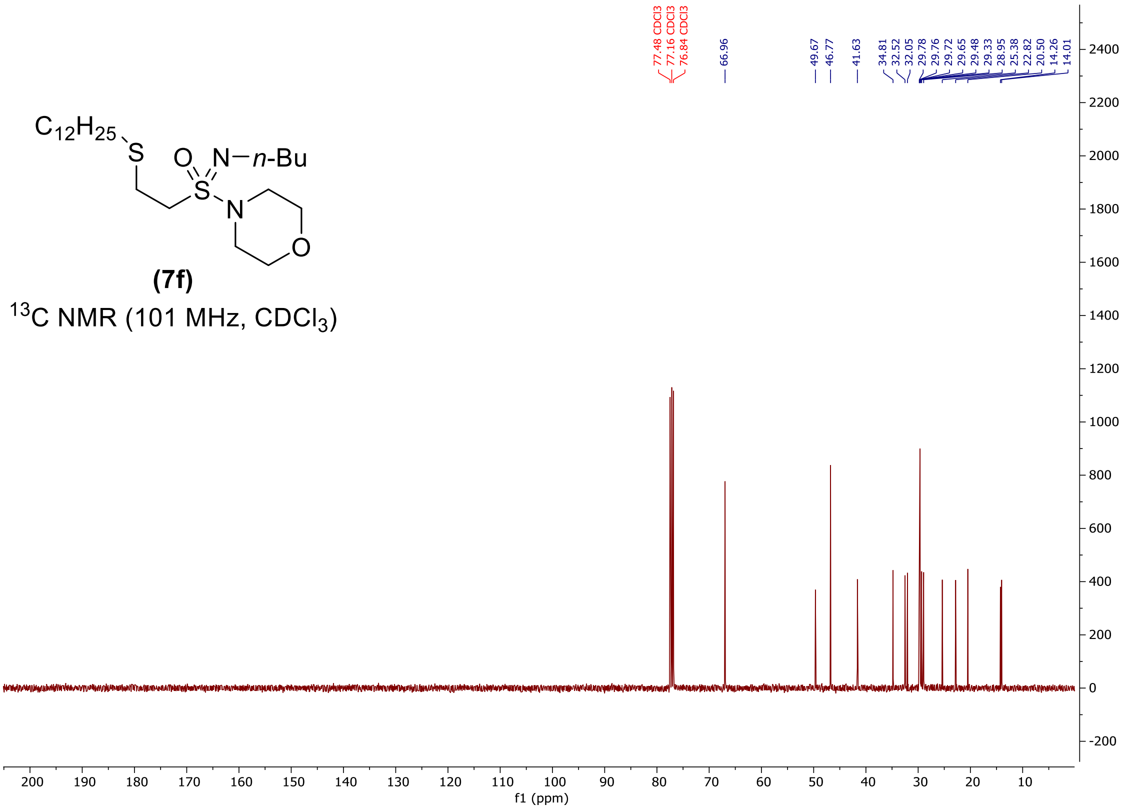

4-(1-(dodecylthio)propan-2-ylsulfonimidoyl)morpholine (7g)

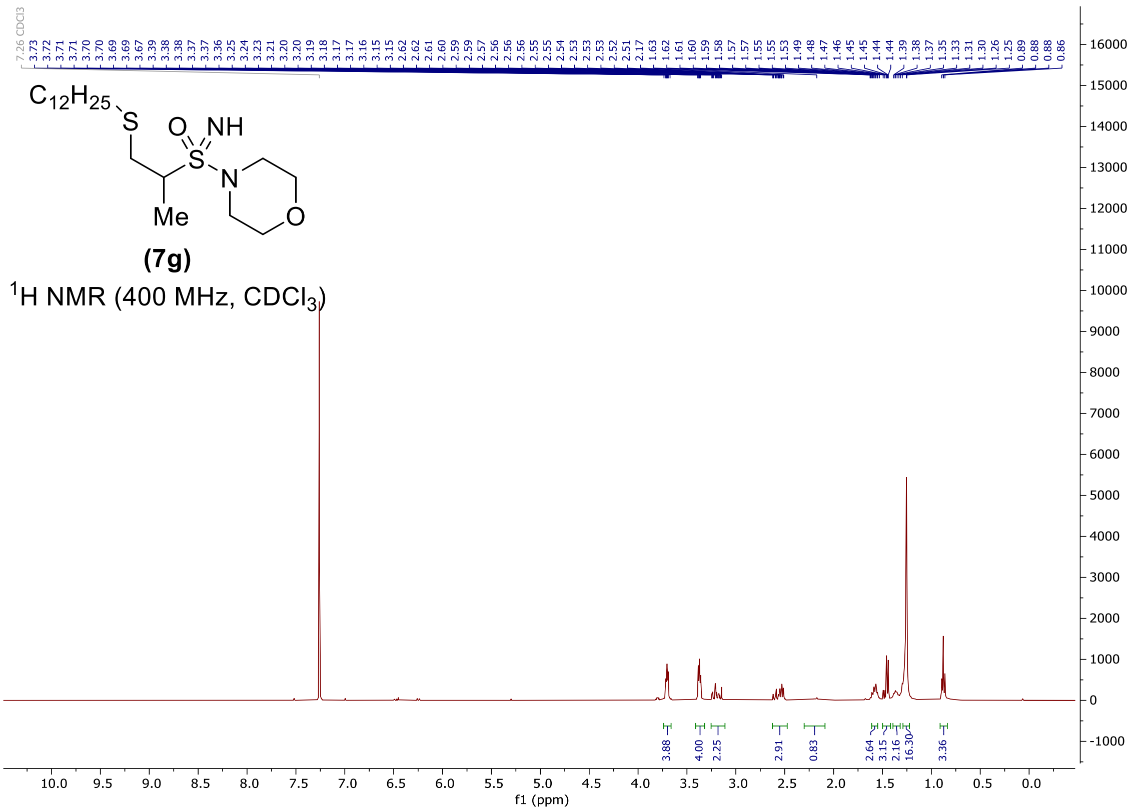

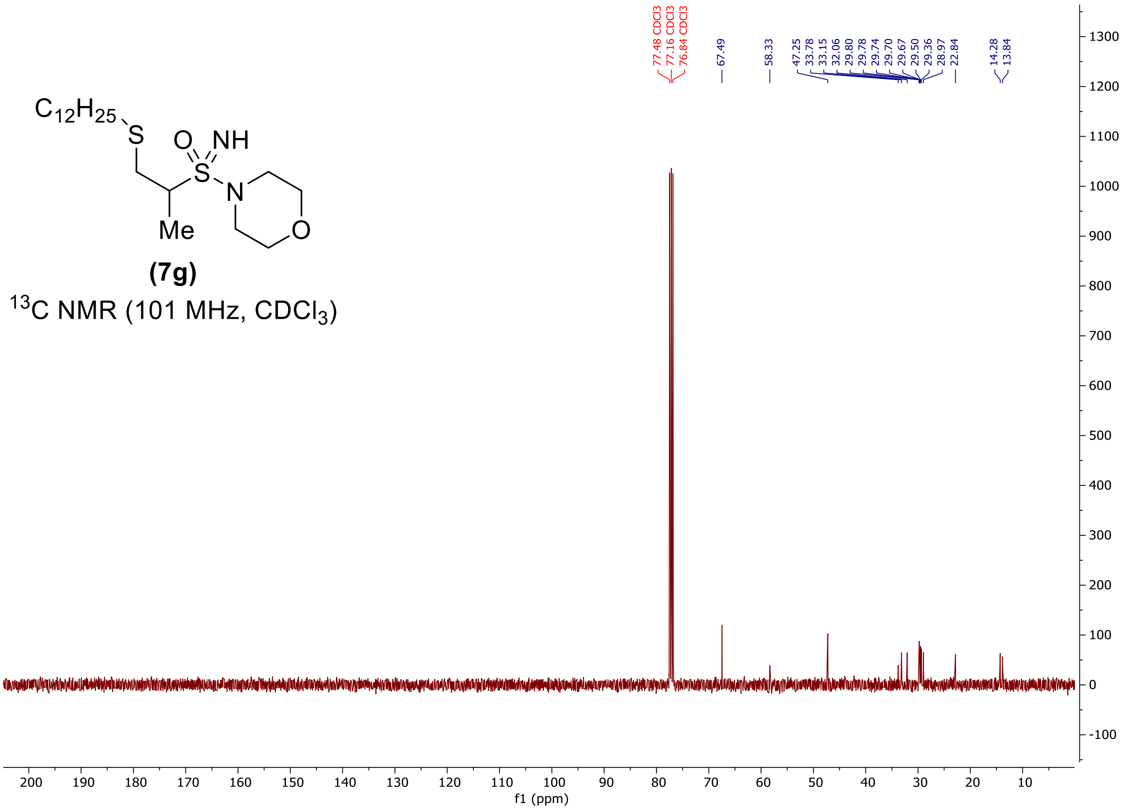

(7g)

^{13}C NMR (101 MHz, $CDCl_3$)

***N*-((1-(dodecylthio)propan-2-yl)(morpholino)(oxo)-l6-sulfaneylidene)methanesulfonamide (7h)**

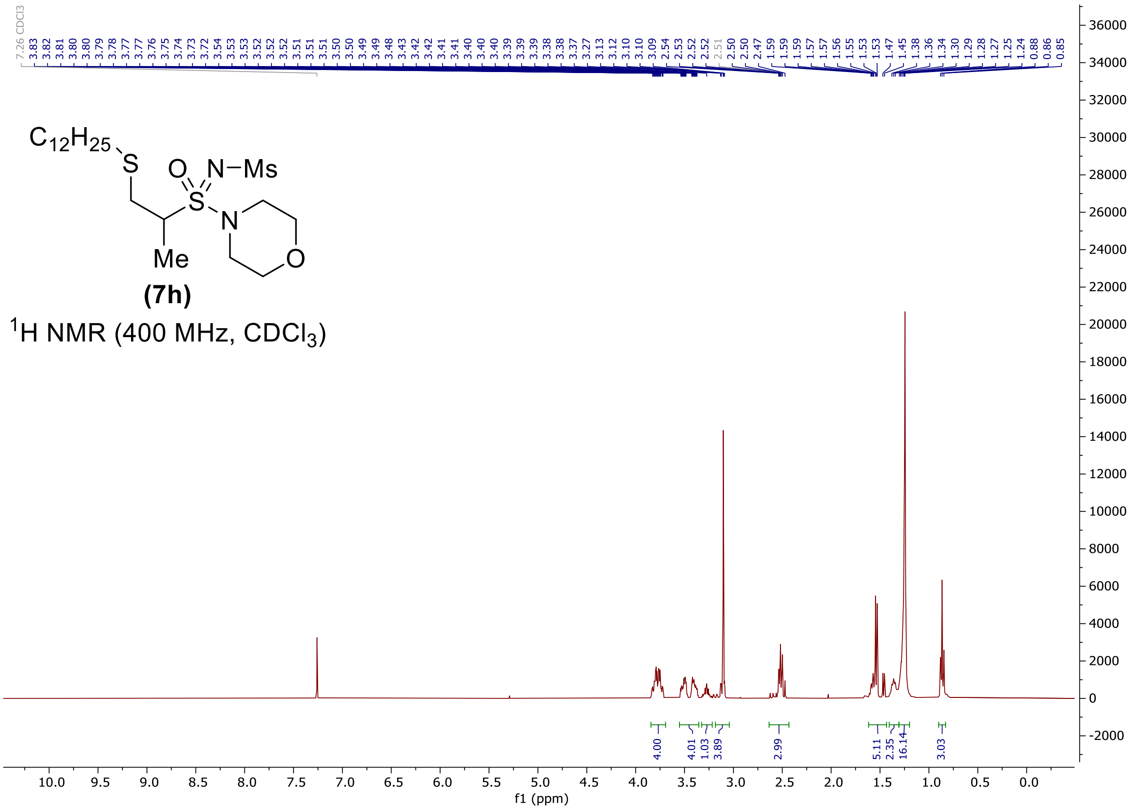

(7h)

^{1}H NMR (400 MHz, $CDCl_3$)

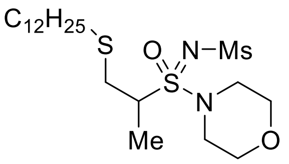

(7h)

^{13}C NMR (101 MHz, $CDCl_3$)

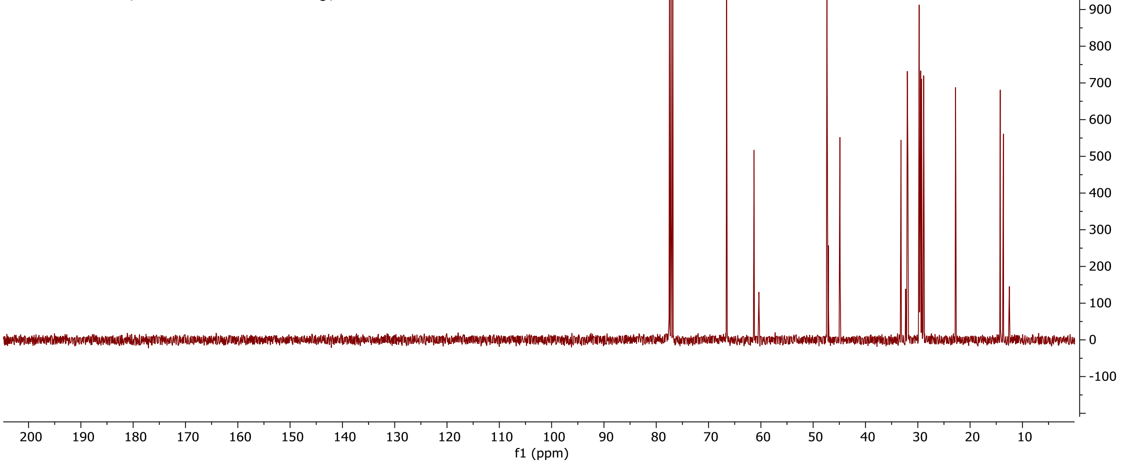

N-((2-(dodecylthio)ethyl)(morpholino)(oxo)-l6-sulfaneylidene)butyramide (7i)

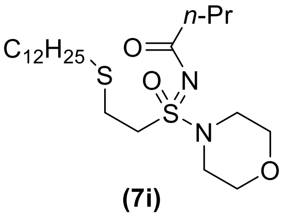

(7i)

^{1}H NMR (400 MHz, $CDCl_3$)

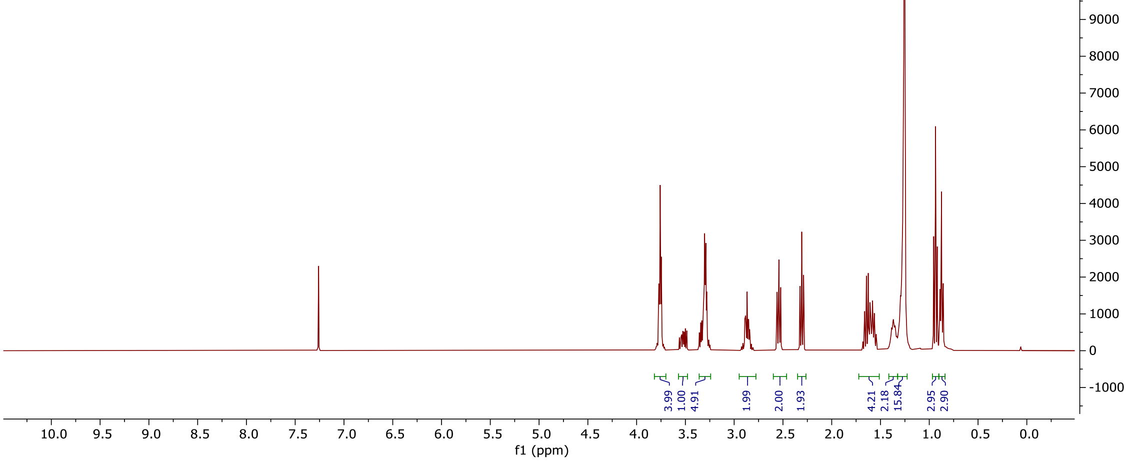

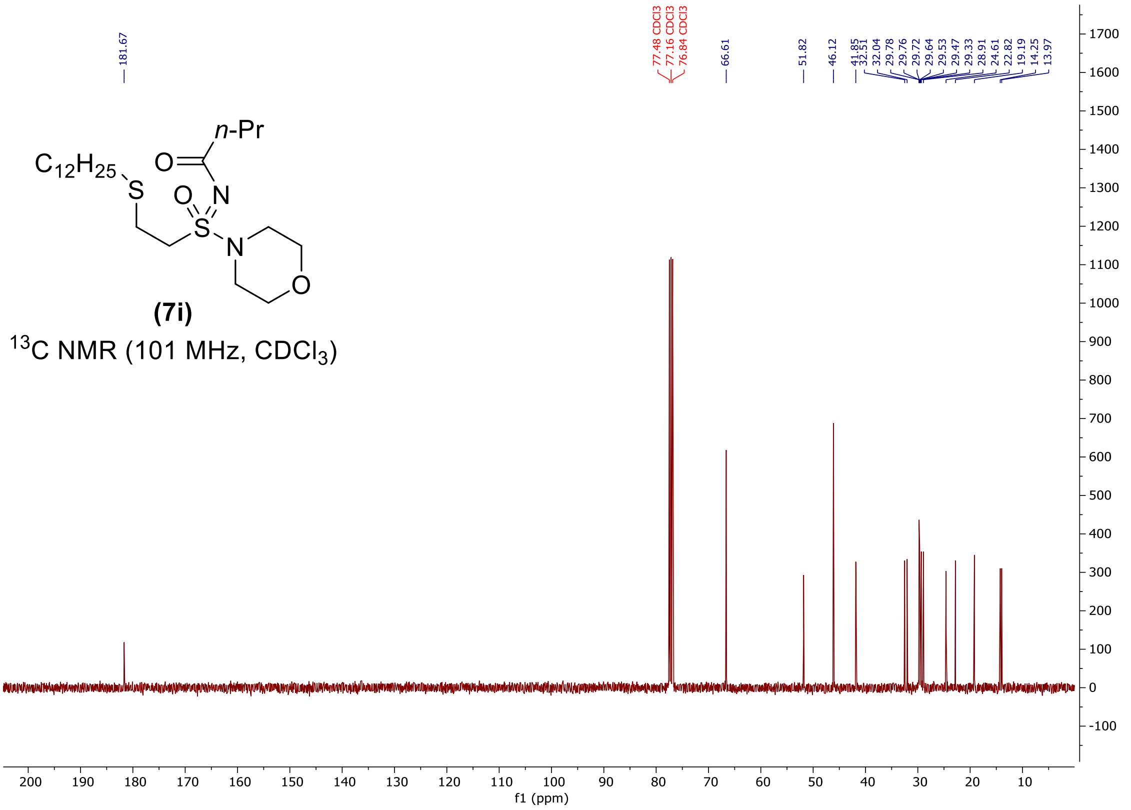

Ethyl ((2-(dodecylthio)ethyl)(morpholino)(oxo)- λ⁶-sulfaneylidene)carbamate (7j)

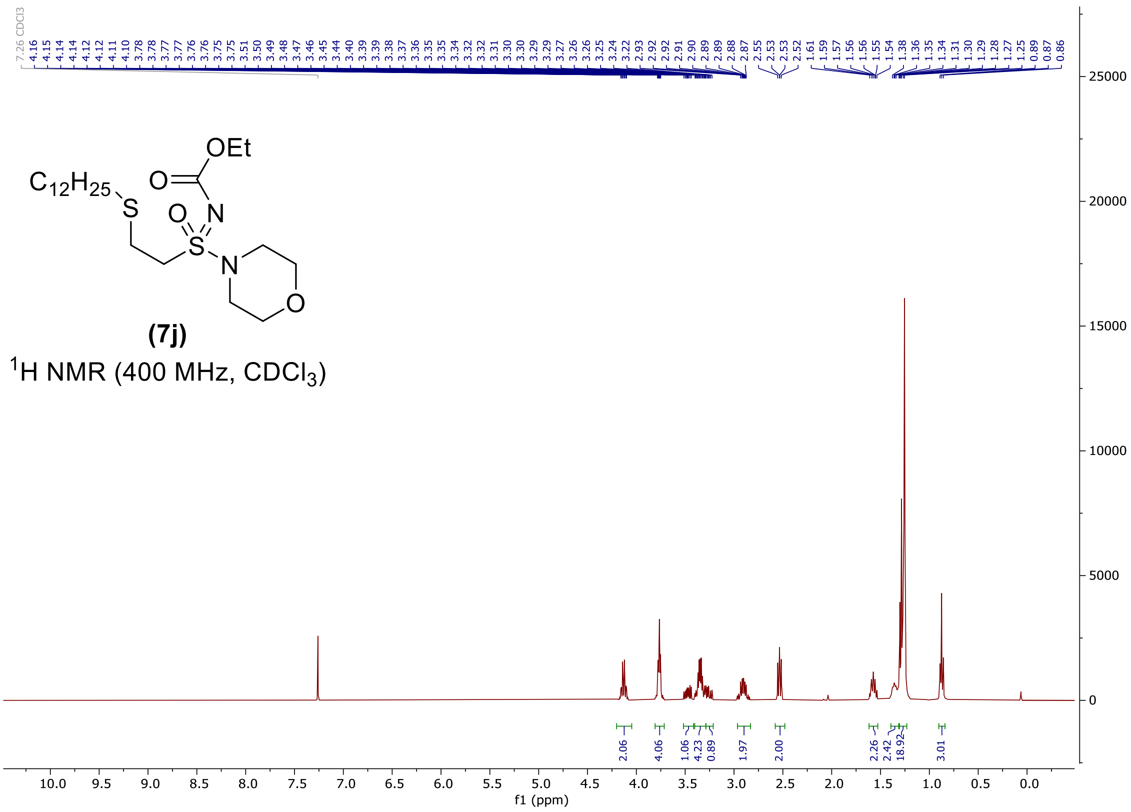

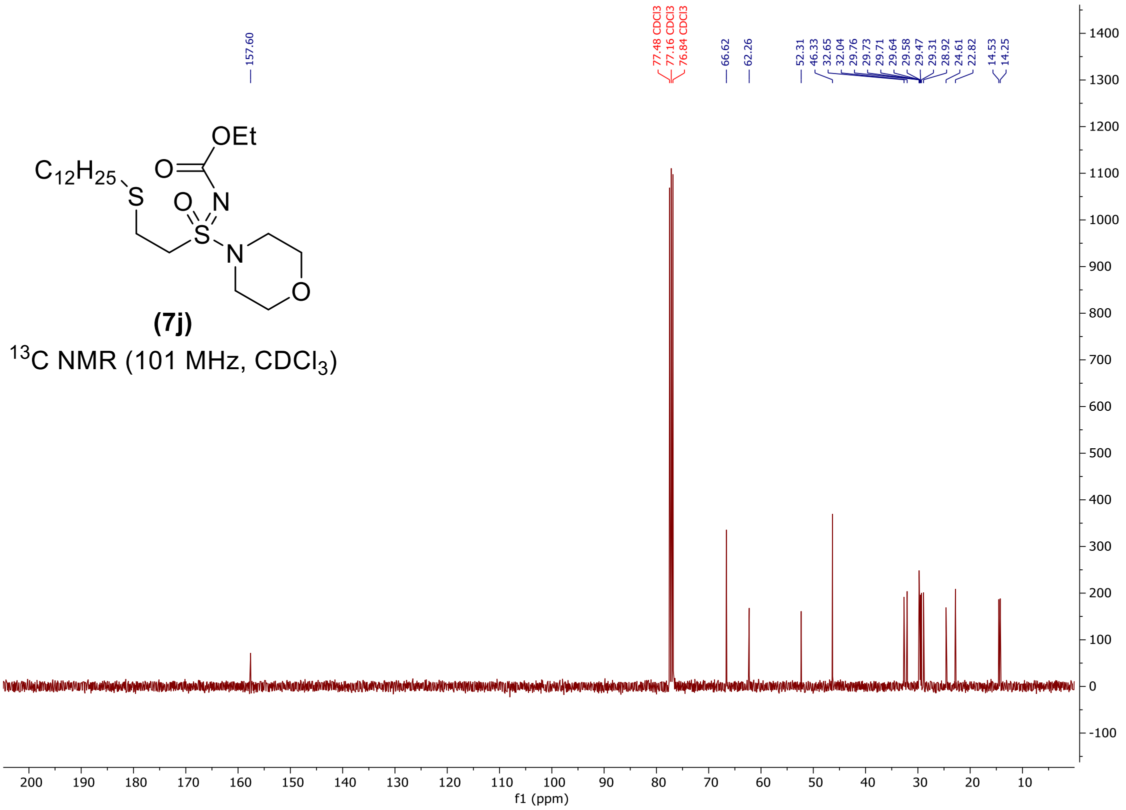

4-(2-(dodecylthio)-N-ethylethylsulfonimidoyl)morpholine (7k)

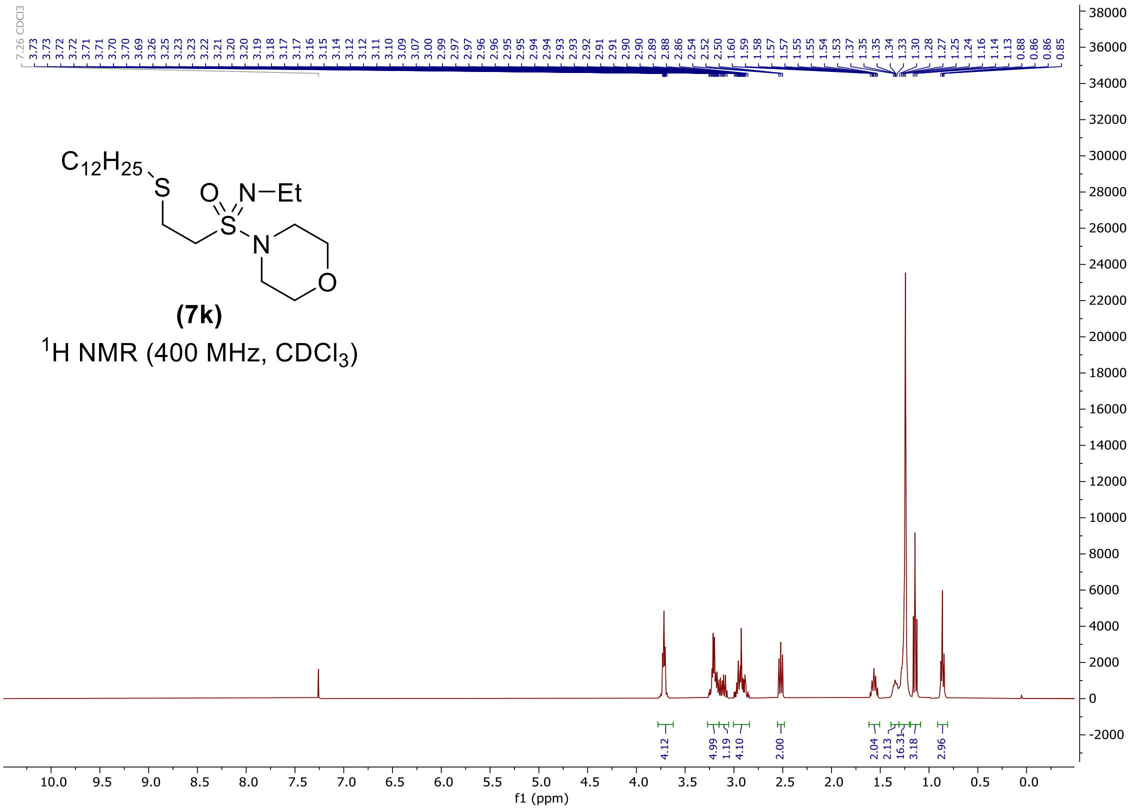

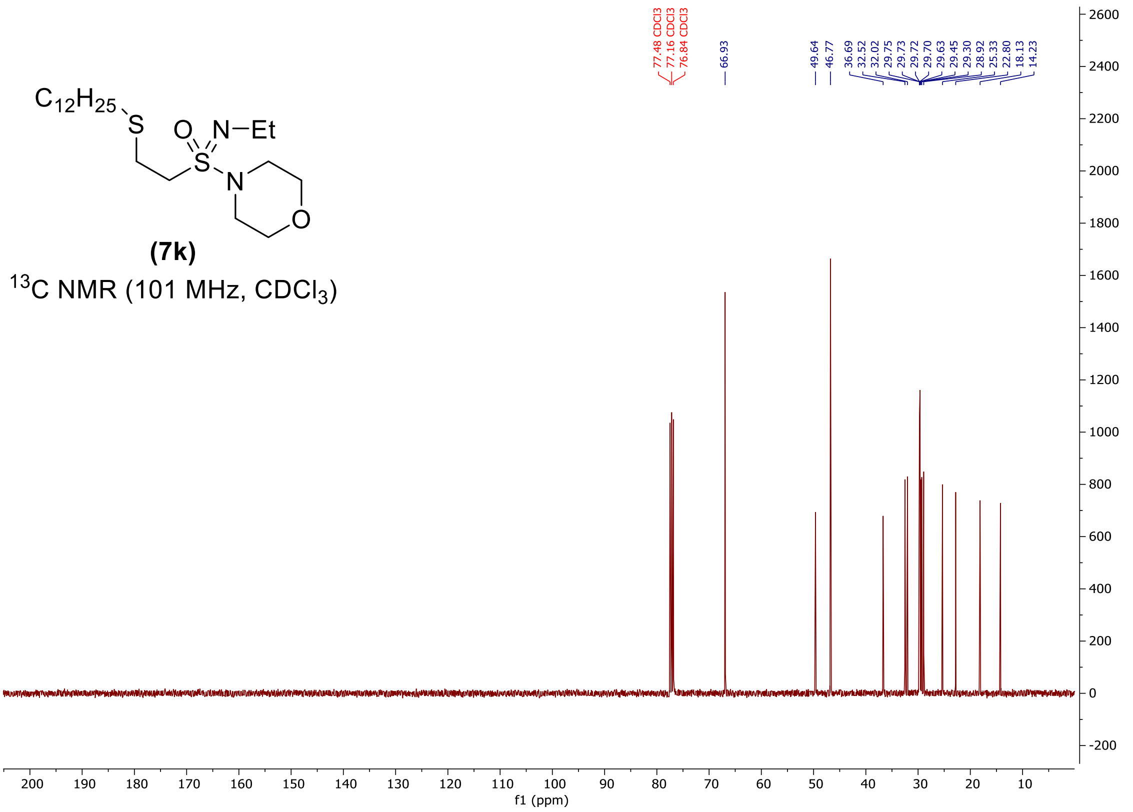

4-(*N*-butyl-1-(dodecylthio)propan-2-ylsulfonimidoyl)morpholine (7l)

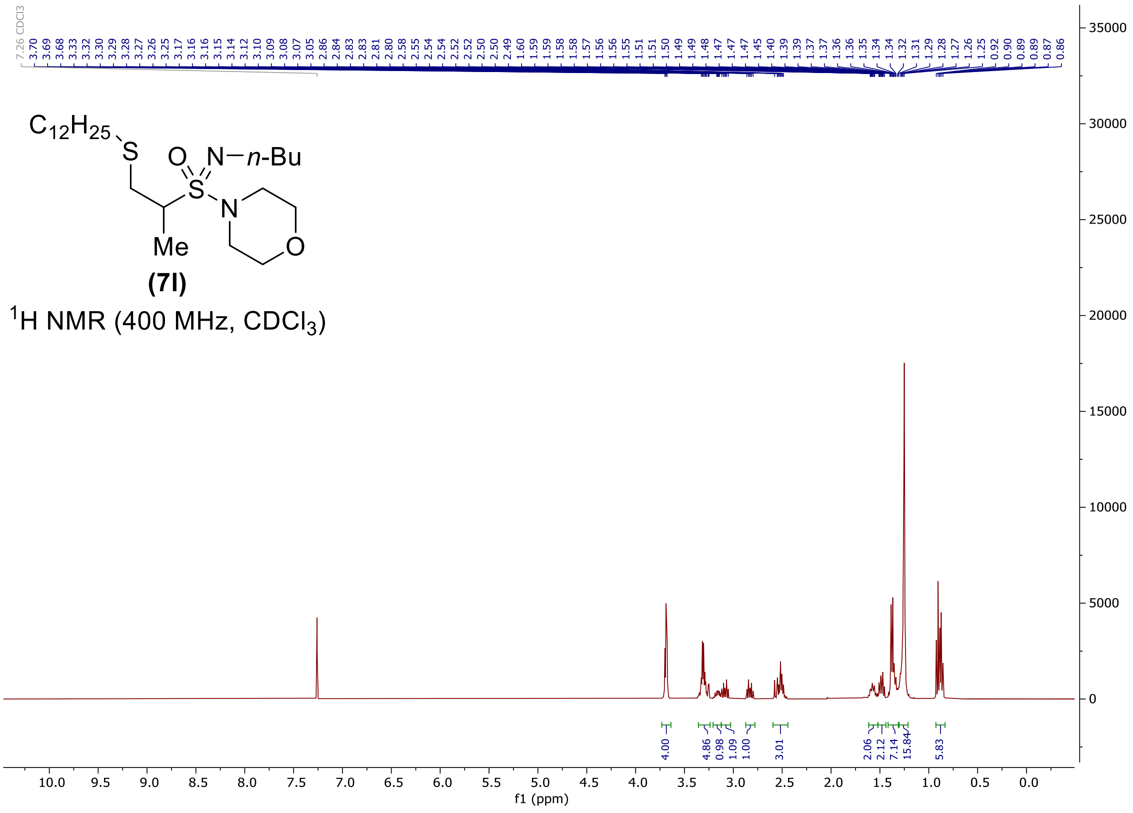

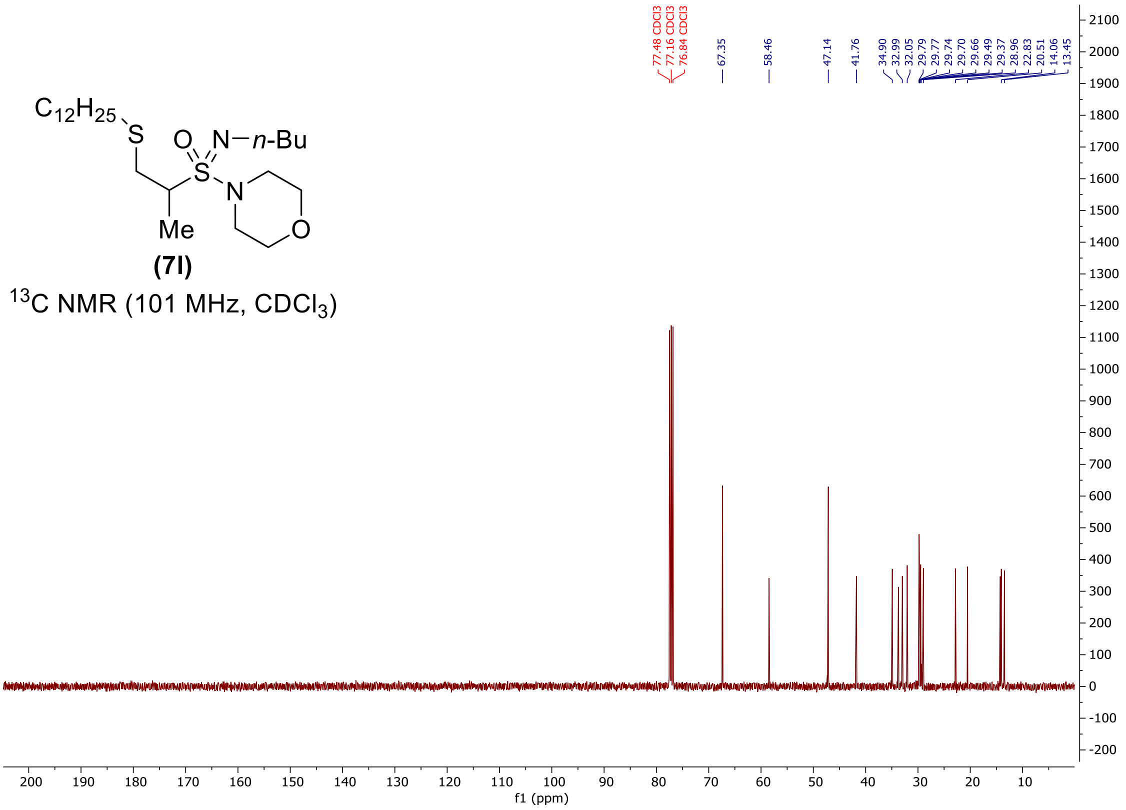

3-(dodecylthio)-1-morpholinopropan-1-one (7m)

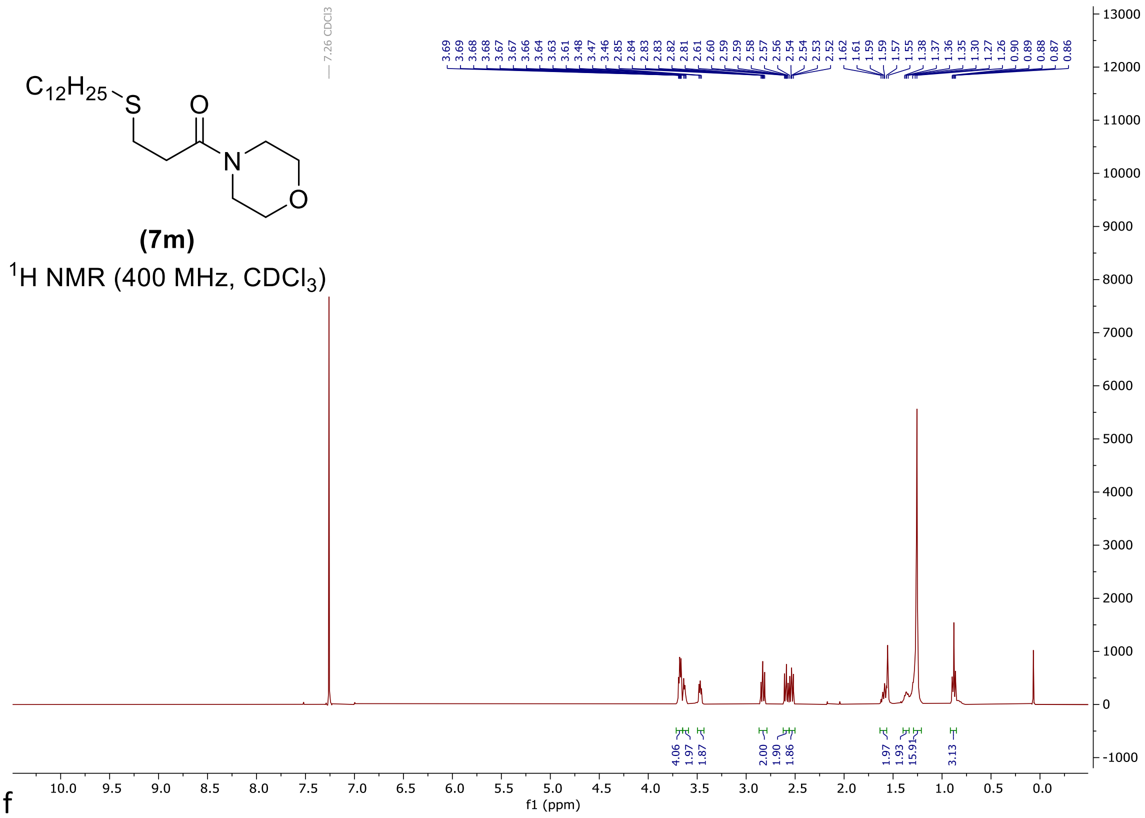

f

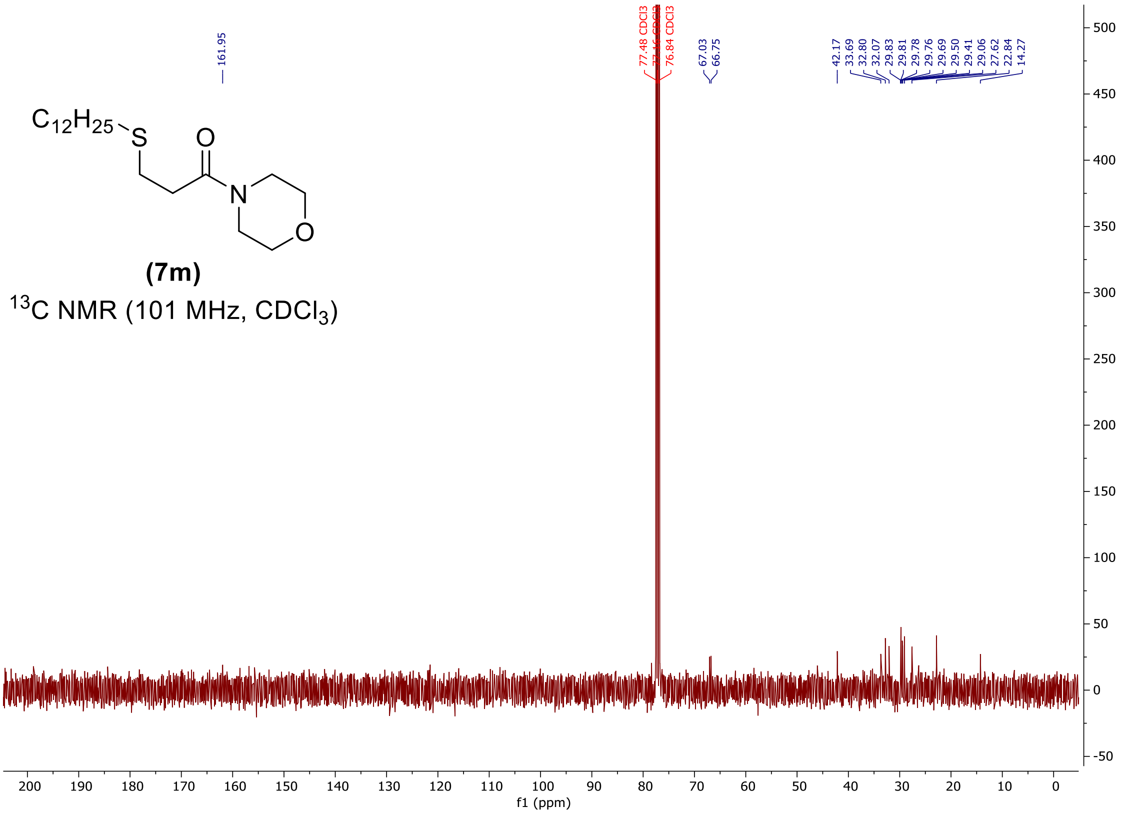

Methyl *N*-(*tert*-butoxycarbonyl)-*S*-(2-(morpholine-4-sulfonimidoyl)ethyl)-*L*-cysteinate (9a)

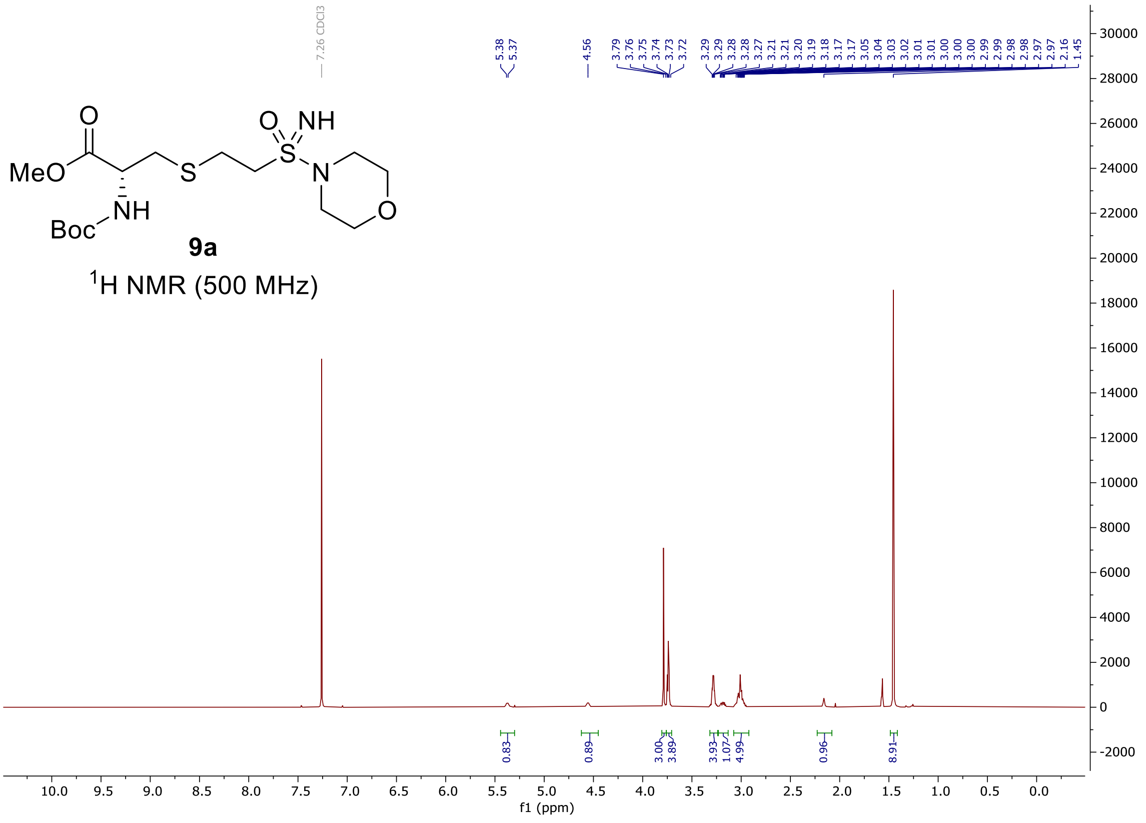

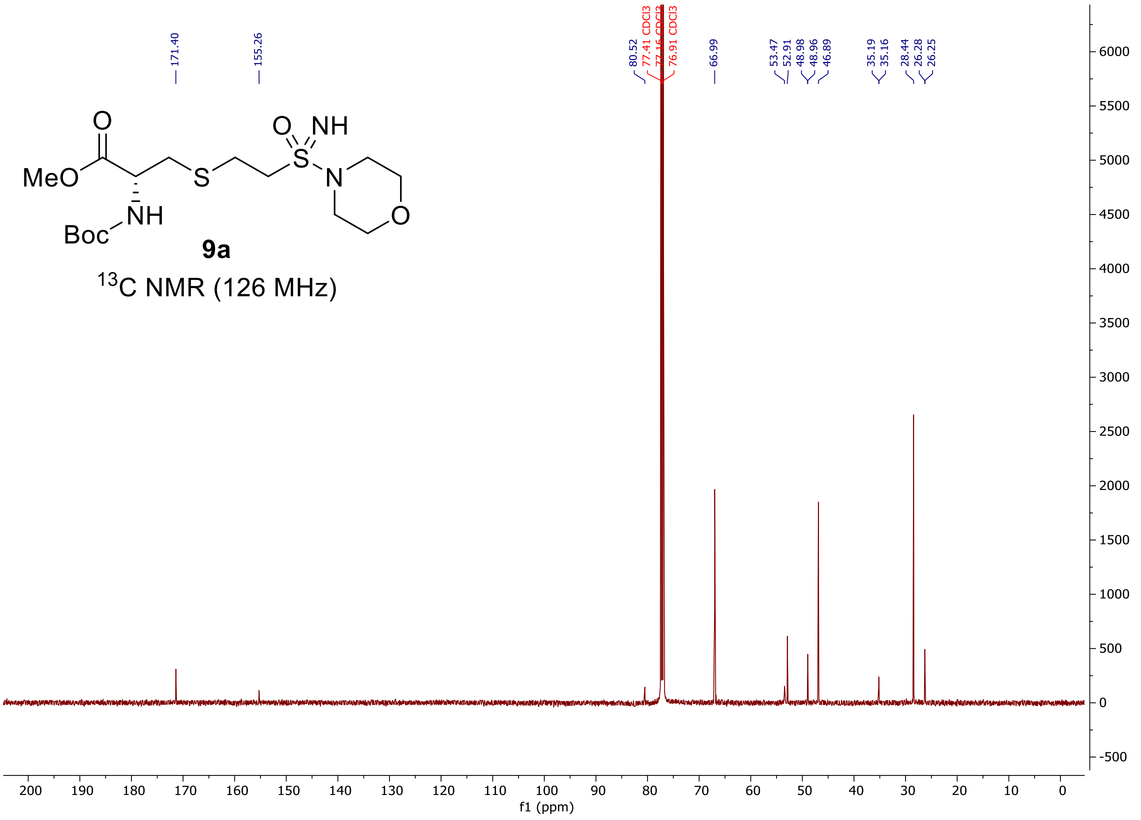

Methyl *N*-(*tert*-butoxycarbonyl)-*S*-(2-(*N*-(methylsulfonyl)morpholine-4-sulfonimidoyl)ethyl)-*L*-cysteinate (9b)

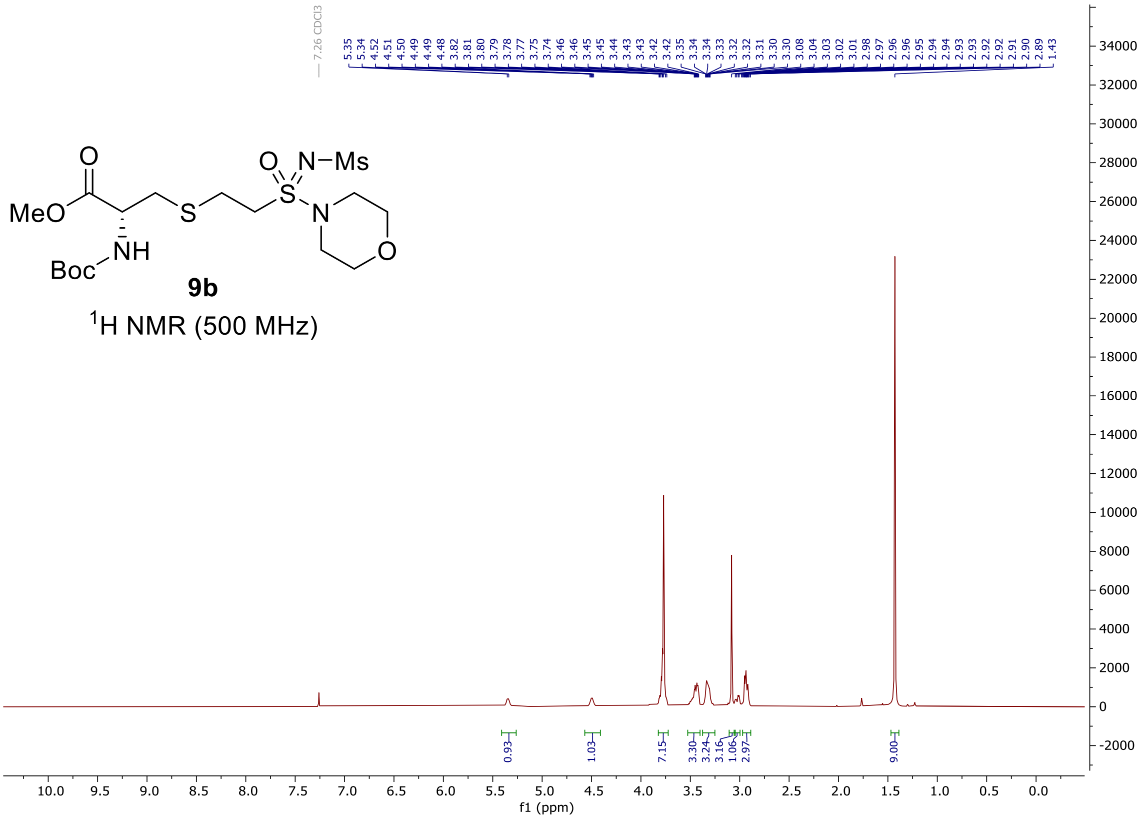

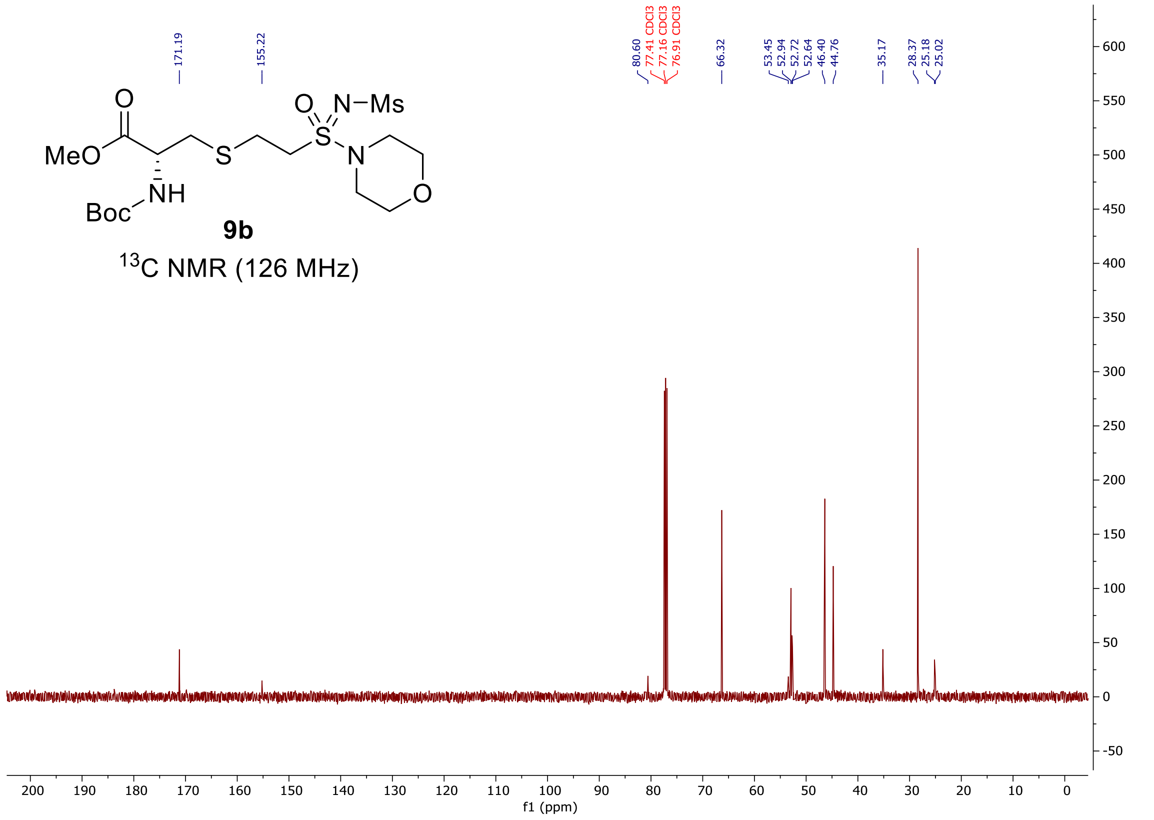

Methyl *N*-(*tert*-butoxycarbonyl)-*S*-(2-(*N*-butylmorpholine-4-sulfonimidoyl)ethyl)-*L*-cysteinate (9c)

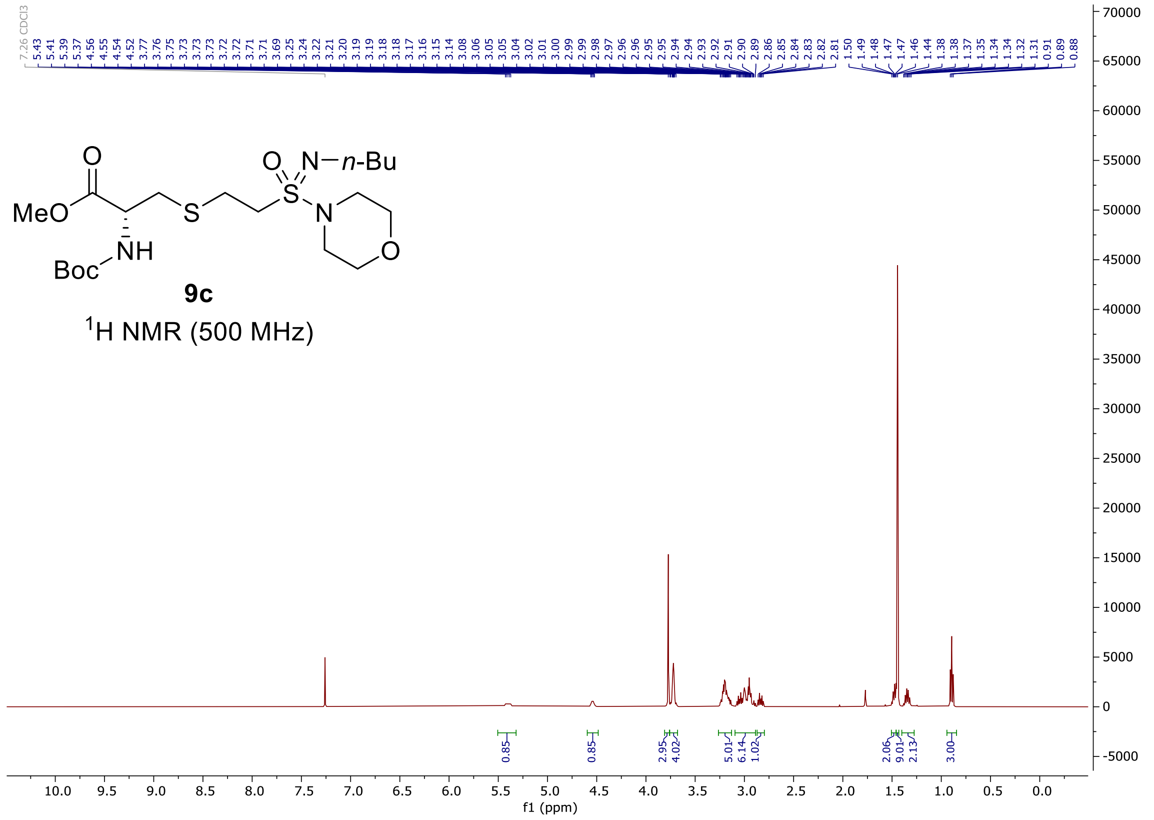

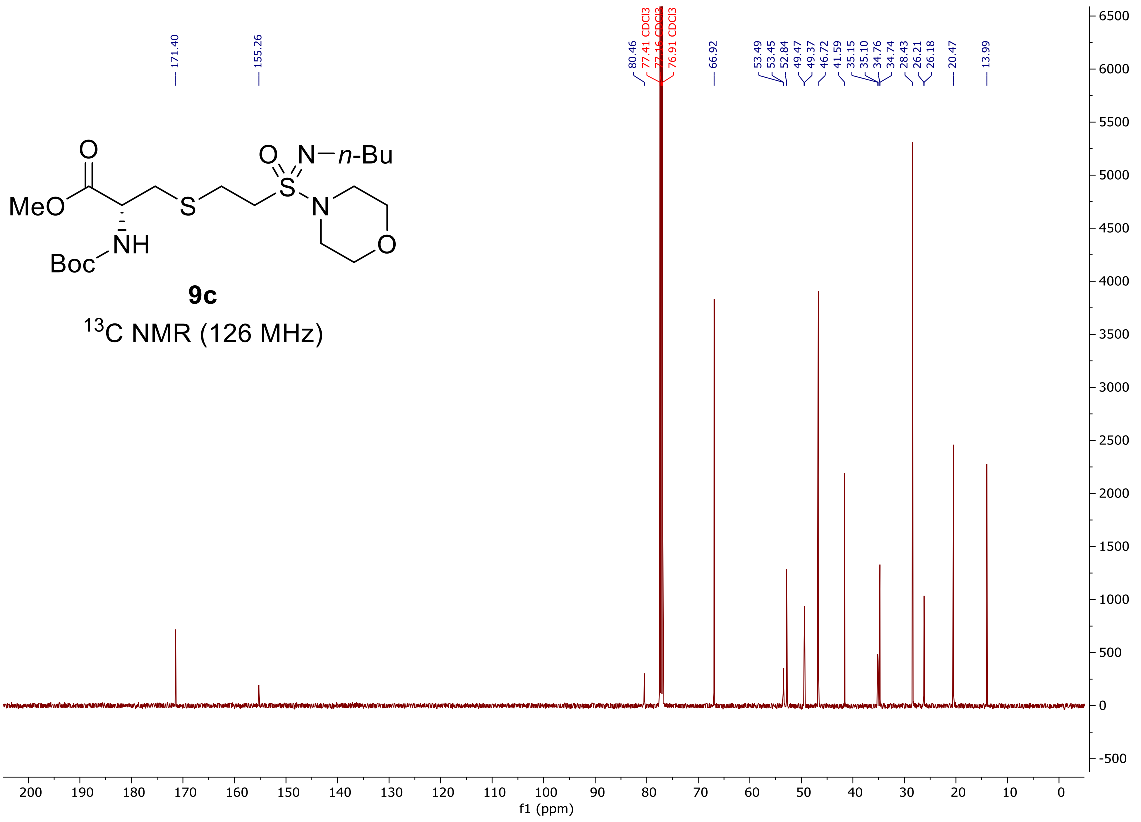

Methyl N²-(tert-butoxycarbonyl)-N⁶-(2-(morpholine-4-sulfonimidoyl)ethyl)-L-lysinate (10a)

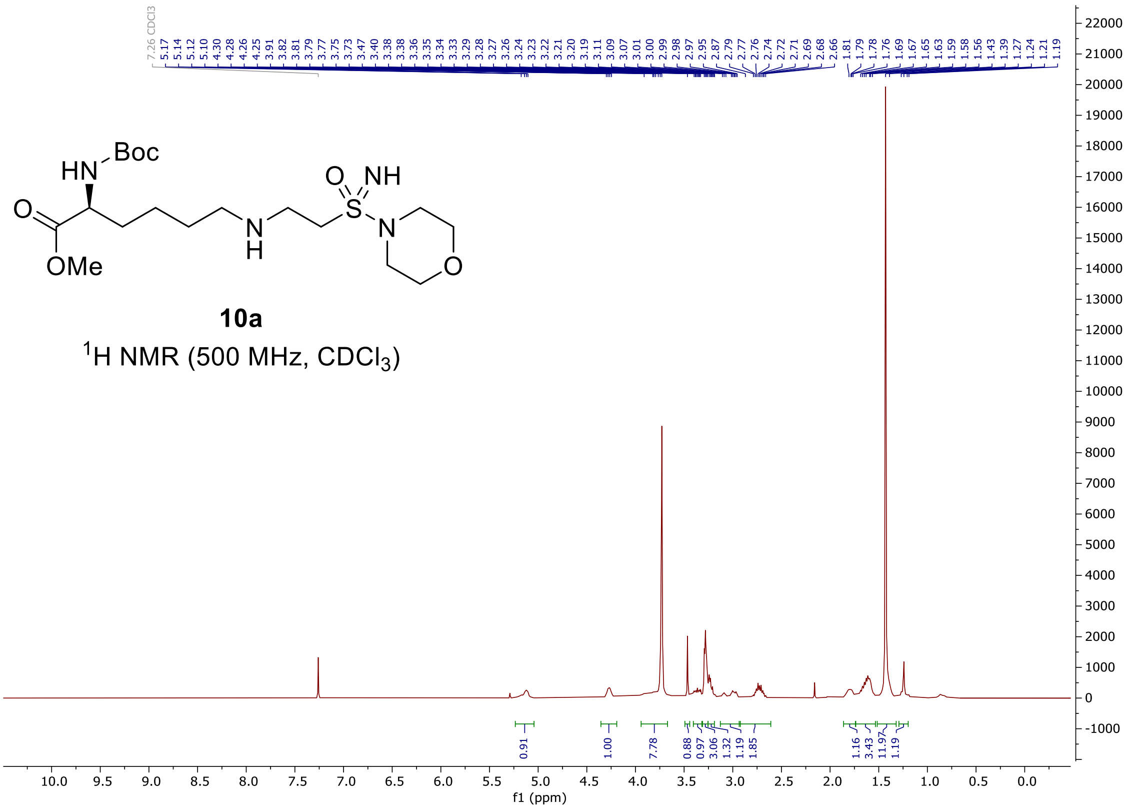

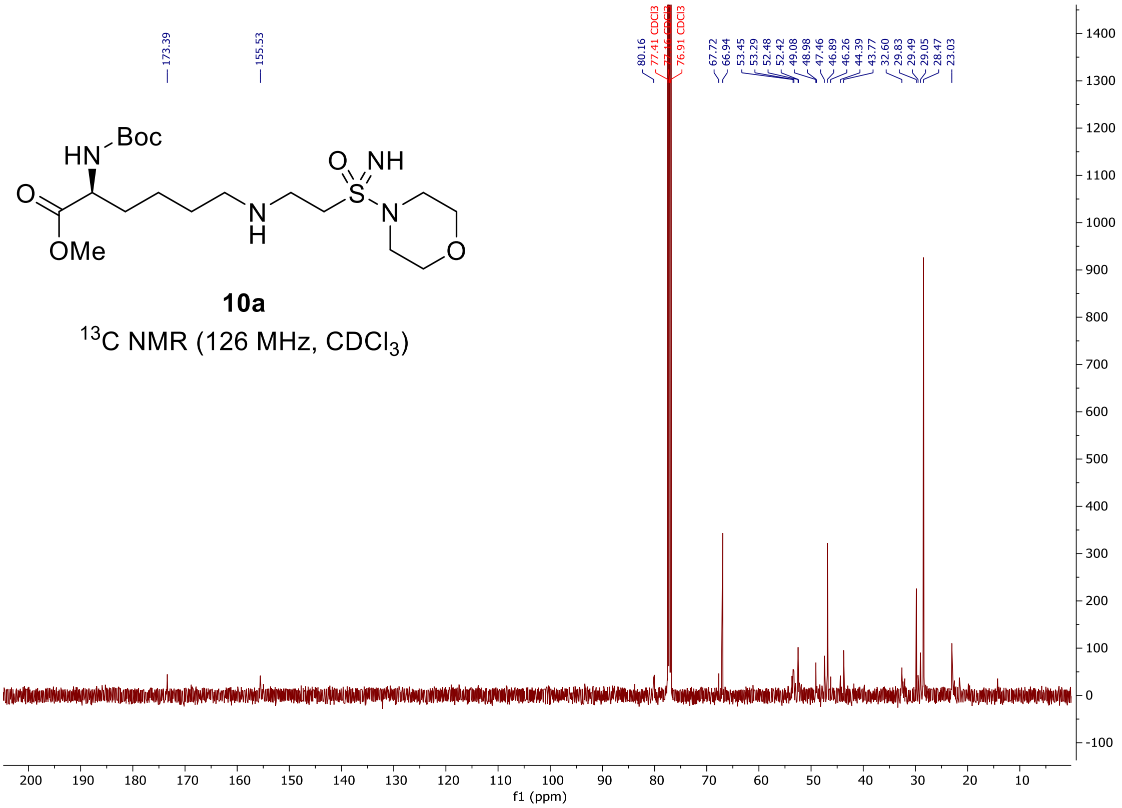

Methyl *N*²-(*tert*-butoxycarbonyl)-*N*⁶,*N*⁶-bis(2-(morpholine-4-sulfonimidoyl)ethyl)-*L*-lysinate (10aa)

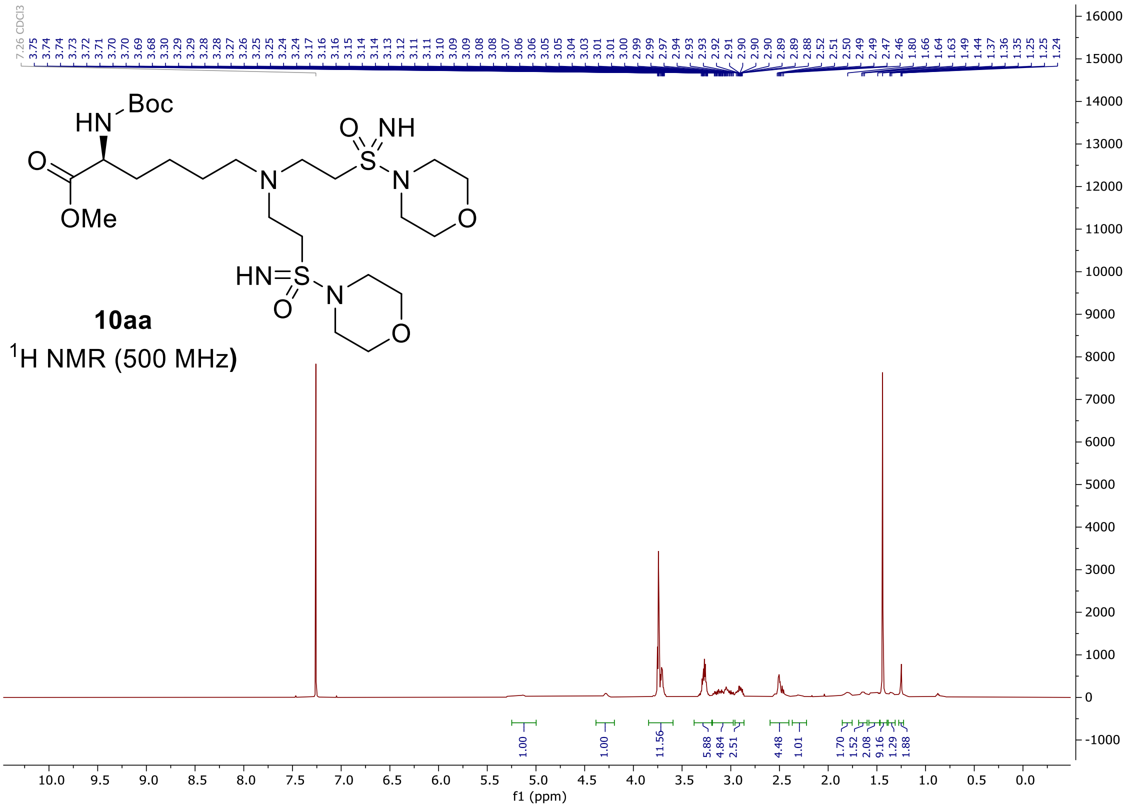

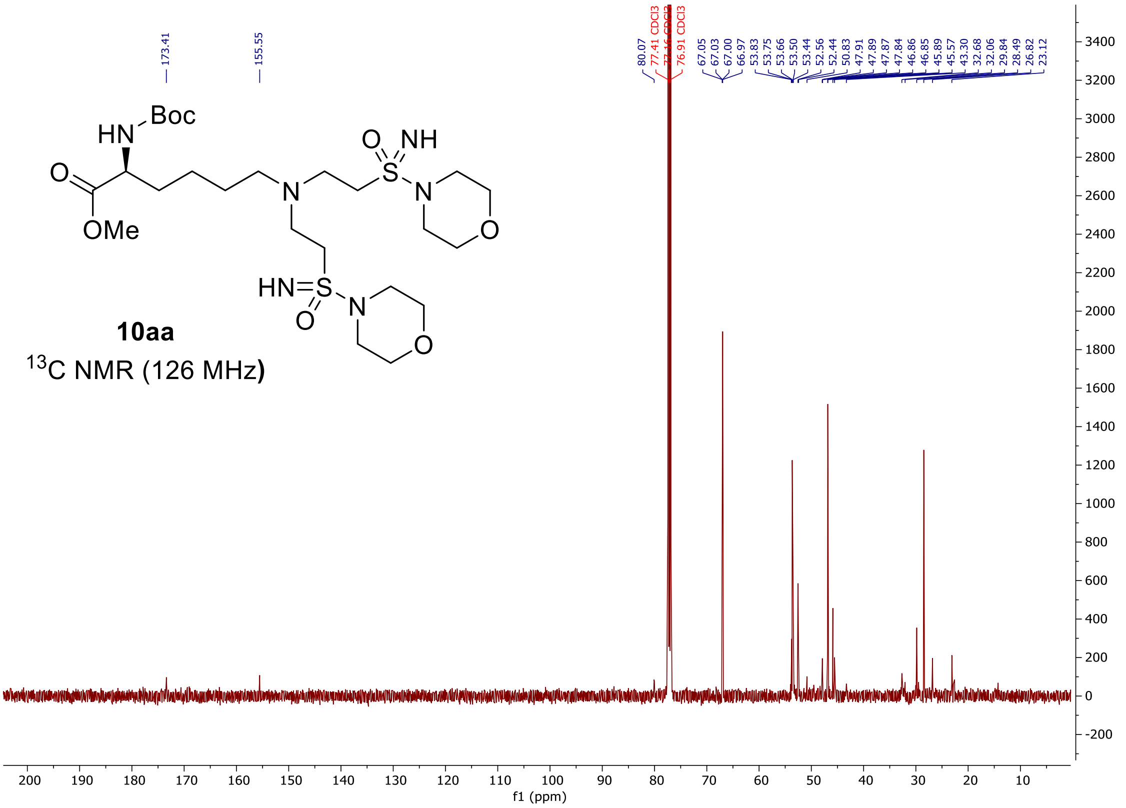

Methyl N^2-(*tert*-butoxycarbonyl)-N^6-(2-(*N*-(methylsulfonyl)morpholine-4-sulfonimidoyl)ethyl)-*L*-lysinate (10b)

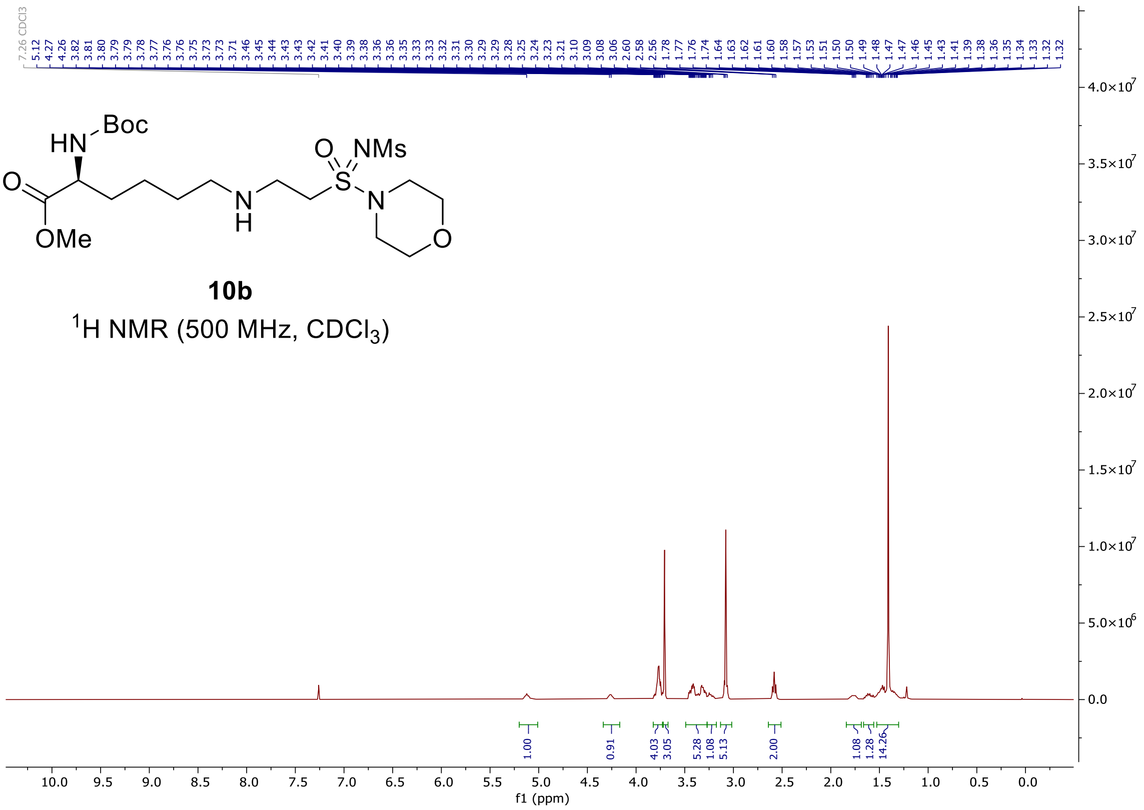

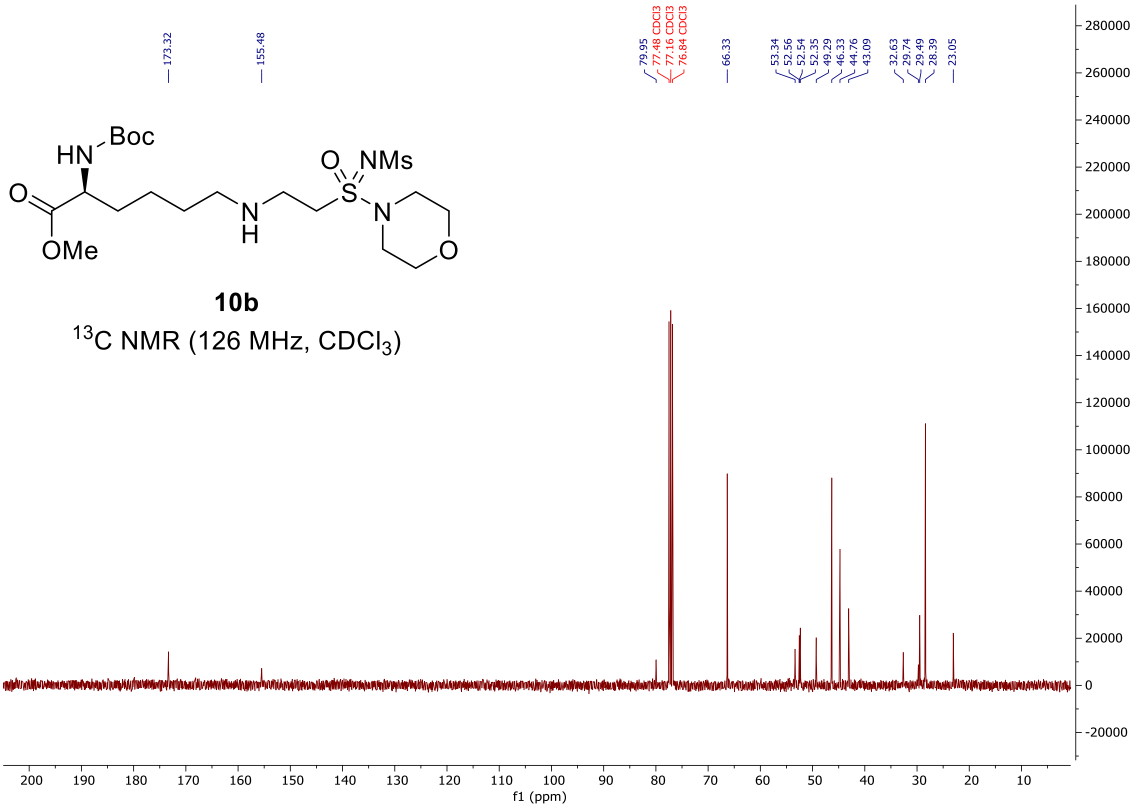

Methyl N^2-(tert-butoxycarbonyl)-N^6-(2-(N-butylmorpholine-4-sulfonimidoyl)ethyl)-L-lysinate (10c)

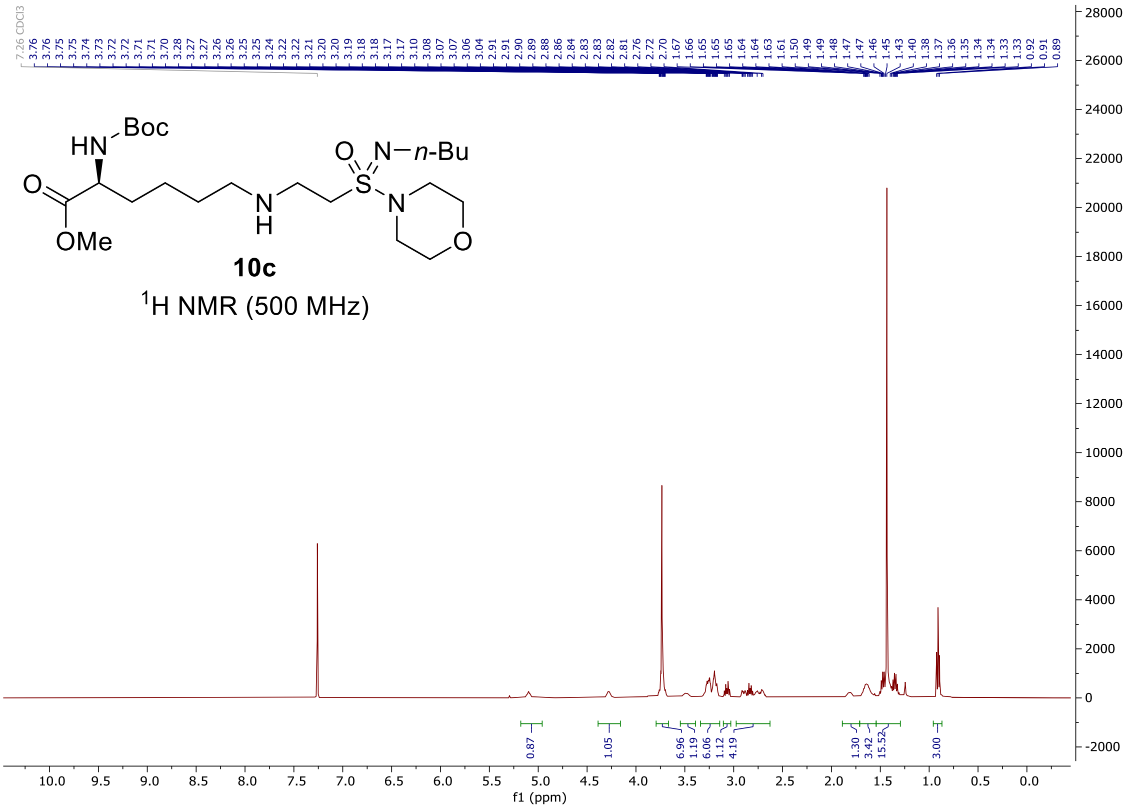

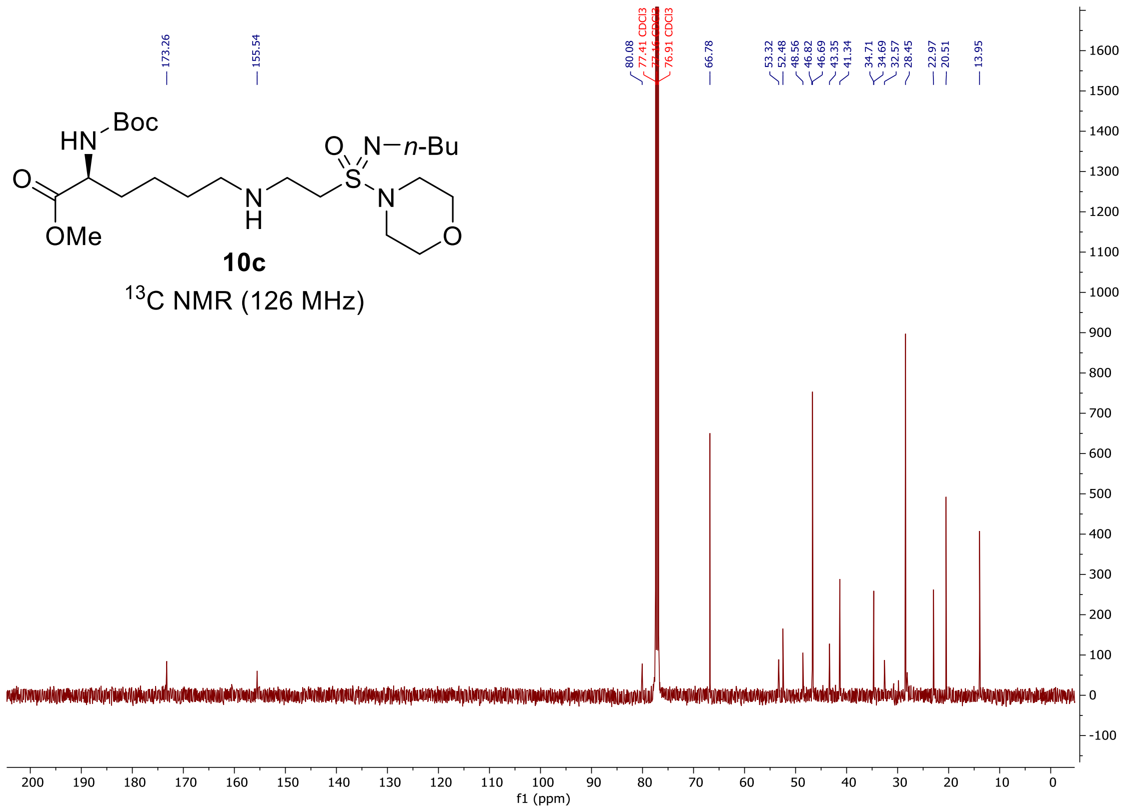

Methyl N^2-(tert-butoxycarbonyl)-N^6,N^6-bis(2-(N-butylmorpholine-4-sulfonimidoyl)ethyl)-L-lysinate (10cc)

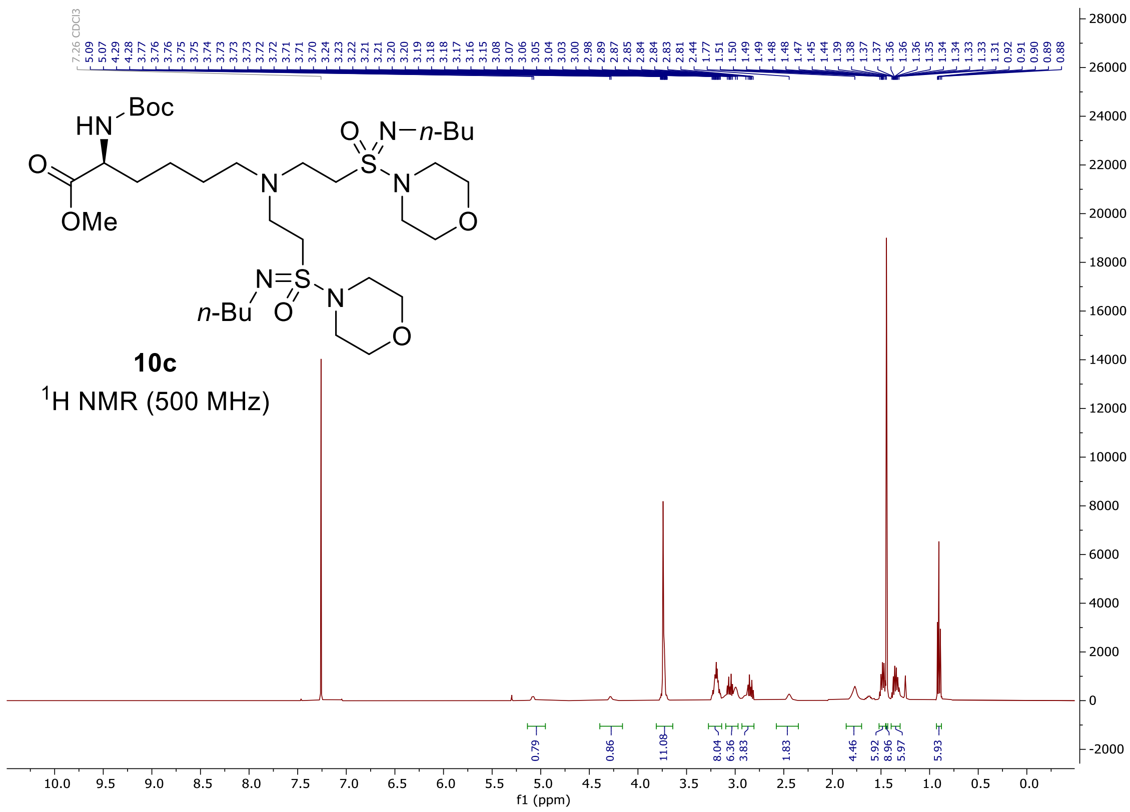

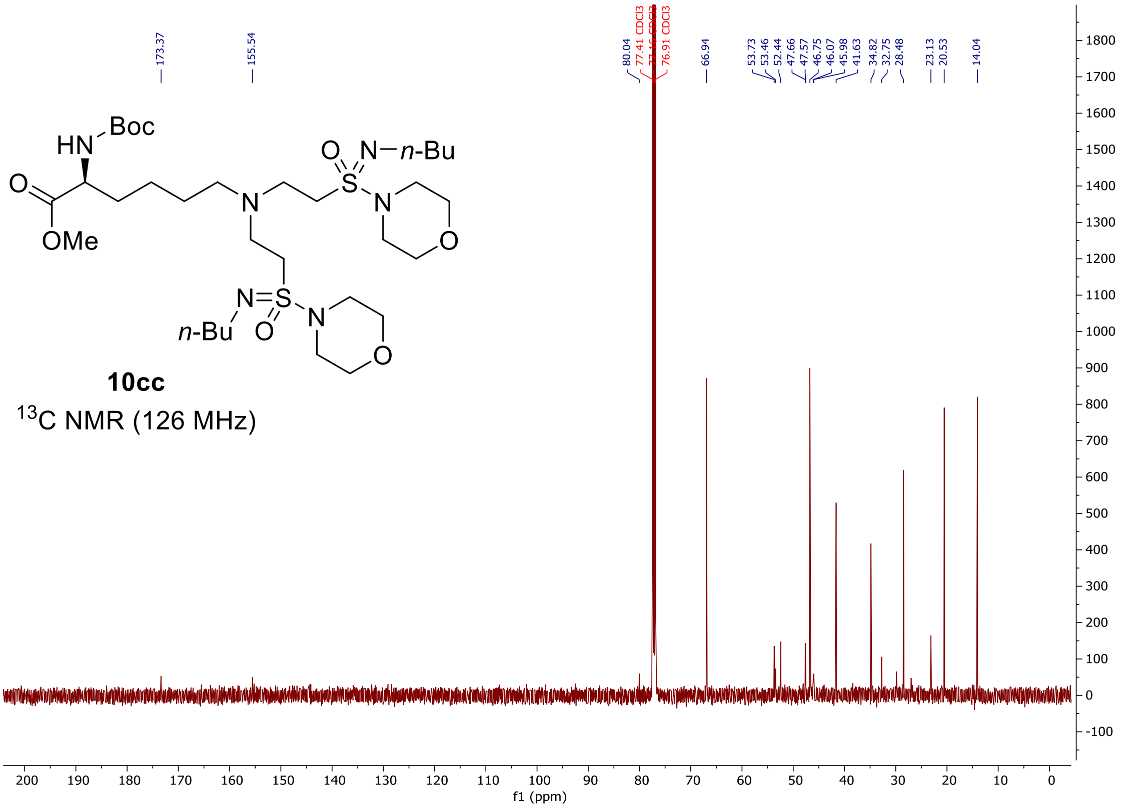

Sulfonimidamide-GSH Adduct (13b)

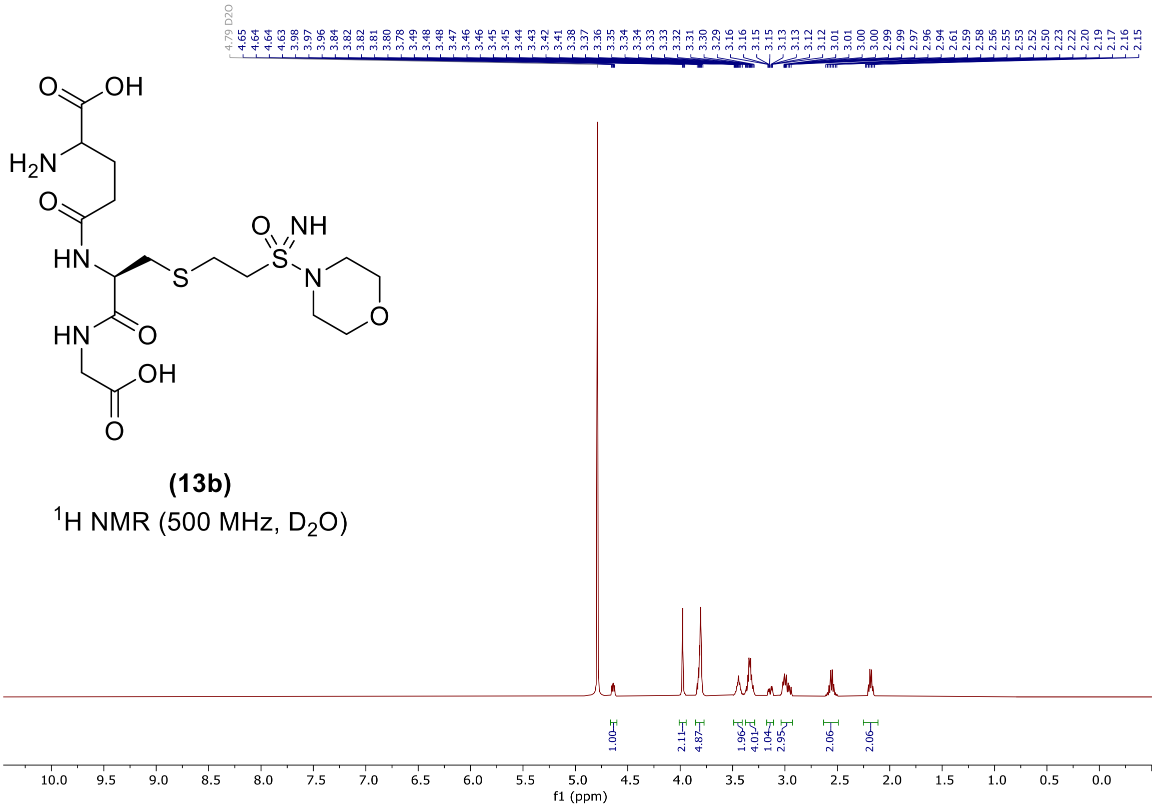

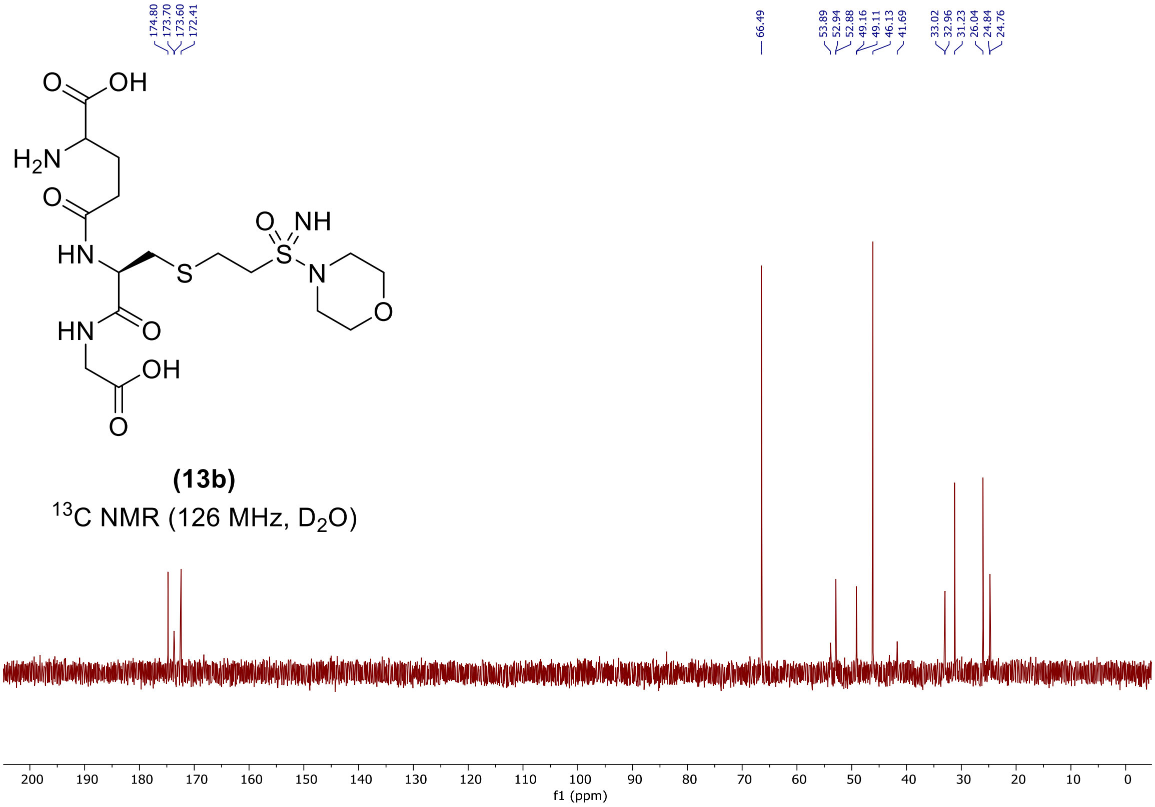

(13b)

^{13}C NMR (126 MHz, D_2O)
